# Intrapleural Perfusion With Staphylococcal Enterotoxin C for Malignant Pleural Effusion: A Clustered Systematic Review and Meta-Analysis

**DOI:** 10.3389/fmed.2022.816973

**Published:** 2022-04-25

**Authors:** Hong Jiang, Xue-Mei Yang, Cheng-Qiong Wang, Jiao Xu, Jun Huang, Ji-Hong Feng, Xiao-Fan Chen, Kai Chen, Lin Zhan, Xue Xiao, Zheng Xiao

**Affiliations:** ^1^Department of General Practice, Affiliated Hospital of Zunyi Medical University, Zunyi, China; ^2^Evidence-Based Medicine Center, MOE Virtual Research Center of Evidence-Based Medicine at Zunyi Medical College, Affiliated Hospital of Zunyi Medical University, Zunyi, China; ^3^Department of Pharmacy, Affiliated Hospital of Zunyi Medical University, Zunyi, China; ^4^Department of Oncology, Lishui People's Hospital, Sixth Affiliated Hospital of Wenzhou Medical University, Lishui, China; ^5^Evidence-Based Medicine Research Centre, Jiangxi University of Traditional Chinese Medicine, Nanchang, China; ^6^Department of Surgery, First Affiliated Hospital of Chongqing Medical and Pharmaceutical College, Chongqing, China; ^7^Laboratory Research Center, Guizhou Provincial People's Hospital, Guizhou University, Guiyang, China

**Keywords:** malignant pleural effusion (MPE), staphylococcal enterotoxin C (SEC), intrapleural infusion, pleurodesis agent, clustered systematic review, meta-analysis

## Abstract

**Introduction:**

The staphylococcal enterotoxin C (SEC), a commercially available bio-product from *Staphylococcus aureus* (*S. aureus*), has been widely used to control MPE.

**Objectives:**

We designed and performed a new systematic review (SR) and meta-analysis to clarify the perfusion protocols with SEC, determine their clinical effectiveness and safety, and reveal the indication and optimum usage for achieving the desired responses.

**Methodology:**

All randomized controlled trials (RCTs) about SEC for MPE were collected from electronic databases (from inception until July 2021), and clustered into multiple logical topics. *After evaluating their methodological quality, we pooled the data from each topic using the meta-analysis or descriptive analysis, and summarized the evidence quality using the* grading of recommendation assessment, development, and evaluation (GRADE) approach.

**Results:**

All 114 *studies* were clustered into SEC *perfusion* alone or plus chemical agents. The SEC alone showed a better complete response (CR), a lower pleurodesis failure, and adverse drug reactions (ADRs), and a higher fever than cisplatin (DDP) alone. The SEC and chemical agents developed 10 perfusion protocols. *Among them, only SEC and DDP perfusion showed a better CR, a lower failure, disease progression and ADRs, and a higher fever than DDP alone*. The SEC (100–200 ng per time, one time a week for one to four times) with DDP (30–40 mg, or 50–60 mg each time) significantly improved clinical responses for patients with moderate to large volume, Karnofsky performance status (KPS) scores ≥40, ≥50, or ≥60, and anticipated survival time (AST) ≥2 or 3 months. Most results were moderate to low quality.

**Conclusion:**

Current pieces of evidence indicate that super-antigen SEC is a pleurodesis agent, which provides an attractive alternative to existing palliative modalities for patients with MPE. Among 10 protocols, the SEC and DDP perfusion is a most commonly used, which shows a significant improvement in clinical responses with low ADRs. These findings also *provide* a possible indication and optimal usage for SEC and DDP perfusion.

## Introduction

*Malignant pleural effusion (MPE) is a common manifestation of malignant tumors and a significant source of cancer morbidity and mortality, which often causes progressive breathlessness, short survival, and poor quality, and requires palliation* (1, 2). So far, the pleurodesis has remained the cornerstone of treatment, and the pleurodesis agents include chemical agents (3–5), biologic response modifiers (6, 7), and traditional Chinese medicine injections (TCMIs) (8, 9), etc. *As important biologic response modifiers*, serial bio-products from *Staphylococcus aureus* (*S. aureus*) (10), hemolytic streptococcialpha (11, 12), corynobactum parvum (*C. parvum*) (13), and streptococcus pyogenes (*S. pyogenes*) (14) have been used in clinical studies to achieve pleurodesis and control fluid recurrence. Most strikingly, the *S. aureus* toxins, *super-antigens*, stimulate *a polyclonal T-cell response*, and result in massive cytokine production as interleukin 2 (IL-2), tumor necrosis factor α (TNF α), and interferon gamma (IFN γ), which cause pleural inflammation and fibrosis, culminating in pleurodesis (7, 15, 16). In China, the staphylococcal enterotoxin C (SEC) injection (highly agglutinative staphylococcin), a commercially available bio-product from *S. aureus* (including enterotoxin C, other proteins, and 18 amino acids) *had been* approved for adjuvant radiotherapy and chemotherapy in patients with malignant tumors (17, 18). *Since the 1990s, SEC alone or in combination with other pleurodesis agents has been widely used to control MPE* through intrapleural perfusion (10, 19, 20). According to the Cochrane systematic evaluation, two meta-analyses (21, 22) reported that the SEC in combination with chemotherapeutic drugs or cisplatin (DDP) might improve the clinical efficacy with good safety in pleural effusion and ascites. *Previous meta-analyses* (21, 22) *only* determined the clinical effectiveness and safety of SEC pluschemotherapeutic drugs or cisplatin (DDP) for MPE. *Obviously, they could not systematically determine whether perfusion with SEC alone is better or equal to other agents. If used with other agents, which perfusion protocols can achieve ideal clinical effectiveness remain unclear*. Additionally, no evidence determines *their* indications and optimal dose, treatment frequency, and times. These questions *became* the main sources for irrational drug use and clinical decision-making failure. Therefore, we further designed and performed a new systematic review (SR) and meta-analysis to (i) clarify the intrapleural perfusion protocols with SEC, (ii) determine their clinical effectiveness and safety, (iii) reveal their indications and optimum usage, and (iv) provide an evidence framework for formulating *scientific and reasonable* control strategies in MPE.

## Methods

*To clarify the perfusion protocols with SEC and determine their clinical effectiveness and safety, it is obvious that this study had clinical heterogeneity. So, we classified the heterogeneity as significant and potential clinical heterogeneity. On the basis of the principle of evidence classification (**23**) and our previous experiences (**6*, *9**), we systematically collected and evaluated all available randomized controlled trials (RCTs), implemented topic clustering to obtain serial homogeneous perfusion protocols, and analyzed the data from each protocol using the meta-analysis or descriptive analysis. Then, we implemented a subgroup analysis to deal with the potential heterogeneity for main protocol. Finally, this study provided an evidence framework for developing a treatment strategy in MPE. This new evaluation was defined as a clustered SR and meta-analysis*. During implementation, any disagreements were settled by discussion between two independent reviewers, or with a third party (Zheng Xiao). We designed, performed, and reported *this analysis*, following the preferred reporting items for systematic reviews and meta-analyses (PRISMA) guidelines (**PRISMA 2020 Checklist**) (24).

### Retrieval and Screening Strategy

We developed the retrieval strategy using MeSH and free words. The retrieval form was [“Pleural Effusion” (Mesh) OR Pleural Effusions OR hydrothorax OR Pleural Effusion OR Carcinomatous pleurisy OR Cancerous pleurisy OR Malignant pleurisy OR MPE OR MPEs] AND [“Enterotoxin C, staphylococcal” (Supplementary Concept) OR Staph enterotoxin C OR Staphjlo Toxoid Injection OR Staphylococcal Enterotoxin C Injection OR Staph enterotoxin C2 OR SEC2 toxin OR toxin SEC2 OR Staph enterotoxin C3 OR Staph enterotoxin C1 OR SEC1 toxin OR Highly agglutinative staphylococcin OR Gao, jusheng OR Gao jusheng OR Jinpusu]. Hong Jiang and Cheng-Qiong Wang independently searched all published studies about “SEC for MPE” from the electronic databases (from inception until May 2021), such as PubMed, Embase, Web of Science, China National Knowledge Infrastructure Database (CNKI), Chinese Scientific Journals Full-Text Database (VIP), Wanfang Database, China Biological Medicine Database (CBM), and Cochrane Central Register of Controlled Trials (CENTRAL, Issue 7 of 12, July 2021). All ongoing trials were searched from Chinese clinical trial registry (Chi-CTR, http://www.chictr.org.cn), WHO International Clinical Trials Registry Platform (WHO-ICTRP, http://apps.who.int/trialsearch/), and US-clinical trials (https://clinicaltrials.gov/, up to July 2021). Additionally, all SRs/meta-analyses about “SEC for MPE” were evaluated, and all eligible *studies* from their references were also included. Hong Jiang and Xue-Mei Yang independently collected eligible *studies* using the pre-designed inclusion and exclusion criteria.

### Inclusion and Exclusion Criteria

All eligible studies must meet *the* following criteria. According to the design characteristics of intervention study, all trials were randomized controlled trials (RCTs), which reported at least a “*random allocation.”* All patients *had symptomatic pleural effusion resulting from an underlying malignant process* (*of any type and stage*), *which was diagnosed* by using a chest imaging, pleural effusion analysis, cytology, or pleural biopsy. *The drainage method of pleural fluid was not limited*. One month before perfusion, *all patients* did not receive intrapleural perfusion with any agents. The intervention studied was SEC (National Medical Products Administration in China, GYZZ.S19990010 or S10970071, 10 ng or 250 IU/ml). The experimental groups received the perfusion with SEC alone or *plus* chemical agents, and the control groups received the pleurodesis agents alone. The primary indicators were clinical responses and survivals, and the secondary were quality of life (QOL) and adverse events. No restriction was set on the research site and follow-up protocols.

The exclusion criteria were as follows: studies about patients *receiving both SEC perfusion and* systemic chemotherapy; studies about SEC *in combinations with* other biologic response modifiers, traditional Chinese medicine injections (TCMIs) or hyperthermia; studies about both groups *receiving SEC perfusion;* and studies without data of primary or secondary indicators.

### Indexes Definition

The clinical responses were measured by using a complete response (CR), pleurodesis failure, and disease progression (DP). Integrating previous criteria (6, 9, 25–27), the CR is defined as a *pleural fluid* disappeared for more than *1* month, or the lack of accumulation of fluid; the partial response (PR) is a *pleural fluid* reduced more than 50% for more than 1 month; the no response (NR)/stable disease (SD) is *pleural fluid* reduced <50% or increased <25% or the *pleural fluid* recurred but required no further therapy; and the DP is *pleural fluid* increased more than 25% along with other signs of progression or symptomatic re-accumulation of the effusion, requiring repeat thoracentesis or chest tube. Accordingly, the pleurodesis failure was defined as NR or SD plus DP. The survivals were measured by using an overall survival (OS) rate, progression-free survival (PFS) rate, or hazard ratio (HR) of the OS and PFS.

*If the scores increased ten points or higher after perfusion, the QOL was considered as an improvement according to the Karnofsky Performance Status (KPS) Scale* (28, 29). The adverse events were measured by using adverse drug reactions (ADRs), SEC-related adverse events, and treatment-related adverse events (TRAEs). According to the World Health Organization (WHO) (30) or Common Terminology Criteria for Adverse Events (CTCAE) standards (31), the ADRs were defined as myelosuppression, neutropenia, thrombocytopenia, anemia, gastrointestinal reactions, hepatorenal dysfunction, and cardiac dysfunction. The SEC-related adverse events were defined as the drug allergy, fever, and others. The TRAEs were defined as treatment-related mortality and thoracentesis-related events, which included the thoracodynia, fever, respiratory failure, pneumothorax, cutaneous emphysema, and catheter-related infection/chest infection, among others.

### Data Extraction

Jiao Xu and Jun Huang independently extracted data using a pre-designed data extraction form. If without Kaplan–Meier survival curves or other relevant data, we contacted the authors to obtain available survival data. When unavailable, we reconstructed the Kaplan–Meier survival curves into available data using Engauge Digitizer 4.1 (32). The data included the time of publication, the primary tumors, the volume of *pleural fluid*, and the KPS score, anticipated survival time (AST) and treatment history, the cases of the experimental and control group, the demographic and methodological characteristics, the drainage method, the usages of SEC and pleurodesis agents, the follow-up, the evaluation criteria, and the primary or secondary indicators.

### Evaluation of Methodological Bias

Hong Jiang and Xue-Mei Yang independently evaluated the methodological bias using the Cochrane Handbook for Systematic Reviews of Interventions Version 6.2.0 (33, 34). The risk indexes were the generating methods of random sequence, the allocation concealment, the blind methods, the incomplete outcome data, the selective reporting, and other bias (e.g., whether the baseline was comparable). The risk of each index was rated as “Yes” for a low bias, “No” for a high risk of bias, or “unclear.”

### Statistical Analysis

The primary and secondary indexes were described as odds ratios (OR) or HR and their 95% confidence intervals (CI), and the *p* < 0.05 was considered statistically significant. We clustered the eligible *trials* into *serial homogeneous topics* as SEC alone or SEC plus chemical agents, and further analyzed their effectiveness and safety. *After resolving significant clinical heterogeneity, we obtained several homogeneous perfusion protocols*. For different protocols, the statistical heterogeneity was measured by using a Cochran's χ^2−^test and *I*^2^ statistic. If without statistical heterogeneity (*p* ≥ 0.1 and *I*^2^ ≤ 50%), a fixed-effects model (FEM) was performed to pool the data. If *p* < 0.1, *I*^2^ > 50%, and the results had good uniformity, a random-effects model (REM) was performed. Otherwise, the pool was abandoned, and a forest graph was adopted to describe the results. Following previous guidance (35) and our experiences (6, 9), a subgroup analysis model was developed to reveal the *potential* heterogeneity between different trials and determine the effects of variables on clinical responses. The variables were patient baselines, usages of SEC or chemical agents, an evaluation criterion, and published time. A univariable random effects meta-regression was performed to reveal the relationship between each variable and clinical response, and a *post-hoc* multiple regression analysis was performed to adjust their OR. Hong Jiang and Cheng-Qiong Wang independently pooled the data from each protocol using the Review Manager 5.4. If the included *trials* > 10, a funnel plot and Egger's test were used to reveal the risk of bias between trials using the STATA V.15.0 software (401506209499).

The methodological quality and over-estimation to *clinical* effectiveness and security were *core* factors affecting the robustness of results. *So, the implementation process strictly followed the principle of underestimating effectiveness and safety*. We defined the trial as a poor quality when at least one item was considered a high risk. *The trial was defined* as an over or underestimation when the result was significant difference, and beneficial to SEC perfusion. A sensitivity analysis model was developed to evaluate the robustness (6). *Before and after rejecting all the trials with poor quality and over-estimation, if the result had good uniformity*, the outcome was good robustness. Otherwise, the outcome was poor.

### Summary of Evidence Quality

Through integrating the *Grading of Recommendation Assessment, Development and Evaluation (GRADE) approach* (36) and the results of publication bias and sensitivity analysis, a modified model was developed to summarize the evidence quality as a “high,” “moderate,” “low,” or “very low” (6, 9) (Appendix 1). The quality was downgraded in five domains as methodological quality, heterogeneity, indirectness, imprecision, and publication bias. Cheng-Qiong Wang and Xiao-Fan Chen summarized the evidence quality and further generated the absolute estimates of effect using the GRADE profiler.

## Results

### Search Results

**Figure 1:**
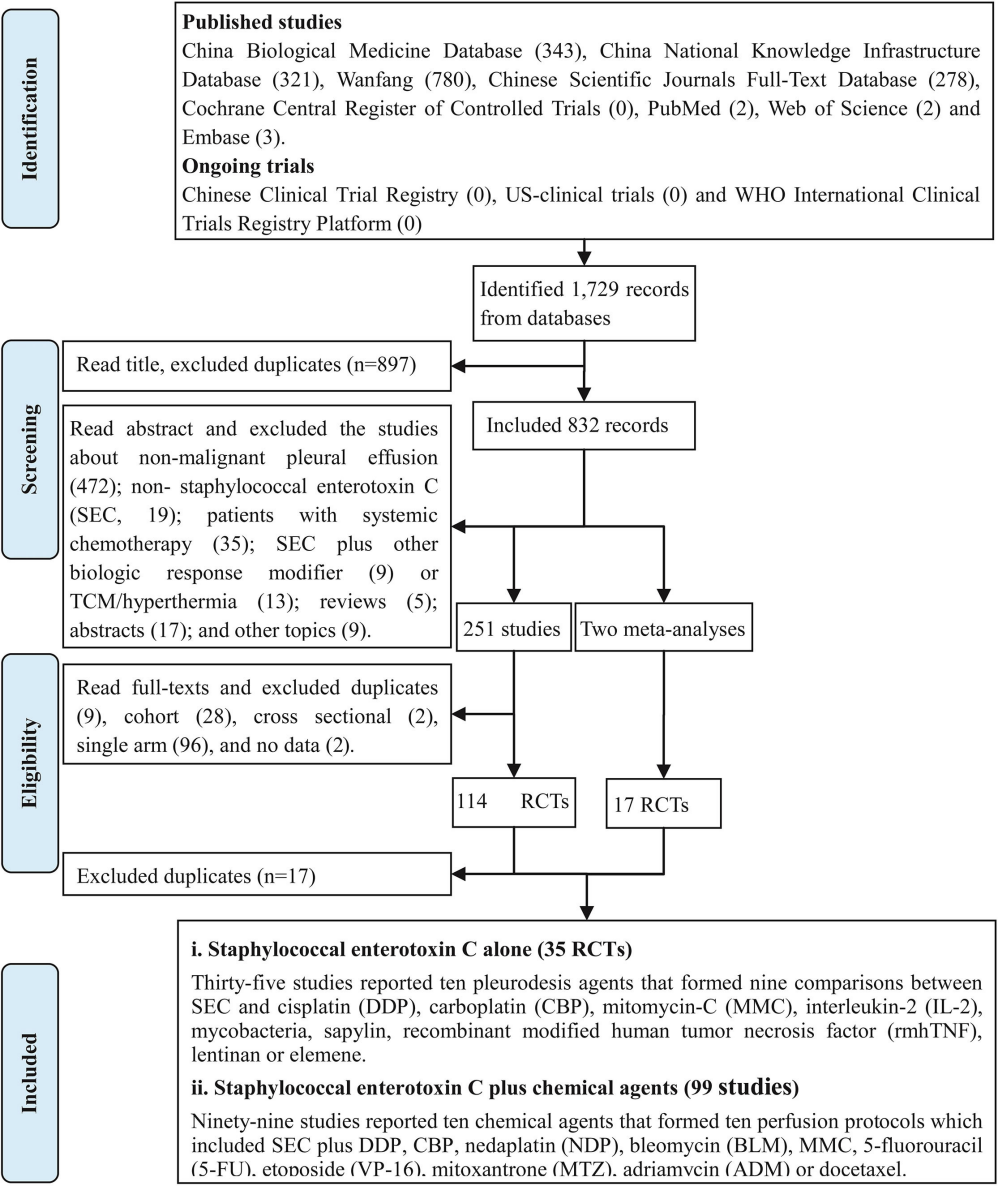
Articles retrieved and assessed for eligibility.

After implementing retrieval strategies, we identified 1,729 records and no ongoing trials. After removing the duplicates, we included 833 records. After reading abstracts and removing irrelevant studies, we collected 250 full texts. After evaluating full texts and removing the ineligible, we collected 114 *studies* (19, 20, 37–148) and two meta-analyses (21, 22). After evaluating the meta-analyses, we collected 17 *studies* (19, 20, 48–50, 56, 59, 63, 69, 73, 77, 89, 109, 121, 133, 141, 144) from their references. Finally, we collected 114 *studies*, which were clustered into SEC alone with 35 *trials* (38, 40, 41, 46–48, 51, 52, 55, 56, 58, 60, 62, 64, 68, 73–75, 77, 79, 83, 86–88, 115, 118, 122, 125, 130, 134, 139, 140, 143, 146, 147) and SEC plus chemical agents with 99 *trials* (19, 20, 37–54, 56, 57, 59, 61–67, 69–73, 75–85, 87, 89–114, 116–121, 123, 124, 126–129, 131–133, 135–139, 141–145, 148). The retrieval results, screening process, and important exclusions are listed in Figure 1 and Appendix 2.

### Characteristics of Included Trials

*In all, we included 114 studies, which were* clustered into intrapleural perfusion with SEC alone and SEC-plus chemical agents. About SEC perfusion alone, the 35 *trials* reported 10 pleurodesis agents, *which* formed nine comparisons between SEC and DDP (38, 40, 41, 46–48, 51, 52, 56, 62, 64, 68, 73–75, 77, 79, 83, 86, 87, 115, 118, 125, 130, 134, 139, 140, 143, 147), carboplatin (CBP), mitomycin-C (MMC), interleukin-2 (IL-2), mycobacteria, sapylin, recombinant modified human tumor necrosis factor (rmhTNF), elemene or lentinan (Table 1). Among them, 29 trials with 1,547 patients evaluated the comparisons of clinical effectiveness and safety between SEC and DDP. Patient ages were ranged from 20 to 86 years, and 606 and 344 cases were male and female, respectively. The experimental groups with 776 cases were administered with SEC through intrapleural perfusion, and the controls with 771 cases were administered with DDP alone. The SEC was used with 80 ng (8 ml, 2,000 IU) to 400 ng (40 ml, 10,000 IU) per time, one time or two times a week, *and lasting one* to eight times. The DDP was 40 to 100 mg per time. Only one to five trials reported other comparisons.

**Table 1:** Characteristics of included studies.

**References**	**Malignant pleural effusions**								**Interventions**			**ET**	**Criteria A, B**	**O**
	**Tumor**	**Volume**	**KPS**	**TH**	**PST**	**E/C**	**M/F**	**Years**	**IPC**	**SEC (Dose, frequency, and times)**	**Pleurodesis agents**			
**Staphylococcal enterotoxin C alone**														
**Staphylococcal enterotoxin C (SEC) vs. Cisplatin (DDP)**														
Li (147)	MT	Un	Un	Un	Un	20/20	27/13	32–71	Un	200–400 ng (20–40 ml),1–2 times/w, Un	DDP: 60–100 mg	Un	Ostrowskimj, Un	O1,3
Cao (143)	MT	Moderate to large	≥50	Un	Un	24/18	26/16	44–70	Un	200 ng (20 ml),1–2 time/w, 1–4 times	DDP: 100 mg	4 w	Ostrowskimj, Un	O1-3
Hu and Jiang (140)	LC	Small to large	Un	Un	Un	13/13	19/7	34–71	Tho	100 ng (10 ml),1 time/w, 2 times/w, Un	DDP: 60 mg	Un	Millar, WHO	O1,3
Jia et al. (139)	MT	Un	≥50	Un	Un	15/15	19/11	30–72	Tho	80 ng (8 ml), 2 time/w, 2–4 times	DDP: 40–60 mg	4 w	Ostrowskimj, Un	O1-3
Huang et al. (134)	MT	Moderate to large	Un	Un	Un	20/18	20/18	42–77	No	100–120 ng (10–12 ml), Un, Un	DDP: 60–100 mg	1 m	Ostrowskimj, Un	O1
Gu et al. (130)	MT	Un	Un	Un	Un	43/43	64/22	59 ± 16.4; 57 ± 14.7	IPC	200 ng (20 ml), 1 time/w, 1–2 times	DDP: 60–80 mg	Un	Ostrowskimj, Un	O1,3
Li (125)	MT	Un	≥70	Un	Un	23/21	27/17	Un	IPC	100 ng (10 ml), 1 time/w, 2–4 times	DDP: 40 mg	Un	Ostrowskimj, Un	O1,3
Fang (118)	MT	Moderate to large	≥70	Un	>3	15/15	18/12	24–72	IPC	120 ng (12 ml), 1 time/w, 2–4 times	DDP: 80 mg	2 w	Ostrowskimj, Un	O1,3
Liu et al. (115)	MT	Un	Un	Un	Un	18/17	21/14	38–69	IPC	24 ng (2.4 ml), 1 time/w, 3 times	DDP: 60 mg	Un	Ostrowskimj, Un	O1,3
Sun and Wang (87)	MT	Un	Un	Un	Un	22/24	31/15	38–83	IPC	200 ng (20 ml),1 time/w, 2–3 times	DDP: 40 mg	1 m	Ostrowskimj, WHO	O1-3
Xue et al. (86)	LC	Moderate to large	Un	Un	Un	33/32	54/11	26–78	IPC	48–80 ng (4.8–8 ml), Un, Un	DDP: 40–60 mg	4 w	Millar, WHO	O1-3
Zhou et al. (83)	MT	Un	≥60	Un	Un	14/12	Un	20–76	IPC	240 ng (24 ml), 2 times/w, 8 times	DDP: 80 mg	4 w	Ostrowskimj, Un	O1,3
Zhang et al. (79)	MT	Moderate to large	≥50	Un	>3	35/34	Un	35–72	Tho	120 ng (12 ml), 1–2 times/w, 1–4 times	DDP: 60–80 mg	4 w	Ostrowskimj, Un	O1-3
Wang et al. (77)	LC	Moderate to large	≥50	Un	≥3	28/28	Un	40–86	Tho	128–160 ng (12.8–16 ml), 2 times/w, 3–4 times	DDP: 80–100 mg	2–3 w	Ostrowskimj, Un	O1,3
Zhang et al. (75)	MT	Un	≥50	Un	>3	37/35	Un	33–78	Tho	120 ng (12 ml), 1–2 times/w, 2–4 times	DDP: 60–80 mg	4 w	Millar, Un	O1-3
Chen et al. (74)	LC	Moderate to large	≥50	Un	Un	32/32	38/26	35–70	IPC	200 ng (20 ml),2 times/w, 4 times	DDP: 40 mg	Un	Ostrowskimj, Un	O1,3
Cheng et al. (73)	LC	Un	≥60	Un	≥3	30/30	33/27	32–76	IPC	200 ng (20 ml), 1 time/w, 3 times	DDP: 40 mg	3 w	Ostrowskimj, Un	O1-3
Wu et al. (68)	MT	Moderate to large	Un	Un	>3	34/30	39/25	68 ± 8; 67 ± 9	IPC	100 ng l (10 ml), 2 times/w, 3 times	DDP: 40 mg	4 w	Ostrowskimj, WHO	O1-3
Xing et al. (64)	LC	Un	>50	Un	Un	16/19	Un	69 ± 5	Un	200 ng (20 ml), 1 time/w, 4 times	DDP: 50 mg	4 w	Ostrowskimj, Un	O1,3
Xu (62)	LC	Un	>50	Un	≥3	26/27	Un	37–78	Tho	128–160 ng (12.8–16 ml), 2 times/w, 4 times	DDP: 80–100 mg	Un	Ostrowskimj, Un	O1,3
Li (56)	LC	Un	≥50	Un	>3	30/30	Un	35–81	Tho	128–160 ng (12.8–16 ml), 1–2 times/w, 4 times	DDP: 80–100 mg	4 w	Ostrowskimj, Un	O1,3
Chen (52)	MT	Moderate to large	Un	Un	Un	30/30	Un	32–86	Tho	Un, Un, Un	DDP: un	Un	Ostrowskimj, Un	O1,3
Tu et al. (51)	LC	Un	>50	Un	Un	32/38	Un	69.4 ± 3.8	Un	200 ng (20 ml), 1 time/w, 4 times	DDP: 50 mg	4 w	Ostrowskimj, Un	O1,3
Zhao (48)	LC	Un	>50	Un	>3	40/40	Un	45–83	Un	200 ng (20 ml), 1 time/w, 4 times	DDP: 50 mg	Un	Ostrowskimj	O1
Cai (47)	LC	Un	Un	Un	Un	21/21	Un	68.5 ± 5.5	Un	200 ng (20 ml), 1 time/w, 4 times	DDP: 50 mg	4 w	Ostrowskimj, Un	O1,3
Liu et al. (46)	LC	Moderate to large	>60	RT	>6	25/29	Un	45–82	IPC	240 ng (24 ml), 1 time/w, 4 times	DDP: 40 mg	4 w	Millar, WHO	O1-3
Yu and Sheng (41)	MT	Un	63	Un	>3	20/20	29/11	59–77	Un	200 ng (20 ml), 1 time/3w, 4 time	DDP: 50 mg	12 w	Ostrowskimj, WHO	O1,3
Luo et al. (40)	LC	Un	≥60	Un	>3	30/30	37/23	40–67	Un	200 ng (20 ml), 1 time/3w, 4 times	DDP: 50 mg	12 w	Ostrowskimj, Un	O1,3
Liu (38)	MT	Un	Un	Un	>3	50/50	57/43	60–76	Un	200 ng (20 ml), 1 time/4w, 4 times	DDP: 50mg	3 m	Ostrowskimj, Un	O1
**Staphylococcal enterotoxin C vs. Carboplatin (CBP)**														
Wang et al. (122)	MT	Un	≥60	Un	>3	40/35	62/13	60–75	IPC	240 ng (24 ml), Un, Un	CBP: 400 mg	3 m	Ostrowskimj, Un	O1-3
**Staphylococcal enterotoxin C vs. Mitomycin-C (MMC)**														
Gao et al. (146)	MT	Small to large	Un	Un	Un	20/20	22/18	34–76	IPC	200–400 ng (20–40 ml), 1–2 times/w, 2–3 times	MMC: 6–8 mg	Un	Ostrowskimj, Un	O1,3
**Staphylococcal enterotoxin C vs. cisplatin (DDP) and mitomycin-C (MMC)**														
Shi (55)	MT	Un	Un	Un	Un	60/56	76/40	32–77	IPC	120–160 ng (12–16 ml), 1 time/w, 1–2 times	MMC: 2 mg; DDP: 20 mg	Un	Millar, Un	O1-3
**Staphylococcal enterotoxin C vs. interleukin-2**														
Tu et al. (51)	LC	Un	>50	Un	Un	32/24	Un	69.4 ± 3.8	Un	200 ng (20 ml), 1 time/w, 4 times	IL-2: 200 IU	4 w	Ostrowskimj, Un	O1,3
Zhao (48)	LC	Un	>50	Un	>3	40/40	Un	45–83	Un	200 ng (20 ml), 1 time/w, 4 times	IL-2: 200 IU	Un	Ostrowskimj	O1
Cai (47)	LC	Un	Un	Un	Un	21/21	Un	68.5 ± 5.5	Un	200 ng (20 ml), 1 time/w, 4 times	IL-2: 200 IU	4 w	Ostrowskimj, Un	O1,3
Yu and Sheng (41)	MT	Un	63	Un	>3	20/20	30/10	57–77	Un	200 ng (20 ml), 1 time/3w, 4 times	IL-2: 200 IU	12 w	Ostrowskimj, WHO	O1,3
Liu (38)	MT	Un	Un	Un	>3	50/50	57/43	60–76	Un	200 ng (20 ml), 1 time/4w, 4 times	IL-2: 200 IU	3 m	Ostrowskimj, Un	O1
**Staphylococcal enterotoxin C vs. mycobacteria**														
Kuang et al. (88)	LC	Un	Un	PT	>3	25/27	30/22	29–85	IPC	160 ng (16 ml), 1–2 times/w, 1–2 times	Mycobacteria: 225 μg	Un	Millar, Un	O1,3
**Staphylococcal enterotoxin C vs. elemene**														
Zhou et al. (60)	MT	Moderate to large	>50	PT	>3	25/27	27/25	26–82	IPC	400 ng (40 ml), Un, Un	Elemene: 400 mg	Un	Ostrowskimj, Un	O1,3
**Staphylococcal enterotoxin C vs. lentinan**														
Gao et al. (58)	LC	Moderate to large	Un	Un	>3	20/20	23/17	55–84	IPC	200 ng (20 ml), 2 times/w, 2 times	Lentinan: 5 mg	4 w	Ostrowskimj, Un	O1,3
**Staphylococcal enterotoxin C vs. sapylin**														
Chen (52)	MT	Moderate to large	Un	Un	Un	30/30	Un	32–86	Tho	Un, Un, Un	Sapylin: un	Un	Ostrowskimj, Un	O1,3
**Staphylococcal enterotoxin C vs. recombinant modified human tumor necrosis factor (rmhTNF)**														
Liu et al. (46)	LC	Moderate to large	>60	PT	>6	25/31	Un	45–82	IPC	240 ng (24 ml), 1 time/w, 4 times	rmhTNF: 15 × 106 U	4 w	Millar, WHO	O1-3
**Staphylococcal enterotoxin C and chemical agents**														
**Staphylococcal enterotoxin C plus cisplatin (DDP)**														
Li et al. (20)	LC	Small to large	≥40	Un	Un	20/20	30/10	36–68	Un	160 ng (16 ml), 1–2 times/w, 4–6 times	DDP: 40–60 mg	Un	Ostrowskimj, Un	O1-3
Li and Yang (148)	MT	Small to large	Un	Un	Un	68/42	59/51	35–72	Tho	160–240 ng (16–24 ml), 1 time/1 to 2 w, 6 times	DDP: 40–60 mg	8 w	Millar, Un	O1,3
Qiu et al. (19)	MT	Moderate to large	≥40	Un	Un	42/42	59/25	28–72	IPC	320–400 ng (32–40 ml), 1 time/w, 2–3 times	DDP: 80–100 mg	Un	Ostrowskimj, Un	O1-3
Zhang et al. (145)	MT	Moderate to large	≥50	Un	Un	15/15	24/6	35–79	IPC	160 ng (16 ml), 1 time/w, 1–2 times	DDP: 60–80 mg	4 w	Ostrowskimj, Un	O1,3
Cao (143)	MT	Moderate to large	≥50	Un	Un	26/18	25/19	44–70	Un	200 ng (20 ml), 1–2 times/w, 1–4 times	DDP: 100 mg	4 w	Ostrowskimj, Un	O1-3
Jia et al. (139)	MT	Un	≥50	Un	Un	15/15	19/11	30–75	Tho	80 ng (8 ml), 2 times/w, 2–4 times	DDP: 40–60 mg	4 w	Ostrowskimj, Un	O1-3
Xu and Meng (137)	MT	Un	Un	Un	Un	34/30	40/24	35–68	Un	160 ng (16 ml), 1–2 times/w, 1–4 times	DDP: 60 mg	Un	Ostrowskimj, Un	O1,3
Zhang et al. (136)	MT	Un	Un	Un	Un	36/30	41/25	20–75	Tho	240 ng (24 ml), 1 time/w, Un	DDP: 50 mg	Un	Ostrowskimj, Un	O1,3
Lang et al. (133)	MT	Un	>40	Un	>3	56/21	49/28	31–69	Tho	200 ng (20 ml), 1–2 times/w, 2–4 times	DDP: 40–60 mg	1 m	Millar, Un	O1-3
Wang (129)	MT	Un	Un	Un	Un	29/29	38/20	21–71	Un	100 ng (10 ml), 1 time/w, 4 times	DDP: 60 mg	4 w	Millar, Un	O1-3
Wang et al. (128)	MT	Un	>40	Un	>3	21/19	Un	Un	Tho	200 ng (20 ml), 1 time/w, 2–3 times	DDP: 80 mg	Un	Ostrowskimj, Un	O1,3
Duan et al. (127)	MT	Un	≥60	Un	Un	76/76	82/70	39–70	IPC	120ng (12ml), 1time/w,2-3 times	DDP:80-100mg	Un	Ostrowskimj, Un	O1,3
Li et al. (126)	MT	Un	Un	Un	Un	23/21	30/14	35–76	Tho	200 ng (20 ml), 1–2 times/w, 1–4 times	DDP: 60–80 mg	Un	Millar, Un	O1,3
Wang and Zhou (123)	MT	Un	Un	Un	Un	15/15	19/11	30–75	Tho	160 ng (16 ml), 1–2 times/w, 1–3 times	DDP: 60 mg	Un	Ostrowskimj, Un	O1,3
Xu et al. (121)	MT	Un	Un	Un	Un	32/30	42/11	58.2 ± 3.1; 57.8 ± 2.7	IPC	200 ng (20 ml), 1 time/w, 4 times, DDP (40 mg)	DDP: 80 mg	1 m	Ostrowskimj, Un	O1-3
Zhang et al. (120)	MT	Un	>50	Un	Un	25/28	30/23	37–69	IPC	200 ng (20 ml), 1 time/w, 2–3 times, DDP (40 mg)	DDP: 60 mg	1 m	Ostrowskimj, Un	O1-3
Chen et al. (119)	MT	Moderate to large	Un	Un	Un	24/24	28/20	35–72	IPC	120 ng (12 ml), 2 times/w, 1–2 times	DDP: 60 mg	4 w	Ostrowskimj, Un	O1,3
Fang (118)	MT	Moderate to large	≥70	Un	>3	15/15	17/13	24–73	IPC	120 ng (12 ml), 1 time/w, 2–4 times, DDP (60 mg)	DDP: 80 mg	2 w	Ostrowskimj, Un	O1,3
Guan et al. (117)	MT	Moderate to large	≥70	Un	>3	23/22	Un	20–71	IPC	200 ng (20 ml), 1 time/w, 1–3 times	DDP: 80 mg	1 m	Ostrowskimj, WHO	O1,3
Hu et al. (116)	MT	Moderate to large	>60	Un	>3	23/22	26/19	32–76	Tho	200 ng (20 ml), 1 time/w, 1–4 times	DDP: 60 mg	2 w	Ostrowskimj, Un	O1-3
Mao (114)	MT	Un	Un	Un	Un	24/16	26/14	36–72	Un	100 ng (10 ml), 1–2 times/w, 1–4 times	DDP: 60 mg	2 w	Ostrowskimj, Un	O1,3
Wang (112)	MT	Moderate to large	>50	PT	Un	23/22	29/16	41–83	Tho	80 ng (8 ml), 1 time/w, 3 times	DDP: 80 mg	1 m	Millar, WHO	O1-3
Zhu et al. (110)	MT	Un	Un	Un	Un	34/30	47/17	62 ± 12; 58 ± 14	IPC	240 ng (24 ml), 1 time/w, 3 times	DDP: 40 mg	Un	Ostrowskimj, Un	O1-4
Chen and Cheng (109)	MT	Un	>60	Un	Un	27/24	30/21	26–72	IPC	120–200 ng (12–20 ml), 1–2 times/w, Un	DDP: 30 mg	4–8 w	Ostrowskimj, Un	O1,3
Feng et al. (108)	MT	Un	>60	Un	>5	17/17	20/14	50–82	IPC	200 ng (20 ml), 1 time/w, 1–4 times	DDP: 60 mg	8 w	Ostrowskimj, Un	O1-3
Huang et al. (107)	LC	Un	Un	Un	Un	27/21	Un	30–73	Tho	80 ng (8 ml), 1 time/w, 1–4 times, DDP (60 mg)	DDP: 60–80 mg	Un	Ostrowskimj, Un	O1,3
Liu et al. (105)	LC	Moderate to large	≥60	Un	>2	50/48	77/21	38–78	IPC	200 ng (20 ml), 1 time/w, 3 times	DDP: 40–60 mg	Un	Millar, WHO	O1,3
Liu et al. (104)	MT	Small to large	≥50	Un	>2	32/29	28/33	34–76	IPC	80–160 ng (8–16 ml), 2 times/w, 2–5 times	DDP: 60–80 mg	4 w	Ostrowskimj, Un	O1,3
Ma et al. (102)	MT	Un	>50	Un	Un	24/24	32/16	32–83	IPC	120 ng (12 ml), 2 times/w, 2–3 times	DDP: 60 mg	2–3 w	Ostrowskimj, WHO	O1,3
Zhang and Hu (101)	LC	Un	Un	Un	Un	23/21	33/11	Un	IPC	80 ng (8 ml), 1 time/w, 1–3 times	DDP: 60 mg	Un	Ostrowskimj, Un	O1-3
Chen et al. (100)	LC	Un	Un	Un	Un	12/11	15/8	Un	IPC	160 ng (16 ml), 1 time/w, 4 times	DDP: 80 mg	Un	Millar, Un	O1,3
Fang et al. (98)	LC	Moderate to large	≥50	Un	≥3	24/22	28/18	33–72	IPC	200 ng (20 ml), 1 time/w, 3 times	DDP: 60 mg	3 w	Ostrowskimj, Un	O1-3
Pan et al. (95)	MT	Moderate to large	Un	Un	Un	28/20	31/17	45–72	IPC	80 ng (8 ml), 2 times/w, 1–2 times	DDP: 100 mg	4 w	Ostrowskimj, Un	O1,3
Wang et al. (93)	MT	Moderate to large	>50	Un	Un	30/30	38/22	39–82	IPC	80–160 ng (8–16 ml), 1 time/w, 2–3 times	DDP: 60–80 mg	4 w	Millar, WHO	O1-3
Xiong and Liu (92)	MT	Large	≥40	Un	Un	22/22	26/18	26–72	IPC	100–120 ng (10–12 ml), 1 time/w, 3 times	DDP: 60 mg	Un	Ostrowskimj, Un	O1,3
Yue and Bai (91)	MT	Un	≥60	Un	>3	33/28	29/32	37–72	Tho	100 ng (10 ml), 1 time/w, 2 times	DDP: 60 mg	4 w	Ostrowskimj, WHO	O1-3
Zhang (90)	MT	Un	≥40	Un	Un	16/16	Un	Un	IPC	100–200 ng (10–20 ml), 1–2 times/w, 4–12 times	DDP: 60–80 mg	Un	Ostrowskimj, Un	O1-3
Zhao et al. (89)	MT	Moderate to large	Un	Un	Un	32/32	36/28	32–75	IPC	80 ng (8 ml), 1–2 times/w, 2–8 times	DDP: 60 mg	4 w	Ostrowskimj, Un	O1-3
Sun and Wang (87)	MT	Un	Un	Un	Un	24/24	34/14	38–83	IPC	200 ng (20 ml), 1 time/w, 2–3 times	DDP: 40 mg	1 m	Ostrowskimj, WHO	O1-3
Yin and Tao (85)	MT	Un	Un	Un	Un	19/19	20/18	38–90	IPC	160 ng (16 ml), Un, Un	DDP: 60–80 mg	Un	Ostrowskimj, Un	O1-3
Zheng (84)	MT	Un	Un	Un	Un	24/20	33/11	36–69	un	100 ng (10 ml), 1–2 times/w, 1–4 times	DDP: 60 mg	Un	Millar, Un	O1,3
Zhou et al. (83)	MT	Un	≥60	Un	Un	16/12	Un	20–76	IPC	240 ng (24 ml), 2 times/w, 8 times	DDP: 80 mg	4 w	Ostrowskimj, Un	O1,3
Huang an Wang (82)	LC	Un	≥50	Un	≥3	28/28	37/19	41–83	Tho	128–160 ng (12.8–16 ml), 2 times/w, 3–4 times	DDP: 80–100 mg	2–3 w	Ostrowskimj, Un	O1,3
Li et al. (81)	MT	Moderate to large	Un	Un	Un	25/25	34/16	40–82	Tho	80 ng (8 ml), 1 time/w, 3 times	DDP: 40 mg	4 w	Ostrowskimj, WHO	O1-3
Zhang et al. (79)	MT	Moderate to large	≥50	Un	>3	33/34	Un	35–72	Tho	120 ng (12 ml), 1–2 times/w, 1–4 times	DDP: 60–80 mg	4 w	Ostrowskimj, Un	O1-3
Gao et al. (78)	MT	Moderate to large	>60	PT	Un	35/30	36/29	Un	IPC	160 ng (16 ml), Un, Un	DDP: 60 mg	4 w	Ostrowskimj, Un	O1,3
Wang et al. (77)	LC	Moderate to large	≥50	Un	≥3	28/28	Un	40–86	Tho	128–160 ng (12.8–16 ml), 2 times/w, 3–4 times	DDP: 80–100 mg	2–3 w	Ostrowskimj, Un	O1,3
Zhang and Chen (75)	MT	Un	≥50	Un	>3	34/35	Un	33–78	Tho	120 ng (12 ml), 1–2 times/w, 2–4 times	DDP: 60–80 mg	4 w	Millar, Un	O1-3
Cheng et al. (73)	LC	Un	≥60	Un	≥3	30/30	34/26	32–76	IPC	200 ng (20 ml), 1 time/w, 3 times	DDP: 40 mg	3 w	Ostrowskimj, Un	O1-3
Gui (72)	MT	Un	≥50	Un	>2	25/25	30/20	35–70	IPC	200 ng (20 ml), 1 time/3d, 1–3 times	DDP: 60 mg	Un	Millar, WHO	O1-3
Li and Man (71)	LC	Un	>50	Un	>2	230/230	300/160	36–78	IPC	80 ng (8 ml), 2 times/w, 2–3 times	DDP: 50 mg	4 w	Millar, WHO	O1-3
Liu (70)	MT	Un	≥50	Un	>3	27/20	27/20	39–81	IPC	160 ng (16 ml), 1 time/w, 3 times	DDP: 60 mg	2 m	Millar, Un	O1-3
Qu et al. (69)	MT	Un	≥60	Un	≥3	40/40	58/22	29–72	IPC	120 ng (12 ml), 1 time/w, 3 times	DDP: 60 mg	1 m	Ostrowskimj, WHO	O1-3
Zhang and Li (66)	MT	Un	Un	Un	Un	19/19	27/11	Un	IPC	200 ng (20 ml), 2 times/w, 2–6 times	DDP: 80 mg	Un	Ostrowskimj, Un	O1,3
Qin and Zhang (65)	MT	Un	>40	Un	Un	34/34	36/32	28–76	IPC	200 ng (20 ml), 2 times/w, 2–6 times	DDP: 30 mg	Un	Ostrowskimj, Un	O1,3
Xing et al. (64)	LC	Un	>50	Un	Un	19/19	Un	69 ± 5	Un	400 ng (40 ml), 1 time/w, 4 times	DDP: 50 mg	4 w	Ostrowskimj, Un	O1,3
Xu (63)	LC	Un	≥50	Un	≥3	27/27	33/21	38–82	Tho	140–160 ng (14–16 ml), 2 times/w, 3 times	DDP: 70–100 mg	2 w	Ostrowskimj	O1
Xu (62)	LC	Un	>50	Un	≥3	27/27	Un	37–78	Tho	128–160 ng (12.8–16 ml), 2 times/w, 4 times	DDP: 80–100 mg	Un	Ostrowskimj,Un	O1,3
Yu and Xiao (61)	LC	Un	Un	Un	Un	25/25	28/22	47–76	IPC	160–200 ng (16–20 ml), 1 time/w, 3 times	DDP: 60–90 mg	1 m	Ostrowskimj	O1
Du (59)	LC	Un	Un	Un	>3	60/60	75/45	37–81	Tho	140–160 ng (14–16 ml), 2 times/w, 3 times	DDP: 70–100 mg	Un	Ostrowskimj, Un	O1,3
Li and Qian (57)	MT	Un	≥70	Un	Un	15/15	19/11	58	IPC	80 ng (8 ml), 1 time/w, 2 times	DDP: 60 mg	4 w	Ostrowskimj, Un	O1-3
Li (56)	LC	Un	≥50	Un	>3	30/30	Un	35–81	Tho	128–160 ng (12.8–16 ml), 1–2 times/w, 4 times, DDP (80 mg)	DDP: 80–100 mg	4 w	Ostrowskimj, Un	O1,3
Yu and Wang (53)	LC	Un	>60	Un	>3	23/22	24/21	35–73	IPC	80 ng (8 ml), Un, Un	DDP: 40 mg	4 w	Ostrowskimj, Un	O1-3
Chen (52)	MT	Moderate to large	Un	Un	Un	30/30	Un	32–86	Tho	Un, Un, Un	DDP: un	Un	Ostrowskimj, Un	O1,3
Tu et al. (51)	LC	Un	>50	Un	Un	38/38	Un	69.4 ± 3.8	Un	200 ng (20 ml), 1 time/w, 4 times	DDP: 50 mg	4 w	Ostrowskimj, Un	O1,3
Yao (50)	MT	Un	Un	Un	>3	25/25	24/26	32–68	IPC	200 ng (20 ml), 1 time/w, 4 times	DDP: 50 mg	Un	Ostrowskimj, CTC3.0	O1,3
Zhang (49)	LC	Un	≥60	Un	Un	40/40	56/24	53–71	Un	200 ng (20 ml), 1 time/5d, 6 times	DDP: 50 mg	Un	Ostrowskimj, Un	O1,3
Zhao (48)	LC	Un	>50	Un	>3	40/40	Un	45–83	Un	200 ng (20 ml), 1 time/w, 4 times	DDP: 50 mg	Un	Ostrowskimj	O1
Cai (47)	LC	Un	Un	Un	Un	21/21	Un	68.5 ± 5.5	Un	200 ng (20 ml), 1 time/w, 4 times	DDP: 50 mg	4 w	Ostrowskimj, Un	O1,3
Liu et al. (46)	LC	Moderate to large	>60	PT	>6	30/29	Un	45–82	IPC	240 ng (24 ml), 1 time/w, 4 times	DDP: 40 mg	4 w	Millar, WHO	O1-3
Zhang et al. (45)	LC	Un	Un	Un	Un	45/45	50/40	36–80	Tho	140–160 ng (14–16 ml), 1 time/4d, 3 times	DDP: 70–100 mg	Un	Ostrowskimj	O1-2
Zhou (44)	LC	Un	Un	Un	Un	21/21	25/17	41–75	IPC	100 ng (10 ml), 1 time/w, 2 times	DDP: 40 mg	4 w	Millar, WHO	O1-3
Li (43)	LC	Un	Un	Un	>3	40/40	47/33	38–83	Tho	140–160 ng (14–16 ml), 2 times/w, 3 times	DDP: 70–100 mg	Un	Ostrowskimj, Un	O1,3
Yan (42)	MT	Un	Un	Un	Un	33/28	38/23	50–74	Un	200 ng (20 ml), 1 time/5d, 6 times	DDP: 50 mg	4 w	Ostrowskimj, Un	O1,3
Yu and Sheng (41)	MT	Un	63	Un	>3	20/20	29/11	59–75	Un	200 ng (20 ml), 1 time/3w, 4 time	DDP: 50 mg	12 w	Ostrowskimj, WHO	O1,3
Luo et al. (40)	LC	Un	≥60	Un	>3	30/30	36/24	40–68	Un	200 ng (20 ml), 1 time/3w, 4 times	DDP: 50 mg	12 w	Ostrowskimj, Un	O1,3
Wang (39)	MT	Un	Un	Un	Un	37/37	42/32	44–72	IPC	80 ng (8 ml), 1 time/3–4d, Un	DDP: 100 mg	4 w	Ostrowskimj, Un	O1,3
Liu (38)	MT	Un	Un	Un	>3	50/50	55/45	60–77	un	200 ng (20 ml), 1 time/4w, 4 times	DDP: 50 mg	16 w	Ostrowskimj, Un	O1
Wu (37)	MT	Un	Un	Un	Un	46/46	53/39	51–74	Un	200 ng (20 ml), 1 time/5d, 6 times	DDP: 50 mg	1 m	Ostrowskimj, Un	O1,3
**Staphylococcal enterotoxin C plus carboplatin (CBP)**														
Xu et al. (111)	MT	Un	≥60	Un	>3	21/15	26/10	35–72	Tho	300 ng (30 ml), 1–2 times/w, 2–4 times	CBP: 300 mg	4 w	Ostrowskimj, Un	O1,3
Liang et al. (106)	MT	Un	≥50	Un	>2	20/18	21/17	50–70	IPC	120 ng (12 ml), 1 time/3w, 1–3 times	CBP: 400 mg	6 w	Ostrowskimj, Un	O1,3
Jiang et al. (96)	MT	Moderate to large	≥50	Un	>3	23/22	25/20	40–78	Tho	160 ng (16 ml), 1–2 times/w, 3–6 times	CBP: 400 mg	3 w	Ostrowskimj, WHO	O1,3
**Staphylococcal enterotoxin C plus nedaplatin (NDP)**														
Xu et al. (67)	MT	Un	≥60	Un	>3	32/26	26/32	39–72	IPC	80 ng (8 ml), 1 time/w, 2 times	NDP: 60 mg	4 W	Ostrowskimj, WHO	O1-3
**Staphylococcal enterotoxin C plus bleomycin (BLM)**														
Chen et al. (142)	MT	Moderate to large	≥40	Un	Un	30/30	32/18	35–72	IPC	240–320 ng (24–32 ml), 1 time/w, 1–2 times	BLM: 45–60 mg	1 m	Ostrowskimj, Un	O1-3
Shen et al. (94)	MT	Small to large	>40	Un	Un	25/24	25/24	28–72	IPC	120 ng (12 ml), 1 time/w, 2 times	BLM: 45 mg	1 m	Millar, WHO	O1-3
Mo (80)	MT	Moderate to large	>50	Un	Un	50/47	58/39	45–83	IPC	120 ng (12 ml), Un, Un	BLM: 1 mg/kg	4 w	Millar, WHO	O1,3
Yuan et al. (76)	MT	Un	>40	Un	Un	60/50	65/45	28–72	IPC	120 ng (12 ml), 1 time/w, 2 times	BLM: 1 mg/kg	Un	Ostrowskimj, WHO	O1,3
**Staphylococcal enterotoxin C plus mitomycin-C (MMC)**														
Zhang (135)	MT	Un	Un	PT	Un	36/33	43/26	33–74	Un	320 ng (32 ml),1 time/w, 4 times	MMC: 6 mg	4 w	Ostrowskimj, Un	O1-3
Tao et al. (113)	MT	Moderate to large	Un	PT	Un	16/16	20/12	39–76	IPC	160 ng (16 ml), Un, Un	MMC: 10 mg	Un	Ostrowskimj, Un	O1-3
Ding et al. (99)	MT	Un	Un	Un	Un	24/21	18/27	72 ± 3; 70 ± 4	IPC	320 ng (32 ml),2 times/w, 2–4 times	MMC: 8 mg	4 w	Ostrowskimj, Un	O1
**Staphylococcal enterotoxin C plus etoposide (VP-16)**														
Tian (132)	MT	Un	≥50	Un	>3	40/38	50/28	30–71	Tho	120 ng (12 ml), 1 time/w, 2 times	VP-16: 300 mg	2 w	Ostrowskimj, Un	O1,3
Liu (103)	MT	Un	>50	Un	>3	29/29	39/18	33–71	Tho	120 ng (12 ml),1 time/w, 2–3 times	VP-16: 300 mg	4 w	Ostrowskimj, WHO	O1,3
**Staphylococcal enterotoxin C plus 5-fluorouracil (5-FU)**														
Sun and Lai (124)	MT	Small to large	Un	Un	Un	31/31	30/32	34–80	IPC	200 ng (20 ml), 1 time/w, 2 times	5-Fu: 0.75–1.0 g	Un	Millar, WHO	O1-3
Huang et al. (97)	MT	Un	Un	Un	Un	30/30	40/20	35–80	IPC	200 ng (20 ml), 2 times/w, 2–5 times	5-Fu: 1.0 g	4 w	Ostrowskimj, Un	O1,3
**Staphylococcal enterotoxin C plus mitoxantrone (MTZ)**														
Zhang (144)	MT	Un	Un	Un	Un	38/20	Un	Un	Un	160 ng (16 ml), 2 times/w, 6 times	MTZ: 10 mg	3 w	Millar, Un	O1
**Staphylococcal enterotoxin C plus adriamycin (ADM)**														
Guan et al. (117)	MT	Moderate to large	≥70	Un	>3	25/23	Un	20–71	IPC	200 ng (20 ml), 1 time/w, 1–3 times	ADM: 30 mg	1 m	Ostrowskimj, WHO	O1,3
**Staphylococcal enterotoxin C plus cisplatin (DDP) and etoposide (VP-16)**														
Zhang (131)	MT	Moderate to large	≥50	Un	Un	20/21	27/14	18–73	Tho	40–80 ng (4–8 ml), 1–2 times/w, 2 times	DDP: 60–80mg; VP16: 0.1 mg	Un	Ostrowskimj, WHO	O1,3
**Staphylococcal enterotoxin C plus docetaxel**														
Xu et al. (54)	LC	Un	≥50	Un	>3	28/28	29/27	42–69	IPC	80 ng (8 ml), 2 times/w, 4 time	Docetaxel: 40 mg	4 w	Ostrowskimj, WHO	O1-3
**Staphylococcal enterotoxin C plus ADM, 5 FU/CBP**														
Fu (141)	MT	Un	Un	Un	Un	28/27	37/18	47.3 ± 9.4; 52.0 ± 9.1	Un	320 ng (32 ml), 1–2 times/w, 4–8 times	ADM, 40 mg; 5 FU: 1 g/CBP: 200 mg	8 w	Millar, WHO	O1-3
**Staphylococcal enterotoxin C plus ADM and DDP**														
Tang (138)	MT	Un	Un	Un	Un	30/30	33/27	32–74	IPC	320 ng (32 ml), 1 time/2w, 1–3 times	ADM: 80 mg; DDP: 80 mg	Un	Ostrowskimj, Un	O1,3

*MT, miscellaneous tumors; NSCLC, non-small cell lung cancer; LC, lung cancer; AST, anticipated survival time; TH, treatment history; PT, primary treatment; MU, million units; IU, international unit; E/C, experimental groups (staphylococcal enterotoxin C)/control groups (pleurodesis agents alone); F/M, female/male; IPC, indwelling pleural catheter; Tho, thoracentesis; ET, evaluation time, Millar: CR, complete response; PR, partial response; SD, stable disease; and DP, disease progression, Ostrowskimj: CR, PR, and NR, no response; WHO, World Health Organization for adverse drug reactions; CTC3.0, common terminology criteria for adverse events 3.0; O, outcomes; O1, clinical responses; O2, quality of life (QOL); O3, adverse events; O4, overall survivals; w, week; m, month; Un, unclear*.

About SEC plus chemical agents, the 99 *trials* (19, 20, 37–54, 56, 57, 59, 61–67, 69–73, 75–85, 87, 89–114, 116–121, 123, 124, 126–129, 131–133, 135–139, 141–145, 148) reported the SEC and 10 *agents, which developed 13 protocols* as SEC plus DDP (19, 20, 37–53, 56, 57, 59, 61–66, 69–73, 75, 77–79, 81–85, 87, 89–93, 95, 98, 100–102, 104, 105, 107–110, 112, 114, 116–121, 123, 126–129, 133, 136, 137, 139, 143, 145, 148), CBP, nedaplatin (NDP), bleomycin (BLM), *5-fluorouracil (5-FU), etoposide (VP-16), mitoxantrone (MTZ), adriamycin (ADM), docetaxel, MMC, or* other agents (Table 1). Seventy-nine *trials* involving 4,924 patients reported the SEC and DDP perfusion. Patient ages were ranged from 20 to 90 years, and 2,523 and 1,547 cases were male and female, respectively. The combination with SEC and DDP perfusion was administered in experimental groups *with* 2,539 patients, and the DDP alone was administered in controls *with* 2,385 patients. The SEC was used with 80 ng (8 ml, 2,000 IU) to 400 ng (40 ml, 10,000 IU) per time, one time or two times a week, *and lasting* one to eight times. The DDP was 30–100 mg per time. Only one to four trials reported other protocols.

On the whole, 82 *studies* involved patients with miscellaneous tumors as lung, breast, and ovarian cancers, among others, and 32 only involved lung cancer (40, 46–48, 51, 56, 62, 64, 73, 74, 77, 86, 140). *Only* some *studies* completely reported the patients' baselines as the volume of pleural fluid, KPS score, AST, and treatment history. Fifty *studies* performed perfusion after draining *pleural fluid* using IPCs. At 2–16 weeks after perfusion, most *studies* evaluated the clinical responses using Ostrowskimj criterion, and QOL using a KPS scale, and only one *study* reported the survivals. One hundred and seven *studies* (19, 20, 37, 39–47, 49–60, 62, 64–98, 100–133, 135–143, 145–148) reported the adverse event. But most trials only reported ADRs using an unclear criterion and ignored the TRAEs and the SEC-related adverse events.

### Risk of Methodological Bias

**Figure 2:**
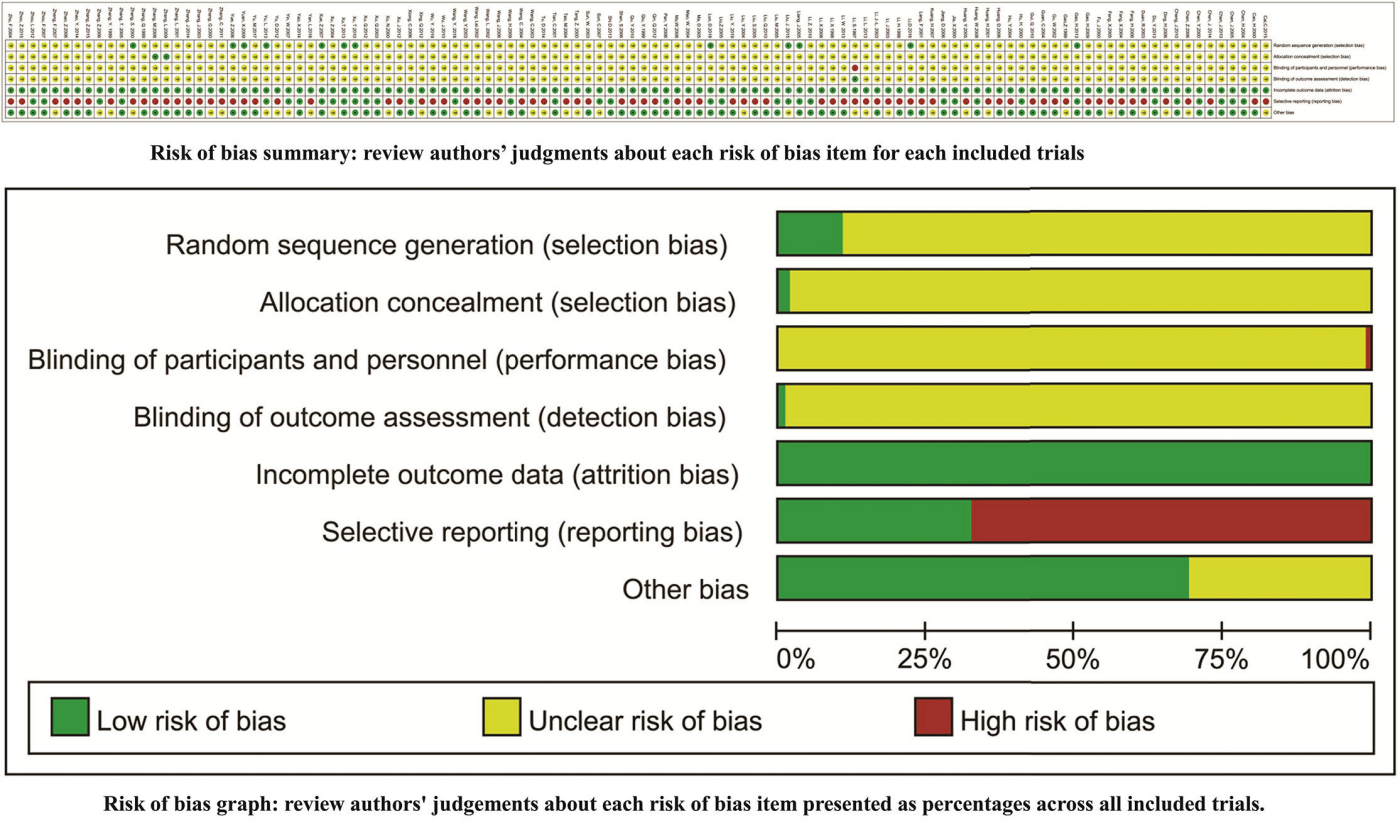
Risk of methodological bias.

*Of 114 studies*, only 11 reported the generating methods of random sequence using a number table (40, 43, 53, 58, 76, 106), coin toss (54, 67, 91), or draw (46, 135). Only three *studies* implemented allocation concealment using an envelope (75, 79, 86). No *studies* provided the detailed information about the blind methods. All the *studies* had complete follow-up. Seventy-seven *studies* had *a* selective reporting for ADRs (19, 20, 37, 38, 41–45, 47–49, 51, 52, 56, 57, 61, 63–66, 68–70, 72, 75, 76, 78–81, 84, 88, 89, 91, 93, 94, 97–100, 102, 104, 107, 108, 110, 112–118, 120, 122–131, 133–138, 141, 143–148). Thirty-five *studies* had an unclear comparability for baselines. The risk of methodological bias is shown in Figure 2.

### Clinical Responses

**Figure 3:**
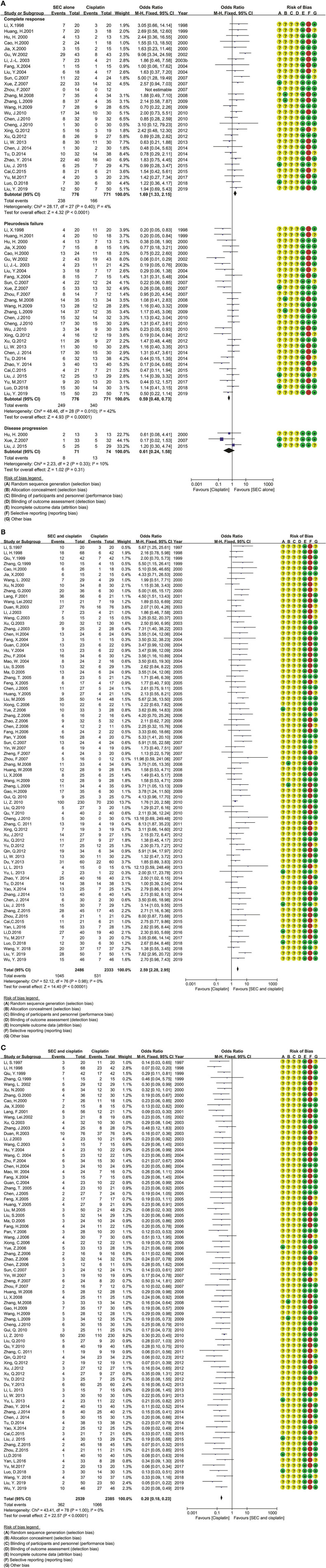
The analysis of clinical responses between the two groups. **(A)** The clinical responses between SEC and DDP alone. SEC, staphylococcal enterotoxin C; DDP, cisplatin; CI, confidence interval. **(B)** The complete response in SEC and cisplatin perfusion. SEC, staphylococcal enterotoxin C; DDP, cisplatin; CI, confidence interval. **(C)** The pleurodesis failure in SEC and cisplatin perfusion. SEC, staphylococcal enterotoxin C; DDP, cisplatin; CI, confidence interval.

In SEC perfusion alone, 35 *trials* reported nine comparisons (Table 2; Figure 3A). The Cochran's χ^2^-test and *I*^2^ statistic only *found* a minimal heterogeneity of CR (*I*^2^ = 4%) and failure (*I*^2^ = 42%) in SEC vs. DDP; we pooled the OR using a FEM. Compared with DDP or IL-2 alone, the results of meta-analyses determined that SEC alone showed a better CR [OR = 1.69, 95% CI (1.33, 2.15), *p* < 0.0001; OR = 1.73, 95% CI (1.03, 2.88), *p* = 0.04] and a lower failure [OR = 0.59, 95% CI (0.48, 0.73), *p* < 0.00001; OR = 0.32, 95% CI (0.19, 0.53), *p* < 0.00001] (Table 2; Figure 3A). In addition, only one trial reported that SEC alone was superior to CBP, and equivalent to MMC, mycobacteria, sapylin, rmhTNF, elemene, or lentinan *alone* (Table 2; Supplementary Figures S1, S2).

**Table 2:** The clinical responses (Figures 3A–C; Appendix 3; Supplementary Figures S1–S5).

**Perfusion protocols**	**Complete response**						**Pleurodesis failure**						**Disease progression**					
	**Trial**	**Cases**	**SM**	**OR (95%CI)**	***I*** **^2^**	***p***	**Trial**	**Cases**	**SM**	**OR (95%CI)**	***I*** **^2^**	***p***	**Trial**	**Cases**	**SM**	**OR (95%CI)**	***I*** **^2^**	***p***
**Staphylococcal enterotoxin C (SEC) alone (****Figure 3A****;** **Appendix 3****;** **Supplementary Figures S1**, **S2**, **S5****)**																		
SEC vs. cisplatin (DDP)	29	1,547	FEM	1.69 (1.33, 2.15)	4%	*p* < 0.0001	29	1,547	FEM	0.59 (0.48, 0.73)	42%	*p* < 0.00001	*3*	*145*	FEM	0.61 (0.24, 1.58)	10%	*p* = 0.31
SEC vs. carboplatin (CBP)	1	75	No	4.42 (1.57, 12.4)	No	*p* = 0.005	1	75	FEM	0.20 (0.07, 0.57)	No	*p* = 0.003	1	75	No	0.24 (0.06, 0.95)	0%	*p* = 0.04
SEC vs. mitomycin-C (MMC)	1	40	No	3.05 (0.66, 14.1)	No	*p* = 0.15	1	40	No	0.20 (0.05, 0.83)	No	*p* = 0.03	No	No	No	No	No	No
SEC vs. DDP and MMC	1	116	No	0.76 (0.33, 1.75)	No	*p* = 0.52	1	116	No	0.36 (0.15, 0.86)	No	*p* = 0.02	1	116	No	0.45 (0.08, 2.55)	No	*p* = 0.33
SEC vs. interleukin-2 (IL-2)	5	318	FEM	1.73 (1.03, 2.88)	0%	*p* = 0.04	5	318	FEM	0.32 (0.19, 0.53)	0%	*p* < 0.00001	No	No	No	No	No	No
SEC vs. rmhTNF	1	56	No	0.44 (0.14, 1.40)	No	*p* = 0.16	1	56	No	3.16 (1.0, 10.0)	No	*p* = 0.05	1	56	No	3.63 (0.64, 20.6)	No	*p* = 0.15
SEC vs. mycobacteria	1	52	No	0.67 (0.16, 2.71)	No	*p* = 0.57	1	52	No	0.88 (0.23, 3.33)	No	*p* = 0.84	1	52	No	0.20 (0.01, 4.38)	No	*p* = 0.31
SEC vs. sapylin	1	60	No	3.10 (0.12, 79.23)	No	*p* = 0.49	1	60	No	1.14 (0.41, 3.17)	No	*p* = 0.80	No	No	No	No	No	No
SEC vs. lentinan	1	40	No	1.86 (0.52, 6.61)	No	*p* = 0.34	1	40	No	0.62 (0.16, 2.43)	No	*p* = 0.49	No	No	No	No	No	No
SEC vs. elemene	1	52	No	0.91 (0.23, 3.61)	No	*p* = 0.89	1	52	No	0.78 (0.16, 3.91)	No	*p* = 0.77	No	No	No	No	No	No
**Staphylococcal enterotoxin C plus chemical agent (****Figures 3B,C**; **Appendix 3**; **Supplementary Figures S3****–****S5****)**																		
SEC plus cisplatin (DDP)	77	4,819	FEM	2.59 (2.28, 2.95)	0%	*p* < 0.00001	79	4,924	FEM	0.20 (0.18, 0.23)	0%	*p* < 0.00001	*13*	*789*	FEM	0.27 (0.16, 0.47)	0%	*p* < 0.00001
SEC plus carboplatin (CBP)	3	119	FEM	3.04 (1.30, 7.12)	0%	*p =* 0.01	3	119	FEM	0.18 (0.07, 0.46)	0%	*p =* 0.0003	No	No	No	No	No	No
SEC plus nedaplatin (NDP)	1	58	No	4.70 (0.92, 24.10)	No	*p =* 0.06	1	58	No	0.17 (0.05, 0.55)	No	*p =* 0.003	No	No	No	No	No	No
SEC plus bleomycin (BLM)	4	316	FEM	2.71 (1.68, 4.36)	0%	*p* < 0.0001	4	316	FEM	0.20 (0.12, 0.36)	0%	*p* < 0.00001	2	146	FEM	0.16 (0.04, 0.56))	0%	*p* = 0.005
SEC plus mitomycin-C (MMC)	3	146	FEM	2.06 (0.91, 4.67)	0%	*p =* 0.08	3	146	FEM	0.21 (0.10, 0.44)	0%	*p* < 0.0001	No	No	No	No	No	No
SEC plus etoposide (VP-16)	2	136	FEM	1.83 (0.90, 3.75)	0%	*p =* 0.10	2	136	FEM	0.17 (0.08, 0.39)	0%	*p* < 0.0001	No	No	No	No	No	No
SEC plus 5-fluorouracil (5-FU)	2	122	FEM	3.60 (1.48, 8.75)	0%	*p =* 0.005	2	122	FEM	0.17 (0.07, 0.39)	0%	*p* < 0.0001	1	62	FEM	0.10 (0.01, 0.82)	No	0.03
SEC plus mitoxantrone (MTZ)	1	58	No	4.68 (0.94, 23.35)	No	*p* = 0.06	1	58	No	0.08 (0.02, 0.30)	No	*p* = 0.0002	1	58	No	0.15 (0.01, 1.58)	No	*p* = 0.12
SEC plus adriamycin (ADM)	1	58	No	2.39 (0.74, 7.66)	No	*p* = 0.14	1	58	No	0.18 (0.04, 0.76)	No	*p* = 0.02	No	No	No	No	No	No
SEC plus docetaxel	1	58	No	1.64 (0.41, 6.58)	No	*p* = 0.49	1	58	No	0.22 (0.06, 0.81)	No	*p* = 0.02	1	58	No		No	*p* = 0.31
SEC plus DDP and VP-16	1	58	No	1.68 (0.25, 11.27)	No	*p* = 0.60	1	58	No	0.75 (0.22, 2.57)	No	*p* = 0.65	No	No	No	No	No	No
SEC plus ADM, 5 FU/CBP	1	58	No	75.0 (12.5, 448.0)	No	*p* < 0.00001	1	58	No	0.02 (0.00, 0.12)	No	*p* < 0.0001	1	58	No	0.06 (0.01, 0.54)	No	*p* = 0.01
SEC plus ADM and DDP	1	58	No	2.14 (0.62, 7.39)	No	*p* = 0.23	1	58	No	0.12 (0.03, 0.39)	No	*p* = 0.0005	No	No	No	No	No	No

*rmhTNF, recombinant modified human tumor necrosis factor; OR, odds ratio; CI, confidence interval; SM, statistical method; FEM, fixed-effects model*.

In SEC-plus chemical agents, the 99 *trials* reported ten protocols as SEC plus DDP, CBP, NDP, BLM, MMC, 5-FU, VP-16, MTZ, ADM, or docetaxel (Table 2; Figures 3B,C; Supplementary Figure S5). The Cochran's χ^2^-test and *I*^2^ statistic *found* no heterogeneity; we pooled the OR using a FEM. Compared with chemical agents alone, the results determined that the SEC plus DDP, BLM or 5-FU significantly improved the CR [OR = 2.59, 95% CI (2.28, 2.95), *p* < 0.00001; OR = 2.71, 95% CI (1.68, 4.36), *p* < 0.0001; OR = 3.60, 95% CI (1.48, 8.75), *p* = 0.005], decreased the failure [OR = 0.20, 95% CI (0.18, 0.23), *p* < 0.00001; OR = 0.20, 95% CI (0.12, 0.36), *p* < 0.00001; OR = 0.17, 95% CI (0.08, 0.39), *p* < 0.0001], and disease progression [OR = 0.27, 95% CI (0.16, 0.47), *p* < 0.00001; OR = 0.16, 95% CI (0.04, 0.56), *p* = 0.005; OR = 0.10, 95% CI (0.01, 0.82), *p* = 0.03]. The SEC plus CBP only improved the CR [OR = 3.04, 95% CI (1.30, 7.12), *p* = 0.01] and decreased the failure [OR = 0.18, 95% CI (0.07, 0.46), *p* = 0.0003]. No statistical difference was *found between other comparisons*.

### Overall Survivals

**Figure 4:**
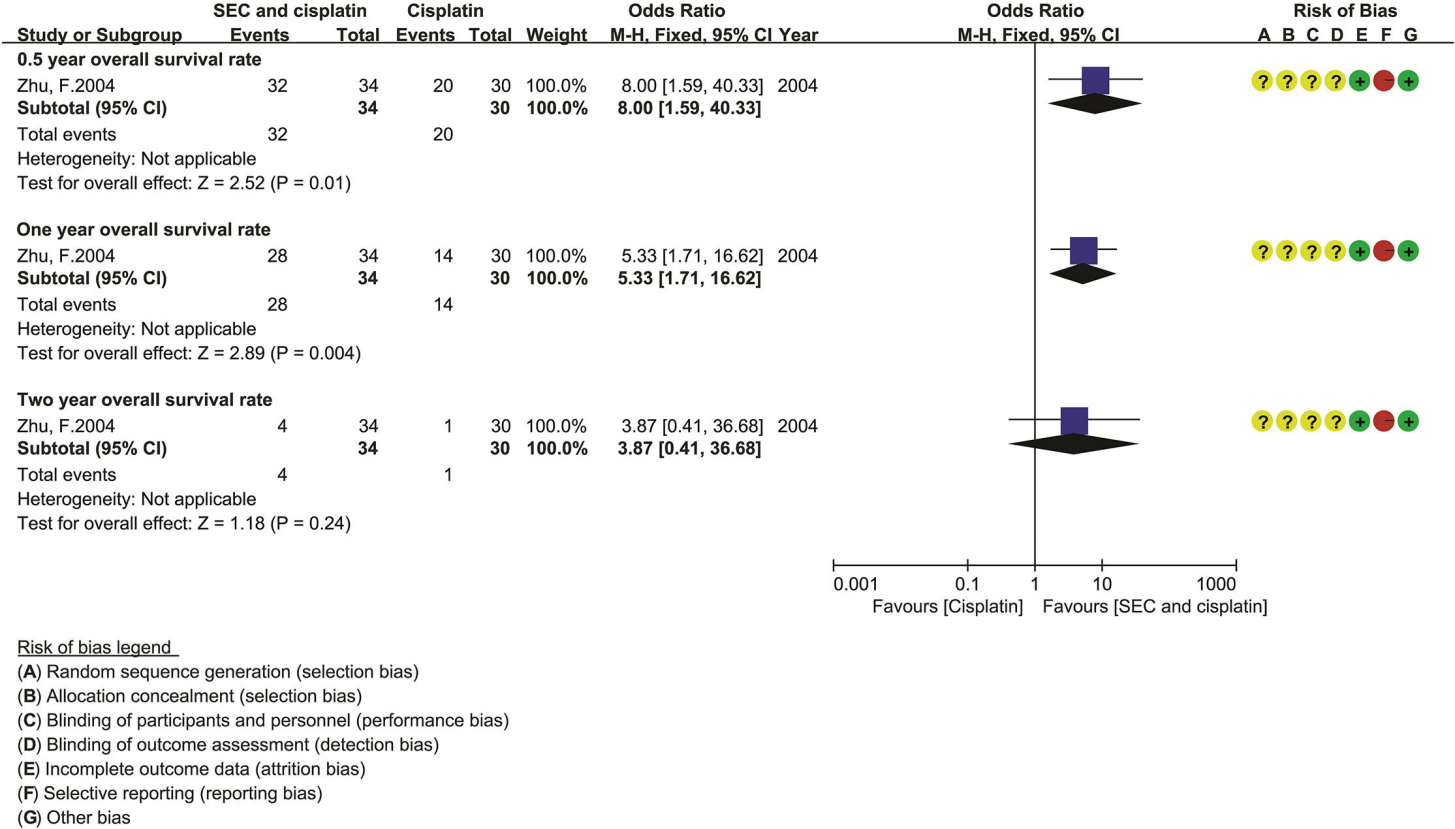
The overall survival of SEC and cisplatin. SEC, staphylococcal enterotoxin C; CI, confidence interval.

Only one trial reported the OS rate (Figure 4). Compared with DDP alone, the statistical analysis showed that the SEC and DDP perfusion significantly improved the 0.5-year OS rate [OR = 8.00, 95% CI (1.59–40.33), *p* = 0.01] and 1 year OS rate [OR = 5.33, 95% CI (1.71–16.62), *p* = 0.004].

### Quality of Life

**Figure 5:**
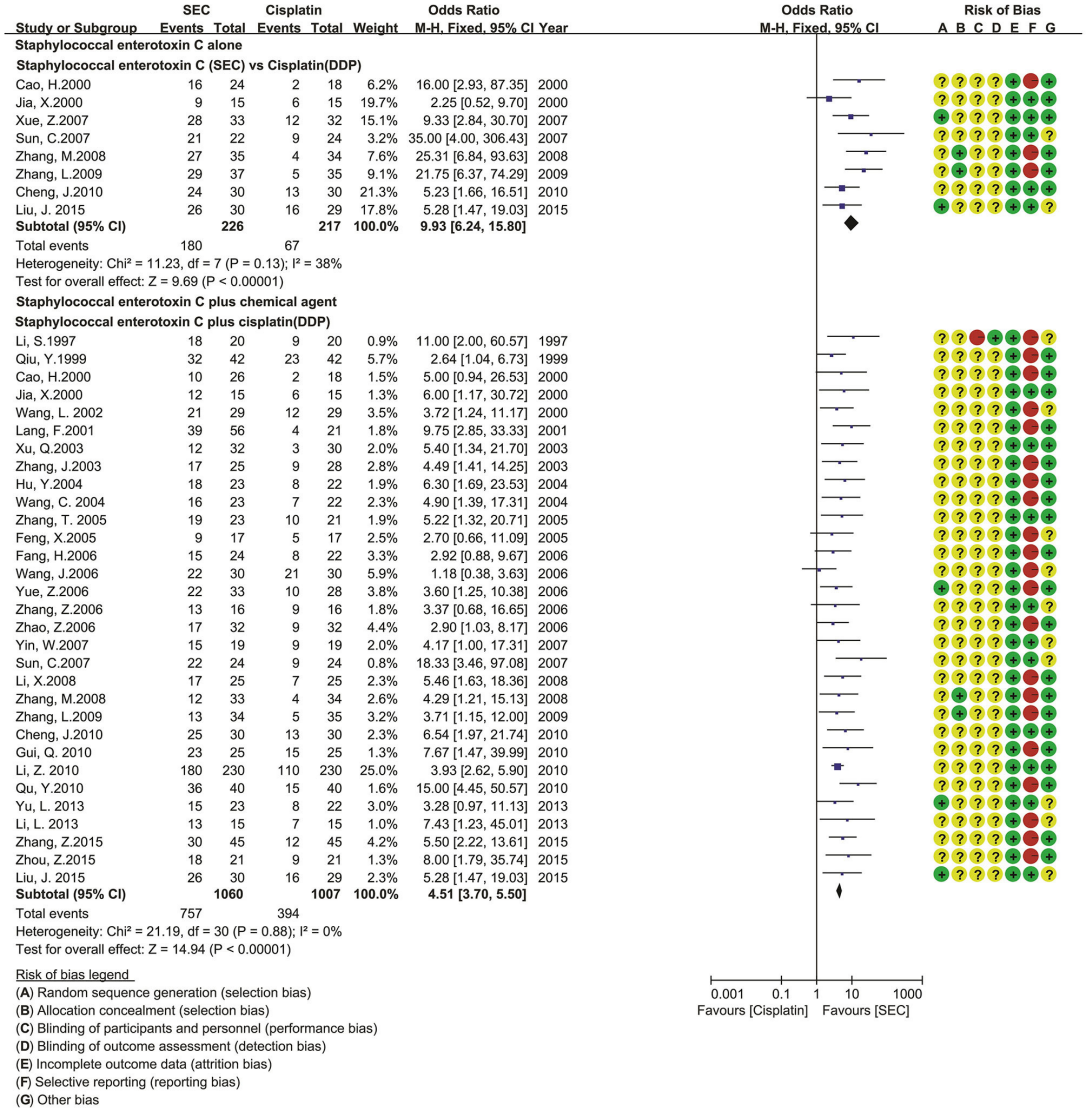
The forest plot of quality of life. CI, confidence interval.

Eight *trials* containing 443 patients reported the QOL in SEC alone, and 31 containing 2,067 patients reported the QOL in SEC and DDP perfusion, and limited trials reported other nine protocols. The Cochran's χ^2^-test and *I*^2^ statistic *only found* a minimal heterogeneity in SEC vs. DDP (*I*^2^ = 38%). The OR was pooled by using a FEM. Compared with DDP alone, the meta-analysis result determined that the SEC alone or/and DDP perfusion significantly improved the QOL [OR = 9.93 95% CI (6.24–15.80), *p* < 0.00001, and OR = 4.51, 95% CI (3.70–5.50), *p* < 0.00001] (Figure 5).

### Adverse Events

Twenty-six *trials reported the adverse events in SEC alone* (40, 41, 46, 47, 51, 52, 56, 62, 64, 68, 73–75, 77, 79, 83, 86, 87, 115, 118, 125, 130, 139, 140, 143, 147), *and 75 reported the adverse events in SEC and DDP perfusion* (19, 20, 37, 39–47, 49–53, 56, 57, 59, 62, 64–66, 69–73, 75, 77–79, 81–85, 87, 89–93, 95, 98, 100–102, 104, 105, 107–110, 112, 114, 116–121, 123, 126–129, 133, 136, 137, 139, 143, 145, 148). Limited trials reported others. In SEC alone, the Cochran's χ^2^-test and *I*^2^ statistic only *found* a statistical heterogeneity in gastrointestinal reaction (*I*^2^ = 52%) and minimal heterogeneity in myelosuppression (*I*^2^ = 19%), leukopenia (*I*^2^ = 8%), and fever (*I*^2^ = 29%) (Table 3; Appendix 4; Supplementary Figures S6–S12); we pooled the data of gastrointestinal reaction using a REM, and other data using a FEM. Compared with DDP alone, the results determined that the SEC alone showed lower myelosuppression [OR = 0.19, 95% CI (0.07–0.53), *p* = 0.002], leukopenia [OR = 0.11, 95% CI (0.05–0.23), *p* < 0.00001], gastrointestinal reaction [OR = 0.12, 95% CI (0.06–0.26), *p* < 0.00001], hepatic dysfunction [OR = 0.22, 95% CI (0.05–0.94), *p* = 0.04], renal dysfunction [OR = 0.13, 95% CI (0.04–0.46), *p* = 0.002], and a higher fever [OR = 6.66, 95% CI (4.30–10.32), *p* < 0.00001]. However, the results revealed no statistical differences in cardiac dysfunction and thoracodynia. Additionally, most trials ignored the thoracentesis or SEC-related adverse events.

**Table 3:** Meta-analysis results of adverse events (Appendix 4; Supplementary Figures S6–S12).

**Indicators**	**Trials**	**Staphylococcal enterotoxin C (events/total)**	**Cisplatin (events/total)**	**Statistical method**	**Odds ratios 95% CI**	***I*** **^2^**	***P***
**Staphylococcal enterotoxin C alone**							
**Staphylococcal enterotoxin C vs. cisplatin**							
Myelosuppression	5	3/138	20/141	Fixed-effects model	0.19 (0.07, 0.53)	19%	*p* = 0.002
Leukopenia	9	5/210	54/204	Fixed-effects model	0.11 (0.05, 0.23)	8%	*p* < 0.00001
Gastrointestinal reaction	15	25/389	158/386	Random-effects model	0.12 (0.06, 0.26)	52%	*p* < 0.00001
Hepatic dysfunction	6	2/147	9/148	Fixed-effects model	0.22 (0.05, 0.94)	0%	*p* = 0.04
Renal dysfunction	8	2/192	18/193	Fixed-effects model	0.13 (0.04, 0.46)	0%	*p* = 0.002
Cardiac dysfunction	1	1/14	0/12	No	2.78 (0.10, 74.70)	0%	*p* = 0.54
Thoracodynia	12	47/305	34/305	Fixed-effects model	1.51 (0.94, 2.44)	0%	*p* = 0.09
Fever	16	149/423	45/421	Random-effects model	6.66 (4.30, 10.32)	29%	*p* < 0.00001
**Staphylococcal enterotoxin C and chemical agent**							
**Staphylococcal enterotoxin C plus cisplatin**							
Myelosuppression	13	17/607	35/592	Fixed-effects model	0.44 (0.24, 0.80)	0%	*p* = 0.007
Leukopenia	27	96/712	191/673	Fixed-effects model	0.36 (0.27, 0.48)	0%	*p* < 0.00001
Gastrointestinal reaction	47	334/1,511	555/1,461	Random-effects model	0.43 (0.36, 0.51)	33%	*p* < 0.00001
Hepatic dysfunction	18	52/716	41/681	Fixed-effects model	1.33 (0.85, 2.09)	0%	*p* = 0.21
Renal dysfunction	18	4/466	16/431	Fixed-effects model	0.26 (0.10, 0.72)	0%	*p* = 0.009
Cardiac dysfunction	8	0/143	0/129	No	No	No	No
Thoracodynia	32	195/1,130	167/1,102	Fixed-effects model	1.17 (0.93, 1.47)	36%	*p* = 0.18
Fever	50	323/1,585	141/1,527	Fixed-effects model	2.70 (2.16, 3.36)	46%	*p* < 0.00001

*CI, confidence interval*.

In SEC and DDP perfusion, the Cochran's χ^2^-test and *I*^2^-statistic *only found* a minimal heterogeneity in gastrointestinal reaction (*I*^2^ = 33%), thoracodynia (*I*^2^ = 36%), and fever (*I*^2^ = 46%) (Table 3; Appendix 4; Supplementary Figures S6–S12); we pooled *all the data* using a FEM. Compared with DDP alone, the results determined that the perfusion protocol showed a low incidence rate of myelosuppression [OR = 0.44, 95% CI (0.24–0.80), *p* = 0.007], leukopenia [OR = 0.36, 95% CI (0.27–0.48), *p* < 0.00001], gastrointestinal reaction [OR = 0.43, 95% CI (0.36–0.51), *p* < 0.00001], renal dysfunction [OR = 0.26, 95% CI (0.10–0.72), *p* = 0.009], and a high fever [OR = 2.70, 95% CI (2.16–3.36), *p* < 0.00001], and no difference in thoracodynia and hepatic dysfunction. Additionally, six trials reported no cardiotoxicity, and most ignored the thoracentesis or SEC-related adverse events.

### Subgroups and Meta-Regression Analysis

*Only the SEC and DDP perfusion protocol included enough trials*. So, a subgroup analysis was performed to reveal their *potential* clinical heterogeneity and determine the effects of variables on clinical responses. The tumors included miscellaneous tumors and lung cancer. The subgroup analysis revealed that the SEC and DDP perfusion significantly improved the CR with a low failure in patients with both conditions (Table 4a; Supplementary Figures S14, S16). The pleural fluid was small to large volume, moderate to large or large; the KPS scores were ≥40, ≥50, or ≥60; the AST was ≥2 or 3 months; and the treatment history was primary treatment or unclear. The perfusion could significantly improve the clinical responses in MPE with moderate to large (Table 4b; Supplementary Figures S18, S20), KPS score ≥40, ≥50, or ≥60 (Table 4c; Supplementary Figures S22, S24), AST ≥ 2 or 3 months (Table 4e; Supplementary Figures S30, S32), and primary treatment (Table 4d; Supplementary Figures S26, S28).

**Table 4:** Subgroups and meta-regression analysis (Appendix 5; Supplementary Figures S14–S65).

**Subgroups**	**Complete response**					**Pleurodesis failure**				
	**Trials**	**Cases**	**Odds ratios (95%CI)**	**Univariable**	**Multiple**	**Trials**	**Cases**	**Odds ratios (95%CI)**	**Univariable**	**Multiple**
**(a) Subgroups analysis** ***via*** **primary tumors (****Supplementary Figures S14****–****S17****)**										
Miscellaneous tumors	51	2,858	2.99 (2.51, 3.57)	0.02	0.71	53	2,963	0.20 (0.16, 0.23)	0.58	0.96
Lung cancer	26	1,961	2.18 (1.79, 2.64)			26	1,961	0.21 (0.17, 0.26)		
**(b) Subgroups analysis** ***via*** **pleural effusion (****Supplementary Figures S18****–****S21****)**										
Small to large	3	211	2.81 (1.43, 5.53)	0.23	0.82	3	211	0.12 (0.06, 0.23)	0.45	0.86
Moderate to large	17	939	3.22 (2.38, 4.36)			19	1,044	0.22 (0.16, 0.29)		
Large	1	44	2.22 (0.63, 7.82)			1	44	0.19 (0.05, 0.73)		
Unclear	56	3,625	2.46 (2.12, 2.85)			56	3,625	0.20 (0.17, 0.24)		
**(c) Subgroups analysis** ***via*** **KPS score (****Supplementary Figures S22****–****S25****)**										
Karnofsky performance status score (≥40)	7	385	3.32 (2.09, 5.27)	0.90	0.97	7	385	0.15 (0.09, 0.24)	0.77	0.94
Karnofsky performance status score (≥50)	20	1,479	2.12 (1.69, 2.65)			22	1,584	0.25 (0.19, 0.32)		
Karnofsky performance status score (≥60)	18	1,063	3.22 (2.38, 4.36)			18	1,063	0.18 (0.13, 0.24)		
Karnofsky performance status score (unclear)	32	1,892	2.64 (2.15, 3.24)			32	1,892	0.19 (0.15, 0.24)		
**(d) Subgroups analysis** ***via*** **treatment history (****Supplementary Figures S26****–****S29****)**										
Primary treatment	2	124	3.45 (1.57, 7.57)	0.45	0.93	3	169	0.20 (0.10, 0.41)	0.95	0.71
Unclear	75	4,695	2.57 (2.26, 2.94)			76	4,755	0.20 (0.17, 0.23)		
**(e) Subgroups analysis** ***via*** **anticipated survival time (****Supplementary Figures S30****–****S33****)**										
Anticipated survival time (>2 months)	4	669	2.25 (1.63, 3.11)	0.67	0.85	4	669	0.25 (0.17, 0.35)	0.66	0.94
Anticipated survival time (≥3 months)	27	1,615	2.58 (2.05, 3.23)			27	1,615	0.18 (0.14, 0.23)		
Others (<1 month or unclear)	46	2,535	2.72 (2.27, 3.27)			48	2,640	0.20 (0.17, 0.24)		
**(f) Subgroups analysis** ***via*** **indwelling pleural catheter (****Supplementary Figures S34****–****S37****)**										
Indwelling pleural catheter	39	2,493	2.74 (2.29, 3.29)	0.60	0.63	40	2,553	0.22 (0.18, 0.27)	0.65	0.48
Thoracentesis	22	1,367	2.36 (1.86, 3.00)			23	1,412	0.18 (0.14, 0.23)		
Unclear	16	959	2.59 (1.93, 3.48)			16	959	0.18 (0.13, 0.25)		
**(g) Subgroups analysis** ***via*** **staphylococcal enterotoxin C dosage (****Supplementary Figures S38****–****S41****)**										
Staphylococcal enterotoxin C (80 ng, 8 ml, 2,000 IU)	10	893	1.95 (1.46, 2.60)	0.15	0.08	11	938	0.26 (0.20, 0.35)	0.11	0.12
Staphylococcal enterotoxin C (100 ng, 10 ml, 2,500 IU)	5	245	2.77 (1.38, 5.54)			5	245	0.28 (0.16, 0.50)		
Staphylococcal enterotoxin C (120 ng, 12 ml, 3,000 IU)	7	494	2.83 (1.81, 4.42)			7	494	0.20 (0.13, 0.31)		
Staphylococcal enterotoxin C (160 ng, 16 ml, 4,000 IU)	8	337	2.50 (1.53, 4.10)			8	337	0.24 (0.14, 0.39)		
Staphylococcal enterotoxin C (200 ng, 20 ml, 5,000 IU)	26	1,533	3.34 (2.65, 4.22)			26	1,533	0.16 (0.13, 0.21)		
Staphylococcal enterotoxin C (100–200 ng, 10–20 ml, 2,500–5,000 IU)	12	747	2.00 (1.48, 2.70)			12	747	0.19 (0.13, 0.28)		
Staphylococcal enterotoxin C (>200 ng, >20 ml, >5,000 IU)	6	339	3.40 (2.05, 5.63)			6	339	0.19 (0.11, 0.32)		
Staphylococcal enterotoxin C (Unable to group or unclear)	3	231	2.52 (1.26, 5.04)			4	291	0.17 (0.09, 0.31)		
**(h) Subgroups analysis** ***via*** **treatment frequency (****Supplementary Figures S42****–****S45****)**										
One to two times/week	69	4,301	2.53 (2.21, 2.90)	0.69	0.64	71	4,406	0.21 (0.18, 0.25)	0.02	0.03
Others (unable to group or unclear)	8	518	3.36 (2.16, 5.23)			8	518	0.12 (0.07, 0.19)		
**(i) Subgroups analysis** ***via*** **treatment times (****Supplementary Figures S46****–****S49****)**										
One to four times	59	3,746	2.49 (2.16, 2.88)	0.72	0.76	61	3,851	0.21 (0.18, 0.25)	0.66	0.40
>4 times	7	443	3.05 (1.91, 4.88)			7	443	0.15 (0.09, 0.23)		
Others (Unable to group or unclear)	11	630	3.02 (2.09, 4.36)			11	630	0.18 (0.12, 0.26)		
**(j) Subgroups analysis** ***via*** **cisplatin dosage (****Supplementary Figures S50****–****S53****)**										
Cisplatin (30–40 mg each time)	9	487	3.72 (2.37, 5.83)	0.34	0.05	9	487	0.18 (0.12, 0.28)	0.22	0.39
Cisplatin (50–60 mg each time)	33	2,240	2.47 (2.04, 2.99)			33	2,240	0.22 (0.18, 0.27)		
Cisplatin (70–100 mg each time)	20	1,238	2.20 (1.73, 2.80)			21	1,283	0.20 (0.15, 0.26)		
Cisplatin (unclear or ungroupable)	15	854	3.32 (2.39, 4.60)			16	914	0.16 (0.12, 0.23)		
**(k) Subgroups analysis** ***via*** **dosage difference in cisplatin (****Supplementary Figures S54****–****S57****)**										
Equivalent dosage	72	4,566	2.61 (2.28, 2.98)	0.75	0.97	74	4,671	0.20 (0.17, 0.23)	0.27	0.28
Low vs. high-dosage	5	253	2.38 (1.35, 4.20)			5	253	0.29 (0.16, 0.54)		
**(l) Subgroups analysis** ***via*** **criterion (****Supplementary Figures S58****–****S61****)**										
Millar	13	1,181	2.37 (1.82, 3.08)	0.53	0.94	15	1,286	0.23 (0.18, 0.30)	0.28	0.70
Ostrowskimj	64	3,638	2.67 (2.30, 3.10)			64	3,638	0.19 (0.16, 0.23)		
**(m) Subgroups analysis** ***via*** **publication year (****Supplementary Figures S62****–****S65****)**										
Before 2010 year	46	2,479	2.84 (2.36, 3.42)	0.03	0.94	48	2,584	0.21 (0.17, 0.25)	0.59	0.92
From 2010 to now	31	2,340	2.37 (1.98, 2.85)			31	2,340	0.19 (0.16, 0.24)		

*KPS score, Karnofsky performance status score; CI, confidence interval*.

The SEC was mainly used with 100 ng (10 ml, 2,500 IU) to 200 ng (20 ml, 5,000 IU) per time, one time or two times a week, and lasting one to four times. The dosages of DDP were categorized into 30–100 mg per time. *In combinations* with DDP (30–40 mg, 50–60 mg, and 70–100 mg per time), mainly 50–60 mg per time, SEC could significantly improve the clinical responses (Tables 4g–j; Supplementary Figures S38, S40, S42, S44, S46, S48, S50, S52). Moreover, there were dosage differences between two groups. Like high dosage DDP, *the* SEC with low-dosage also significantly improved a similar response (Table 4k; Supplementary Figures S54, S57). The drainage was IPC or thoracentesis; the criterion was Ostrowskimj or Millar, and the publication year was before or after 2010 year. *The perfusion* achieved above effects under these conditions (Tables 4f,l,m; Supplementary Figures S34, S36, S58, S60, S62, S64). But the univariable meta-regression only revealed a correlation between tumor type and CR (*p* = 0.02), and between treatment frequency and pleurodesis failure (*p* = 0.02). The multiple meta-regression analysis further determined that the treatment frequency was associated with pleurodesis failure (Table 4).

### Publication Bias Analysis

In perfusion with SEC alone, more than ten trials were included for CR, pleurodesis failure, gastrointestinal reactions, thoracodynia, and fever. The funnel plot and Egger's test showed a publication bias in failure (*P* > |*t*| = 0.00001, Coef = −4.31, 95% CI −6.52 to −2.11), gastrointestinal reactions (*P* > |*t*| = 0.009, Coef = −2.6495%, CI −4.50 to −0.77), and the trials underestimated them. No publication bias was found in other outcomes, *which were objectively reported* (Table 5; Supplementary Figures S67, S68). In perfusion with SEC and DDP, *more than 10 trials were included for* CR, pleurodesis failure, disease progression, quality of life, myelosuppression, gastrointestinal reactions, leukopenia, thoracodynia, and fever. A publication bias was found in CR (*P* > |*t*| = 0.00001, Coef = 0.99, 95% CI, 0.50–1.49), failure (*P* > |*t*| = 0.004, Coef = −0.8, 95% CI, −1.33 to −0.26), gastrointestinal reactions (*P* > |*t*| = 0.03, Coef = −1.03, 95% CI, −1.95 to −0.11), and fever (*P* > |*t*| = 0.00001, Coef = 1.593, 95% CI, 0.77–2.40); the trials underestimated the failure and gastrointestinal reactions, and overestimated the CR and fever. No publication bias was found in others, *which were objectively reported* (Table 5; Supplementary Figures S71, S72, S77, S79).

**Table 5:** Publication bias risk (Appendix 6; Supplementary Figures S66–S79).

**Indicators**	**Included trials**	**Odds ratios 95% CI**	**Egger's test**			**Risk assessment**
			**Coefficient**	**95% CI**	***P* > |*t*|**	
**Staphylococcal enterotoxin C alone**						
Staphylococcal enterotoxin C vs. cisplatin (DDP)						
Complete response	29	1.69 (1.33, 2.15)	−0.084	−1.54 to 1.37	0.91	Objective
Pleurodesis failure	29	0.59 (0.48, 0.73)	−4.31	−6.52 to −2.11	0.00001	Underestimation
Gastrointestinal reactions	15	0.12 (0.08, 0.18)	−2.64	−4.50 to −0.77	0.009	Underestimation
Thoracodynia	12	1.51 (0.94, 2.44)	0.31	−1.64 to 2.26	0.73	Objective
Fever	16	6.66 (4.30, 10.32)	−0.39	−2.42 to 1.64	0.69	Objective
**Staphylococcal enterotoxin C and chemical agent**						
Staphylococcal enterotoxin C plus cisplatin (DDP)						
Complete response	77	2.59 (2.28, 2.95)	0.99	0.50 to 1.49	0.00001	Overestimation
Pleurodesis failure	79	0.20 (0.18, 0.23)	−0.8	−1.33 to −0.26	0.004	Underestimation
Disease progression	13	0.27 (0.16, 0.47)	0.09	−1.73 to 1.92	0.91	Objective
Quality of life	31	4.51 (3.70, 5.50)	0.75	−0.06 to 1.56	0.07	Objective
Myelosuppression	13	0.44 (0.24, 0.80)	0.28	−1.64 to 2.19	0.74	Objective
Leukopenia	27	0.36 (0.27, 0.48)	−0.33	−1.79 to 1.13	0.64	Objective
Gastrointestinal reactions	47	0.43 (0.36, 0.51)	−1.03	−1.95 to −0.11	0.03	Underestimation
Thoracodynia	32	1.17 (0.93, 1.47)	0.77	−0.13 to 1.64	0.09	Objective
Fever	50	2.70 (2.16, 3.36)	1.59	0.77 to 2.40	0.00001	Overestimation

*CI, confidence interval*.

### Sensitivity Analysis

In perfusion with SEC alone, all indicators involved poor and over- or under-estimated *trials*. *In SEC vs*. *DDP/IL-2*, the OR of CR, failure, QOL, and neutropenia had poor robustness *before and after removing the poor and over- or underestimation*, and others had good robustness (Table 6). In SEC and chemical agent perfusion, all indicators involved poor and over- or underestimated *trials*. *In SEC and DDP perfusion, the OR of disease progression, myelosuppression, and nephrotoxicity was poor robustness before and after removing the poor and underestimation. In SEC with BLM, 5-FU or MMC, the OR of CR, failure, and disease progression were poor robustness before and after removing the poor and over- or underestimation, and others had good robustness* (Table 6).

**Table 6:** Sensitivity analysis.

**Indicators**	**Before excludedtrials**				**Excluded poor and over/under-estimation**	**After excludedtrials**				**Sensitivity**
	**Trials**	**SM**	**OR (95%CI)**	***I*** **^2^**		**Trials**	**SM**	**OR (95%CI)**	***I*** **^2^**	
**Staphylococcal enterotoxin C alone**										
**Staphylococcal enterotoxin C vs. cisplatin (DDP)**										
Complete response	29	FEM	1.69 (1.33, 2.15)	4%	Poor*: (38, 41, 47, 48, 51, 52, 56, 64, 68, 75, 79, 115, 118, 125, 130, 134, 143, 147), Over*: (87)	10	FEM	1.23 (0.80, 1.90)	0%	Poor
Pleurodesis failure	29	FEM	0.59 (0.48, 0.73)	42%	Poor*: (38, 41, 47, 48, 51, 52, 56, 64, 68, 75, 79, 115, 118, 125, 130, 134, 143, 147), Under*: (86, 87)	9	FEM	1.08 (0.74, 1.58)	0%	Poor
Disease progression	3	FEM	0.61 (0.24, 1.58)	10%	Poor*: No, Under*: No	3	FEM	0.61 (0.24, 1.58)	10%	Robustness
Quality of life	8	FEM	9.93 (6.24, 15.80)	38%	Poor*: (64, 68, 143), Over*: (46, 73, 86, 87)	1	No	2.25 (0.52, 9.70)	No	Poor
Myelosuppression	5	FEM	0.19 (0.07, 0.53)	19%	Poor*: (41), Under*: (73)	3	FEM	0.25 (0.07, 0.93)	0%	Robustness
Neutropenia	9	FEM	0.11 (0.05, 0.23)	8%	Poor*: (52, 118), Under*: (62, 74, 77, 118, 139)	3	FEM	0.34 (0.08, 1.50)	0%	Poor
Thrombocytopenia	2	FEM	0.09 (0.00, 2.02)	No	Poor*: (118), Under*: No	1	No	0.09 (0.00, 2.02)	No	Robustness
Gastrointestinal reactions	15	REM	0.12 (0.08, 0.18)	52%	Poor*: (52, 68, 75, 79, 118), Under*: (62, 73–75, 77, 79, 87, 118, 139, 140)	3	FEM	0.37 (0.14, 0.99)	0%	Robustness
Hepatotoxicity	6	FEM	0.22 (0.05, 0.94)	0%	Poor*: No; Under*: No	6	FEM	0.22 (0.05, 0.94)	0%	Robustness
Nephrotoxicity	8	FEM	0.13 (0.04, 0.46)	0%	Poor*: (125), Under*: (87)	6	FEM	0.23 (0.05, 0.98)	0%	Robustness
Fever	16	FEM	6.66 (4.30, 10.32)	29%	Poor*: (41, 52, 68, 75, 79), Under*: (46, 52, 68, 73, 86)	8	FEM	3.14 (1.57, 6.29)	0%	Robustness
Thoracodynia	12	FEM	1.51 (0.94, 2.44)	0%	Poor*: (41, 52, 68), Under*: No	9	FEM	1.62 (0.94, 2.79)	0%	Robustness
**Staphylococcal enterotoxin C vs. interleukin-2 (IL-2)**										
Complete response	5	FEM	1.73 (1.03, 2.88)	0%	Poor*: (38, 41, 47, 48, 51), Under*: No	No	No	No	No	Poor
Treatment failure	8	FEM	0.32 (0.19, 0.53)	0%	Poor*: (38, 41, 47, 48, 51), Under*: (48)	No	No	No	No	Poor
**Staphylococcal enterotoxin C and chemical agent**										
**Staphylococcal enterotoxin C plus cisplatin**										
Complete response	77	FEM	2.59 (2.28, 2.95)	0%	Poor*: (19, 20, 37, 38, 41–45, 47–49, 51, 52, 56, 57, 61, 63–66, 69, 70, 72, 75, 78, 79, 81, 84, 89, 91, 93, 98, 100, 102, 104, 107, 108, 110, 112, 114, 116–118, 120, 123, 126–129, 133, 136, 137, 143, 145, 148), Over*: (20, 38, 45, 46, 48, 65, 66, 71, 75, 78, 79, 95, 102, 105, 110, 119, 120, 133, 136, 145)	17	FEM	2.13 (1.59, 2.85)	0%	Robustness
Pleurodesis failure	79	FEM	0.20 (0.18, 0.23)	0%	Poor*: (19, 20, 37, 38, 41–45, 47–49, 51, 52, 56, 57, 61, 63–66, 69, 70, 72, 75, 78, 79, 81, 84, 89, 91, 93, 98, 100, 102, 104, 107, 108, 110, 112, 114, 116–118, 120, 123, 126–129, 133, 136, 137, 143, 145, 148), Under*: (19, 20, 37, 38, 40, 41, 43–46, 48–53, 56, 59, 63–66, 69, 71–73, 75, 77–79, 81, 83, 85, 87, 89–92, 95, 98, 101, 102, 104, 105, 107, 110, 112, 116, 117, 119, 126, 127, 129, 133, 136, 139, 143, 148, 149)	4	FEM	0.29 (0.15, 0.58)	0%	Robustness
Disease progression	13	FEM	0.27 (0.16, 0.47)	0%	Poor*: (37, 44, 70, 72, 84, 100, 126, 129, 133, 148), Under*: (105, 133)	2	FEM	0.25 (0.05, 1.25)	0%	Poor
Quality of life	31	FEM	4.51 (3.70, 5.50)	0%	Poor*: (19, 20, 44, 45, 57, 69, 72, 75, 79, 81, 89, 91, 93, 98, 108, 112, 116, 120, 129, 133, 143), Over*: (19, 20, 44–46, 57, 69, 71–73, 75, 79, 81, 87, 89, 91, 101, 112, 116, 120, 121, 129, 133, 139)	3	FEM	3.57 (1.60, 7.94)	0%	Robustness
Myelosuppression	13	FEM	0.44 (0.24, 0.80)	0%	Poor*: (41, 104, 107, 127, 128), Under*: (73)	7	FEM	0.51 (0.25, 1.05)	0%	Poor
Neutropenia	27	FEM	0.36 (0.27, 0.48)	0%	Poor*: (20, 66, 69, 72, 81, 89, 91, 110, 114, 118, 128, 145), Under*: (72, 89, 91, 110, 121, 139)	13	FEM	0.56 (0.37, 0.84)	0%	Robustness
Thrombocytopenia	3	FEM	0.97 (0.28, 3.35)	0%	Poor*: (118), Under*: No	2	FEM	0.97 (0.28, 3.35)	0%	Robustness
Anemia	3	FEM	0.71 (0.14, 3.63)	No	Poor*: (118), Under*: No	2	FEM	0.71 (0.14, 3.63)	No	Robustness
Gastrointestinal reactions	47	FEM	0.43 (0.36, 0.51)	33%	Poor*: (19, 20, 37, 43, 49, 52, 66, 69, 72, 75, 79, 81, 84, 89, 91, 98, 100, 107, 114, 118, 120, 123, 126, 137, 145), Under*: (19, 20, 72, 73, 75, 79, 89–91, 95, 100, 101, 114, 120, 126, 137)	17	FEM	0.65 (0.51, 0.84)	0%	Robustness
Hepatotoxicity	18	FEM	1.33 (0.85, 2.09)	0%	Poor*: (70, 78, 84, 102, 108, 128, 136), Under*: No	11	FEM	1.33 (0.85, 2.09)	0%	Robustness
Nephrotoxicity	18	FEM	0.26 (0.10, 0.72)	0%	Poor*: (70, 78, 84, 102, 108, 128, 136), Under*: No	10	FEM	0.38 (0.11, 1.34)	0%	Poor
Fever	50	FEM	2.70 (2.16, 3.36)	46%	Poor*: (19, 37, 41, 43, 44, 49, 52, 57, 65, 75, 79, 81, 84, 89, 91, 98, 100, 102, 107, 114, 118, 120, 123, 126, 128, 136, 137, 145), Under*: (95)	21	FEM	1.94 (1.43, 2.63)	44%	Robustness
Thoracodynia	32	FEM	1.17 (0.93, 1.47)	36%	Poor*: (37, 41, 43, 44, 49, 52, 57, 65, 66, 102, 107, 120, 126), Under*: No	19	FEM	0.95 (0.72, 1.24)	14%	Robustness
**Staphylococcal enterotoxin C plus carboplatin (CBP)**										
Complete response	3	FEM	3.04 (1.30, 7.12)	0%	Poor*: No, Over*: No	3	FEM	3.04 (1.30, 7.12)	0%	Robustness
Pleurodesis failure	3	FEM	0.18 (0.07, 0.46)	0%	Poor*: No, Under*: (96)	2	FEM	0.18 (0.06, 0.59)	No	Robustness
**Staphylococcal enterotoxin C plus bleomycin (BLM)**										
Complete response	4	FEM	2.71 (1.68, 4.36)	0%	Poor*: (76, 80, 94), Over*: (76, 80, 142)	No	No	No	No	Poor
Pleurodesis failure	4	FEM	0.20 (0.12, 0.36)	0%	Poor*: (76, 80, 94), Under*: (76, 80, 142)	No	No	No	No	Poor
Disease progression	2	FEM	0.16 (0.04, 0.56)	0%	Poor*: (80, 94), Under*: (80)	No	No	No	No	Poor
**Staphylococcal enterotoxin C plus 5-fluorouracil (5-FU)**										
Complete response	2	FEM	3.60 (1.48, 8.75)	0%	Poor*: (97, 124), Over*: (124)	No	No	No	No	Poor
Pleurodesis failure	2	FEM	0.17 (0.07, 0.39)	0%	Poor*: (97, 124), Under*: (124)	No	No	No	No	Poor
**Staphylococcal enterotoxin C plus mitomycin-C (MMC)**										
Complete response	3	FEM	2.06 (0.91, 4.67)	0%	Poor*: (99, 113, 135), Over*: No	No	No	No	No	Poor
Pleurodesis failure	3	FEM	0.21 (0.10, 0.44)	0%	Poor*: (99, 113, 135), Under*: (113, 135)	No	No	No	No	Poor
**Staphylococcal enterotoxin C plus etoposide (VP-16)**										
Complete response	2	FEM	1.83 (0.90, 3.75)	0%	Poor*: No, Over*: No	2	FEM	1.83 (0.90, 3.75)	0%	Robustness
Pleurodesis failure	2	FEM	0.17 (0.08, 0.39)	0%	Poor*: No, Under*: No	2	FEM	0.17 (0.08, 0.39)	0%	Robustness

SM, statistical method; FEM, fixed-effects model; REM, random-effects model; OR, odds ratio; CI, confidence interval; Poor^*^: poor trials that had at least one domain being considered as high risk of bias; Over^*^or Under^*^: over-or underestimated trials whose results had significant difference and beneficial to staphylococcal enterotoxin C perfusion.

### Quality of Pieces of Evidence

In methodology, *21 poor trials were involved in SEC perfusion alone* (38, 41, 47, 48, 51, 52, 56, 64, 68, 75, 79, 88, 115, 118, 122, 125, 130, 134, 143, 146, 147). *In SEC vs*. *DDP/IL-2*, the OR of CR, failure, QOL, and neutropenia had poor robustness. Therefore, we downgraded the quality with two grades. While others had robustness, we downgraded the quality one grade. The statistical heterogeneity was found for CR, failure, QOL, and neutropenia in SEC vs. DDP, and, for CR and failure in SEC vs. IL-2, the sensitivity analysis showed poor robustness. The sample size for disease progression, QOL and hepatotoxicity was lower than 300 subjects. A publication bias was found *in* failure and gastrointestinal reactions, and the failure had poor robustness. So, we downgraded their quality one grade. *Finally*, we summarized a “moderate” quality for gastrointestinal reactions, nephrotoxicity, thoracodynia, and fever in SEC vs. DDP, and a “low” to “very low” for others (Table 7).

**Table 7:** A GRADE evidence profile.

**Indicators (RCTs)**	**Quality assessment**					**Malignant pleural effusion**		**Clinical effectiveness and safety**		**Quality**
	**i**	**ii**	**iii**	**iv**	**v**	**SEC**	**Pleurodesis agents**	**Odds ratios (95% CI)**	**Absolute effects**	
**Staphylococcal enterotoxin C (SEC) alone**										
**Staphylococcal enterotoxin C vs. cisplatin (DDP)**										
Complete response (29)	Veryserious^d^	Serious^h^	No	No	None	238/776 (30.7%)	166/771 (21.5%)	1.69 (1.33–2.15)	101 more per 1,000 (from 52 more to 156 more)	⊕◯◯◯
Pleurodesis failure (29)	Veryserious^d^	Serious^h^	No	No	Bias^i^	249/776 (32.1%)	340/771 (44.1%)	0.59 (0.48–0.73)	123 fewer per 1,000 (from 76 fewer to 166 fewer)	⊕◯◯◯
Disease progression (3)	Serious^g^	No^f^	No	Serious^e^	None	8/71 (11.3%)	13/74 (17.6%)	0.61 (0.24–1.58)	61 fewer per 1,000 (from 127 fewer to 76 more)	⊕⊕◯◯
Quality of life (8)	Very serious^d^	Serious^h^	No	No	None	180/226 (79.6%)	67/217 (30.9%)	9.93 (6.24–15.8)	507 more per 1,000 (from 427 more to 567 more)	⊕◯◯◯
Myelosuppression (5)	Serious^a^	No^f^	No	Serious^e^	None	3/138 (2.2%)	20/141 (14.2%)	0.19 (0.07–0.53)	111 fewer per 1,000 (from 61 fewer to 130 fewer)	⊕⊕◯◯
Neutropenia (9)	Very serious^d^	Serious^h^	No	No	None	5/210(2.4%)	54/204(26.5%)	0.11 (0.05–0.23)	227 fewer per 1,000 (from 188 fewer to 247 fewer)	⊕◯◯◯
Gastrointestinal reactions (15)	Serious^a^	No^f^	No	No	None^c^	25/389 (6.4%)	158/386 (40.9%)	0.12 (0.08–0.18)	333 fewer per 1,000 (from 298 fewer to 357 fewer)	⊕⊕⊕◯
Hepatotoxicity (6)	Serious^a^	No	No	Serious^e^	None	2/147(1.4%)	9/148 (6.1%)	0.22 (0.05–0.94)	47 fewer per 1,000 (from 3 fewer to 58 fewer)	⊕⊕◯◯
Nephrotoxicity (8)	Serious^a^	No	No	No	None	2/192 (1%)	18/193 (9.3%)	0.13 (0.04–0.46)	80 fewer per 1,000 (from 48 fewer to 89 fewer)	⊕⊕⊕◯
Thoracodynia (12)	Serious^a^	No	No	No	None	47/305 (15.4%)	34/305 (11.1%)	1.51 (0.94–2.44)	48 more per 1,000 (from 6 fewer to 123 more)	⊕⊕⊕◯
Fever (16)	Serious^a^	No^f^	No	No	None	149/423 (35.2%)	45/421 (10.7%)	6.66 (4.3–10.32)	337 more per 1,000 (from 233 more to 446 more)	⊕⊕⊕◯
**Staphylococcal enterotoxin C vs. interleukin-2 (IL-2)**										
Complete response (5)	Veryserious^d^	No	No	No	None	56/163 (34.4%)	37/155 (23.9%)	1.73 (1.03–2.88)	113 more per 1,000 (from 5 more to 236 more)	⊕⊕◯◯
Pleurodesis failure (5)	Veryserious^d^	No	No	No	None	37/163 (22.7%)	72/155 (46.5%)	0.32 (0.19–0.53)	247 fewer per 1,000 (from 150 fewer to 323 fewer)	⊕⊕◯◯
**Staphylococcal enterotoxin C and chemical agent**										
**Staphylococcal enterotoxin C plus cisplatin (DDP)**										
Complete response (77)	Serious^a^	No	No	No	None^b^	1,045/2,486 (42%)	531/2,333 (22.8%)	2.59 (2.28–2.95)	205 more per 1,000 (from 174 more to 237 more)	⊕⊕⊕◯
Pleurodesis failure (79)	Serious^a^	No	No	No	None^c^	362/2,539 (14.3%)	1,066/2,385 (44.7%)	0.2 (0.18–0.23)	308 fewer per 1,000 (from 290 fewer to 320 fewer)	⊕⊕⊕◯
Disease progression (13)	Veryserious^d^	No	No	No	None	19/434 (4.4%)	52/355 (14.6%)	0.27 (0.16–0.47)	102 fewer per 1,000 (from 72 fewer to 120 fewer)	⊕⊕◯◯
Quality of life (31)	Serious^a^	No	No	No	None	757/1,060 (71.4%)	394/1,007 (39.1%)	4.51 (3.7–5.5)	352 more per 1,000 (from 313 more to 388 more)	⊕⊕⊕◯
Myelosuppression (13)	Veryserious^d^	No	No	No	None	17/606 (2.8%)	35/592 (5.9%)	0.44 (0.24–0.8)	32 fewer per 1,000 (from 11 fewer to 44 fewer)	⊕⊕◯◯
Neutropenia (27)	Serious^a^	No	No	No	None	96/712 (13.5%)	191/673 (28.4%)	0.36 (0.27–0.48)	159 fewer per 1,000 (from 124 fewer to 187 fewer)	⊕⊕⊕◯
Thrombocytopenia (3)	Serious^a^	No	No	Serious^e^	None	7/53 (13.2%)	6/49 (12.2%)	0.97 (0.28–3.35)	3 fewer per 1,000 (from 85 fewer to 196 more)	⊕⊕◯◯
Gastrointestinal reactions (47)	Serious^a^	No^f^	No	No	None^c^	334/1,511 (22.1%)	555/1,461 (38%)	0.43 (0.36–0.51)	171 fewer per 1,000 (from 142 fewer to 199 fewer)	⊕⊕⊕◯
Hepatotoxicity (18)	Serious^a^	No	No	No	None	52/716 (7.3%)	41/681 (6%)	1.33 (0.85–2.09)	18 more per 1,000 (from 9 fewer to 58 more)	⊕⊕⊕◯
Nephrotoxicity (18)	Veryserious^d^	No	No	No	None	4/466 (0.9%)	16/431 (3.7%)	0.26 (0.1–0.72)	27 fewer per 1,000 (from 10 fewer to 33 fewer)	⊕⊕◯◯
Thoracodynia (32)	Serious^a^	No^f^	No	No	None	195/1,130 (17.3%)	167/1,102 (15.2%)	1.17 (0.93–1.47)	21 more per 1,000 (from 9 fewer to 56 more)	⊕⊕⊕◯
Fever (50)	Serious^a^	No^f^	No	No	None^b^	323/1,585 (20.4%)	141/1,527 (9.2%)	2.7 (2.16–3.36)	123 more per 1,000 (from 88 more to 162 more)	⊕⊕⊕◯
**Staphylococcal enterotoxin C plus carboplatin (CBP)**										
Complete response (3)	Serious^g^	No	No	Serious^e^	None	26/64 (40.6%)	10/55 (18.2%)	3.04 (1.3–7.12)	221 more per 1,000 (from 42 more to 431 more)	⊕⊕◯◯
Pleurodesis failure (3)	Serious^g^	No	No	Serious^e^	None	8/64(12.5%)	24/55(43.6%)	0.18 (0.07–0.46)	314 fewer per 1,000 (from 174 fewer to 385 fewer)	⊕⊕◯◯
**Staphylococcal enterotoxin C plus bleomycin (BLM)**										
Complete response (4)	Veryserious^d^	No	No	No	None	80/165 (48.5%)	39/151 (25.8%)	2.71 (1.68–4.36)	227 more per 1,000 (from 111 more to 345 more)	⊕⊕◯◯
Pleurodesis failure (4)	Veryserious^d^	No	No	No	None	22/165 (13.3%)	64/151 (42.4%)	0.2 (0.12–0.36)	296 fewer per 1,000 (from 214 fewer to 343 fewer)	⊕⊕◯◯
Disease progression (2)	Veryserious^d^	No	No	Serious^e^	None	3/75 (4%)	15/71 (21.1%)	0.16 (0.04–0.56)	170 fewer per 1,000 (from 81 fewer to 201 fewer)	⊕◯◯◯
**Staphylococcal enterotoxin C plus 5-fluorouracil (5-FU)**										
Complete response (2)	Veryserious^d^	No	No	Serious^e^	None	23/61 (37.7%)	9/61 (14.8%)	3.6 (1.48–8.75)	236 more per 1,000 (from 56 more to 455 more)	⊕◯◯◯
Pleurodesis failure (2)	Veryserious^d^	No	No	Serious^e^	None	10/61 (16.4%)	33/61 (54.1%)	0.17 (0.07–0.39)	374 fewer per 1,000 (from 226 fewer to 465 fewer)	⊕◯◯◯
**Staphylococcal enterotoxin C plus mitomycin-C (MMC)**										
Complete response (3)	Veryserious^d^	No	No	Serious^e^	None	21/76 (27.6%)	11/70 (15.7%)	2.06 (0.91–4.67)	120 more per 1,000 (from 12 fewer to 308 more)	⊕◯◯◯
Pleurodesis failure (3)	very serious^d^	No	No	Serious^e^	None	17/76 (22.4%)	40/70 (57.1%)	0.21 (0.1–0.44)	353 fewer per 1,000 (from 202 fewer to 454 fewer)	⊕◯◯◯
**Staphylococcal enterotoxin C plus etoposide (VP-16)**										
Complete response (2)	Serious^g^	No	No	Serious^e^	None	29/69 (42%)	19/67 (28.4%)	1.83 (0.9–3.75)	136 more per 1,000 (from 21 fewer to 314 more)	⊕⊕◯◯
Pleurodesis failure (2)	Serious^g^	No	No	Serious^e^	None	11/69 (15.9%)	35/67 (52.2%)	0.17 (0.08–0.39)	366 fewer per 1,000 (from 223 fewer to 442 fewer)	⊕⊕◯◯

*i: risk of bias; ii: inconsistency; iii: indirectness; iv: imprecision; v: publication bias; CI, confidence interval*.

a *Most trials had unclear risk, and some trials had high risk. If good robustness, we downgraded it by one grade*.

b *Publication bias was found in them; the result was overestimated; the result showed good robustness, and not be downgraded*.

c *Publication bias was found in them; the result was underestimated; the result showed good robustness, and not be downgraded*.

d *Most trials had unclear risk, and some trials had high risk; if sensitivity analysis results had poor robustness, we downgraded them by two grades*.

e *The number of patients in each result was <300, and we downgraded it with one grade*.

f *Heterogeneity was found in them; the result showed robustness, and not be downgraded*.

g *Most trials were unclear risk and no high risk, and we downgraded them with one grade*.

h *Heterogeneity was found in them; the result showed poor robustness; and we downgraded it with one grade*.

i *Publication bias was found in them; the result was underestimated; the result showed poor robustness; and we downgraded it with one grade*.

*Sixty-eight poor trials were involved in* SEC and chemical agent perfusion (19, 20, 37, 38, 41–45, 47–49, 51, 52, 56, 57, 61, 63–66, 69, 70, 72, 75, 76, 78–81, 84, 89, 91, 93, 94, 97–100, 102, 104, 107, 108, 110, 112–114, 116–118, 120, 123, 124, 126–129, 131, 133, 135–138, 141, 143–145, 148). *In SEC and DDP perfusion, the poor robustness was found for the OR of disease progression, myelosuppression, and nephrotoxicity. In SEC and BLM, MMC or 5-FU perfusion, the poor robustness was found in CR, failure, and disease progression*. And we downgraded their quality with two grades. While others had robustness, we downgraded the quality *with* one grade. *For* SEC and DDP perfusion, the statistical heterogeneity was found in gastrointestinal reaction, fever, and thoracodynia, which had robustness. A publication bias was found in CR, failure, gastrointestinal reactions, and fever, which had robustness, and we did not downgrade the quality. *For* SEC and DDP perfusion, the samples were lower than 300 subjects *in* thrombocytopenia. *For* SEC plus CBP, BLM, 5-FU, MMC or VP-16, the samples were lower than 300 subjects *in* CR and failure. So, we downgraded the quality one grade. *Finally*, we summarized a “moderate” for CR, failure, QOL, neutropenia, gastrointestinal reactions, hepatotoxicity, thoracodynia, and fever in SEC and DDP perfusion, and a “low” to “very low” for others (Table 7).

## Discussion

**Figure 6:**
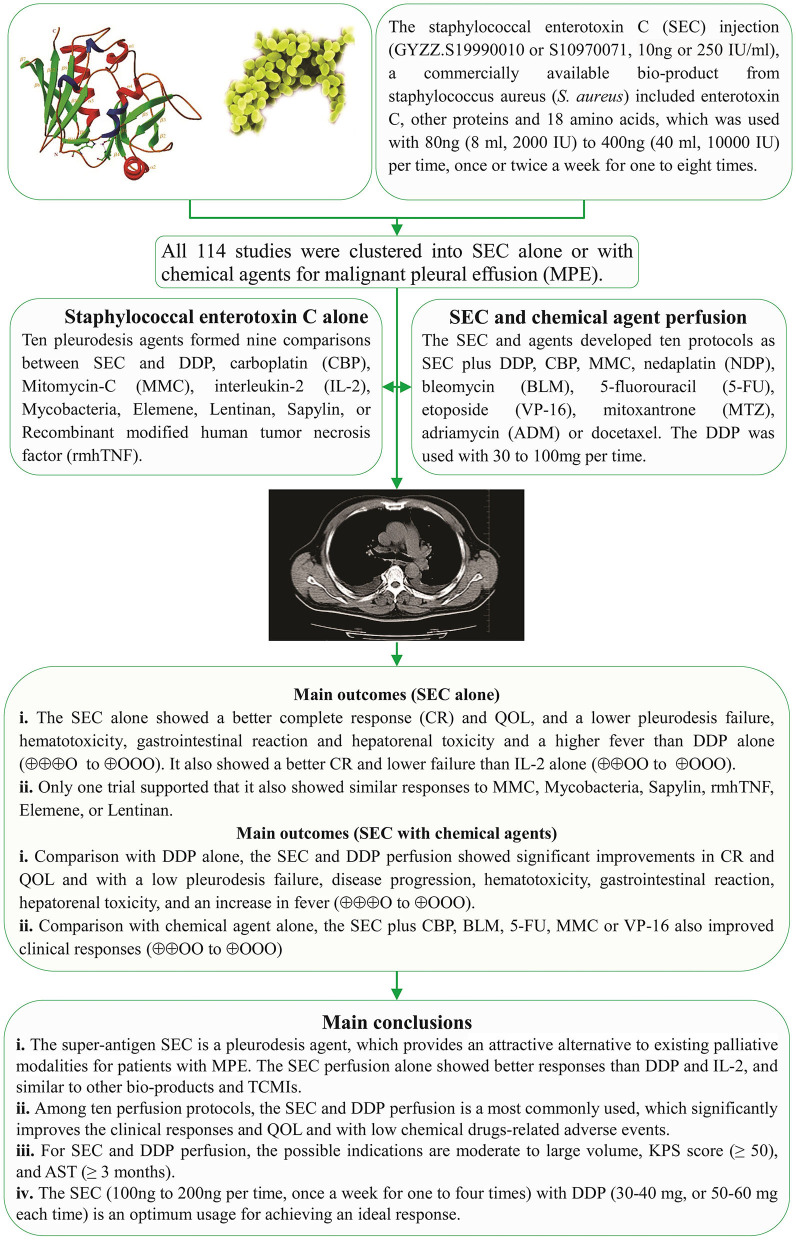
The evidence framework of SEC in MPE.

In China, the staphylococcal enterotoxin C (SEC), a super-antigen, has been used to control the MPE in the 1990s. To clarify the intrapleural perfusion protocols with SEC, determine their clinical effectiveness and safety, and reveal their indications and optimum usage, we integrated the previous *meta-analyses* (21, 22), supplemented 97 *studies* (37–47, 51–55, 57, 58, 60–62, 64–68, 70–72, 74–76, 78–88, 90–108, 110–120, 122–132, 134–140, 142, 143, 145–148), and implemented a clustered SR/meta-analysis. This new analysis found that the perfusion protocols were mainly SEC alone or plus chemical agents, *which showed significant clinical heterogeneity. So, we implemented topic clustering to obtain serial homogeneous protocols, and analyzed the data from each protocol using the meta-analysis or descriptive analysis*. In SEC perfusion alone, 10 pleurodesis agents formed nine comparisons. The results of meta-analysis determined that the SEC perfusion alone could show a better CR and QOL, a lower pleurodesis failure, hematotoxicity, gastrointestinal reactions *and* hepatorenal toxicity, and a higher fever than DDP alone. And it also showed better responses than IL-2 alone. But most results had “low to very low” quality. In addition, *limited trials showed that* it might obtain similar responses to bio-products as mycobacteria (88), sapylin (52) or rmhTNF (46), and TCMIs as elemene (60) or lentinan (58). *Many studies (**7*, *10*, *15**) had reported that treatment with staphylococcal super-antigenic products could result in massive cytokine production (IL-2, TNF* α, *and IFN* γ*), which plays a crucial role in the initiation and maintenance of pleural inflammation and pleural space obliteration. In addition, the bio-products from hemolytic streptococcialpha (**11*, *12**), corynobactum parvum (**13**), and streptococcus pyogenes (**14**) have been used in clinical studies to achieve pleurodesis and control fluid recurrence*. These results indicate that the super-antigen SEC is a pleurodesis agent, which induces pleural inflammation and achieves pleurodesis (Figure 6). This analysis further revealed that the SEC and 10 agents developed 30 perfusion protocols. The results determined that only the SEC and DDP perfusion could significantly improve the CR and QOL with a low failure, disease progression, hematotoxicity, gastrointestinal reactions, and hepatorenal toxicity, *but with* a high fever. Enough trials were included, and most results had “moderate” quality. Other protocols only included one to four trials, and the results had a “low to very low” quality. The related SR/meta-analyses reported that the biologic response modifiers, as Rh-Endostatin, lentinan or IL-2 with DDP perfusion (6, 9, 150) also showed a clinical benefit rate in MPE. *These results indicate that among 13* protocols, the SEC and DDP perfusion is *a commonly used protocol*, which shows a significant improvement in clinical responses with low ADRs (Figure 6).

Among 13 protocols, only the SEC and DDP perfusion included enough trials. The potential clinical heterogeneity still exists in baseline characteristics, interventions, and evaluation criteria between different trials. *Different from previous studies* (21, 22)*, we implemented a subgroup analysis to deal with the potential heterogeneity*. Further subgroup analysis revealed that the SEC and DDP perfusion could improve clinical responses in *both patients with* lung cancer and miscellaneous tumors. It also improved clinical responses in patients with moderate to large volume, KPS scores ≥40, ≥50, or ≥60, AST ≥2 or 3 months or primary treatment. However, only two to seven trials were included *for* treatment conditions such as KPS score (≥40), AST (≥2) or primary treatment. The univariable meta-regression revealed only a positive correlation between the tumor type and CR. *So, we adjusted the treatment conditions as* moderate to large volume, KPS scores ≥50 or ≥60, or AST ≥3 months, *and no restriction on the tumor type*. The relevant SR/meta-analyses (6, 9) reported that the Rh-endostatin or lentinan and DDP infusion could *also* improve the clinical responses under these conditions. So, we believe that bio-products perfusion may have similar treatment conditions, and a moderate to large fluid, KPS scores ≥50 or ≥60, and AST ≥3 months is a possible indication for SEC and DDP perfusion. The rational drug use is another key to affect clinical effectiveness and safety. Previous SR/meta-analyses (6, 9) reported that, in combination with Rh-endostatin/lentinan, the DDP perfusion was mainly used with 30–60 mg per time. This analysis found that the SEC was used with 80 ng (8 ml, 2,000 IU) to 400 ng (40 ml, 10,000 IU) per time, one time or two times a week and lasting one to four times, and the DDP was used with 30–100 mg per time. Fifty-eight *trials* reported the dosage of SEC as mainly 100 ng (10 ml, 2,500 IU) to 200 ng (20 ml, 5,000 IU), and 42 *trials* reported the DDP *as* 30–60 mg per time. The subgroup analysis revealed that, under these conditions, the SEC and DDP perfusion could improve the clinical responses, and the SEC with low-dosage obtained similar responses to high dosage. *The results indicate that the SEC combined with DDP can reduce the dosage of DDP. Finally*, the subgroup analysis found that drainage methods, evaluation criteria, or the publication year showed no impact on clinical responses. *However*, the univariable meta-regression and multivariate regression analysis only revealed a positive correlation between the pleurodesis failure and treatment frequency. Based on the principle of cost to effectiveness, we believe that the SEC (100–200 ng per time, one or two times a week and lasting one to four times) and DDP (30–40 mg or 50–60 mg each time) are possible optimal usage for achieving an ideal response (Figure 6).

In this study, we developed a clustered SR/meta-analysis, and some potential shortcomings were inevitable. *During the implementation, we followed the strategy of underestimating effectiveness and security. We tested the robustness of the results in an extreme condition, developed a modified model to summarize the evidence quality, and actively reduced the quality of all the results*. We only retrieved the Chinese and English databases, which existed potential retrieval bias. In 114 *studies*, most had unclear or high risk of methodological bias. Only some studies completely reported the baseline information, such as *fluid* volume, treatment history, functional status, and expected survival. Most selectively reported the ADRs and ignored the TRAEs, treatment-related death, overall mortality, *and hospital stay*. Two criteria were used to evaluate the clinical effectiveness and safety. In subgroup analysis, the univariate or multivariate regression analysis only found a sporadic correlation between clinical responses and tumor type or treatment frequency. These potential shortcomings might lead to an unfair evaluation for SEC in controlling MPE. In SEC perfusion alone, only one to five trials were included *for* other eight comparisons; most results had “low to very low” quality, and the network meta-analysis could not be performed. Therefore, the current evidence could not determine which does better between SEC and other bio-products or TCMIs. In SEC and chemical agents, only one trial supported that the SEC and DDP perfusion might improve the *overall survival. Two* to four trials for SEC plus CBP, BLM, 5-FU, MMC or VP-16, and the outcomes had “low to very low” quality. So, the current evidence could not demonstrate their clinical effectiveness, safety levels, indications, and optimal usage.

*This clustered SR/meta-analysis found that the perfusion protocols were mainly SEC alone or plus chemical agents, which showed obvious complexity and diversity*. The super-antigen SEC is a pleurodesis agent, which provides an attractive alternative to existing palliative modalities for patients with MPE. But the relationship between the SEC and others and which pleurodesis agent does better need to be further confirmed by new trials or network meta-analysis. Among 13 *SEC plus chemical agent* protocols, only the SEC and DDP perfusion could significantly improve the clinical responses with low ADRs. These findings provide a main perfusion protocol for controlling MPE, which have clinical significance for improving decision-making, preventing recurrence, and improving clinical response and a prognosis. *But only one trial reported that the SEC and DDP perfusion could improve overall survival. Most studies* selectively reported the ADRs, and ignored the TRAEs, which might lead to an unfair evaluation for its long-term survival and security. *Compared with previous meta-analyses* [*21, 22*]*, this analysis successfully implemented topic clustering to solve the complex problems, analyzed the data from each protocol using the meta-analysis or descriptive analysis, and provided serial systematic and complete pieces of evidence for treatment strategy using the TPs alone or plus chemical agents to control MPE, which will also provide theoretical and technical references for evaluating similar biological products*. In addition, the included trials reported that the dosage of SEC was 80–400 ng per time, and the DDP was 30–100 mg per time, which might be main reasons *for* irrational drug use and clinical decision-making failure. The subgroup analyses further found that, under the *conditions, as* moderate to large volume, KPS scores ≥50 or ≥60, or AST ≥3 months, the SEC (100–200 ng per time, one time or two times a week and lasting one to four times) and DDP (30–40 mg or 50–60 mg each time) are possible optimal usage for achieving an ideal response. All these provide a possible indication and optimal usage for SEC and DDP perfusion. *Compared with traditional analysis (**21*, *22**), this analysis performed a subgroup analysis to analyze the potential heterogeneity and found serial indirect results, which further provide an indication and optimal usage for an optimal control strategy, which is of clinical significance to formulate the optimal perfusion protocol, reject the unreasonable, and control medical expenses*. But these conclusions came from indirect evidence. So, these conclusions need be further confirmed by using direct evidence.

## Conclusion

Current pieces of evidence indicate that the super-antigen SEC is a pleurodesis agent, which provides an attractive alternative to existing palliative modalities for patients with MPE. Among 13 perfusion protocols, the SEC and DDP perfusion is a most commonly used, which shows a significant improvement in clinical responses and QOL with low chemical drugs-related adverse events. For this protocol, the possible indications are moderate to large volume, KPS score (≥50), and AST (≥3 months). The SEC (100–200 ng per time, one time a week for one to four times) with DDP (30–40 mg, or 50–60 mg each time) is optimum usage for achieving an ideal response. Finally, *we hope that this analysis provides a* valuable evidence framework for an optimal control strategy of using SEC in MPE.

## Data Availability Statement

The original contributions presented in the study are included in the article/Supplementary Material, further inquiries can be directed to the corresponding authors.

## Author Contributions

Conception and design by ZX, XX, and LZ. Development of methodology by ZX, X-FC, and C-QW. Literature search and statistical analysis by HJ and C-QW. Article selection and assessment of methodological bias risk by HJ and X-MY. Data extraction by JX and JH. GRADE assessment by C-QW and X-FC. Preparing the manuscript draft by HJ, X-MY, and ZX. Review and revision of the manuscript by KC, J-HF, and LZ. Study supervision by ZX and XX. All authors contributed to the article and approved the submitted version.

## Funding

This work was funded by a High-Level Innovative Talent Program in Guizhou (No. fzc 120171001), and special funds for Science and Technology Research Into Traditional Chinese and National Medicine in Guizhou (No. QZYY 2017-084).

## Conflict of Interest

The authors declare that the research was conducted in the absence of any commercial or financial relationships that could be construed as a potential conflict of interest.

## Publisher's Note

All claims expressed in this article are solely those of the authors and do not necessarily represent those of their affiliated organizations, or those of the publisher, the editors and the reviewers. Any product that may be evaluated in this article, or claim that may be made by its manufacturer, is not guaranteed or endorsed by the publisher.

## Abbreviations

5-FU: 5-fluorouracil
ADM: adriamycin
ADRs: adverse drug reactions
AST: anticipated survival time
BLM: bleomycin
CBM: China biological medicine database
CBP: carboplatin
CENTRAL: Cochrane central register of controlled trials
CI: confidence intervals
CNKI: China National Knowledge Infrastructure Database
Coef: coefficient
CR: complete response
CTCAE: common terminology criteria for adverse events
DDP: cisplatin
DP: disease progression
FEM: fixed-effects model
GRADE approach: grades of recommendation assessment, development, and evaluation approach
HR: hazard ratio
IL-2: interleukin 2
IFN γ: interferon gamma
IPCs: indwelling pleural catheters
IU: international unit
KPS: Karnofsky performance status
LBP: lobaplatin
MMC: mitomycin-C
MPE: malignant pleural effusion
MTZ: mitoxantrone
MU: million units
NDP: nedaplatin
NMPA: National Medical Products Administration
NR: no response
NSCLC: non-small cell lung cancer
OR: odds ratios
OS: overall survival
PFS: progression-free survival
PR: partial response
PRISMA guidelines: preferred reporting items for systematic reviews and meta-analyses guidelines
PT: primary treatment
QOL: quality of life
RCTs: randomized controlled trials
rmhTNF: recombinant modified human tumor necrosis factor
RT: retreatment
REM: random-effects model
SM: statistical method
SEC: staphylococcal enterotoxin C
SD: stable disease
SR: systematic review
TCMIs: traditional Chinese medicine injections
TNF α: tumor necrosis factor α
TH: treatment history
TRAEs: thoracentesis-related adverse events
VIP: Chinese Scientific Journals Full-Text Database
VP-16: etoposide
WHO: World Health Organization.
