# Supplementary material for: Intrapleural Perfusion With Staphylococcal Enterotoxin C for Malignant Pleural Effusion: A Clustered Systematic Review and Meta-Analysis
Source: Front Med (Lausanne). 2022 Apr 25;9:816973. doi: 10.3389/fmed.2022.816973 (PMC9081816; doi:10.3389/fmed.2022.816973)
Supplement: Supplementary file 1 [file Data_Sheet_1.PDF]

Appendix 1. Evidence quality summary model

a Most trials were unclear risk and with high risk, the result showed good robustness, and the evidence was rated down by only one level;

b Most trials were unclear risk and with high risk, the result showed poor robustness, and the evidence was rated down by two level;

c Heterogeneity was found in them, the results showed good robustness, and not rated down;

d Heterogeneity was found in them, the result showed poor robustness, and the evidence was rated down by one level;

e The sample size for indicator was fewer than 300 cases, and the evidence was rated down by one level;

f Publication bias was found among them, excluded the under- and over-estimated studies, the results showed good robustness, and not be downgraded;

g Publication bias was found among them, excluded the under- and over-estimated studies, the result showed poor robustness, and the evidence was rated down by one level;
