# Supplementary material for: Intrapleural Perfusion With Staphylococcal Enterotoxin C for Malignant Pleural Effusion: A Clustered Systematic Review and Meta-Analysis
Source: Front Med (Lausanne). 2022 Apr 25;9:816973. doi: 10.3389/fmed.2022.816973 (PMC9081816; doi:10.3389/fmed.2022.816973)
Supplement: Supplementary file 2 [file Data_Sheet_2.PDF]

Appendix 2 Retrieval results

Table S1. Identified 2 records from Pubmed

Search	Query	Items found
**#26**	**Search: (((((((((((((("enterotoxin C, staphylococcal" [Supplementary Concept]) OR (Staph enterotoxin C)) OR (Staphjlo Toxoid Injection)) OR (Staphylococcal Enterotoxin C Injection)) OR (Staph enterotoxin C2)) OR (SEC2 toxin)) OR (toxin SEC2)) OR (Staph enterotoxin C3)) OR (Staph enterotoxin C1)) OR (SEC1 toxin)) OR ("Highly"[All Fields] AND "staphy"[All Fields])) OR (gao, jusheng)) OR (gao jusheng)) OR (Jinpusu)) AND (((((((("Pleural Effusion"[Mesh]) OR ("Pleural Effusions")) OR ("Pleural Effusion")) OR ("hydrothorax")) OR ("MPE")) OR ("MPEs")) OR ("carcinomatous pleurisy")) OR ("cancerous pleurisy")) OR ("Malignant pleurisy"))**	**2**
#25	Search: **((((((("Pleural Effusion"[Mesh]) OR ("Pleural Effusions")) OR ("Pleural Effusion")) OR ("hydrothorax")) OR ("MPE")) OR ("MPEs")) OR ("carcinomatous pleurisy")) OR ("cancerous pleurisy")) OR ("Malignant pleurisy")**	37,287
#24	Search: **"Malignant pleurisy"**	76
#23	Search: **"cancerous pleurisy"**	19
#22	Search: **"carcinomatous pleurisy"**	61
#21	Search: **"MPEs"**	313
#20	Search: **"MPE"**	2,500
#19	Search: **"hydrothorax"**	2,254
#18	Search: **"Pleural Effusion"**	31,418
#17	Search: **"Pleural Effusions"**	7,765
#16	Search: **"Pleural Effusion"[Mesh]**	20,307
#15	Search: **((((((((((((("enterotoxin C, staphylococcal" [Supplementary Concept]) OR (Staph enterotoxin C)) OR (Staphjlo Toxoid Injection)) OR (Staphylococcal Enterotoxin C Injection)) OR (Staph enterotoxin C2)) OR (SEC2 toxin)) OR (toxin SEC2)) OR (Staph enterotoxin C3)) OR (Staph enterotoxin C1)) OR (SEC1 toxin)) OR ("Highly"[All Fields] AND "staphy"[All Fields])) OR (gao, jusheng)) OR (gao jusheng)) OR (Jinpusu)**	2,440
#14	Search: **Jinpusu**	0
#13	Search: **gao jusheng**	19
#12	Search: **gao, jusheng**	19
#11	Search: **Highly agglutinitine staphy Ilclclin**	0
#10	Search: **SEC1 toxin**	293
#9	Search: **Staph enterotoxin C1**	293
#8	Search: **Staph enterotoxin C3**	293
#7	Search: **toxin SEC2**	293
#6	Search: **SEC2 toxin**	293

#5	Search: **Staph enterotoxin C2**	293
#4	Search: **Staphylococcal Enterotoxin C Injection**	19
#3	Search: **Staphjlo Toxoid Injection**	2,125
#2	Search: **Staph enterotoxin C**	293
#1	Search: **"enterotoxin C, staphylococcal" [Supplementary Concept]**	291

Table S2. Identified 3 records from Embase

Search	Query	Items found
**#25**	**#15 AND #24**	**3**
**#24**	#16 OR #17 OR #18 OR #19 OR #20 OR #21 OR #22 OR #23	55,116
**#23**	**malignant** AND **pleurisy**	1,568
**#22**	**cancerous** AND **pleurisy**	54
**#21**	**carcinomatous** AND **pleurisy**	272
**#20**	**mpes**	457
**#19**	**mpe**	3,807
**#18**	**hydrothorax**	5,182
**#17**	**pleural** AND **effusions**	15,579
**#16**	**pleural** AND **effusion**	46,292
**#15**	#1 OR #2 OR #3 OR #4 OR #5 OR #6 OR #7 OR #8 OR #9 OR #10 OR #11 OR #12 OR #13 OR #14	2,610
**#14**	**jinpusu**	0
**#13**	**gao** AND **jusheng**	32
**#12**	**gao,** AND **jusheng**	32
**#11**	**highly** AND **agglutinitine** AND **staphy** AND **ilclclin**	0
**#10**	**sec1** AND **toxin**	41
**#9**	**staph** AND **enterotoxin** AND **c1**	1
**#8**	**staph** AND **enterotoxin** AND **c3**	0
**#7**	**toxin** AND **sec2**	25
**#6**	**sec2** AND **toxin**	25
**#5**	**staph** AND **enterotoxin** AND **c2**	0
**#4**	**staphylococcal** AND **enterotoxin** AND **c** AND **injection**	120
**#3**	**staphjlo** AND **toxoid** AND **injection**	0
**#2**	**staph** AND **enterotoxin** AND **c**	
**#1**	**enterotoxin c, staphylococcal'** OR ((**'enterotoxin'**/exp OR **enterotoxin**) AND **c,** AND **staphylococcal**)	2,527

Table S3. Identified 343 records from China Biological Medicine Database

Search	Query	Items found
#24	(#23) AND (#14)	343
#23	(#22) OR (#21) OR (#20) OR (#19) OR (#18) OR (#17) OR (#16) OR (#15)	34,507
#22	"胸膜腔积水"[全部字段:智能]	0
#21	"胸腔积液"[全部字段:智能]	30,621
#20	"胸水"[全部字段:智能]	9,416
#19	"胸腔积水"[全部字段:智能]	42
#18	"MPE"[全部字段:智能]	680
#17	"癌性胸膜炎"[全部字段:智能]	70
#16	"恶性胸膜炎"[全部字段:智能]	31
#15	"胸膜腔积液"[全部字段:智能]	295
#14	(#13) OR (#12) OR (#11) OR (#10) OR (#9) OR (#8) OR (#7) OR (#6) OR (#5) OR (#4) OR (#3) OR (#2) OR (#1)	2,122
#13	"肠毒素 C"[全部字段:智能]	73
#12	"SEC"[全部字段:智能]	1,385
#11	"思复胜"[全部字段:智能]	2
#10	"葡萄球菌肠毒素 C"[全部字段:智能]	49
#9	"葡萄球菌 C 型肠毒素"[全部字段:智能]	5
#8	"金葡素注射液"[全部字段:智能]	31
#7	"金葡素"[全部字段:智能]	382
#6	"高聚金葡素"[全部字段:智能]	295
#5	"高聚生"[全部字段:智能]	186
#4	"恩格菲"[全部字段:智能]	52
#3	"芬倍舒"[全部字段:智能]	10
#2	"金葡液"[全部字段:智能]	128
#1	"金葡菌毒素注射液"[全部字段:智能]	1

Table S4.The included trials from the meta-analyses

Authors	Title	Included trials	Trials included in this study	Journals
Liu, J. 2001[1]	The efficacy of highly agglutinative staphylococcin combined with chemotherapeutic drugs in the treatment of malignant pleural and ascites: A Meta analysis.	12 trials	3 trials [2-4]	Shenyang Army Med **14**(6): 556-557.
Liu, Y. 2016[5]	Meta -analysis of the treatment efficacy of staphylococcin plus cisplatin versus cisplatin monotherapy in malignant pleural effusion and malignant intraperitoneal effusion	16 trials	14 trials[6-19]	J Mod Oncol **24**(21): 3479-3484.

In all, we included 17 trials [2-4 6-19]after evaluating previous two meta analyses

1. Liu J, Zhao H, Liu X. The efficacy of highly agglutinative staphylococcin combined with chemotherapeutic drugs in the treatment of malignant pleural and ascites: A meta analysis. *Shenyang Army Med* 2001;14(6):556-57.
2. Li S, Wu Y, Wang X. Observation on the clinical efficacy of combined intrapleural injection of high-aggregating staphylococcus aureus and cisplatin in the treatment of malignant pleural effusion (analysis of 40 cases). *J Pract Oncol* 1997;12(4):171-72.
3. Zhang Y. Observation on the effect of high-aggregating staphylococcus aureus combined with mitoxantrone in the treatment of lung pleural effusion. *West China J Pharmaceut Sci* 1999;14(3):200-00. doi: 10.3969/j.issn.1006-0103.1999.03.025
4. Fu J. Treatment of 55 cases of cancerous pleural effusion with highly agglutinative staphylococcin. *China Pharmaceuticals* 2000;9(10):40-41. doi: 10.3969/j.issn.1006-4931.2000.10.033
5. Liu Y, Zhang X, Huang L. Meta -analysis of the treatment efficacy of staphylococcin plus cisplatin versus cisplatin monotherapy in malignant pleural effusion and malignant intraperitoneal effusion. *J Mod Oncol* 2016;24(21):3479-84. doi: 10.3969/j.issn.1672-4992.2016.21.037
6. Qiu Y, Han G, Ma S. Observation of the curative effect of high-agglomerated staphylococcus and cisplatin on malignant pleural fluid. *Sichuan J Cancer Contr* 1999;12(3):28-30.
7. Lang F, Zhao M, Zhao M, et al. Observtion of the result of highly agglutinative staphylococcin and cisplatin in treating of malignant pleural feeusion. *China J Cancer Prev Treat* 2001;8(z1):256-58. doi: 10.3969/j.issn.1673-5269.2001.z1.073
8. Xu Q, Luo K, Qin H. Observation on the curative effect of low-dose cisplatin combined with high-aggregation staphylococcus aureus in the treatment of 62 cases of malignant pleural effusion. *Chongqing Med* 2003;32(12):1659-59,61. doi: 10.3969/j.issn.1671-8348.2003.12.088
9. Chen J, Cheng X. Clinical observation on treating malignant pleural effussion with both gaojusheng and cisplatin. *Jiangxi J TCM* 2005;36(8):19-20. doi: 10.3969/j.issn.0411-9584.2005.08.012
10. Zhao Z, Li K, Lu W, et al. Clinical observation of Gaojusheng combined with cisplatin in the treatment of malignant pleural effusion. *Pract Clin J ITCWM* 2006;6(6):19-20. doi: 10.3969/j.issn.1671-4040.2006.06.013
11. Wang H, Wang Y, Su L, et al. Clinical observation on the treatment of 84 cases of pleural effusion caused by lung cancer. *Chongqing Med* 2009;38(01):66-67. doi: 10.3969/j.issn.1671-8348.2009.01.030
12. Cheng J, An Y, Zhang X, et al. Clinical observation on treatment of malignant hydrothorax with locally administered highly agglutinative staphylococcin and cisplatin. *China Pharm* 2010;21(12):1130-31.
13. Qu Y, Liu T, Cao y, et al. The therapeutic effects of highly agglutinative staphylococcin combined with cisplatin in the treatment of patients with malignant pleural effusion. *J Basic Clin Oncol* 2010;23(5):413-14. doi: 10.3969/j.issn.1673-5412.2010.05.019
14. Xu J. Efficacy of high-aggregation staphylococcus aureus combined with cisplatin in the treatment of pleural effusion caused by lung cancer. *Strait Pharm J* 2012;24(10):191-92. doi: 10.3969/j.issn.1006-3765.2012.10.102
15. Du Y. Clinical observation on the treatment of pleural effusion caused by lung cancer with

high aggregate staphylococcus and cisplatin. *Chin J Clin Oncol Rehab* 2013;20(09):987-89. doi: 10.13455/j.cnki.cjcor.2013.09.033

16. Li W. Efficacy of high-aggregation staphylococcus aureus combined with cisplatin in the treatment of pleural effusion caused by lung cancer. *China Health Care Nutr* 2013;23(9):4857-58. doi: 10.3969/j.issn.1004-7484(s).2013.09.077
17. Yao X. Therapeutic effect of cisplatin combined with Staphylococcin on malignant pleural effusion. *Chin Pract J Rural Doct* 2014;21(18):51-52. doi: 10.3969/j.issn.1672-7185.2014.18.031
18. Zhang J. Observation and nursing of intrapleural injection of different drugs in treating malignant pleural effusion. *Nurs Pract Res* 2014;11(10):65-66. doi: 10.3969/j.issn.1672-9676.2014.10.035
19. Zhao Y. Comparative observation on the efficacy of intrapleural injection of different drugs in the treatment of lung cancer pleural effusion. *Yiyao Qianyan* 2014;4(12):208-08. doi: 10.3969/j.issn.2095-1752.2014.12.227

List of excluded studies

i) Study about patients with systemic chemotherapy (35 studies)

[1] X. Chen, Endovascular treatment of 26 cases of malignant pleural effusion with highly agglutinated staphylococcin, JOURNAL OF SNAKE 11(2) (1999).
[2] L. Hua, Q. Qi, Z. Song, W. Yao, Therapeutical effect of Fanbrecin combined with Cisplatin on malignant pleural effusion, JOURNAL OF XINXIANG MEDICAL COLLEGE 17(4) (2000) 284-285.
[3] J. Jin, F. Wang, W. Lu, Clinical report of 48 cases of malignant pleural effusion treated with highly agglutinative staphylococcin (highly agglutinative staphylococcin) combined with cisplatin, CHINESE JOURNAL OF CLINICAL ONCOLOGY 27(12) (2000) 928.
[4] C. Li, J. Gao, X. Cao, Q. Li, Clinical Efficacy of Highly Agglutinative Staphylococci plus Cisplatin in the Treatment of Malignant Pleural Effusions, CANCER RESEARCH ON PREVENTION AND TREATMENT 27(3) (2000) 226-227.
[5] H. Li, X. Bao, Y. Li, Therapeutic effect of highly agglutinative staphylococin combined cisplatin on malignant pleural effusion, JOURNAL OF CLINICAL INTERNAL MEDICINE 17(1) (2000) 59-60.
[6] H. Li, W. Yang, Y. Jin, Y. Li, X. Bao, The Effect of Combination of Highly Agglutinative Staphylococcin and Carboplatin in Treatment of Malignant Thoracic Effusion, JOURNAL OF INTERNAL INTENSIVE MEDICINE 6(3) (2000) 126， 129.
[7] L. Wei, Therapeutic effect of intrapleural injection of cisplatin and Highly Agglutinative Staphylococcin on Cancerous Pleural Effusion, HUBEI JOURNAL OF PREVENTIVE MEDICINE (6) (2000) 52.
[8] F. Zhao, Y. Cui, D. Jiang, The Influence of Fanbrecin on The Levels of CEA, CT, CY21-1 in The Cancerous Pleural Effusion, LABELED IMMUNOASSAYS AND CLINICAL MEDICINE 7(3) (2000) 124-127.
[9] D. Sun, H. Chen, X. Yao, Clinical observation and nursing of 36 cases of malignant pleural effusion treated with staphylococcin and chemotherapy, Chinese Journal of Medical Writing (10) (2001).
[10] H. Fang, A. Gao, H. Wang, Clinical observation of intrapleural injection combined with systemic chemotherapy in the treatment of malignant pleural effusion, CHINA MEDICINE BULLETIN 2(4) (2002) 40-41.
[11] H. Wu, F. Pan, Therapeutic effect of gaoyusheng on 28 cases of lung cancer complicated with pleural effusion, Chinese general medicine 003(4) (2002) 333-334.
[12] W. Zheng, J. Zhang, Therapeutic effect of highly agglutinated staphylococcin combined with low-dose bleomycin in the treatment of malignant pleural effusion, CHINESE JOURNAL OF CLINICAL ONCOLOGY AND REHABILITATION 9(3) (2002) 66-67.
[13] J. Gao, X. Ma, Effect of intrapleural injection of highly agglutinated Staphylococcin on pleural effusion of lung cancer, HERALD OF MEDICINE 22(9) (2003) 631-632.
[14] Y. Li, X. Guan, Comparison of intrapleural injection of highly agglutinated staphylococcin and cisplatin in the treatment of lung cancer pleural effusion and systemic chemotherapy in the treatment of lung cancer pleural effusion, Chinese Research Journal of Practical Medicine 002(001) (2003).
[15] H. Qian, Treatment of 18 cases of malignant pleural effusion with integrated traditional

Chinese and Western Medicine, HENAN TRADITIONAL CHINESE MEDICINE 23(8) (2003) 53-54.
[16] X. Wang, G. Cao, Y. Sun, Clinical observation on malignant pleural effusion treated by intrapleural perfusion of staphylococcal entero toxin combined with hydroxycamptothecin, CHINESE JOURNAL OF DIFFICULT AND COMPLICATED CASES 3(3) (2004) 137-139.
[17] H. Zhu, Intrathoracic administration of highly agglutinated staphylococcin combined with systemic chemotherapy in the treatment of Cancerous Pleural Effusion, Chinese Journal of modern practical medicine 003(3) (2004).
[18] W. Jia, H. Li, Clinical Effects of Injecting Highly Agglutinated Staphylococcin on Pleural Effusion Induced by Lung Cancer, PRACTICAL CLINICAL MEDICINE 6(7) (2005) 42-43.
[19] X. Tang, T. Jiang, Y. Wu, Effect of High agglomerative staphylococoin on malignant pleural effusion, JOURNAL OF CHONGQING MEDICAL UNIVERSITY 31(1) (2006) 124-125,128.
[20] X. Zhang, W. Zhang, C. Shi, Clinical effect of intrapleural cavity perfusion of carboplatin combined with staphylococcal enterotoxin C treating malignant pleural effusion, CHINA MEDICAL HERALD 4(35) (2007) 21-23.
[21] S. He, C. Ma, H. Liu, Effect of central venous catheter drainage and perfusion of high polymer in the treatment of malignant pleural effusion, CHINA MODERN DOCTOR 46(26) (2008) 35-36.
[22] J. Liu, Y. Li, Z. Dong, X. He, Clinical observation of continuous drainage intrathoracic injection of cisplatin combined with high polymer in the treatment of malignant pleural effusion, JOURNAL OF PRACTICAL DIAGNOSIS AND THERAPY 22(6) (2008) 460-461.
[23] Y. Zhou, S. Gong, J. Ni, Study of highly agglutinntive Staphyloccocin or Ientinan combined with chemotherapy on malignant pleural effusion of non-small cell lung cancer, Modern Journal of Integrated Traditional Chinese and Western Medicine 17(36) (2008) 5561-5562,5713.
[24] L. Zhang, X. Li, J. Gu, F. Ma, L.Z. Zheng, Intracavitary infusion treatment of malignant effusion in body cavity with highly agglutinated staphytococcin plus cisplatin, THE JOURNAL OF PRACTICAL MEDICINE 25(22) (2009) 3856-3858.
[25] Q. Zhu, G. Sun, Comparison of pingyangmycin and highly agglutinative staphylococcin pleurodesis for malignant pleural effusion, JOURNAL OF CLINICAL PULMONARY MEDICINE 14(7) (2009) 914-915.
[26] J. Ke, H. Ge, Effect of HAS on the growth of NSCLC cell line A549 and malignant pleural effusion, JOURNAL OF NANTONG UNIVERSITY(MEDICAL SCIENCES) 31(4) (2011) 248-250,253,封 2.
[27] W. Li, J. Wang, Effects of Intrapleural Injection of Urokinase on the Therpaeutic Efficacy of Staphylococcus Aureus Enterotoxin in the Treatment of Multilocular Encapsulated Malignant Pleural Effusion, China Pharmacy 22(36) (2011) 3404-3406.
[28] G. Lu, L. Yu, Y. Zhang, Comparison of Mannatide and Highly Agglutinative Staphylococcin in treatment of Malignant Pleural Effusion, JOURNAL OF CLINICAL PULMONARY MEDICINE 17(10) (2012) 1846-1847.
[29] X. Yuan, G. Zhu, B. Liu, Intrathoracic injection combined with systemic chemotherapy in the treatment of Cancerous Pleural Effusion, Xinli Yesheng (8) (2012) 32-33.
[30] J. Zhang, Y. Wang, A. Mao, Y. Zhou, Studies of the significance of intercostal arterial infusion in multi-interventional therapy for malignant pleural effusion with pulmonary carcinoma, SURGICAL RESEARCH AND NEW TECHNIQUE 1(1) (2012) 56-60.

[31] G. Weng, Clinical study of highly agglutinative staphylococcin combining with bleomycin by intrapleural injection in the treatment of malignant pleural effusion in 82 cases, China Modern Medicine 20(29) (2013) 82-83.
[32] T. Zhang, X. Gao, K. Zhu, Therapeutic effect of abdominal drainage catheter combined with gefitinib in the control of Cancerous Pleural Effusion, Chinese and Foreign Medical Research (11) (2013) 11.
[33] J. Cui, X. Zhao, J. Chen, Therapeutic effect of intrapleural injection of mitomycin and staphylococcin in the treatment of malignant pleural effusion, Journal Of China Prescription Drug (12) (2014) 52-52.
[34] G. Lu, L. Yu, Y. Zhang, Y. Xia, W. Hu, Comparison of Lentinan and Highly Agglutinative Staphylococcin in treatment of Malignant Pleural Effusion, Progress in Modern Biomedicine 14(13) (2014) 2497-2499.
[35] Y. Zhang, Clinical comparison of intrapleural injection of different drugs in the treatment of lung cancer pleural effusion, Strait Pharmaceutical Journal 31(1) (2019) 216-217.

ii) Study about SEC plus other BRMs (nine studies)

[1] L. Chen, W. Li, Z. Song, B. Liang, Improved percutaneous placement catheters drainage of thoracocentesis accompanying with intracavitary chemotherapy for the treatment of malignant pleural effusion, JOURNAL OF MODERN ONCOLOGY 13(2) (2005) 235-236.
[2] G. Ge, F. Men, J. Wu, Z. Hu, Clinical observation of biological agents combined with intrathoracic injection in the treatment of malignant pleural effusion, CHINESE CLINICAL ONCOLOGY (2) (1998) 60-61.
[3] X. Gu, P. Chen, C. Zhang, The clinical observation on curative effect of intrathoracic indwelling central venous catheter and high poly gold pt element,interleukin-II plus cisplatin joint perfusion chemotherapy applied in contoling of malignant pleural effusion after breast cancer operation, Chinese Community Doctors (13) (2015) 61-62.
[4] H. Huang, 40 cases of malignant pleural effusion treated with endo and staphylococcin, Chinese Journal of Medical Device (16) (2015) 77-78.
[5] F. Long, X. Pan, H. Qing, D. Li, B. Peng, W. Zhang, L. Wu, Treatment of Cancerous Pleural Effusion with highly agglutinated staphylococcin and thrombin, CHINESE JOURNAL OF CLINICAL ONCOLOGY AND REHABILITATION 9(2) (2002) 5-5.
[6] H. Song, Nursing care of elderly patients with malignant pleural effusion treated by central venous catheter drainage, JOURNAL OF QILU NURSING 11(1) (2005) 56-57.
[7] A. Wang, Q. Wang, Analysis of 41 cases of malignant pleural effusion treated with percutaneous intrathoracic tube combined with drugs, MEDICAL INNOVATION OF CHINA 6(16) (2009) 140.
[8] C. Zhang, P. Fan, W. Zheng, Z. Zhang, M. Tang, Observation of curative effect of Gao Ju Sheng, Yin Kang combined with cisplatin on postoperative pleural effusion after breast cancer operation, JOURNAL OF CHINESE PHYSICIAN 12(4) (2010) 555-557.
[9] Y. Zhu, D. Li, Z. Wang, M. Tao, F. Xiong, Clinical observation of cytokine-induced killer cells in treatment of cisplatin-resistant malignant pleural effusion, Journal of Nantong University (Medical Sciences) (6) (2013) 487-490.

iii) Study about traditional Chinese medicine injections (TCMIs)/hyperthermia (13 studies)

[1] Y. Chen, J.q. Lin, 28 cases of malignant pleural effusion treated with integrated traditional Chinese and Western Medicine, Fujian Medical Journal (06) (1998) 103.
[2] Y. Gao, T. Wen, L. Deng, Cinobufagin injection, Staphylococcin Aureus combined with cisplatin in the treatment of 89 elderly patients with lung cancer and pleural effusion, CONTEMPORARY MEDICINE 18(24) (2012) 139-140.
[3] G. Li, H. Xue, Combination of Thermotherapy and Intrathoracic Infusion with En-Ge-Fei for the Treatment of Advanced Malignant Pleural Effusion, BULLETIN OF CHINESE CANCER 14(2) (2005) 139-140.
[4] X. Ni, H. Feng, Clinical study of Xiaoyin Mixture combined with Biological Response Modifiers in Elderly Patients with Malignant Pleural Effusion, Journal of Emergency in Traditional Chinese Medicine 23(1) (2014) 56-57,72.
[5] J. Wang, X. He, Experience of elemene combined with highly agglutinated staphylococcin in the treatment of malignant pleural effusion, MODERN JOURNAL OF INTEGRATED TRADITIONAL CHINESE AND WESTERN MEDICINE 11(9) (2002) 828.
[6] J. Wang, A. Zhu, C. Zhai, Clinical observation on 30 cases of malignant pleural effusion treated with integrated traditional Chinese and Western Medicine, JIANGSU JOURNAL OF TRADITIONAL CHINESE MEDICINE 42(11) (2010) 32-33.
[7] F. Xu, Curative effect observation and nursing care of elemene emulsion and high poly staphylococcin in the treatment of malignant pleural effusion, ZHEJIANG CLINICAL MEDICAL JOURNAL 5(12) (2003) 956-957.
[8] W. Ye, Study of intraplearal infusion of staphylococcus enterotoxin combined with radiofrequency hyperthermia in the treatment of malignant pleural effusion, Journal of Modern Oncology (6) (2014) 1443-1445.
[9] Q. Yu, H. Wu, Observation and nursing care of patients with malignant pleural effusion treated with engf plus cisplatin pleural perfusion chemotherapy combined with hyperthermia, CHINESE JOURNAL OF PRACTICAL NURSING 27(22) (2011) 45-46.
[10] L. Zhang, Z. Reng, Y. Xue, X. Yuan, Clinical Analysis of Jijiao Lihuang Wan as Adjunctive Therapy for Lung Cancer Accompanied by Pleural Effusion, Chinese Journal of Experimental Traditional Medical Formulae 22(3) (2016) 174-178.
[11] J. Zheng, F. Ru, J. Zhang, Q. Ye, Q. Tong, S. Xi, W. Gu, Immunochemotherapy Combined with Hyperthermia Treatment for Malignant Pleural Effusion, JOURNAL OF CLINICAL RADIOLOGY 23(12) (2004) 1080-1084.
[12] L. Zhou, X. Fu, J. Hong, L. Wu, G. Cheng, THE CLINICAL STUDY ON THE PLAN OF ELEMENE EMULSION WITH STAPHYLOCOCCAL ENTEROTOXIN TO TREAT MALIGNANT EFFUSION, CANCER RESEARCH ON PREVENTION AND TREATMENT (3) (1998) 218-219.
[13] A. Zhu, Clinical Observation of Integrated Traditional and Western Medicine in the Treatment of Malignancy Pleural Effusions, Liaoning Journal of Traditional Chinese Medicine 35(12) (2008).

iv) Other subjects (SEC used in both groups and others)

[1] H. Jin, J. Liu, Clinical Effect of Highly Agglutinative Staphylococcin plus Cisplatin in Treatment of Malignant Pleural Effusion, CHINESE JOURNAL OF CLINICAL ONCOLOGY 29(11) (2002) 799-801.

[2] C. Sheng, S. Huang, X. Huang, Z. Wu, Z. Huang, Treatment of malignant pleural effusion caused by lung cancer with high polymer and hydroxycamptothecin, GUANGZHOU MEDICAL JOURNAL 33(5) (2002) 40-41.
[3] X. Song, W. Su, H. Zhu, W. Guo, R. Tian, X. Guo, Hydroxycamptothecin combined with hyperagglutination in the treatment of malignant and refractory pleural effusion, CHINESE JOURNAL OF CLINICAL ONCOLOGY 31(11) (2004) 640-642.
[4] G. Yang, J. Bian, Observation on the efficacy of bleomycin combined with high polymer in the treatment of malignant pleural effusion, CHINESE JOURNAL OF CLINICAL ONCOLOGY AND REHABILITATION 11(5) (2004) 464-465.
[5] L. Li, L. Song, Z. Wang, D. Wang, Y. Song, Observation on the short-term efficacy of high polymer combined with carboplatin combined with psychotherapy in the treatment of malignant pleural effusion, Hebei Medical Journal (07) (2006) 597-598.
[6] Y. Zhang, Clinical observation of bleomycin combined with Staphylococcus aureus in the treatment of Cancerous Pleural Effusion, ZHEJIANG CLINICAL MEDICAL JOURNAL 9(9) (2007) 1242-1243.
[7] P. Yan, X. Yu, H. Zhang, X. Piao, C. Ge, Central venous catheter insertion drainage and intrapleural administration staphylococcal enterotoxin C injection plus low dose cisplatin for treatment of malignant pleural effusion compared with bleomycin:a clinical control study, CHINESE JOURNAL OF PRACTICAL MEDICINE 35(24) (2008) 26-27.
[8] S. Wang, J. Zhang, Z. Yin, M. Li, Y. Zhang, F. Zhang, Analysis of Differences of Fever Caused by Cisplatin Combined with Staphylococcin or Lentinan in Malignant Pleural Effusion, JOURNAL OF BASIC AND CLINICAL ONCOLOGY 26(1) (2013) 54-56.
[9] F. Liu, Curative Effect Analysis of Lung Cancer with Malignant Pleural Effusion Trunk Line of Nida Platinum Joint High Perfusion Treatment, Guide of China Medicine (33) (2014) 15-15,16.

v) **The duplicates (nine studies)**

[1] W. Gu, J. Du, Y. Ding, Observation of Staphylococcus aureus in the treatment of malignant pleural effusion, MEDICAL JOURNAL OF CASC 5(1) (2003) 35-36.
[2] H. Huang, N. Sun, D. Wang, Observation on 38 cases of malignant pleural effusion treated with highly agglutinated staphylococcin and cisplatin, Practical Clinical Medicine 13(2) (1999) 43-44.
[3] S. Li, J. Yu, Y. Wu, Therapeutic effect of highly agglutinated staphylococcin and cisplatin combined with intrapleural injection in the treatment of malignant pleural effusion (report of 40 cases), CHINESE JOURNAL OF CLINKAL ONCOLOGY AND REHABILITATION (4) (1997) 69.
[4] J. Liu, P. Han, Clinical analysis of 64 cases of Cancerous Pleural Effusion Treated with highly agglutinative staphylococcin, NINGXIA MEDICAL JOURNAL 23(8) (2001) 490-491.
[5] T. Xu, T. Zhang, H. Gao, J. Zhou, Y. Zheng, Efficacy of docetaxel combined with hyperagglutination in local treatment of non-small cell lung cancer with malignant pleural effusion, New Med (2) (2012).
[6] J. Zhai, Intracavitary injection of high polymer combined with bleomycin through central venous catheter in the treatment of cancerous refractory pleural effusion, Hebei Medical University, 2007.
[7] M. Zhang, Y. Xu, Clinical observation of highly agglutinated staphylococcin combined with

cisplatin in the treatment of malignant pleural effusion, 2000 Beijing Symposium on pharmaceutical biotechnology and its clinical application, Beijing, 2000, pp. 88-89.
[8] Y. Zhang, Curative effect of Hight agglutinative staphylococin add Mitoxantron in treatment of pleural effusions of lung carcinoma, SHAANXI ONCOLOGY MEDICINE 7(2) (1999).
[9] Y. Zhao, Comparative observation on the efficacy of intrapleural injection of different drugs in the treatment of lung cancer pleural effusion, Medical Aesthetics and Cosmetology (6) (2014) 118-118.

vi) The studies without data of primary or secondary indexes (two studies)

[1] M. Li, Therapeutic effect of high polymer combined with cisplatin on malignant pleural effusion, JILIN MEDICAL JOURNAL 32(27) (2011) 5713.
[2] L. Wi, J. Chang, Effects of cisplatin combined with Staphylococcin on tumor markers and vascular endothelial growth factor in patients with pleural effusion of lung adenocarcinoma, Modern Diagnosis & Treatment 32(7) (2021) 1042-1043.
