# Supplementary material for: Intrapleural Perfusion With Staphylococcal Enterotoxin C for Malignant Pleural Effusion: A Clustered Systematic Review and Meta-Analysis
Source: Front Med (Lausanne). 2022 Apr 25;9:816973. doi: 10.3389/fmed.2022.816973 (PMC9081816; doi:10.3389/fmed.2022.816973)
Supplement: Supplementary file 3 [file Data_Sheet_3.PDF]

Appendix 3 The clinical responses of staphylococcal enterotoxin C (Figs.S1, S2, S3, S4 and S5)

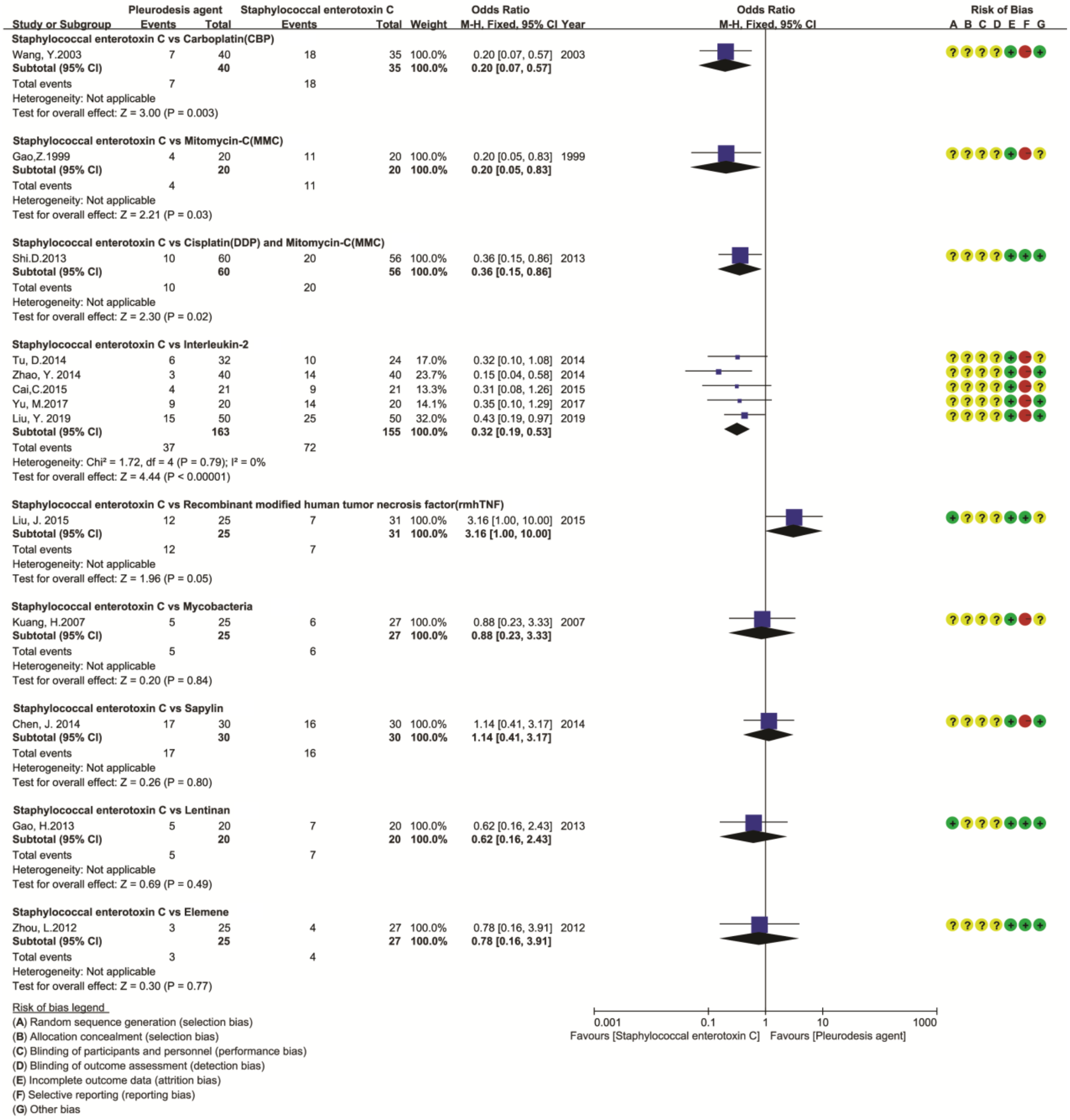

Fig. S1 The complete response of staphylococcal enterotoxin C injection alone

Study or Subgroup	Staphylococcal enterotoxin C Events	Staphylococcal enterotoxin C Total	Pleurodesis agent Events	Pleurodesis agent Total	Weight	Odds Ratio M-H, Fixed, 95% CI	Year
**Staphylococcal enterotoxin C vs Carboplatin(CBP)**							
Wang, Y.2003	21	40	7	35	100.0%	4.42 [1.57, 12.44]	2003
**Subtotal (95% CI)**		**40**		**35**	**100.0%**	**4.42 [1.57, 12.44]**	
Total events	21		7				
Heterogeneity: Not applicable							
Test for overall effect: Z = 2.81 (P = 0.005)							
**Staphylococcal enterotoxin C vs Mitomycin-C(MMC)**							
Gao,Z.1999	7	20	3	20	100.0%	3.05 [0.66, 14.14]	1999
**Subtotal (95% CI)**		**20**		**20**	**100.0%**	**3.05 [0.66, 14.14]**	
Total events	7		3				
Heterogeneity: Not applicable							
Test for overall effect: Z = 1.43 (P = 0.15)							
**Staphylococcal enterotoxin C vs Cisplatin(DDP) and Mitomycin-C(MMC)**							
Shi.D.2013	14	60	16	56	100.0%	0.76 [0.33, 1.75]	2013
**Subtotal (95% CI)**		**60**		**56**	**100.0%**	**0.76 [0.33, 1.75]**	
Total events	14		16				
Heterogeneity: Not applicable							
Test for overall effect: Z = 0.64 (P = 0.52)							
**Staphylococcal enterotoxin C vs Interleukin-2**							
Zhao, Y. 2014	22	40	16	40	31.9%	1.83 [0.75, 4.45]	2014
Tu, D.2014	10	32	8	24	27.8%	0.91 [0.29, 2.82]	2014
Cai,C.2015	8	21	6	21	16.4%	1.54 [0.42, 5.61]	2015
Yu, M.2017	4	20	2	20	7.1%	2.25 [0.36, 13.97]	2017
Liu, Y. 2019	12	50	5	50	16.8%	2.84 [0.92, 8.79]	2019
**Subtotal (95% CI)**		**163**		**155**	**100.0%**	**1.73 [1.03, 2.88]**	
Total events	56		37				
Heterogeneity: $Chi^2 = 2.11$, df = 4 (P = 0.71); $I^2 = 0\%$							
Test for overall effect: Z = 2.09 (P = 0.04)							
**Staphylococcal enterotoxin C vs Recombinant modified human tumor necrosis factor(rmhTNF)**							
Liu, J. 2015	6	25	13	31	100.0%	0.44 [0.14, 1.40]	2015
**Subtotal (95% CI)**		**25**		**31**	**100.0%**	**0.44 [0.14, 1.40]**	
Total events	6		13				
Heterogeneity: Not applicable							
Test for overall effect: Z = 1.39 (P = 0.16)							
**Staphylococcal enterotoxin C vs Mycobacteria**							
Kuang, H.2007	4	25	6	27	100.0%	0.67 [0.16, 2.71]	2007
**Subtotal (95% CI)**		**25**		**27**	**100.0%**	**0.67 [0.16, 2.71]**	
Total events	4		6				
Heterogeneity: Not applicable							
Test for overall effect: Z = 0.57 (P = 0.57)							
**Staphylococcal enterotoxin C vs Sapylin**							
Chen, J. 2014	1	30	0	30	100.0%	3.10 [0.12, 79.23]	2014
**Subtotal (95% CI)**		**30**		**30**	**100.0%**	**3.10 [0.12, 79.23]**	
Total events	1		0				
Heterogeneity: Not applicable							
Test for overall effect: Z = 0.68 (P = 0.49)							
**Staphylococcal enterotoxin C vs Lentinan**							
Gao, H.2013	13	20	10	20	100.0%	1.86 [0.52, 6.61]	2013
**Subtotal (95% CI)**		**20**		**20**	**100.0%**	**1.86 [0.52, 6.61]**	
Total events	13		10				
Heterogeneity: Not applicable							
Test for overall effect: Z = 0.96 (P = 0.34)							
**Staphylococcal enterotoxin C vs Elemene**							
Zhou, L.2012	20	25	22	27	100.0%	0.91 [0.23, 3.61]	2012
**Subtotal (95% CI)**		**25**		**27**	**100.0%**	**0.91 [0.23, 3.61]**	
Total events	20		22				
Heterogeneity: Not applicable							
Test for overall effect: Z = 0.14 (P = 0.89)							

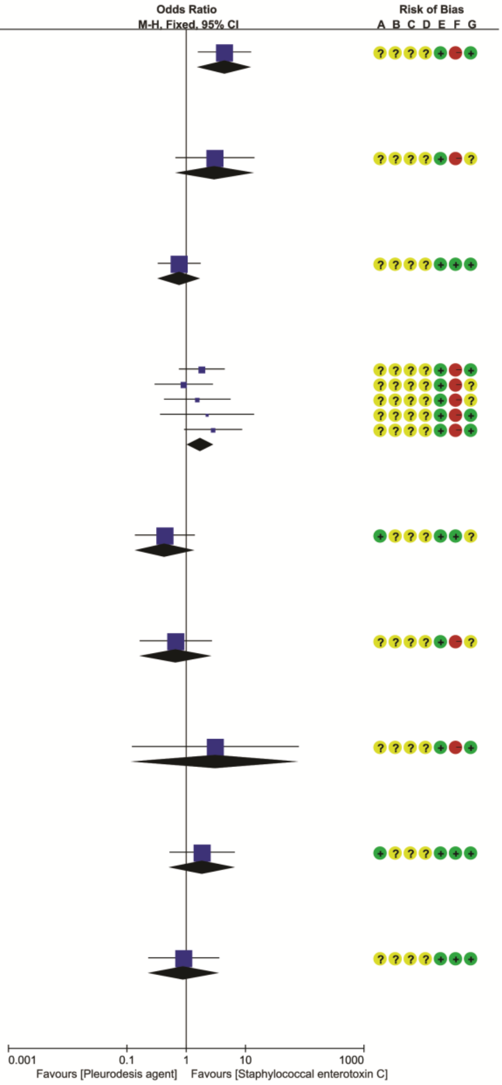

Risk of bias legend
(**A**) Random sequence generation (selection bias)
(**B**) Allocation concealment (selection bias)
(**C**) Blinding of participants and personnel (performance bias)
(**D**) Blinding of outcome assessment (detection bias)
(**E**) Incomplete outcome data (attrition bias)
(**F**) Selective reporting (reporting bias)
(**G**) Other bias

Fig. S2 The pleurodesis failure of staphylococcal enterotoxin C alone

Study or Subgroup	Staphylococcal enterotoxin C Events	Staphylococcal enterotoxin C Total	Chemical agents Events	Chemical agents Total	Weight	Odds Ratio M-H, Fixed, 95% CI	Year
**Staphylococcal enterotoxin C plus carboplatin (CBP)**							
Xu, Z.2004	10	21	3	15	28.8%	3.64 [0.79, 16.76]	2004
Liang, J.2005	8	20	4	18	39.7%	2.33 [0.56, 9.72]	2005
Jiang, D.2006	8	23	3	22	31.4%	3.38 [0.76, 14.98]	2006
**Subtotal (95% CI)**		**64**		**55**	**100.0%**	**3.04 [1.30, 7.12]**	
Total events	26		10				
Heterogeneity: Chi² = 0.20, df = 2 (P = 0.90); I² = 0%							
Test for overall effect: Z = 2.56 (P = 0.01)							
**Staphylococcal enterotoxin C plus nedaplatin (NDP)**							
Xu, T.2010	9	32	2	26	100.0%	4.70 [0.92, 24.10]	2010
**Subtotal (95% CI)**		**32**		**26**	**100.0%**	**4.70 [0.92, 24.10]**	
Total events	9		2				
Heterogeneity: Not applicable							
Test for overall effect: Z = 1.85 (P = 0.06)							
**Staphylococcal enterotoxin C plus bleomycin (BLM)**							
Chen, Y.2000	16	30	8	30	17.8%	3.14 [1.07, 9.27]	2000
Shen, S.2006	12	25	6	24	15.2%	2.77 [0.82, 9.31]	2006
Mo,W.2008	23	50	12	47	31.9%	2.48 [1.05, 5.87]	2008
Yuan, X.2009	29	60	13	50	35.0%	2.66 [1.18, 5.98]	2009
**Subtotal (95% CI)**		**165**		**151**	**100.0%**	**2.71 [1.68, 4.36]**	
Total events	80		39				
Heterogeneity: Chi² = 0.11, df = 3 (P = 0.99); I² = 0%							
Test for overall effect: Z = 4.10 (P < 0.0001)							
**Staphylococcal enterotoxin C plus mitomycin-C (MMC)**							
Zhang, S. 2000	9	36	4	33	38.1%	2.42 [0.67, 8.77]	2000
Tao, M.2004	5	16	3	16	25.1%	1.97 [0.38, 10.17]	2004
Ding, H.2006	7	24	4	21	36.8%	1.75 [0.43, 7.10]	2006
**Subtotal (95% CI)**		**76**		**70**	**100.0%**	**2.06 [0.91, 4.67]**	
Total events	21		11				
Heterogeneity: Chi² = 0.11, df = 2 (P = 0.94); I² = 0%							
Test for overall effect: Z = 1.73 (P = 0.08)							
**Staphylococcal enterotoxin C plus etoposide(VP-16)**							
Tian, C.2001	17	40	10	38	52.8%	2.07 [0.80, 5.38]	2001
Liu,Z.2005	12	29	9	29	47.2%	1.57 [0.53, 4.62]	2005
**Subtotal (95% CI)**		**69**		**67**	**100.0%**	**1.83 [0.90, 3.75]**	
Total events	29		19				
Heterogeneity: Chi² = 0.14, df = 1 (P = 0.71); I² = 0%							
Test for overall effect: Z = 1.66 (P = 0.10)							
**Staphylococcal enterotoxin C plus 5-fluorouracil (5-FU)**							
Sun, W. 2003	14	31	6	31	61.0%	3.43 [1.10, 10.70]	2003
Huang, D.2006	9	30	3	30	39.0%	3.86 [0.93, 16.05]	2006
**Subtotal (95% CI)**		**61**		**61**	**100.0%**	**3.60 [1.48, 8.75]**	
Total events	23		9				
Heterogeneity: Chi² = 0.02, df = 1 (P = 0.90); I² = 0%							
Test for overall effect: Z = 2.82 (P = 0.005)							
**Staphylococcal enterotoxin C plus mitoxantrone (MTZ)**							
Zhang, Y. 1999	13	38	2	20	100.0%	4.68 [0.94, 23.35]	1999
**Subtotal (95% CI)**		**38**		**20**	**100.0%**	**4.68 [0.94, 23.35]**	
Total events	13		2				
Heterogeneity: Not applicable							
Test for overall effect: Z = 1.88 (P = 0.06)							
**Staphylococcal enterotoxin C plus adriamycin (ADM)**							
Guan, C.2004	14	25	8	23	100.0%	2.39 [0.74, 7.66]	2004
**Subtotal (95% CI)**		**25**		**23**	**100.0%**	**2.39 [0.74, 7.66]**	
Total events	14		8				
Heterogeneity: Not applicable							
Test for overall effect: Z = 1.46 (P = 0.14)							
**Staphylococcal enterotoxin C plus docetaxel**							
Xu,T.2013	6	28	4	28	100.0%	1.64 [0.41, 6.58]	2013
**Subtotal (95% CI)**		**28**		**28**	**100.0%**	**1.64 [0.41, 6.58]**	
Total events	6		4				
Heterogeneity: Not applicable							
Test for overall effect: Z = 0.69 (P = 0.49)							
**Staphylococcal enterotoxin C plus DDP and VP-16**							
Zhang, L. 2001	3	20	2	21	100.0%	1.68 [0.25, 11.27]	2001
**Subtotal (95% CI)**		**20**		**21**	**100.0%**	**1.68 [0.25, 11.27]**	
Total events	3		2				
Heterogeneity: Not applicable							
Test for overall effect: Z = 0.53 (P = 0.60)							
**Staphylococcal enterotoxin C plus ADM, 5 FU/CBP**							
Fu, J.2000	24	28	2	27	100.0%	75.00 [12.55, 448.04]	2000
**Subtotal (95% CI)**		**28**		**27**	**100.0%**	**75.00 [12.55, 448.04]**	
Total events	24		2				
Heterogeneity: Not applicable							
Test for overall effect: Z = 4.73 (P < 0.00001)							
**Staphylococcal enterotoxin C plus ADM and DDP**							
Tang, Z. 2000	9	30	5	30	100.0%	2.14 [0.62, 7.39]	2000
**Subtotal (95% CI)**		**30**		**30**	**100.0%**	**2.14 [0.62, 7.39]**	
Total events	9		5				
Heterogeneity: Not applicable							
Test for overall effect: Z = 1.21 (P = 0.23)							

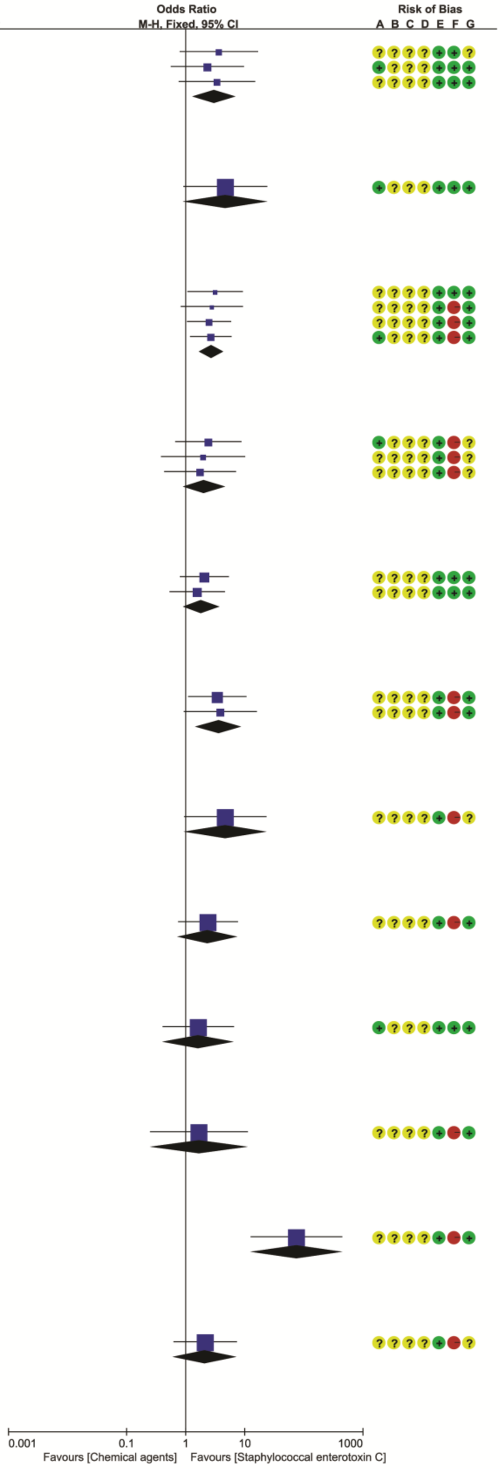

Risk of bias legend
(**A**) Random sequence generation (selection bias)
(**B**) Allocation concealment (selection bias)
(**C**) Blinding of participants and personnel (performance bias)
(**D**) Blinding of outcome assessment (detection bias)
(**E**) Incomplete outcome data (attrition bias)
(**F**) Selective reporting (reporting bias)
(**G**) Other bias

Fig. S3The complete response of staphylococcal enterotoxin C and chemical agents

Study or Subgroup	Staphylococcal enterotoxin C Events	Staphylococcal enterotoxin C Total	Chemical agents Events	Chemical agents Total	Weight	Odds Ratio M-H, Fixed, 95% CI	Year	Risk of Bias A B C D E F G
**Staphylococcal enterotoxin C plus carboplatin (CBP)**								
Xu, Z.2004	3	21	7	15	31.1%	0.19 [0.04, 0.93]	2004	? ? ? ? + + ?
Liang, J.2005	2	20	7	18	29.4%	0.17 [0.03, 1.00]	2005	+ ? ? ? + + +
Jiang, D.2006	3	23	10	22	39.5%	0.18 [0.04, 0.79]	2006	? ? ? ? + + +
**Subtotal (95% CI)**		**64**		**55**	**100.0%**	**0.18 [0.07, 0.46]**		
Total events	8		24					
Heterogeneity: Chi² = 0.01, df = 2 (P = 1.00); I² = 0%								
Test for overall effect: Z = 3.64 (P = 0.0003)								
**Staphylococcal enterotoxin C plus nedaplatin (NDP)**								
Xu, T.2010	6	32	15	26	100.0%	0.17 [0.05, 0.55]	2010	+ ? ? ? + + +
**Subtotal (95% CI)**		**32**		**26**	**100.0%**	**0.17 [0.05, 0.55]**		
Total events	6		15					
Heterogeneity: Not applicable								
Test for overall effect: Z = 2.95 (P = 0.003)								
**Staphylococcal enterotoxin C plus bleomycin (BLM)**								
Chen, Y.2000	2	30	10	30	16.2%	0.14 [0.03, 0.72]	2000	? ? ? ? + + +
Shen, S.2006	4	25	11	24	16.3%	0.23 [0.06, 0.86]	2006	? ? ? ? + − +
Mo,W.2008	7	50	21	47	32.2%	0.20 [0.08, 0.54]	2008	? ? ? ? + − +
Yuan, X.2009	9	60	22	50	35.3%	0.22 [0.09, 0.55]	2009	+ ? ? ? + − +
**Subtotal (95% CI)**		**165**		**151**	**100.0%**	**0.20 [0.12, 0.36]**		
Total events	22		64					
Heterogeneity: Chi² = 0.25, df = 3 (P = 0.97); I² = 0%								
Test for overall effect: Z = 5.58 (P < 0.00001)								
**Staphylococcal enterotoxin C plus mitomycin-C (MMC)**								
Zhang, S. 2000	11	36	21	33	47.6%	0.25 [0.09, 0.69]	2000	+ ? ? ? + − ?
Tao, M.2004	2	16	10	16	27.4%	0.09 [0.01, 0.52]	2004	? ? ? ? + − ?
Ding, H.2006	4	24	9	21	25.0%	0.27 [0.07, 1.06]	2006	? ? ? ? + − ?
**Subtotal (95% CI)**		**76**		**70**	**100.0%**	**0.21 [0.10, 0.44]**		
Total events	17		40					
Heterogeneity: Chi² = 1.20, df = 2 (P = 0.55); I² = 0%								
Test for overall effect: Z = 4.19 (P < 0.0001)								
**Staphylococcal enterotoxin C plus etoposide(VP-16)**								
Tian, C.2001	6	40	21	38	61.2%	0.14 [0.05, 0.42]	2001	? ? ? ? + + +
Liu,Z.2005	5	29	14	29	38.8%	0.22 [0.07, 0.75]	2005	? ? ? ? + + +
**Subtotal (95% CI)**		**69**		**67**	**100.0%**	**0.17 [0.08, 0.39]**		
Total events	11		35					
Heterogeneity: Chi² = 0.29, df = 1 (P = 0.59); I² = 0%								
Test for overall effect: Z = 4.27 (P < 0.0001)								
**Staphylococcal enterotoxin C plus 5-fluorouracil (5-FU)**								
Sun, W. 2003	5	31	17	31	51.7%	0.16 [0.05, 0.52]	2003	? ? ? ? + − +
Huang, D.2006	5	30	16	30	48.3%	0.17 [0.05, 0.58]	2006	? ? ? ? + − +
**Subtotal (95% CI)**		**61**		**61**	**100.0%**	**0.17 [0.07, 0.39]**		
Total events	10		33					
Heterogeneity: Chi² = 0.01, df = 1 (P = 0.91); I² = 0%								
Test for overall effect: Z = 4.16 (P < 0.0001)								
**Staphylococcal enterotoxin C plus mitoxantrone (MTZ)**								
Zhang, Y. 1999	5	38	13	20	100.0%	0.08 [0.02, 0.30]	1999	? ? ? ? + − ?
**Subtotal (95% CI)**		**38**		**20**	**100.0%**	**0.08 [0.02, 0.30]**		
Total events	5		13					
Heterogeneity: Not applicable								
Test for overall effect: Z = 3.74 (P = 0.0002)								
**Staphylococcal enterotoxin C plus adriamycin (ADM)**								
Guan, C.2004	3	25	10	23	100.0%	0.18 [0.04, 0.76]	2004	? ? ? ? + − +
**Subtotal (95% CI)**		**25**		**23**	**100.0%**	**0.18 [0.04, 0.76]**		
Total events	3		10					
Heterogeneity: Not applicable								
Test for overall effect: Z = 2.32 (P = 0.02)								
**Staphylococcal enterotoxin C plus docetaxel**								
Xu,T.2013	4	28	12	28	100.0%	0.22 [0.06, 0.81]	2013	+ ? ? ? + + +
**Subtotal (95% CI)**		**28**		**28**	**100.0%**	**0.22 [0.06, 0.81]**		
Total events	4		12					
Heterogeneity: Not applicable								
Test for overall effect: Z = 2.27 (P = 0.02)								
**Staphylococcal enterotoxin C plus DDP and VP-16**								
Zhang, L. 2001	10	20	12	21	100.0%	0.75 [0.22, 2.57]	2001	? ? ? ? + − +
**Subtotal (95% CI)**		**20**		**21**	**100.0%**	**0.75 [0.22, 2.57]**		
Total events	10		12					
Heterogeneity: Not applicable								
Test for overall effect: Z = 0.46 (P = 0.65)								
**Staphylococcal enterotoxin C plus ADM, 5 FU/CBP**								
Fu, J.2000	2	28	21	27	100.0%	0.02 [0.00, 0.12]	2000	? ? ? ? + − +
**Subtotal (95% CI)**		**28**		**27**	**100.0%**	**0.02 [0.00, 0.12]**		
Total events	2		21					
Heterogeneity: Not applicable								
Test for overall effect: Z = 4.40 (P < 0.0001)								
**Staphylococcal enterotoxin C plus ADM and DDP**								
Tang, Z. 2000	5	30	19	30	100.0%	0.12 [0.03, 0.39]	2000	? ? ? ? + − ?
**Subtotal (95% CI)**		**30**		**30**	**100.0%**	**0.12 [0.03, 0.39]**		
Total events	5		19					
Heterogeneity: Not applicable								
Test for overall effect: Z = 3.48 (P = 0.0005)								

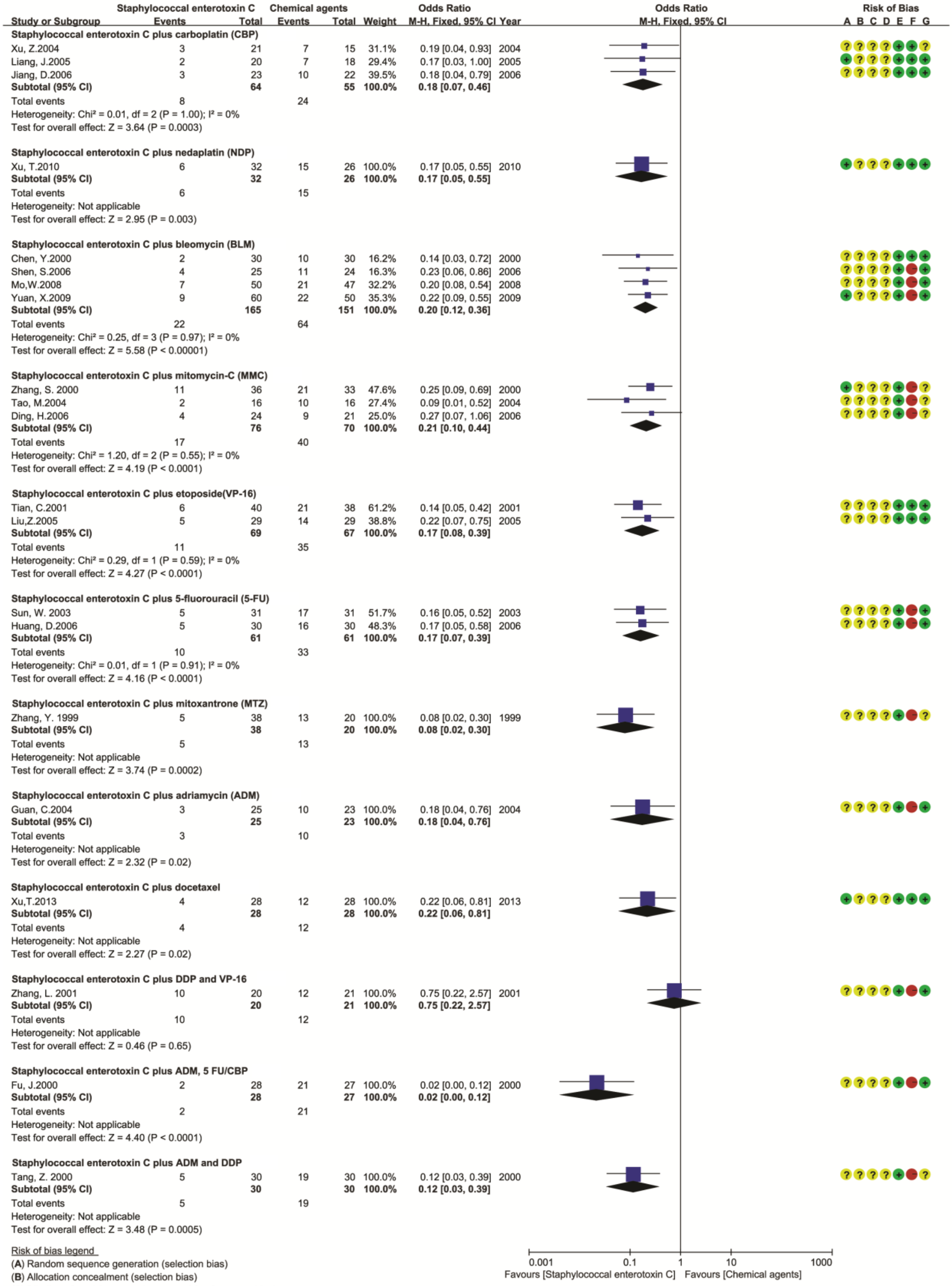

Risk of bias legend
(**A**) Random sequence generation (selection bias)
(**B**) Allocation concealment (selection bias)
(**C**) Blinding of participants and personnel (performance bias)
(**D**) Blinding of outcome assessment (detection bias)
(**E**) Incomplete outcome data (attrition bias)
(**F**) Selective reporting (reporting bias)
(**G**) Other bias

Fig. S4 The pleurodesis failure of staphylococcal enterotoxin C and chemical agents

Study or Subgroup	Staphylococcal enterotoxin C Events	Staphylococcal enterotoxin C Total	Pleurodesis agents Events	Pleurodesis agents Total	Weight	Odds Ratio M-H, Fixed, 95% CI	Year
**Staphylococcal enterotoxin C vs Cisplatin(DDP)**							
Hu, H. 2000	2	13	3	13	22.7%	0.61 [0.08, 4.41]	2000
Xue, Z.2007	1	33	5	32	44.1%	0.17 [0.02, 1.53]	2007
Liu, J. 2015	5	25	5	29	33.2%	1.20 [0.30, 4.74]	2015
**Subtotal (95% CI)**		**71**		**74**	**100.0%**	**0.61 [0.24, 1.58]**	
Total events	8		13				
Heterogeneity: Chi² = 2.23, df = 2 (P = 0.33); I² = 10%							
Test for overall effect: Z = 1.02 (P = 0.31)							
**Staphylococcal enterotoxin C vs Cisplatin(DDP) and Mitomycin-C(MMC)**							
Shi.D.2013	2	60	4	56	100.0%	0.45 [0.08, 2.55]	2013
**Subtotal (95% CI)**		**60**		**56**	**100.0%**	**0.45 [0.08, 2.55]**	
Total events	2		4				
Heterogeneity: Not applicable							
Test for overall effect: Z = 0.90 (P = 0.37)							
**Staphylococcal enterotoxin C vs Recombinant modified human tumor necrosis factor(rmhTNF)**							
Liu, J. 2015	5	25	2	31	100.0%	3.63 [0.64, 20.57]	2015
**Subtotal (95% CI)**		**25**		**31**	**100.0%**	**3.63 [0.64, 20.57]**	
Total events	5		2				
Heterogeneity: Not applicable							
Test for overall effect: Z = 1.45 (P = 0.15)							
**Staphylococcal enterotoxin C vs Mycobacteria**							
Kuang, H.2007	0	25	2	27	100.0%	0.20 [0.01, 4.38]	2007
**Subtotal (95% CI)**		**25**		**27**	**100.0%**	**0.20 [0.01, 4.38]**	
Total events	0		2				
Heterogeneity: Not applicable							
Test for overall effect: Z = 1.02 (P = 0.31)							
**Staphylococcal enterotoxin C plus cisplatin(DDP)**							
Li, H.1998	1	68	4	42	9.0%	0.14 [0.02, 1.31]	1998
Wang, L. 2002	2	29	5	29	8.6%	0.36 [0.06, 2.00]	2000
Lang, F.2001	2	56	5	21	13.0%	0.12 [0.02, 0.67]	2001
Li, J.2003	3	23	7	21	11.8%	0.30 [0.07, 1.37]	2003
Liu, M.2005	1	50	8	48	14.8%	0.10 [0.01, 0.85]	2005
Chen, Z.2006	1	12	2	11	3.5%	0.41 [0.03, 5.28]	2006
Zheng, F.2007	2	24	3	20	5.6%	0.52 [0.08, 3.44]	2007
Gui, Q. 2010	1	25	2	25	3.6%	0.48 [0.04, 5.65]	2010
Liu, Q.2010	2	27	3	20	5.9%	0.45 [0.07, 3.01]	2010
Yu, L. 2013	1	23	2	22	3.6%	0.45 [0.04, 5.40]	2013
Liu, J. 2015	1	30	5	29	9.1%	0.17 [0.02, 1.52]	2015
Zhou, Z.2015	0	21	1	21	2.7%	0.32 [0.01, 8.26]	2015
Wu, Y. 2019	2	46	5	46	8.8%	0.37 [0.07, 2.03]	2019
**Subtotal (95% CI)**		**434**		**355**	**100.0%**	**0.27 [0.16, 0.47]**	
Total events	19		52				
Heterogeneity: Chi² = 3.67, df = 12 (P = 0.99); I² = 0%							
Test for overall effect: Z = 4.70 (P < 0.00001)							
**Staphylococcal enterotoxin C plus bleomycin (BLM)**							
Shen, S.2006	1	25	5	24	33.1%	0.16 [0.02, 1.47]	2006
Mo,W.2008	2	50	10	47	66.9%	0.15 [0.03, 0.75]	2008
**Subtotal (95% CI)**		**75**		**71**	**100.0%**	**0.16 [0.04, 0.56]**	
Total events	3		15				
Heterogeneity: Chi² = 0.00, df = 1 (P = 0.98); I² = 0%							
Test for overall effect: Z = 2.83 (P = 0.005)							
**Staphylococcal enterotoxin C plus 5-fluorouracil (5-FU)**							
Sun, W. 2003	1	31	8	31	100.0%	0.10 [0.01, 0.82]	2003
**Subtotal (95% CI)**		**31**		**31**	**100.0%**	**0.10 [0.01, 0.82]**	
Total events	1		8				
Heterogeneity: Not applicable							
Test for overall effect: Z = 2.14 (P = 0.03)							
**Staphylococcal enterotoxin C plus mitoxantrone (MTZ)**							
Zhang, Y. 1999	1	38	3	20	100.0%	0.15 [0.01, 1.58]	1999
**Subtotal (95% CI)**		**38**		**20**	**100.0%**	**0.15 [0.01, 1.58]**	
Total events	1		3				
Heterogeneity: Not applicable							
Test for overall effect: Z = 1.58 (P = 0.12)							
**Staphylococcal enterotoxin C plus ADM, 5 FU/CBP**							
Fu, J.2000	1	28	10	27	100.0%	0.06 [0.01, 0.54]	2000
**Subtotal (95% CI)**		**28**		**27**	**100.0%**	**0.06 [0.01, 0.54]**	
Total events	1		10				
Heterogeneity: Not applicable							
Test for overall effect: Z = 2.53 (P = 0.01)							

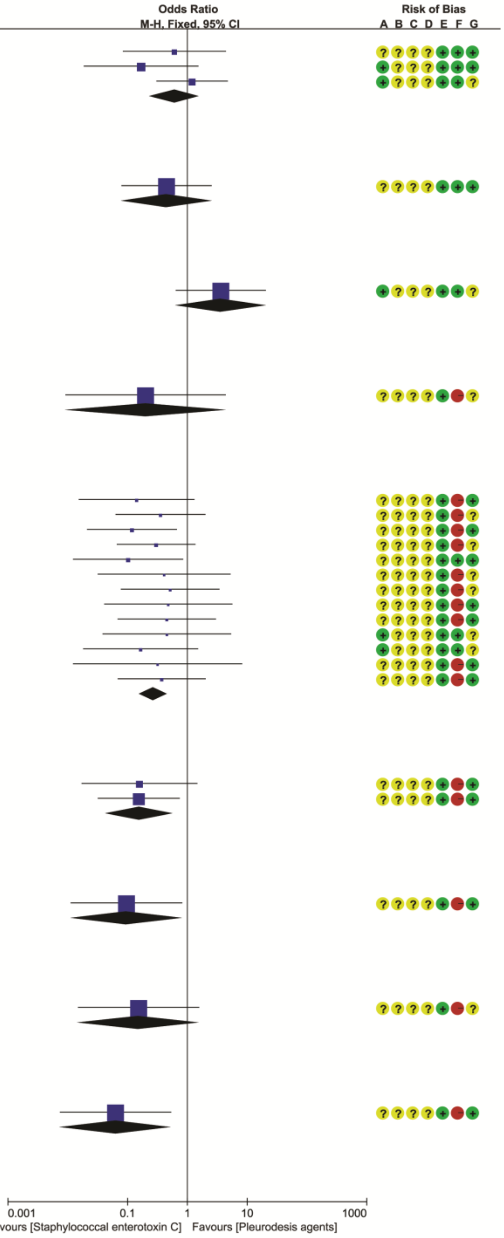

Risk of bias legend

(**A**) Random sequence generation (selection bias)
(**B**) Allocation concealment (selection bias)
(**C**) Blinding of participants and personnel (performance bias)
(**D**) Blinding of outcome assessment (detection bias)
(**E**) Incomplete outcome data (attrition bias)
(**F**) Selective reporting (reporting bias)
(**G**) Other bias

Fig. S5 The disease progression of staphylococcal enterotoxin C
