# Supplementary material for: Intrapleural Perfusion With Staphylococcal Enterotoxin C for Malignant Pleural Effusion: A Clustered Systematic Review and Meta-Analysis
Source: Front Med (Lausanne). 2022 Apr 25;9:816973. doi: 10.3389/fmed.2022.816973 (PMC9081816; doi:10.3389/fmed.2022.816973)
Supplement: Supplementary file 4 [file Data_Sheet_4.PDF]

Appendix.4 Meta-analysis results of adverse events (Figs.S6-S13)

Study or Subgroup	Staphylococcal enterotoxin C Events	Staphylococcal enterotoxin C Total	Cisplatin Events	Cisplatin Total	Weight	Odds Ratio M-H, Fixed, 95% CI	Year
**Staphylococcal enterotoxin C (SEC) vs Cisplatin(DDP)**							
Xue, Z.2007	0	33	6	32	31.0%	0.06 [0.00, 1.13]	2007
Cheng, J.2010	0	30	9	30	44.6%	0.04 [0.00, 0.67]	2010
Liu, J. 2015	0	25	1	29	6.5%	0.37 [0.01, 9.56]	2015
Yu, M.2017	1	20	1	20	4.5%	1.00 [0.06, 17.18]	2017
Luo, D.2018	2	30	3	30	13.4%	0.64 [0.10, 4.15]	2018
**Subtotal (95% CI)**		**138**		**141**	**100.0%**	**0.19 [0.07, 0.53]**	
Total events	3		20				
Heterogeneity: Chi² = 4.91, df = 4 (P = 0.30); I² = 19%							
Test for overall effect: Z = 3.17 (P = 0.002)							
**Staphylococcal enterotoxin C plus cisplatin(DDP)**							
Wang, Lei.2002	0	21	0	19		Not estimable	2002
Duan, R.2003	0	76	0	76		Not estimable	2003
Liu, S.2005	0	32	0	29		Not estimable	2005
Huang, Y.2005	0	27	2	21	8.2%	0.14 [0.01, 3.12]	2005
Liu, M.2005	5	50	10	48	27.5%	0.42 [0.13, 1.34]	2005
Zhang, Z.2006	0	16	1	16	4.4%	0.31 [0.01, 8.28]	2006
Yin, W.2007	3	19	6	19	15.2%	0.41 [0.08, 1.95]	2007
Cheng, J.2010	2	30	9	30	25.2%	0.17 [0.03, 0.85]	2010
Li, Z. 2010	0	230	0	230		Not estimable	2010
Yao, X.2014	1	25	2	25	5.8%	0.48 [0.04, 5.65]	2014
Liu, J. 2015	1	30	1	29	2.9%	0.97 [0.06, 16.20]	2015
Yu, M.2017	2	20	1	20	2.7%	2.11 [0.18, 25.35]	2017
Luo, D.2018	3	30	3	30	8.1%	1.00 [0.19, 5.40]	2018
**Subtotal (95% CI)**		**606**		**592**	**100.0%**	**0.44 [0.24, 0.80]**	
Total events	17		35				
Heterogeneity: Chi² = 4.67, df = 8 (P = 0.79); I² = 0%							
Test for overall effect: Z = 2.68 (P = 0.007)							

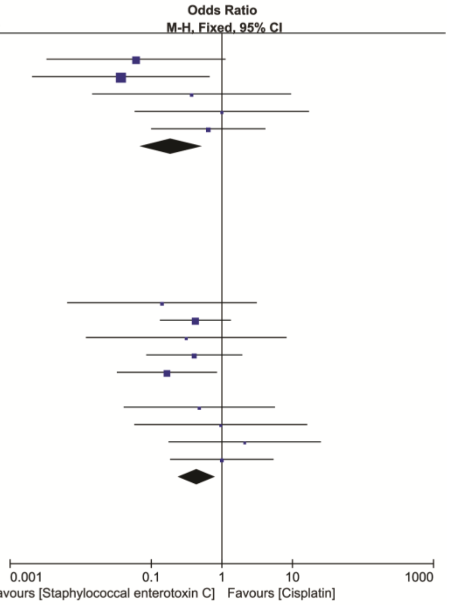

Fig.S6 The analysis of myelosuppression between the two groups

Study or Subgroup	Staphylococcal enterotoxin C Events	Staphylococcal enterotoxin C Total	Cisplatin Events	Cisplatin Total	Weight	Odds Ratio M-H, Fixed, 95% CI	Year
**Staphylococcal enterotoxin C (SEC) vs Cisplatin(DDP)**							
Hu, H. 2000	0	13	3	13	6.1%	0.11 [0.01, 2.40]	2000
Jia, X.2000	0	15	11	15	20.0%	0.01 [0.00, 0.26]	2000
Fang, X.2004	0	15	6	15	11.3%	0.05 [0.00, 0.94]	2004
Zhou, F.2007	0	14	2	12	4.7%	0.14 [0.01, 3.34]	2007
Xue, Z.2007	1	33	0	32	0.9%	3.00 [0.12, 76.40]	2007
Wang, H.2009	0	28	9	28	16.8%	0.04 [0.00, 0.66]	2009
Wu, J.2010	0	34	3	30	6.6%	0.11 [0.01, 2.30]	2010
Chen, J.2010	4	32	12	32	18.9%	0.24 [0.07, 0.85]	2010
Xu, Q.2012	0	26	8	27	14.7%	0.04 [0.00, 0.80]	2012
**Subtotal (95% CI)**		**210**		**204**	**100.0%**	**0.11 [0.05, 0.23]**	
Total events	5		54				
Heterogeneity: Chi² = 8.67, df = 8 (P = 0.37); I² = 8%							
Test for overall effect: Z = 5.86 (P < 0.00001)							
**Staphylococcal enterotoxin C plus cisplatin(DDP)**							
Li, S.1997	0	20	3	20	2.1%	0.12 [0.01, 2.53]	1997
Zhang, Q.1999	0	15	0	15		Not estimable	1999
Jia, X.2000	5	15	11	15	4.6%	0.18 [0.04, 0.87]	2000
Wang, Lei.2002	0	21	0	19		Not estimable	2002
Xu, Q.2003	1	32	9	30	5.6%	0.08 [0.01, 0.64]	2003
Mao, W. 2004	1	24	4	16	2.9%	0.13 [0.01, 1.30]	2004
Chen, H.2004	4	24	6	24	3.1%	0.60 [0.15, 2.47]	2004
Zhu, F.2004	12	34	24	30	10.3%	0.14 [0.04, 0.43]	2004
Fang, X.2004	2	15	6	15	3.2%	0.23 [0.04, 1.41]	2004
Chen, J.2005	3	27	4	24	2.3%	0.63 [0.12, 3.13]	2005
Zhang, T. 2005	5	23	7	21	3.6%	0.56 [0.14, 2.13]	2005
Zhao, Z.2006	6	32	18	32	9.1%	0.18 [0.06, 0.56]	2006
Pan, Y.2006	4	28	6	20	3.7%	0.39 [0.09, 1.62]	2006
Xiong, C.2006	3	22	5	22	2.7%	0.54 [0.11, 2.59]	2006
Yue, Z.2006	4	33	10	28	5.9%	0.25 [0.07, 0.91]	2006
Yin, W.2007	3	19	6	19	3.1%	0.41 [0.08, 1.95]	2007
Zhou, F.2007	3	16	2	12	1.2%	1.15 [0.16, 8.27]	2007
Huang, W.2008	6	28	9	28	4.4%	0.58 [0.17, 1.91]	2008
Li, X.2008	3	25	7	25	3.8%	0.35 [0.08, 1.55]	2008
Wang, H.2009	6	28	9	28	4.4%	0.58 [0.17, 1.91]	2009
Qu, Y.2010	6	40	12	40	6.4%	0.41 [0.14, 1.24]	2010
Gui, Q. 2010	5	25	12	25	6.0%	0.27 [0.08, 0.95]	2010
Zhang, C. 2011	3	19	3	19	1.6%	1.00 [0.17, 5.72]	2011
Xu, Q.2012	6	27	8	27	3.9%	0.68 [0.20, 2.31]	2012
Yu, L. 2013	3	23	4	22	2.2%	0.68 [0.13, 3.43]	2013
Du, Y.2013	0	60	2	60	1.5%	0.19 [0.01, 4.11]	2013
Wang, Y. 2018	2	37	4	37	2.4%	0.47 [0.08, 2.75]	2018
**Subtotal (95% CI)**		**712**		**673**	**100.0%**	**0.36 [0.27, 0.48]**	
Total events	96		191				
Heterogeneity: Chi² = 16.41, df = 24 (P = 0.87); I² = 0%							
Test for overall effect: Z = 7.02 (P < 0.00001)							

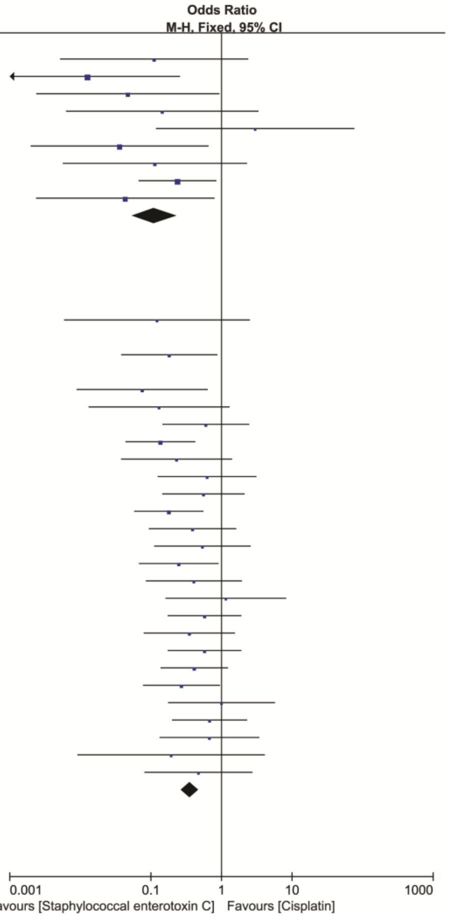

Fig.S7 The analysis of leukopenia between the two groups

Study or Subgroup	Staphylococcal enterotoxin C Events	Total	Cisplatin Events	Total	Weight	Odds Ratio M-H, Random, 95% CI
Chen, J.2010	4	32	20	32	9.9%	0.09 [0.02, 0.30]
Chen, J. 2014	4	30	9	30	9.7%	0.36 [0.10, 1.33]
Cheng, J.2010	0	30	14	30	4.4%	0.02 [0.00, 0.33]
Fang, X.2004	1	15	8	15	6.0%	0.06 [0.01, 0.60]
Hu, H. 2000	0	13	6	13	4.1%	0.04 [0.00, 0.87]
Jia, X.2000	0	15	6	15	4.2%	0.05 [0.00, 0.94]
Liu, J. 2015	3	25	4	29	8.4%	0.85 [0.17, 4.23]
Sun, C.2007	0	22	10	24	4.3%	0.03 [0.00, 0.56]
Wang, H.2009	3	28	13	28	9.3%	0.14 [0.03, 0.57]
Wu, J.2010	5	34	4	30	9.3%	1.12 [0.27, 4.62]
Xu, Q.2012	2	26	12	27	8.3%	0.10 [0.02, 0.53]
Xue, Z.2007	3	33	9	32	9.3%	0.26 [0.06, 1.05]
Zhang, L.2009	0	37	20	35	4.4%	0.01 [0.00, 0.18]
Zhang, M.2008	0	35	21	34	4.4%	0.01 [0.00, 0.16]
Zhou, F.2007	0	14	2	12	3.9%	0.14 [0.01, 3.34]
**Total (95% CI)**		**389**		**386**	**100.0%**	**0.12 [0.06, 0.26]**
Total events	25		158			

Heterogeneity: Tau² = 0.96; Chi² = 28.97, df = 14 (P = 0.01); I² = 52%
Test for overall effect: Z = 5.62 (P < 0.00001)

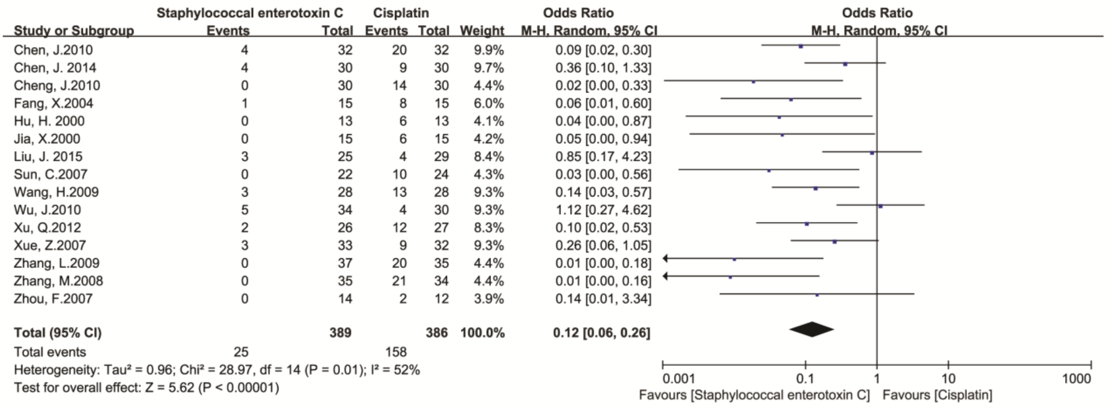

Fig.S8a The gastrointestinal reactions of staphylococcal enterotoxin C alone

Study or Subgroup	Staphylococcal enterotoxin C Events	Total	Cisplatin Events	Total	Weight	Odds Ratio M-H, Fixed, 95% CI	Year
Li, S.1997	0	20	7	20	1.8%	0.04 [0.00, 0.83]	1997
Qiu, Y.1999	18	42	29	42	4.0%	0.34 [0.14, 0.82]	1999
Zhang, Q.1999	0	15	0	15		Not estimable	1999
Jia, X.2000	1	15	6	15	1.4%	0.11 [0.01, 1.04]	2000
Xu, N.2000	13	34	22	30	3.5%	0.23 [0.08, 0.65]	2000
Zhang, J.2003	5	25	13	28	2.4%	0.29 [0.08, 0.99]	2003
Xu, Q.2003	3	32	20	30	4.5%	0.05 [0.01, 0.21]	2003
Wang, C.2003	4	15	0	15	0.1%	12.13 [0.59, 248.49]	2003
Li, J.2003	7	23	14	21	2.5%	0.22 [0.06, 0.78]	2003
Fang, X.2004	6	15	8	15	1.2%	0.58 [0.14, 2.48]	2004
Chen, H.2004	12	24	18	24	2.2%	0.33 [0.10, 1.13]	2004
Mao, W. 2004	6	24	10	16	2.2%	0.20 [0.05, 0.79]	2004
Huang, Y.2005	6	27	5	21	1.1%	0.91 [0.24, 3.54]	2005
Chen, J.2005	2	27	2	24	0.5%	0.88 [0.11, 6.78]	2005
Liu, M.2005	4	50	9	48	2.0%	0.38 [0.11, 1.32]	2005
Zhang, T. 2005	3	23	9	21	2.0%	0.20 [0.05, 0.89]	2005
Xiong, C.2006	4	22	6	22	1.2%	0.59 [0.14, 2.48]	2006
Zhang, Z.2006	3	16	10	16	2.0%	0.14 [0.03, 0.69]	2006
Fang, H.2006	6	24	9	22	1.7%	0.48 [0.14, 1.69]	2006
Pan, Y.2006	11	28	17	20	2.9%	0.11 [0.03, 0.48]	2006
Yue, Z.2006	3	33	9	28	2.1%	0.21 [0.05, 0.88]	2006
Zhao, Z.2006	12	32	26	32	3.9%	0.14 [0.04, 0.43]	2006
Chen, Z.2006	1	12	7	11	1.6%	0.05 [0.00, 0.57]	2006
Yin, W.2007	7	19	12	19	1.8%	0.34 [0.09, 1.27]	2007
Sun, C.2007	6	24	10	24	1.8%	0.47 [0.14, 1.60]	2007
Zheng, F.2007	7	24	5	20	0.9%	1.24 [0.32, 4.72]	2007
Zhou, F.2007	3	16	2	12	0.4%	1.15 [0.16, 8.27]	2007
Zhang, M.2008	12	33	21	34	3.2%	0.35 [0.13, 0.95]	2008
Huang, W.2008	10	28	13	28	2.0%	0.64 [0.22, 1.87]	2008
Li, X.2008	3	25	9	25	1.9%	0.24 [0.06, 1.04]	2008
Wang, H.2009	10	28	13	28	2.0%	0.64 [0.22, 1.87]	2009
Zhang, L.2009	11	34	20	35	3.2%	0.36 [0.13, 0.96]	2009
Gui, Q. 2010	4	25	15	25	3.0%	0.13 [0.03, 0.48]	2010
Qu, Y.2010	9	40	14	40	2.6%	0.54 [0.20, 1.45]	2010
Li, Z. 2010	60	230	70	230	12.5%	0.81 [0.54, 1.21]	2010
Cheng, J.2010	5	30	14	30	2.8%	0.23 [0.07, 0.76]	2010
Zhang, C. 2011	9	19	8	19	1.0%	1.24 [0.34, 4.45]	2011
Xu, Q.2012	9	27	12	27	1.9%	0.63 [0.21, 1.88]	2012
Yu, L. 2013	5	23	5	22	1.0%	0.94 [0.23, 3.85]	2013
Du, Y.2013	3	60	2	60	0.5%	1.53 [0.25, 9.48]	2013
Chen, J. 2014	6	30	9	30	1.7%	0.58 [0.18, 1.91]	2014
Yao, X.2014	1	25	1	25	0.2%	1.00 [0.06, 16.93]	2014
Zhang, J.2014	3	40	9	40	2.0%	0.28 [0.07, 1.12]	2014
Liu, J. 2015	5	30	4	29	0.8%	1.25 [0.30, 5.21]	2015
Li,D.2016	1	40	4	40	0.9%	0.23 [0.02, 2.16]	2016
Wang, Y. 2018	12	37	18	37	2.9%	0.51 [0.20, 1.30]	2018
Wu, Y. 2019	3	46	9	46	2.0%	0.29 [0.07, 1.14]	2019
**Total (95% CI)**		**1511**		**1461**	**100.0%**	**0.43 [0.36, 0.51]**	
Total events	334		555				

Heterogeneity: Chi² = 66.77, df = 45 (P = 0.02); I² = 33%
Test for overall effect: Z = 9.84 (P < 0.00001)

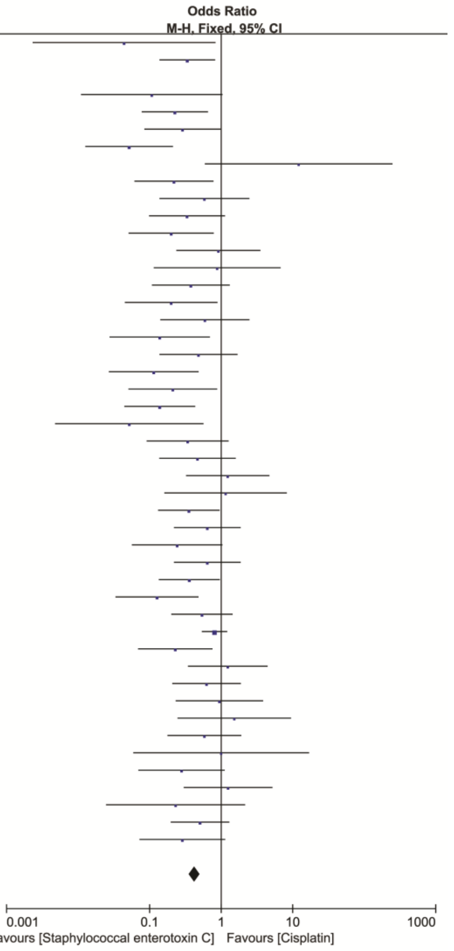

Fig.S8b The gastrointestinal reactions of staphylococcal enterotoxin C plus Cisplatin

Study or Subgroup	Staphylococcal enterotoxin C Events	Staphylococcal enterotoxin C Total	Cisplatin Events	Cisplatin Total	Weight	Odds Ratio M-H, Fixed, 95% CI	Year
**Staphylococcal enterotoxin C(SEC) vs Cisplatin(DDP)**							
Jia, X.2000	0	15	0	15		Not estimable	2000
Xue, Z.2007	0	33	1	32	16.7%	0.31 [0.01, 7.98]	2007
Zhou, F.2007	0	14	0	12		Not estimable	2007
Wang, H.2009	0	28	0	28		Not estimable	2009
Chen, J.2010	2	32	8	32	83.3%	0.20 [0.04, 1.03]	2010
Liu, J. 2015	0	25	0	29		Not estimable	2015
**Subtotal (95% CI)**		**147**		**148**	**100.0%**	**0.22 [0.05, 0.94]**	
Total events	2		9				
Heterogeneity: Chi² = 0.06, df = 1 (P = 0.81); I² = 0%							
Test for overall effect: Z = 2.04 (P = 0.04)							
**Staphylococcal enterotoxin C plus cisplatin(DDP)**							
Jia, X.2000	0	15	0	15		Not estimable	2000
Zhang, G.2000	0	36	0	30		Not estimable	2000
Wang, Lei.2002	0	21	0	19		Not estimable	2002
Feng, X.2005	0	17	0	17		Not estimable	2005
Ma, D.2005	0	24	0	24		Not estimable	2005
Chen, J.2005	1	27	0	24	1.5%	2.77 [0.11, 71.35]	2005
Liu, M.2005	0	50	0	48		Not estimable	2005
Zheng, F.2007	0	24	0	20		Not estimable	2007
Zhou, F.2007	0	16	0	12		Not estimable	2007
Huang, W.2008	0	28	0	28		Not estimable	2008
Gao, H.2009	0	35	0	30		Not estimable	2009
Wang, H.2009	0	28	0	28		Not estimable	2009
Li, Z. 2010	50	230	40	230	95.5%	1.32 [0.83, 2.10]	2010
Liu, Q.2010	0	27	0	20		Not estimable	2010
Du, Y.2013	0	60	0	60		Not estimable	2013
Yu, L. 2013	0	23	0	22		Not estimable	2013
Yao, X.2014	1	25	1	25	2.9%	1.00 [0.06, 16.93]	2014
Liu, J. 2015	0	30	0	29		Not estimable	2015
**Subtotal (95% CI)**		**716**		**681**	**100.0%**	**1.33 [0.85, 2.09]**	
Total events	52		41				
Heterogeneity: Chi² = 0.24, df = 2 (P = 0.89); I² = 0%							
Test for overall effect: Z = 1.24 (P = 0.21)							

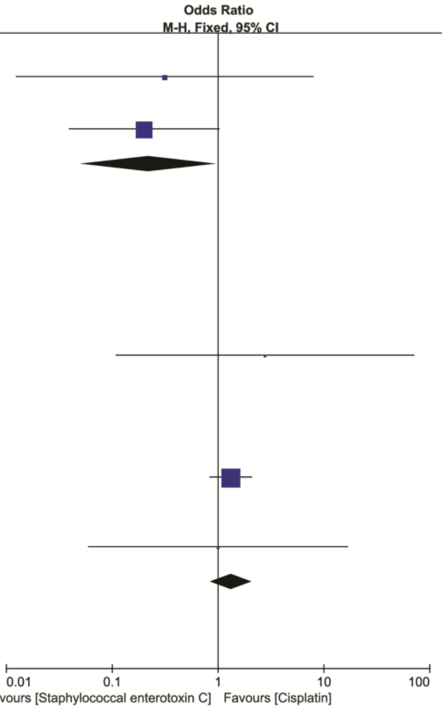

Fig.S9 The analysis of hepatic dysfunction between the two groups

Study or Subgroup	Staphylococcal enterotoxin C Events	Staphylococcal enterotoxin C Total	Cisplatin Events	Cisplatin Total	Weight	Odds Ratio M-H, Fixed, 95% CI	Year
**Staphylococcal enterotoxin C (SEC) vs Cisplatin(DDP)**							
Jia, X.2000	0	15	0	15		Not estimable	2000
Li, J.-L. 2003	0	23	0	21		Not estimable	2003b
Sun, C.2007	0	22	9	24	50.1%	0.04 [0.00, 0.67]	2007
Zhou, F.2007	0	14	0	12		Not estimable	2007
Xue, Z.2007	0	33	0	32		Not estimable	2007
Wang, H.2009	0	28	0	28		Not estimable	2009
Chen, J.2010	2	32	8	32	42.2%	0.20 [0.04, 1.03]	2010
Liu, J. 2015	0	25	1	29	7.7%	0.37 [0.01, 9.56]	2015
**Subtotal (95% CI)**		**192**		**193**	**100.0%**	**0.13 [0.04, 0.46]**	
Total events	2		18				
Heterogeneity: Chi² = 1.40, df = 2 (P = 0.50); I² = 0%							
Test for overall effect: Z = 3.17 (P = 0.002)							
**Staphylococcal enterotoxin C plus cisplatin(DDP)**							
Zhang, G.2000	0	36	0	30		Not estimable	2000
Jia, X.2000	0	15	0	15		Not estimable	2000
Wang, Lei.2002	0	21	0	19		Not estimable	2002
Chen, J.2005	1	27	2	24	12.2%	0.42 [0.04, 4.99]	2005
Feng, X.2005	0	17	0	17		Not estimable	2005
Ma, D.2005	0	24	0	24		Not estimable	2005
Liu, M.2005	0	50	0	48		Not estimable	2005
Zhang, Z.2006	0	16	1	16	8.7%	0.31 [0.01, 8.28]	2006
Sun, C.2007	2	24	9	24	49.5%	0.15 [0.03, 0.80]	2007
Zheng, F.2007	0	24	0	20		Not estimable	2007
Zhou, F.2007	0	16	0	12		Not estimable	2007
Huang, W.2008	0	28	0	28		Not estimable	2008
Gao, H.2009	0	35	0	30		Not estimable	2009
Wang, H.2009	0	28	0	28		Not estimable	2009
Liu, Q.2010	0	27	0	20		Not estimable	2010
Yu, L. 2013	0	23	1	22	9.0%	0.30 [0.01, 7.89]	2013
Yao, X.2014	1	25	2	25	11.5%	0.48 [0.04, 5.65]	2014
Liu, J. 2015	0	30	1	29	9.0%	0.31 [0.01, 7.96]	2015
**Subtotal (95% CI)**		**466**		**431**	**100.0%**	**0.26 [0.10, 0.72]**	
Total events	4		16				
Heterogeneity: Chi² = 0.82, df = 5 (P = 0.98); I² = 0%							
Test for overall effect: Z = 2.60 (P = 0.009)							

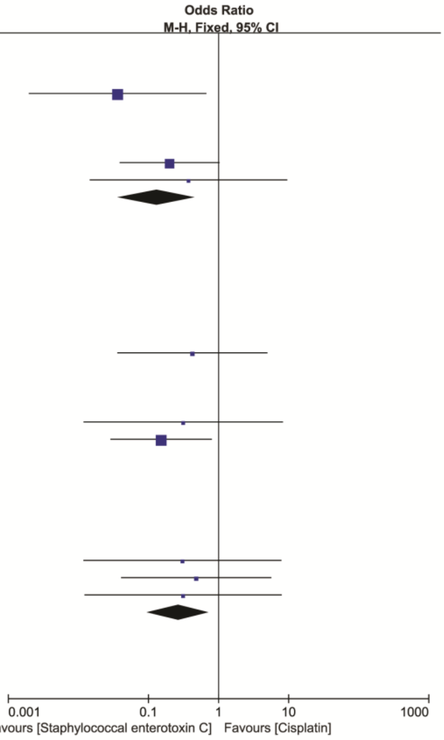

Fig.S10 The analysis of renal dysfunction between the two groups

Study or Subgroup	Staphylococcal enterotoxin C Events	Staphylococcal enterotoxin C Total	Cisplatin Events	Cisplatin Total	Weight	Odds Ratio M-H, Fixed, 95% CI
**Staphylococcal enterotoxin C (SEC) vs Cisplatin(DDP)**						
Zhou, F.2007	1	14	0	12	100.0%	2.78 [0.10, 74.70]
**Subtotal (95% CI)**		**14**		**12**	**100.0%**	**2.78 [0.10, 74.70]**
Total events	1		0			
Heterogeneity: Not applicable						
Test for overall effect: Z = 0.61 (P = 0.54)						
**Staphylococcal enterotoxin C plus cisplatin(DDP)**						
Guan, C.2004	0	23	0	22		Not estimable
Hu, Y.2004	0	23	0	22		Not estimable
Ma, D.2005	0	24	0	24		Not estimable
Wang, Lei.2002	0	21	0	19		Not estimable
Zhang, G.2000	0	36	0	30		Not estimable
Zhou, F.2007	0	16	0	12		Not estimable
**Subtotal (95% CI)**		**143**		**129**		**Not estimable**
Total events	0		0			
Heterogeneity: Not applicable						
Test for overall effect: Not applicable						

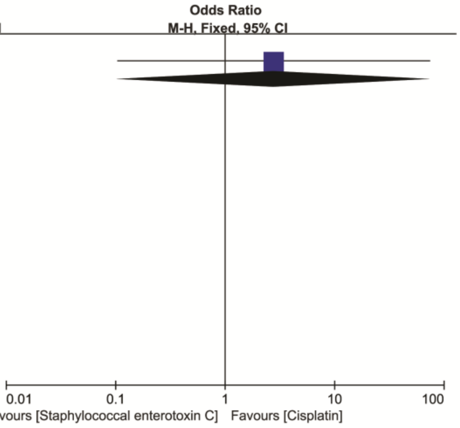

Fig.S11 The analysis of cardiac dysfunction **between the two groups**

Study or Subgroup	Staphylococcal enterotoxin C Events	Staphylococcal enterotoxin C Total	Cisplatin Events	Cisplatin Total	Weight	Odds Ratio M-H, Fixed, 95% CI	Year
**Staphylococcal enterotoxin C(SEC) vs Cisplatin(DDP)**							
Hu, H. 2000	2	13	0	13	1.5%	5.87 [0.25, 135.15]	2000
Zhou, F.2007	0	14	0	12		Not estimable	2007
Sun, C.2007	5	22	6	24	16.1%	0.88 [0.23, 3.44]	2007
Xue, Z.2007	3	33	4	32	13.4%	0.70 [0.14, 3.41]	2007
Wang, H.2009	4	28	2	28	6.2%	2.17 [0.36, 12.92]	2009
Cheng, J.2010	8	30	3	30	8.0%	3.27 [0.77, 13.83]	2010
Wu, J.2010	4	34	3	30	10.2%	1.20 [0.25, 5.85]	2010
Xu, Q.2012	3	26	2	27	6.3%	1.63 [0.25, 10.65]	2012
Chen, J. 2014	5	30	4	30	12.1%	1.30 [0.31, 5.40]	2014
Liu, J. 2015	9	25	6	29	12.9%	2.16 [0.64, 7.26]	2015
Yu, M.2017	1	20	1	20	3.4%	1.00 [0.06, 17.18]	2017
Luo, D.2018	3	30	3	30	9.8%	1.00 [0.19, 5.40]	2018
**Subtotal (95% CI)**		**305**		**305**	**100.0%**	**1.51 [0.94, 2.44]**	
Total events	47		34				
Heterogeneity: Chi² = 4.26, df = 10 (P = 0.93); I² = 0%							
Test for overall effect: Z = 1.70 (P = 0.09)							
**Staphylococcal enterotoxin C plus cisplatin(DDP)**							
Xu, Q.2003	0	32	3	30	2.6%	0.12 [0.01, 2.44]	2003
Zhang, J.2003	6	25	0	28	0.3%	19.00 [1.01, 357.09]	2003
Li, J.2003	3	23	7	21	4.6%	0.30 [0.07, 1.37]	2003
Chen, H.2004	4	24	10	24	6.1%	0.28 [0.07, 1.08]	2004
Chen, J.2005	5	27	4	24	2.5%	1.14 [0.27, 4.83]	2005
Huang, Y.2005	9	27	0	21	0.3%	22.08 [1.20, 405.72]	2005
Liu, M.2005	4	50	2	48	1.4%	2.00 [0.35, 11.46]	2005
Ma, D.2005	5	24	5	24	2.9%	1.00 [0.25, 4.03]	2005
Zhang, T. 2005	9	23	4	21	1.9%	2.73 [0.69, 10.79]	2005
Pan, Y.2006	4	28	8	20	5.8%	0.25 [0.06, 1.00]	2006
Yin, W.2007	6	19	11	19	5.5%	0.34 [0.09, 1.27]	2007
Zhou, F.2007	0	16	0	12		Not estimable	2007
Sun, C.2007	6	24	6	24	3.3%	1.00 [0.27, 3.69]	2007
Huang, W.2008	4	28	2	28	1.2%	2.17 [0.36, 12.92]	2008
Wang, H.2009	4	28	2	28	1.2%	2.17 [0.36, 12.92]	2009
Cheng, J.2010	5	30	3	30	1.8%	1.80 [0.39, 8.32]	2010
Li, Z. 2010	60	230	60	230	32.3%	1.00 [0.66, 1.52]	2010
Zhang, C. 2011	4	19	5	19	2.9%	0.75 [0.17, 3.36]	2011
Xu, Q.2012	3	27	2	27	1.3%	1.56 [0.24, 10.19]	2012
Qin, Q.2012	5	34	0	34	0.3%	12.86 [0.68, 242.49]	2012
Yu, L. 2013	4	23	3	22	1.8%	1.33 [0.26, 6.78]	2013
Du, Y.2013	2	60	0	60	0.3%	5.17 [0.24, 110.01]	2013
Li, L. 2013	7	15	0	15	0.2%	27.35 [1.39, 539.83]	2013
Zhang, J.2014	2	40	4	40	2.8%	0.47 [0.08, 2.75]	2014
Chen, J. 2014	5	30	4	30	2.4%	1.30 [0.31, 5.40]	2014
Zhou, Z.2015	10	21	0	21	0.2%	39.26 [2.10, 732.33]	2015
Liu, J. 2015	8	30	6	29	3.3%	1.39 [0.42, 4.67]	2015
Li,D.2016	1	40	2	40	1.4%	0.49 [0.04, 5.60]	2016
Yu, M.2017	2	20	1	20	0.7%	2.11 [0.18, 25.35]	2017
Wang, Y. 2018	3	37	7	37	4.7%	0.38 [0.09, 1.59]	2018
Luo, D.2018	3	30	3	30	2.0%	1.00 [0.19, 5.40]	2018
Wu, Y. 2019	2	46	3	46	2.1%	0.65 [0.10, 4.09]	2019
**Subtotal (95% CI)**		**1130**		**1102**	**100.0%**	**1.17 [0.93, 1.47]**	
Total events	195		167				
Heterogeneity: Chi² = 47.20, df = 30 (P = 0.02); I² = 36%							
Test for overall effect: Z = 1.33 (P = 0.18)							

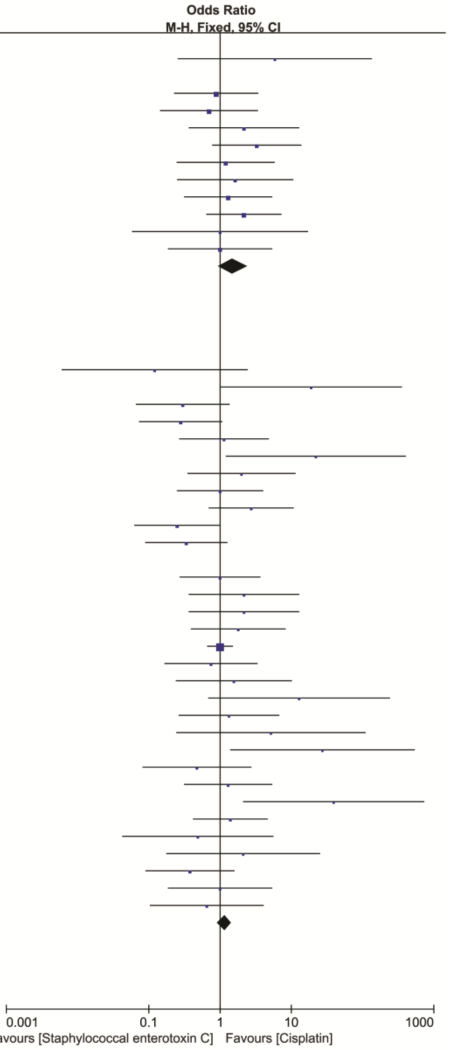

Fig.S12 The analysis of thoracodynia between the two groups

	Staphylococcal enterotoxin C		Cisplatin			Odds Ratio	
Study or Subgroup	Events	Total	Events	Total	Weight	M-H, Fixed, 95% CI	Year
**Staphylococcal enterotoxin C (SEC) vs Cisplatin(DDP)**							
Hu, H. 2000	4	13	0	13	1.9%	12.79 [0.61, 266.66]	2000
Jia, X.2000	3	15	0	15	2.2%	8.68 [0.41, 184.28]	2000
Sun, C.2007	2	22	1	24	4.8%	2.30 [0.19, 27.30]	2007
Zhou, F.2007	5	14	0	12	1.9%	14.47 [0.71, 295.24]	2007
Xue, Z.2007	25	33	4	32	5.5%	21.88 [5.87, 81.55]	2007
Zhang, M.2008	5	35	0	34	2.4%	12.44 [0.66, 234.37]	2008
Wang, H.2009	2	28	1	28	5.2%	2.08 [0.18, 24.31]	2009
Zhang, L.2009	6	36	0	35	2.3%	15.13 [0.82, 279.67]	2009
Wu, J.2010	30	34	6	30	4.2%	30.00 [7.59, 118.57]	2010
Cheng, J.2010	10	30	1	30	3.7%	14.50 [1.72, 122.40]	2010
Chen, J.2010	12	32	5	32	17.4%	3.24 [0.98, 10.68]	2010
Xu, Q.2012	2	26	1	27	5.0%	2.17 [0.18, 25.46]	2012
Chen, J. 2014	27	30	18	30	10.0%	6.00 [1.48, 24.30]	2014
Liu, J. 2015	11	25	3	29	8.6%	6.81 [1.63, 28.52]	2015
Yu, M.2017	3	20	2	20	9.4%	1.59 [0.24, 10.70]	2017
Luo, D.2018	2	30	3	30	15.6%	0.64 [0.10, 4.15]	2018
**Subtotal (95% CI)**		**423**		**421**	**100.0%**	**6.66 [4.30, 10.32]**	
Total events	149		45				
Heterogeneity: Chi² = 21.19, df = 15 (P = 0.13); I² = 29%							
Test for overall effect: Z = 8.49 (P < 0.00001)							
**Staphylococcal enterotoxin C plus cisplatin(DDP)**							
Zhang, Q.1999	0	15	0	15		Not estimable	1999
Qiu, Y.1999	7	42	1	42	0.8%	8.20 [0.96, 69.93]	1999
Zhang, G.2000	5	36	0	30	0.5%	10.65 [0.56, 200.98]	2000
Jia, X.2000	2	15	0	15	0.4%	5.74 [0.25, 130.37]	2000
Xu, N.2000	5	34	0	30	0.4%	11.37 [0.60, 214.91]	2000
Wang, Lei.2002	7	21	0	19	0.3%	20.17 [1.06, 382.45]	2002
Wang, C.2003	3	15	0	15	0.4%	8.68 [0.41, 184.28]	2003
Li, J.2003	15	23	4	21	1.4%	7.97 [1.99, 31.88]	2003
Zhang, J.2003	7	25	0	28	0.3%	23.11 [1.24, 429.27]	2003
Xu, Q.2003	10	32	7	30	4.9%	1.49 [0.48, 4.62]	2003
Chen, H.2004	2	24	6	24	5.4%	0.27 [0.05, 1.52]	2004
Mao, W. 2004	3	24	0	16	0.5%	5.37 [0.26, 111.39]	2004
Fang, X.2004	8	17	5	17	2.6%	2.13 [0.52, 8.76]	2004
Chen, J.2005	3	27	2	24	1.9%	1.38 [0.21, 9.01]	2005
Ma, D.2005	6	24	0	24	0.4%	17.22 [0.91, 325.37]	2005
Zhang, T. 2005	7	23	0	21	0.4%	19.55 [1.04, 367.40]	2005
Liu, M.2005	18	50	14	48	9.0%	1.37 [0.58, 3.19]	2005
Huang, Y.2005	14	27	0	21	0.3%	46.19 [2.54, 839.53]	2005
Pan, Y.2006	2	28	6	20	6.4%	0.18 [0.03, 1.01]	2006
Fang, H.2006	2	24	1	22	0.9%	1.91 [0.16, 22.66]	2006
Yue, Z.2006	3	33	0	28	0.5%	6.54 [0.32, 132.28]	2006
Xiong, C.2006	3	22	1	22	0.9%	3.32 [0.32, 34.65]	2006
Chen, Z.2006	3	12	2	11	1.5%	1.50 [0.20, 11.24]	2006
Zhang, Z.2006	8	16	0	16	0.2%	33.00 [1.69, 643.09]	2006
Zhao, Z.2006	9	32	3	32	2.1%	3.78 [0.92, 15.60]	2006
Yin, W.2007	13	19	15	19	4.7%	0.58 [0.13, 2.51]	2007
Sun, C.2007	2	24	1	24	0.9%	2.09 [0.18, 24.73]	2007
Zhou, F.2007	6	16	0	12	0.3%	15.48 [0.78, 308.11]	2007
Zheng, F.2007	5	24	3	20	2.6%	1.49 [0.31, 7.20]	2007
Huang, W.2008	3	28	1	28	0.9%	3.24 [0.32, 33.22]	2008
Zhang, M.2008	4	33	0	34	0.4%	10.53 [0.54, 203.69]	2008
Li, X.2008	10	25	3	25	1.8%	4.89 [1.15, 20.79]	2008
Wang, H.2009	3	28	1	28	0.9%	3.24 [0.32, 33.22]	2009
Zhang, L.2009	3	34	0	35	0.4%	7.89 [0.39, 158.73]	2009
Li, Z. 2010	20	230	20	230	18.0%	1.00 [0.52, 1.91]	2010
Cheng, J.2010	9	30	1	30	0.7%	12.43 [1.46, 105.74]	2010
Xu, Q.2012	3	27	1	27	0.9%	3.25 [0.32, 33.41]	2012
Qin, Q.2012	8	34	0	34	0.4%	22.13 [1.22, 400.93]	2012
Du, Y.2013	1	60	0	60	0.5%	3.05 [0.12, 76.39]	2013
Li, L. 2013	7	15	0	15	0.3%	27.35 [1.39, 539.83]	2013
Yu, L. 2013	11	23	3	22	1.6%	5.81 [1.34, 25.17]	2013
Chen, J. 2014	25	30	18	30	3.0%	3.33 [1.00, 11.14]	2014
Zhang, J.2014	1	40	2	40	1.9%	0.49 [0.04, 5.60]	2014
Zhou, Z.2015	10	21	0	21	0.3%	39.26 [2.10, 732.33]	2015
Liu, J. 2015	16	30	3	29	1.4%	9.90 [2.46, 39.92]	2015
Li,D.2016	1	40	4	40	3.9%	0.23 [0.02, 2.16]	2016
Yu, M.2017	4	20	2	20	1.6%	2.25 [0.36, 13.97]	2017
Wang, Y. 2018	2	37	4	37	3.7%	0.47 [0.08, 2.75]	2018
Luo, D.2018	2	30	3	30	2.8%	0.64 [0.10, 4.15]	2018
Wu, Y. 2019	2	46	4	46	3.8%	0.48 [0.08, 2.74]	2019
**Subtotal (95% CI)**		**1585**		**1527**	**100.0%**	**2.70 [2.16, 3.36]**	
Total events	323		141				
Heterogeneity: Chi² = 88.62, df = 48 (P = 0.0003); I² = 46%							
Test for overall effect: Z = 8.76 (P < 0.00001)							

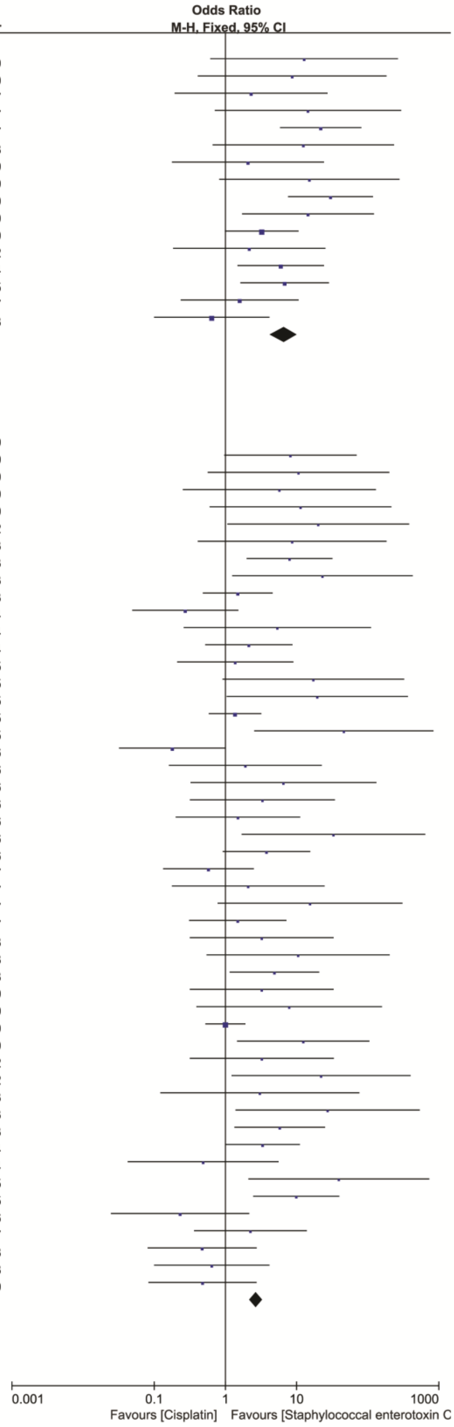

Fig.S13 The fever between the two groups
