# Supplementary material for: Intrapleural Perfusion With Staphylococcal Enterotoxin C for Malignant Pleural Effusion: A Clustered Systematic Review and Meta-Analysis
Source: Front Med (Lausanne). 2022 Apr 25;9:816973. doi: 10.3389/fmed.2022.816973 (PMC9081816; doi:10.3389/fmed.2022.816973)
Supplement: Supplementary file 5 [file Data_Sheet_5.PDF]

Appendix.5 Subgroups analysis and meta-regression (Figs.S14-65)

Study or Subgroup	Staphylococcal enterotoxin C Events	Staphylococcal enterotoxin C Total	Cisplatin Events	Cisplatin Total	Weight	Odds Ratio M-H, Fixed, 95% CI	Year
**Malignant tumors**							
Li, H.1998	18	68	6	42	1.9%	2.16 [0.78, 5.98]	1998
Qiu, Y.1999	12	42	7	42	1.7%	2.00 [0.70, 5.73]	1999
Zhang, Q.1999	10	15	4	15	0.5%	5.50 [1.15, 26.41]	1999
Cao, H.2000	6	26	1	18	0.3%	5.10 [0.56, 46.65]	2000
Jia, X.2000	6	15	2	15	0.4%	4.33 [0.71, 26.53]	2000
Xu, N.2000	10	34	8	30	2.1%	1.15 [0.38, 3.43]	2000
Zhang, G.2000	20	36	6	30	1.0%	5.00 [1.65, 15.17]	2000
Wang, L. 2002	7	29	4	29	1.0%	1.99 [0.51, 7.71]	2000
Lang, F.2001	36	56	6	21	1.1%	4.50 [1.51, 13.43]	2001
Wang, Lei.2002	11	21	7	19	1.2%	1.89 [0.53, 6.69]	2002
Duan, R.2003	27	76	16	76	3.6%	2.07 [1.00, 4.26]	2003
Wang, C.2003	5	15	2	15	0.5%	3.25 [0.52, 20.37]	2003
Xu, Q.2003	20	32	12	30	1.6%	2.50 [0.90, 6.95]	2003
Zhang, J.2003	9	25	2	28	0.4%	7.31 [1.40, 38.22]	2003
Li, J.2003	7	23	4	21	1.0%	1.86 [0.46, 7.58]	2003
Chen, H.2004	13	24	6	24	0.9%	3.55 [1.04, 12.06]	2004
Fang, X.2004	3	15	1	15	0.3%	3.50 [0.32, 38.23]	2004
Guan, C.2004	13	23	6	22	0.9%	3.47 [0.99, 12.09]	2004
Hu, Y.2004	13	23	6	22	0.9%	3.47 [0.99, 12.09]	2004
Zhu, F.2004	16	34	6	30	1.2%	3.56 [1.16, 10.89]	2004
Mao, W. 2004	8	24	2	16	0.6%	3.50 [0.63, 19.30]	2004
Liu, S.2005	13	32	6	29	1.3%	2.62 [0.84, 8.22]	2005
Ma, D.2005	13	24	6	24	0.9%	3.55 [1.04, 12.06]	2005
Feng, X.2005	6	17	4	17	0.9%	1.77 [0.40, 7.93]	2005
Chen, J.2005	11	27	5	24	1.1%	2.61 [0.75, 9.11]	2005
Xiong, C.2006	10	22	6	22	1.1%	2.22 [0.63, 7.82]	2006
Yue, Z.2006	10	33	3	28	0.8%	3.62 [0.89, 14.83]	2006
Zhang, Z.2006	6	16	2	16	0.4%	4.20 [0.70, 25.26]	2006
Zhao, Z.2006	9	32	5	32	1.2%	2.11 [0.62, 7.20]	2006
Pan, Y.2006	16	28	4	20	0.7%	5.33 [1.41, 20.10]	2006
Sun, C.2007	13	24	4	24	0.6%	5.91 [1.55, 22.58]	2007
Yin, W.2007	6	19	4	19	0.9%	1.73 [0.40, 7.51]	2007
Zhou, F.2007	5	16	0	12	0.1%	11.96 [0.59, 241.06]	2007
Zheng, F.2007	4	24	3	20	0.9%	1.13 [0.22, 5.79]	2007
Zhang, M.2008	11	33	4	34	0.9%	3.75 [1.05, 13.35]	2008
Li, X.2008	8	25	6	25	1.4%	1.49 [0.43, 5.17]	2008
Gao, H.2009	17	35	6	30	1.1%	3.78 [1.24, 11.50]	2009
Zhang, L.2009	11	34	4	35	0.9%	3.71 [1.05, 13.13]	2009
Qu, Y.2010	4	40	2	40	0.6%	2.11 [0.36, 12.24]	2010
Gui, Q. 2010	9	25	3	25	0.7%	4.13 [0.96, 17.70]	2010
Liu, Q.2010	5	27	3	20	1.0%	1.29 [0.27, 6.16]	2010
Zhang, C. 2011	13	19	4	19	0.4%	8.13 [1.87, 35.23]	2011
Qin, Q.2012	19	34	6	34	0.9%	5.91 [1.94, 17.97]	2012
Li, L. 2013	4	15	0	15	0.1%	12.13 [0.59, 248.49]	2013
Yao, X.2014	13	25	7	25	1.2%	2.79 [0.86, 9.01]	2014
Chen, J. 2014	6	30	2	30	0.6%	3.50 [0.65, 18.98]	2014
Yan, L.2016	16	33	7	28	1.3%	2.82 [0.95, 8.44]	2016
Yu, M.2017	7	20	3	20	0.7%	3.05 [0.66, 14.14]	2017
Wang, Y. 2018	20	37	17	37	2.7%	1.38 [0.55, 3.45]	2018
Liu, Y. 2019	28	50	7	50	1.1%	7.82 [2.95, 20.72]	2019
Wu, Y. 2019	15	46	7	46	1.6%	2.70 [0.98, 7.43]	2019
**Subtotal (95% CI)**		**1498**		**1360**	**51.4%**	**2.99 [2.51, 3.57]**	
Total events	598		254				
Heterogeneity: Chi² = 29.57, df = 50 (P = 0.99); I² = 0%							
Test for overall effect: Z = 12.20 (P < 0.00001)							
**Lung cancer**							
Li, S.1997	10	20	3	20	0.5%	5.67 [1.25, 25.61]	1997
Zhang, T. 2005	8	23	5	21	1.2%	1.71 [0.46, 6.39]	2005
Huang, Y.2005	9	27	4	21	1.0%	2.13 [0.55, 8.21]	2005
Liu, M.2005	35	50	14	48	1.5%	5.67 [2.38, 13.50]	2005
Chen, Z.2006	4	12	2	11	0.5%	2.25 [0.32, 15.76]	2006
Fang, H.2006	6	24	2	22	0.5%	3.33 [0.60, 18.66]	2006
Huang, W.2008	12	28	9	28	1.8%	1.58 [0.53, 4.71]	2008
Wang, H.2009	12	28	9	28	1.8%	1.58 [0.53, 4.71]	2009
Cheng, J.2010	5	30	0	30	0.1%	13.16 [0.69, 249.48]	2010
Li, Z. 2010	100	230	70	230	13.7%	1.76 [1.20, 2.58]	2010
Xing, Q.2012	7	19	3	19	0.7%	3.11 [0.66, 14.60]	2012
Xu, J.2012	14	27	9	27	1.5%	2.15 [0.72, 6.47]	2012
Xu, Q.2012	11	27	9	27	1.8%	1.38 [0.45, 4.17]	2012
Yu, D.2012	17	25	12	25	1.3%	2.30 [0.73, 7.27]	2012
Yu, L. 2013	2	23	1	22	0.3%	2.00 [0.17, 23.78]	2013
Du, Y.2013	31	60	22	60	3.7%	1.85 [0.89, 3.83]	2013
Li, W. 2013	13	30	11	30	2.2%	1.32 [0.47, 3.72]	2013
Zhang, J.2014	13	40	6	40	1.4%	2.73 [0.92, 8.13]	2014
Zhao, Y. 2014	25	40	16	40	2.1%	2.50 [1.02, 6.15]	2014
Tu, D.2014	14	38	14	38	3.1%	1.00 [0.39, 2.54]	2014
Zhang, Z.2015	28	45	17	45	2.2%	2.71 [1.16, 6.36]	2015
Zhou, Z.2015	6	21	1	21	0.2%	8.00 [0.87, 73.68]	2015
Cai,C.2015	11	21	6	21	1.0%	2.75 [0.77, 9.86]	2015
Liu, J. 2015	15	30	7	29	1.2%	3.14 [1.03, 9.55]	2015
Li,D.2016	27	40	19	40	2.1%	2.30 [0.93, 5.69]	2016
Luo, D.2018	12	30	6	30	1.2%	2.67 [0.84, 8.46]	2018
**Subtotal (95% CI)**		**988**		**973**	**48.6%**	**2.18 [1.79, 2.64]**	
Total events	447		277				
Heterogeneity: Chi² = 17.02, df = 25 (P = 0.88); I² = 0%							
Test for overall effect: Z = 7.89 (P < 0.00001)							
**Total (95% CI)**		**2486**		**2333**	**100.0%**	**2.59 [2.28, 2.95]**	
Total events	1045		531				
Heterogeneity: Chi² = 52.12, df = 76 (P = 0.98); I² = 0%							
Test for overall effect: Z = 14.40 (P < 0.00001)							
Test for subgroup differences: Chi² = 5.66, df = 1 (P = 0.02), I² = 82.3%							

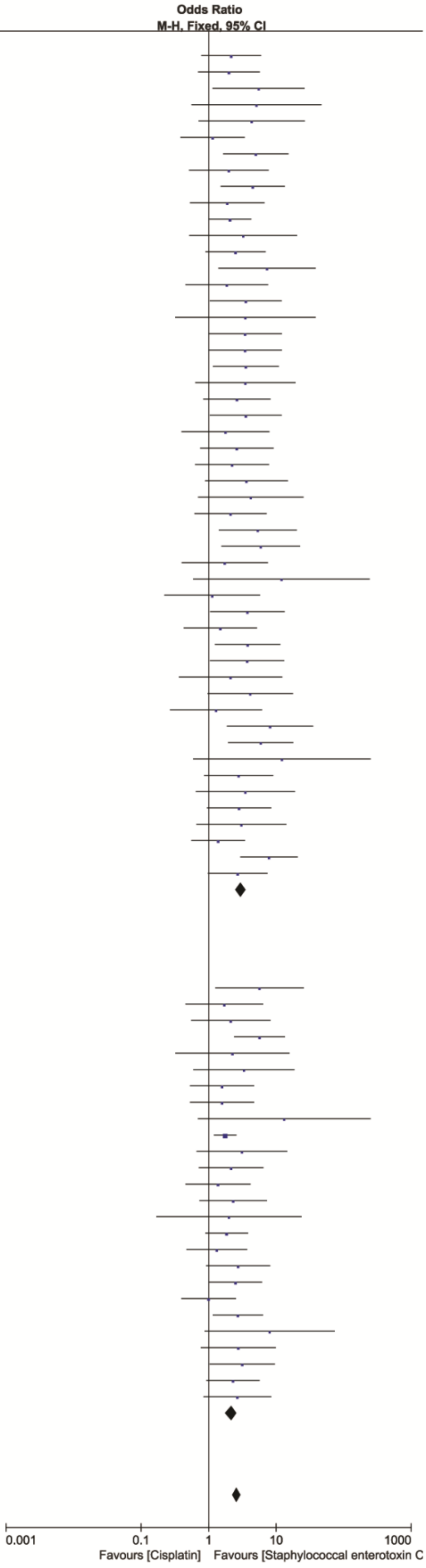

Fig.S14 Subgroups analysis of complete response via primary tumors

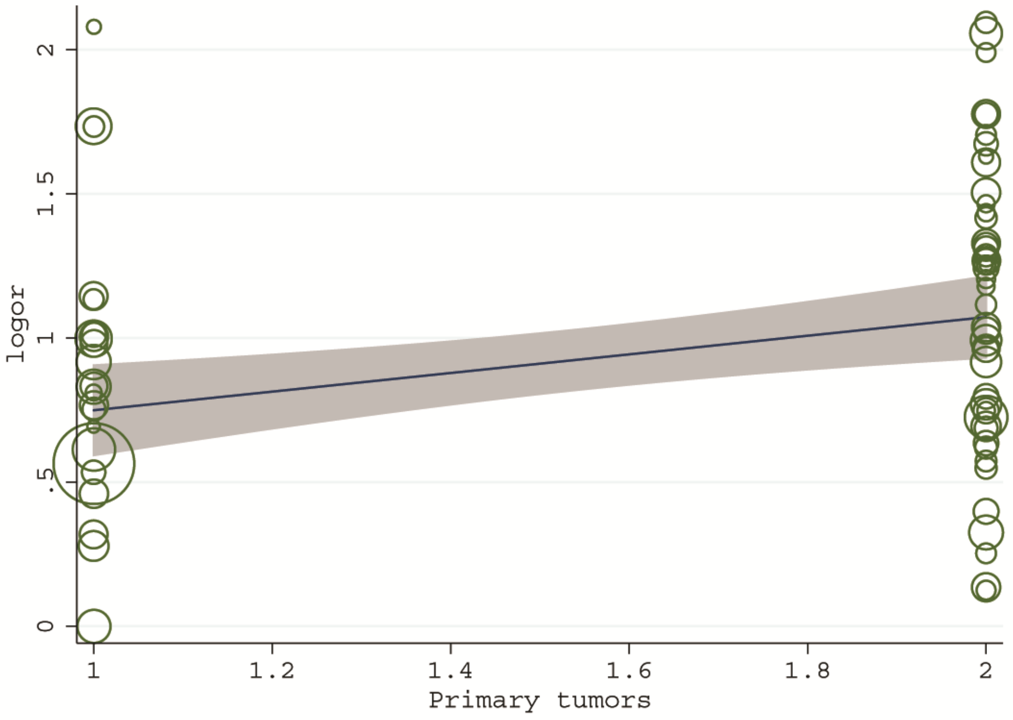

Fig.S15 Meta regression of complete response via primary tumors

Study or Subgroup	Staphylococcal enterotoxin C Events	Total	Cisplatin Events	Total	Weight	Odds Ratio M-H, Fixed, 95% CI	Year
**Malignant tumors**							
Li, H.1998	5	68	23	42	2.8%	0.07 [0.02, 0.20]	1998
Qiu, Y.1999	7	42	17	42	1.5%	0.29 [0.11, 0.81]	1999
Zhang, Q.1999	1	15	2	15	0.2%	0.46 [0.04, 5.75]	1999
Xu, N.2000	6	34	12	30	1.1%	0.32 [0.10, 1.01]	2000
Zhang, G.2000	4	36	12	30	1.2%	0.19 [0.05, 0.67]	2000
Cao, H.2000	7	26	11	18	1.0%	0.23 [0.06, 0.85]	2000
Jia, X.2000	2	15	8	15	0.7%	0.13 [0.02, 0.82]	2000
Wang, L. 2002	5	29	12	29	1.1%	0.30 [0.09, 0.99]	2000
Lang, F.2001	6	56	12	21	1.7%	0.09 [0.03, 0.30]	2001
Wang, Lei.2002	3	21	8	19	0.8%	0.23 [0.05, 1.05]	2002
Xu, Q.2003	4	32	10	30	1.0%	0.29 [0.08, 1.04]	2003
Zhang, J.2003	4	25	8	28	0.7%	0.48 [0.12, 1.83]	2003
Duan, R.2003	10	76	37	76	3.4%	0.16 [0.07, 0.36]	2003
Li, J.2003	4	23	10	21	0.9%	0.23 [0.06, 0.92]	2003
Wang, C.2003	3	15	7	15	0.6%	0.29 [0.06, 1.45]	2003
Hu, Y.2004	4	23	10	22	0.9%	0.25 [0.06, 0.99]	2004
Wang, C. 2004	5	23	12	22	1.0%	0.23 [0.06, 0.85]	2004
Zhu, F.2004	6	34	15	30	1.4%	0.21 [0.07, 0.67]	2004
Chen, H.2004	3	24	10	24	0.9%	0.20 [0.05, 0.86]	2004
Mao, W. 2004	4	24	7	16	0.7%	0.26 [0.06, 1.11]	2004
Fang, X.2004	3	15	7	15	0.6%	0.29 [0.06, 1.45]	2004
Guan, C.2004	4	23	10	22	0.9%	0.25 [0.06, 0.99]	2004
Chen, J.2005	2	27	7	24	0.7%	0.19 [0.04, 1.05]	2005
Feng, X.2005	2	17	7	17	0.7%	0.19 [0.03, 1.11]	2005
Liu, S.2005	5	32	14	29	1.3%	0.20 [0.06, 0.66]	2005
Ma, D.2005	3	24	10	24	0.9%	0.20 [0.05, 0.86]	2005
Xiong, C.2006	4	22	12	22	1.1%	0.19 [0.05, 0.73]	2006
Yue, Z.2006	5	33	13	28	1.3%	0.21 [0.06, 0.69]	2006
Zhang, Z.2006	2	16	9	16	0.8%	0.11 [0.02, 0.66]	2006
Zhao, Z.2006	5	32	14	32	1.3%	0.24 [0.07, 0.78]	2006
Pan, Y.2006	3	28	10	20	1.1%	0.12 [0.03, 0.53]	2006
Wang, J.2006	4	30	7	30	0.6%	0.51 [0.13, 1.95]	2006
Sun, C.2007	3	24	12	24	1.1%	0.14 [0.03, 0.61]	2007
Yin, W.2007	3	19	10	19	0.9%	0.17 [0.04, 0.78]	2007
Zhou, F.2007	2	16	7	12	0.7%	0.10 [0.02, 0.66]	2007
Zheng, F.2007	6	24	8	20	0.7%	0.50 [0.14, 1.81]	2007
Li, X.2008	4	25	11	25	1.0%	0.24 [0.06, 0.92]	2008
Zhang, M.2008	3	33	13	34	1.2%	0.16 [0.04, 0.64]	2008
Gao, H.2009	7	35	17	30	1.6%	0.19 [0.06, 0.57]	2009
Zhang, L.2009	3	34	12	35	1.2%	0.19 [0.05, 0.73]	2009
Liu, Q.2010	5	27	9	20	0.9%	0.28 [0.07, 1.03]	2010
Gui, Q. 2010	3	25	11	25	1.0%	0.17 [0.04, 0.73]	2010
Qu, Y.2010	8	40	19	40	1.6%	0.28 [0.10, 0.75]	2010
Zhang, C. 2011	1	19	9	19	0.9%	0.06 [0.01, 0.56]	2011
Qin, Q.2012	4	34	23	34	2.2%	0.06 [0.02, 0.23]	2012
Li, L. 2013	3	15	7	15	0.6%	0.29 [0.06, 1.45]	2013
Chen, J. 2014	5	30	15	30	1.3%	0.20 [0.06, 0.66]	2014
Yao, X.2014	2	25	12	25	1.2%	0.09 [0.02, 0.49]	2014
Yan, L.2016	4	33	8	28	0.8%	0.34 [0.09, 1.30]	2016
Yu, M.2017	2	20	13	20	1.3%	0.06 [0.01, 0.34]	2017
Wang, Y. 2018	4	37	10	37	1.0%	0.33 [0.09, 1.16]	2018
Liu, Y. 2019	2	50	23	50	2.4%	0.05 [0.01, 0.22]	2019
Wu, Y. 2019	10	46	27	46	2.3%	0.20 [0.08, 0.49]	2019
**Subtotal (95% CI)**		**1551**		**1412**	**61.0%**	**0.20 [0.16, 0.23]**	
Total events	219		639				
Heterogeneity: Chi² = 29.01, df = 52 (P = 1.00); I² = 0%							
Test for overall effect: Z = 17.84 (P < 0.00001)							
**Lung cancer**							
Li, S.1997	3	20	11	20	1.0%	0.14 [0.03, 0.65]	1997
Zhang, T. 2005	4	23	10	21	0.9%	0.23 [0.06, 0.92]	2005
Huang, Y.2005	4	27	9	21	0.9%	0.23 [0.06, 0.91]	2005
Liu, M.2005	3	50	21	48	2.2%	0.08 [0.02, 0.30]	2005
Chen, Z.2006	3	12	6	11	0.5%	0.28 [0.05, 1.62]	2006
Fang, H.2006	4	24	11	22	1.0%	0.20 [0.05, 0.78]	2006
Huang, W.2008	5	28	12	28	1.1%	0.29 [0.09, 0.98]	2008
Wang, H.2009	5	28	12	28	1.1%	0.29 [0.09, 0.98]	2009
Cheng, J.2010	6	30	15	30	1.3%	0.25 [0.08, 0.79]	2010
Li, Z. 2010	50	230	110	230	9.2%	0.30 [0.20, 0.45]	2010
Xing, Q.2012	2	19	12	19	1.1%	0.07 [0.01, 0.39]	2012
Xu, J.2012	3	27	12	27	1.1%	0.16 [0.04, 0.65]	2012
Xu, Q.2012	4	27	9	27	0.8%	0.35 [0.09, 1.31]	2012
Yu, D.2012	3	25	7	25	0.7%	0.35 [0.08, 1.55]	2012
Li, W. 2013	3	30	10	30	1.0%	0.22 [0.05, 0.91]	2013
Yu, L. 2013	4	23	11	22	1.0%	0.21 [0.05, 0.82]	2013
Du, Y.2013	6	60	25	60	2.4%	0.16 [0.06, 0.42]	2013
Zhang, J.2014	8	40	25	40	2.1%	0.15 [0.05, 0.41]	2014
Zhao, Y. 2014	2	40	13	40	1.3%	0.11 [0.02, 0.52]	2014
Tu, D.2014	4	38	13	38	1.2%	0.23 [0.07, 0.78]	2014
Zhang, Z.2015	2	45	18	45	1.8%	0.07 [0.01, 0.32]	2015
Zhou, Z.2015	4	21	11	21	1.0%	0.21 [0.05, 0.85]	2015
Cai,C.2015	3	21	7	21	0.6%	0.33 [0.07, 1.53]	2015
Liu, J. 2015	4	30	13	29	1.2%	0.19 [0.05, 0.68]	2015
Li,D.2016	1	40	10	40	1.0%	0.08 [0.01, 0.63]	2016
Luo, D.2018	3	30	14	30	1.3%	0.13 [0.03, 0.51]	2018
**Subtotal (95% CI)**		**988**		**973**	**39.0%**	**0.21 [0.17, 0.26]**	
Total events	143		427				
Heterogeneity: Chi² = 14.12, df = 25 (P = 0.96); I² = 0%							
Test for overall effect: Z = 13.84 (P < 0.00001)							
**Total (95% CI)**		**2539**		**2385**	**100.0%**	**0.20 [0.18, 0.23]**	
Total events	362		1066				
Heterogeneity: Chi² = 43.41, df = 78 (P = 1.00); I² = 0%							
Test for overall effect: Z = 22.57 (P < 0.00001)							
Test for subgroup differences: Chi² = 0.20, df = 1 (P = 0.65), I² = 0%							

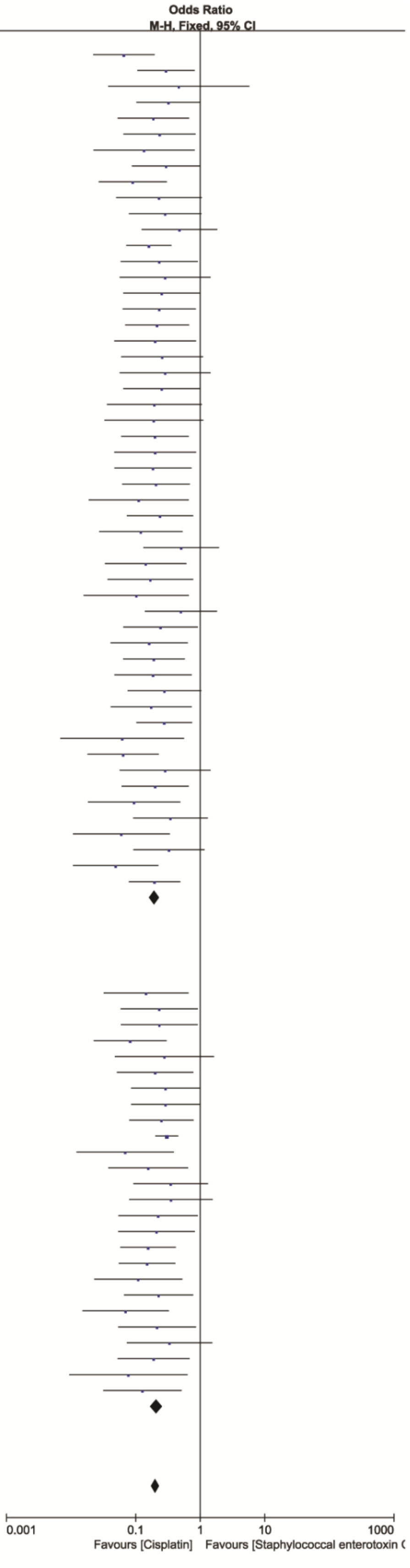

Fig.S16 Subgroups analysis of treatment failure via primary tumors

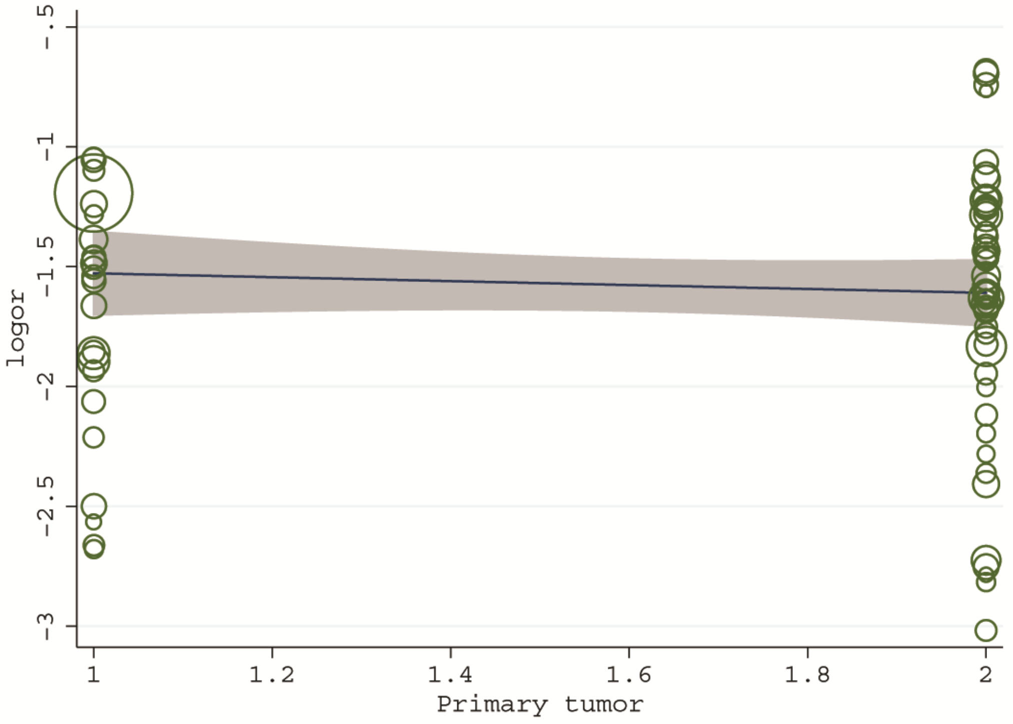

Fig.S17 Meta regression of treatment failure via primary tumors

	Staphylococcal enterotoxin C		Cisplatin			Odds Ratio	
Study or Subgroup	Events	Total	Events	Total	Weight	M-H, Fixed, 95% CI	Year
**Small to large**							
Li, S.1997	10	20	3	20	0.5%	5.67 [1.25, 25.61]	1997
Li, H.1998	18	68	6	42	1.9%	2.16 [0.78, 5.98]	1998
Liu, S.2005	13	32	6	29	1.3%	2.62 [0.84, 8.22]	2005
**Subtotal (95% CI)**		**120**		**91**	**3.7%**	**2.81 [1.43, 5.53]**	
Total events	41		15				
Heterogeneity: Chi² = 1.10, df = 2 (P = 0.58); I² = 0%							
Test for overall effect: Z = 3.00 (P = 0.003)							
**Moderate to large**							
Qiu, Y.1999	12	42	7	42	1.7%	2.00 [0.70, 5.73]	1999
Zhang, Q.1999	10	15	4	15	0.5%	5.50 [1.15, 26.41]	1999
Cao, H.2000	6	26	1	18	0.3%	5.10 [0.56, 46.65]	2000
Fang, X.2004	3	15	1	15	0.3%	3.50 [0.32, 38.23]	2004
Guan, C.2004	13	23	6	22	0.9%	3.47 [0.99, 12.09]	2004
Hu, Y.2004	13	23	6	22	0.9%	3.47 [0.99, 12.09]	2004
Chen, H.2004	13	24	6	24	0.9%	3.55 [1.04, 12.06]	2004
Liu, M.2005	35	50	14	48	1.5%	5.67 [2.38, 13.50]	2005
Pan, Y.2006	16	28	4	20	0.7%	5.33 [1.41, 20.10]	2006
Zhao, Z.2006	9	32	5	32	1.2%	2.11 [0.62, 7.20]	2006
Fang, H.2006	6	24	2	22	0.5%	3.33 [0.60, 18.66]	2006
Li, X.2008	8	25	6	25	1.4%	1.49 [0.43, 5.17]	2008
Zhang, M.2008	11	33	4	34	0.9%	3.75 [1.05, 13.35]	2008
Gao, H.2009	17	35	6	30	1.1%	3.78 [1.24, 11.50]	2009
Wang, H.2009	12	28	9	28	1.8%	1.58 [0.53, 4.71]	2009
Chen, J. 2014	6	30	2	30	0.6%	3.50 [0.65, 18.98]	2014
Liu, J. 2015	15	30	7	29	1.2%	3.14 [1.03, 9.55]	2015
**Subtotal (95% CI)**		**483**		**456**	**16.5%**	**3.22 [2.38, 4.36]**	
Total events	205		90				
Heterogeneity: Chi² = 7.34, df = 16 (P = 0.97); I² = 0%							
Test for overall effect: Z = 7.54 (P < 0.00001)							
**Large**							
Xiong, C.2006	10	22	6	22	1.1%	2.22 [0.63, 7.82]	2006
**Subtotal (95% CI)**		**22**		**22**	**1.1%**	**2.22 [0.63, 7.82]**	
Total events	10		6				
Heterogeneity: Not applicable							
Test for overall effect: Z = 1.24 (P = 0.21)							
**Unclear**							
Jia, X.2000	6	15	2	15	0.4%	4.33 [0.71, 26.53]	2000
Xu, N.2000	10	34	8	30	2.1%	1.15 [0.38, 3.43]	2000
Zhang, G.2000	20	36	6	30	1.0%	5.00 [1.65, 15.17]	2000
Wang, L. 2002	7	29	4	29	1.0%	1.99 [0.51, 7.71]	2000
Lang, F.2001	36	56	6	21	1.1%	4.50 [1.51, 13.43]	2001
Wang, Lei.2002	11	21	7	19	1.2%	1.89 [0.53, 6.69]	2002
Duan, R.2003	27	76	16	76	3.6%	2.07 [1.00, 4.26]	2003
Wang, C.2003	5	15	2	15	0.5%	3.25 [0.52, 20.37]	2003
Xu, Q.2003	20	32	12	30	1.6%	2.50 [0.90, 6.95]	2003
Zhang, J.2003	9	25	2	28	0.4%	7.31 [1.40, 38.22]	2003
Li, J.2003	7	23	4	21	1.0%	1.86 [0.46, 7.58]	2003
Zhu, F.2004	16	34	6	30	1.2%	3.56 [1.16, 10.89]	2004
Mao, W. 2004	8	24	2	16	0.6%	3.50 [0.63, 19.30]	2004
Zhang, T. 2005	8	23	5	21	1.2%	1.71 [0.46, 6.39]	2005
Feng, X.2005	6	17	4	17	0.9%	1.77 [0.40, 7.93]	2005
Chen, J.2005	11	27	5	24	1.1%	2.61 [0.75, 9.11]	2005
Huang, Y.2005	9	27	4	21	1.0%	2.13 [0.55, 8.21]	2005
Ma, D.2005	13	24	6	24	0.9%	3.55 [1.04, 12.06]	2005
Yue, Z.2006	10	33	3	28	0.8%	3.62 [0.89, 14.83]	2006
Zhang, Z.2006	6	16	2	16	0.4%	4.20 [0.70, 25.26]	2006
Chen, Z.2006	4	12	2	11	0.5%	2.25 [0.32, 15.76]	2006
Sun, C.2007	13	24	4	24	0.6%	5.91 [1.55, 22.58]	2007
Yin, W.2007	6	19	4	19	0.9%	1.73 [0.40, 7.51]	2007
Zhou, F.2007	5	16	0	12	0.1%	11.96 [0.59, 241.06]	2007
Zheng, F.2007	4	24	3	20	0.9%	1.13 [0.22, 5.79]	2007
Huang, W.2008	12	28	9	28	1.8%	1.58 [0.53, 4.71]	2008
Zhang, L.2009	11	34	4	35	0.9%	3.71 [1.05, 13.13]	2009
Qu, Y.2010	4	40	2	40	0.6%	2.11 [0.36, 12.24]	2010
Cheng, J.2010	5	30	0	30	0.1%	13.16 [0.69, 249.48]	2010
Gui, Q. 2010	9	25	3	25	0.7%	4.13 [0.96, 17.70]	2010
Li, Z. 2010	100	230	70	230	13.7%	1.76 [1.20, 2.58]	2010
Liu, Q.2010	5	27	3	20	1.0%	1.29 [0.27, 6.16]	2010
Zhang, C. 2011	13	19	4	19	0.4%	8.13 [1.87, 35.23]	2011
Xing, Q.2012	7	19	3	19	0.7%	3.11 [0.66, 14.60]	2012
Xu, J.2012	14	27	9	27	1.5%	2.15 [0.72, 6.47]	2012
Xu, Q.2012	11	27	9	27	1.8%	1.38 [0.45, 4.17]	2012
Yu, D.2012	17	25	12	25	1.3%	2.30 [0.73, 7.27]	2012
Qin, Q.2012	19	34	6	34	0.9%	5.91 [1.94, 17.97]	2012
Li, W. 2013	13	30	11	30	2.2%	1.32 [0.47, 3.72]	2013
Du, Y.2013	31	60	22	60	3.7%	1.85 [0.89, 3.83]	2013
Li, L. 2013	4	15	0	15	0.1%	12.13 [0.59, 248.49]	2013
Yu, L. 2013	2	23	1	22	0.3%	2.00 [0.17, 23.78]	2013
Zhao, Y. 2014	25	40	16	40	2.1%	2.50 [1.02, 6.15]	2014
Tu, D.2014	14	38	14	38	3.1%	1.00 [0.39, 2.54]	2014
Yao, X.2014	13	25	7	25	1.2%	2.79 [0.86, 9.01]	2014
Zhang, J.2014	13	40	6	40	1.4%	2.73 [0.92, 8.13]	2014
Zhang, Z.2015	28	45	17	45	2.2%	2.71 [1.16, 6.36]	2015
Cai,C.2015	11	21	6	21	1.0%	2.75 [0.77, 9.86]	2015
Zhou, Z.2015	6	21	1	21	0.2%	8.00 [0.87, 73.68]	2015
Yan, L.2016	16	33	7	28	1.3%	2.82 [0.95, 8.44]	2016
Li,D.2016	27	40	19	40	2.1%	2.30 [0.93, 5.69]	2016
Yu, M.2017	7	20	3	20	0.7%	3.05 [0.66, 14.14]	2017
Luo, D.2018	12	30	6	30	1.2%	2.67 [0.84, 8.46]	2018
Wang, Y. 2018	20	37	17	37	2.7%	1.38 [0.55, 3.45]	2018
Liu, Y. 2019	28	50	7	50	1.1%	7.82 [2.95, 20.72]	2019
Wu, Y. 2019	15	46	7	46	1.6%	2.70 [0.98, 7.43]	2019
**Subtotal (95% CI)**		**1861**		**1764**	**78.6%**	**2.46 [2.12, 2.85]**	
Total events	789		420				
Heterogeneity: Chi² = 40.85, df = 55 (P = 0.92); I² = 0%							
Test for overall effect: Z = 11.91 (P < 0.00001)							
**Total (95% CI)**		**2486**		**2333**	**100.0%**	**2.59 [2.28, 2.95]**	
Total events	1045		531				
Heterogeneity: Chi² = 52.12, df = 76 (P = 0.98); I² = 0%							
Test for overall effect: Z = 14.40 (P < 0.00001)							
Test for subgroup differences: Chi² = 2.55, df = 3 (P = 0.47), I² = 0%							

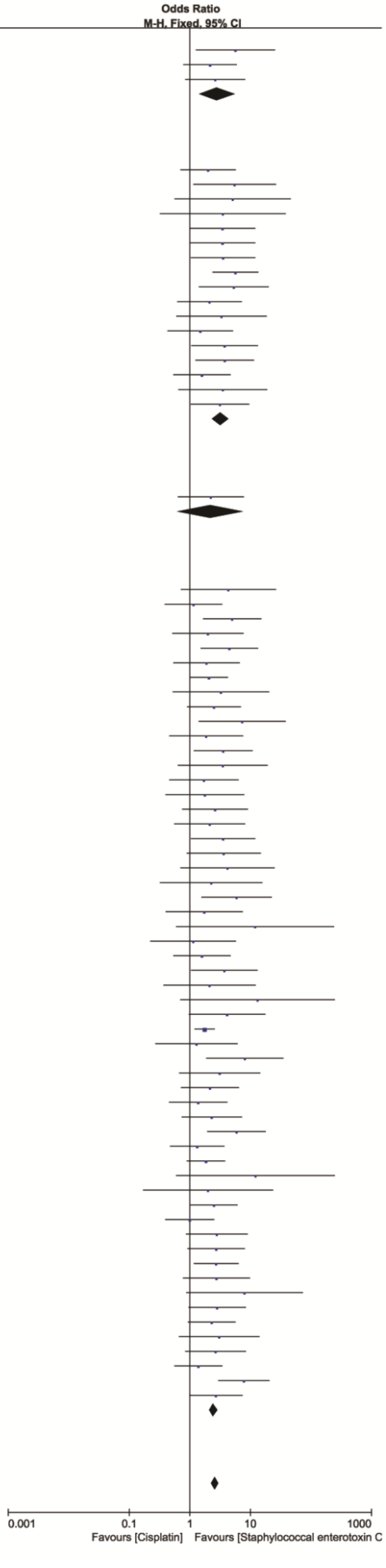

Fig.S18 Subgroups analysis of complete response via volume of pleural effusion

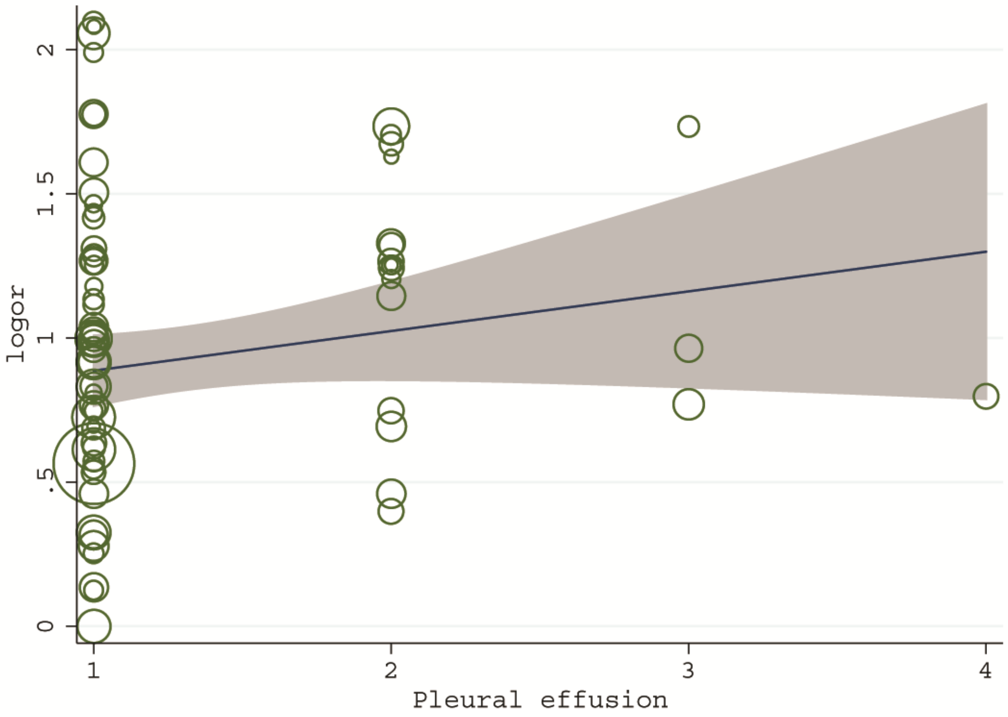

Fig.S19 Meta regression of complete response via volume of pleural effusion

Study or Subgroup	Staphylococcal enterotoxin C Events	Staphylococcal enterotoxin C Total	Cisplatin Events	Cisplatin Total	Weight	Odds Ratio M-H, Fixed, 95% CI	Year
**Small to large**							
Li, S.1997	3	20	11	20	1.0%	0.14 [0.03, 0.65]	1997
Li, H.1998	5	68	23	42	2.8%	0.07 [0.02, 0.20]	1998
Liu, S.2005	5	32	14	29	1.3%	0.20 [0.06, 0.66]	2005
**Subtotal (95% CI)**		**120**		**91**	**5.1%**	**0.12 [0.06, 0.23]**	
Total events	13		48				
Heterogeneity: Chi² = 1.89, df = 2 (P = 0.39); I² = 0%							
Test for overall effect: Z = 6.01 (P < 0.00001)							
**Moderate to large**							
Qiu, Y.1999	7	42	17	42	1.5%	0.29 [0.11, 0.81]	1999
Zhang, Q.1999	1	15	2	15	0.2%	0.46 [0.04, 5.75]	1999
Cao, H.2000	7	26	11	18	1.0%	0.23 [0.06, 0.85]	2000
Fang, X.2004	3	15	7	15	0.6%	0.29 [0.06, 1.45]	2004
Guan, C.2004	4	23	10	22	0.9%	0.25 [0.06, 0.99]	2004
Hu, Y.2004	4	23	10	22	0.9%	0.25 [0.06, 0.99]	2004
Wang, C. 2004	5	23	12	22	1.0%	0.23 [0.06, 0.85]	2004
Chen, H.2004	3	24	10	24	0.9%	0.20 [0.05, 0.86]	2004
Liu, M.2005	3	50	21	48	2.2%	0.08 [0.02, 0.30]	2005
Zhao, Z.2006	5	32	14	32	1.3%	0.24 [0.07, 0.78]	2006
Fang, H.2006	4	24	11	22	1.0%	0.20 [0.05, 0.78]	2006
Pan, Y.2006	3	28	10	20	1.1%	0.12 [0.03, 0.53]	2006
Wang, J.2006	4	30	7	30	0.6%	0.51 [0.13, 1.95]	2006
Li, X.2008	4	25	11	25	1.0%	0.24 [0.06, 0.92]	2008
Zhang, M.2008	3	33	13	34	1.2%	0.16 [0.04, 0.64]	2008
Gao, H.2009	7	35	17	30	1.6%	0.19 [0.06, 0.57]	2009
Wang, H.2009	5	28	12	28	1.1%	0.29 [0.09, 0.98]	2009
Chen, J. 2014	5	30	15	30	1.3%	0.20 [0.06, 0.66]	2014
Liu, J. 2015	4	30	13	29	1.2%	0.19 [0.05, 0.68]	2015
**Subtotal (95% CI)**		**536**		**508**	**20.7%**	**0.22 [0.16, 0.29]**	
Total events	81		223				
Heterogeneity: Chi² = 5.77, df = 18 (P = 1.00); I² = 0%							
Test for overall effect: Z = 9.99 (P < 0.00001)							
**Large**							
Xiong, C.2006	4	22	12	22	1.1%	0.19 [0.05, 0.73]	2006
**Subtotal (95% CI)**		**22**		**22**	**1.1%**	**0.19 [0.05, 0.73]**	
Total events	4		12				
Heterogeneity: Not applicable							
Test for overall effect: Z = 2.41 (P = 0.02)							
**Unclear**							
Xu, N.2000	6	34	12	30	1.1%	0.32 [0.10, 1.01]	2000
Zhang, G.2000	4	36	12	30	1.2%	0.19 [0.05, 0.67]	2000
Jia, X.2000	2	15	8	15	0.7%	0.13 [0.02, 0.82]	2000
Wang, L. 2002	5	29	12	29	1.1%	0.30 [0.09, 0.99]	2000
Lang, F.2001	6	56	12	21	1.7%	0.09 [0.03, 0.30]	2001
Wang, Lei.2002	3	21	8	19	0.8%	0.23 [0.05, 1.05]	2002
Xu, Q.2003	4	32	10	30	1.0%	0.29 [0.08, 1.04]	2003
Zhang, J.2003	4	25	8	28	0.7%	0.48 [0.12, 1.83]	2003
Duan, R.2003	10	76	37	76	3.4%	0.16 [0.07, 0.36]	2003
Li, J.2003	4	23	10	21	0.9%	0.23 [0.06, 0.92]	2003
Wang, C.2003	3	15	7	15	0.6%	0.29 [0.06, 1.45]	2003
Zhu, F.2004	6	34	15	30	1.4%	0.21 [0.07, 0.67]	2004
Mao, W. 2004	4	24	7	16	0.7%	0.26 [0.06, 1.11]	2004
Zhang, T. 2005	4	23	10	21	0.9%	0.23 [0.06, 0.92]	2005
Chen, J.2005	2	27	7	24	0.7%	0.19 [0.04, 1.05]	2005
Feng, X.2005	2	17	7	17	0.7%	0.19 [0.03, 1.11]	2005
Huang, Y.2005	4	27	9	21	0.9%	0.23 [0.06, 0.91]	2005
Ma, D.2005	3	24	10	24	0.9%	0.20 [0.05, 0.86]	2005
Yue, Z.2006	5	33	13	28	1.3%	0.21 [0.06, 0.69]	2006
Zhang, Z.2006	2	16	9	16	0.8%	0.11 [0.02, 0.66]	2006
Chen, Z.2006	3	12	6	11	0.5%	0.28 [0.05, 1.62]	2006
Sun, C.2007	3	24	12	24	1.1%	0.14 [0.03, 0.61]	2007
Yin, W.2007	3	19	10	19	0.9%	0.17 [0.04, 0.78]	2007
Zhou, F.2007	2	16	7	12	0.7%	0.10 [0.02, 0.66]	2007
Zheng, F.2007	6	24	8	20	0.7%	0.50 [0.14, 1.81]	2007
Huang, W.2008	5	28	12	28	1.1%	0.29 [0.09, 0.98]	2008
Zhang, L.2009	3	34	12	35	1.2%	0.19 [0.05, 0.73]	2009
Liu, Q.2010	5	27	9	20	0.9%	0.28 [0.07, 1.03]	2010
Gui, Q. 2010	3	25	11	25	1.0%	0.17 [0.04, 0.73]	2010
Cheng, J.2010	6	30	15	30	1.3%	0.25 [0.08, 0.79]	2010
Qu, Y.2010	8	40	19	40	1.6%	0.28 [0.10, 0.75]	2010
Li, Z. 2010	50	230	110	230	9.2%	0.30 [0.20, 0.45]	2010
Zhang, C. 2011	1	19	9	19	0.9%	0.06 [0.01, 0.56]	2011
Xu, J.2012	3	27	12	27	1.1%	0.16 [0.04, 0.65]	2012
Xu, Q.2012	4	27	9	27	0.8%	0.35 [0.09, 1.31]	2012
Yu, D.2012	3	25	7	25	0.7%	0.35 [0.08, 1.55]	2012
Qin, Q.2012	4	34	23	34	2.2%	0.06 [0.02, 0.23]	2012
Xing, Q.2012	2	19	12	19	1.1%	0.07 [0.01, 0.39]	2012
Du, Y.2013	6	60	25	60	2.4%	0.16 [0.06, 0.42]	2013
Li, L. 2013	3	15	7	15	0.6%	0.29 [0.06, 1.45]	2013
Li, W. 2013	3	30	10	30	1.0%	0.22 [0.05, 0.91]	2013
Yu, L. 2013	4	23	11	22	1.0%	0.21 [0.05, 0.82]	2013
Zhao, Y. 2014	2	40	13	40	1.3%	0.11 [0.02, 0.52]	2014
Zhang, J.2014	8	40	25	40	2.1%	0.15 [0.05, 0.41]	2014
Tu, D.2014	4	38	13	38	1.2%	0.23 [0.07, 0.78]	2014
Yao, X.2014	2	25	12	25	1.2%	0.09 [0.02, 0.49]	2014
Zhou, Z.2015	4	21	11	21	1.0%	0.21 [0.05, 0.85]	2015
Cai,C.2015	3	21	7	21	0.6%	0.33 [0.07, 1.53]	2015
Zhang, Z.2015	2	45	18	45	1.8%	0.07 [0.01, 0.32]	2015
Li,D.2016	1	40	10	40	1.0%	0.08 [0.01, 0.63]	2016
Yan, L.2016	4	33	8	28	0.8%	0.34 [0.09, 1.30]	2016
Yu, M.2017	2	20	13	20	1.3%	0.06 [0.01, 0.34]	2017
Luo, D.2018	3	30	14	30	1.3%	0.13 [0.03, 0.51]	2018
Wang, Y. 2018	4	37	10	37	1.0%	0.33 [0.09, 1.16]	2018
Liu, Y. 2019	2	50	23	50	2.4%	0.05 [0.01, 0.22]	2019
Wu, Y. 2019	10	46	27	46	2.3%	0.20 [0.08, 0.49]	2019
**Subtotal (95% CI)**		**1861**		**1764**	**73.1%**	**0.20 [0.17, 0.24]**	
Total events	264		783				
Heterogeneity: Chi² = 32.96, df = 55 (P = 0.99); I² = 0%							
Test for overall effect: Z = 19.22 (P < 0.00001)							
**Total (95% CI)**		**2539**		**2385**	**100.0%**	**0.20 [0.18, 0.23]**	
Total events	362		1066				
Heterogeneity: Chi² = 43.41, df = 78 (P = 1.00); I² = 0%							
Test for overall effect: Z = 22.57 (P < 0.00001)							
Test for subgroup differences: Chi² = 2.71, df = 3 (P = 0.44), I² = 0%							

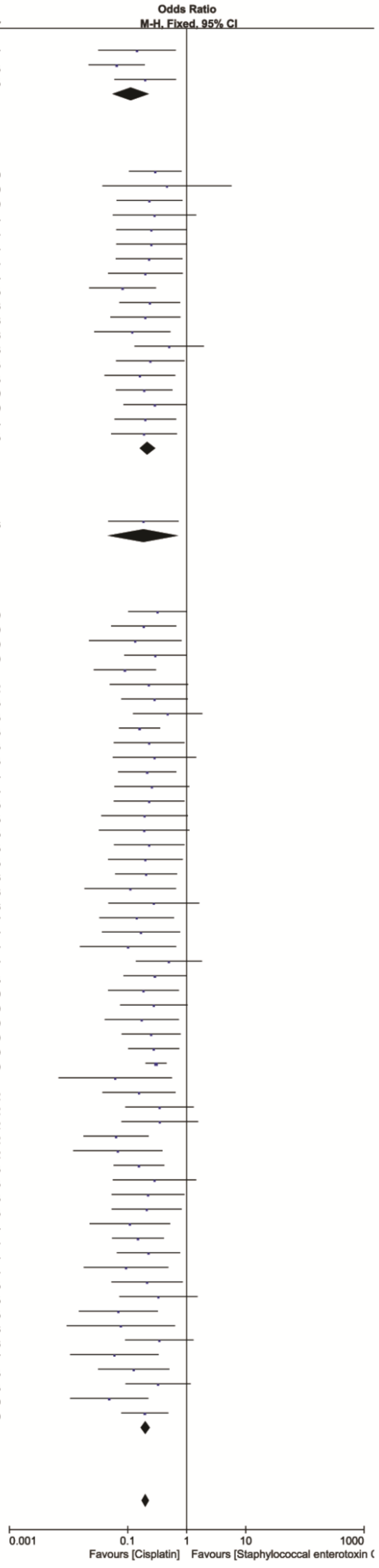

Fig.S20 Subgroups analysis of treatment failure via volume of pleural effusion

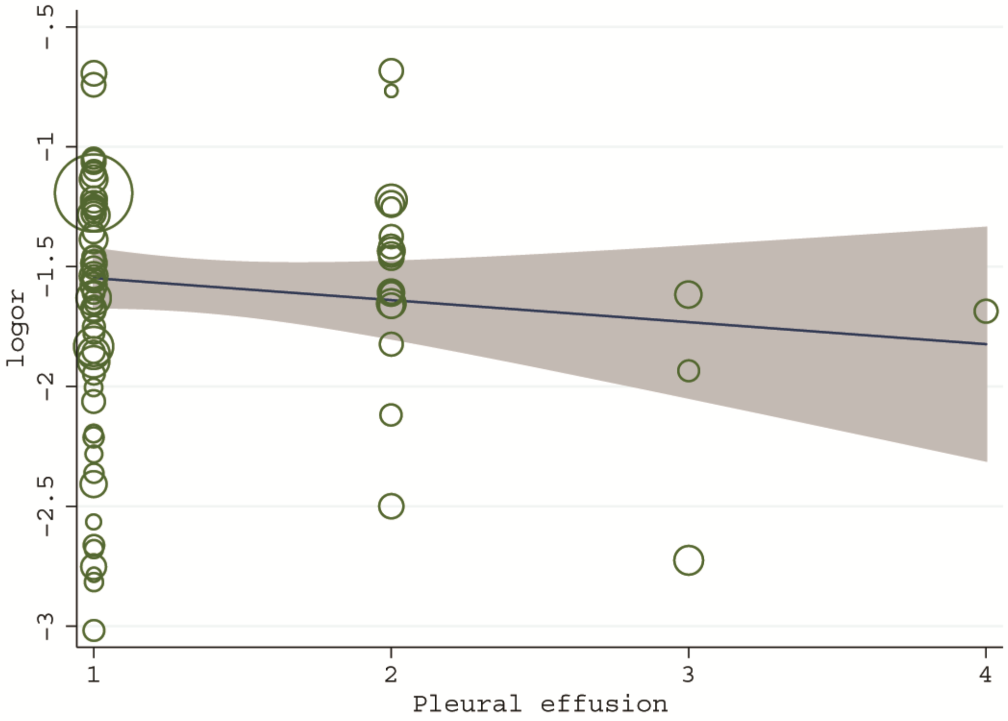

Fig.S21 Meta regression of treatment failure via volume of pleural effusion

Study or Subgroup	Staphylococcal enterotoxin C Events	Staphylococcal enterotoxin C Total	Cisplatin Events	Cisplatin Total	Weight	Odds Ratio M-H, Fixed, 95% CI	Year
**Karnofsky Performance Status score (⩾ 40)**							
Li, S.1997	10	20	3	20	0.5%	5.67 [1.25, 25.61]	1997
Qiu, Y.1999	12	42	7	42	1.7%	2.00 [0.70, 5.73]	1999
Lang, F.2001	36	56	6	21	1.1%	4.50 [1.51, 13.43]	2001
Wang, Lei.2002	11	21	7	19	1.2%	1.89 [0.53, 6.69]	2002
Xiong, C.2006	10	22	6	22	1.1%	2.22 [0.63, 7.82]	2006
Zhang, Z.2006	6	16	2	16	0.4%	4.20 [0.70, 25.26]	2006
Qin, Q.2012	19	34	6	34	0.9%	5.91 [1.94, 17.97]	2012
**Subtotal (95% CI)**		**211**		**174**	**7.0%**	**3.32 [2.09, 5.27]**	
Total events	104		37				
Heterogeneity: Chi² = 3.93, df = 6 (P = 0.69); I² = 0%							
Test for overall effect: Z = 5.09 (P < 0.00001)							
**Karnofsky Performance Status score (⩾ 50)**							
Zhang, Q.1999	10	15	4	15	0.5%	5.50 [1.15, 26.41]	1999
Jia, X.2000	6	26	1	18	0.3%	5.10 [0.56, 46.65]	2000
Cao, H.2000	6	15	2	15	0.4%	4.33 [0.71, 26.53]	2000
Zhang, J.2003	9	25	2	28	0.4%	7.31 [1.40, 38.22]	2003
Liu, S.2005	13	32	6	29	1.3%	2.62 [0.84, 8.22]	2005
Ma, D.2005	13	24	6	24	0.9%	3.55 [1.04, 12.06]	2005
Fang, H.2006	6	24	2	22	0.5%	3.33 [0.60, 18.66]	2006
Zhang, M.2008	11	33	4	34	0.9%	3.75 [1.05, 13.35]	2008
Huang, W.2008	12	28	9	28	1.8%	1.58 [0.53, 4.71]	2008
Wang, H.2009	12	28	9	28	1.8%	1.58 [0.53, 4.71]	2009
Zhang, L.2009	11	34	4	35	0.9%	3.71 [1.05, 13.13]	2009
Gui, Q. 2010	9	25	3	25	0.7%	4.13 [0.96, 17.70]	2010
Li, Z. 2010	100	230	70	230	13.7%	1.76 [1.20, 2.58]	2010
Liu, Q.2010	5	27	3	20	1.0%	1.29 [0.27, 6.16]	2010
Xing, Q.2012	7	19	3	19	0.7%	3.11 [0.66, 14.60]	2012
Xu, J.2012	14	27	9	27	1.5%	2.15 [0.72, 6.47]	2012
Xu, Q.2012	11	27	9	27	1.8%	1.38 [0.45, 4.17]	2012
Li, W. 2013	13	30	11	30	2.2%	1.32 [0.47, 3.72]	2013
Tu, D.2014	14	38	14	38	3.1%	1.00 [0.39, 2.54]	2014
Zhao, Y. 2014	25	40	16	40	2.1%	2.50 [1.02, 6.15]	2014
**Subtotal (95% CI)**		**747**		**732**	**36.3%**	**2.12 [1.69, 2.65]**	
Total events	307		187				
Heterogeneity: Chi² = 14.28, df = 19 (P = 0.77); I² = 0%							
Test for overall effect: Z = 6.51 (P < 0.00001)							
**Karnofsky Performance Status score (⩾ 60)**							
Duan, R.2003	27	76	16	76	3.6%	2.07 [1.00, 4.26]	2003
Guan, C.2004	13	23	6	22	0.9%	3.47 [0.99, 12.09]	2004
Hu, Y.2004	13	23	6	22	0.9%	3.47 [0.99, 12.09]	2004
Fang, X.2004	3	15	1	15	0.3%	3.50 [0.32, 38.23]	2004
Feng, X.2005	6	17	4	17	0.9%	1.77 [0.40, 7.93]	2005
Liu, M.2005	35	50	14	48	1.5%	5.67 [2.38, 13.50]	2005
Chen, J.2005	11	27	5	24	1.1%	2.61 [0.75, 9.11]	2005
Yue, Z.2006	10	33	3	28	0.8%	3.62 [0.89, 14.83]	2006
Zhou, F.2007	5	16	0	12	0.1%	11.96 [0.59, 241.06]	2007
Gao, H.2009	17	35	6	30	1.1%	3.78 [1.24, 11.50]	2009
Qu, Y.2010	4	40	2	40	0.6%	2.11 [0.36, 12.24]	2010
Cheng, J.2010	5	30	0	30	0.1%	13.16 [0.69, 249.48]	2010
Li, L. 2013	4	15	0	15	0.1%	12.13 [0.59, 248.49]	2013
Yu, L. 2013	2	23	1	22	0.3%	2.00 [0.17, 23.78]	2013
Zhang, J.2014	13	40	6	40	1.4%	2.73 [0.92, 8.13]	2014
Liu, J. 2015	15	30	7	29	1.2%	3.14 [1.03, 9.55]	2015
Yu, M.2017	7	20	3	20	0.7%	3.05 [0.66, 14.14]	2017
Luo, D.2018	12	30	6	30	1.2%	2.67 [0.84, 8.46]	2018
**Subtotal (95% CI)**		**543**		**520**	**16.9%**	**3.22 [2.38, 4.36]**	
Total events	202		86				
Heterogeneity: Chi² = 6.84, df = 17 (P = 0.99); I² = 0%							
Test for overall effect: Z = 7.61 (P < 0.00001)							
**Karnofsky Performance Status score(Unclear)**							
Li, H.1998	18	68	6	42	1.9%	2.16 [0.78, 5.98]	1998
Xu, N.2000	10	34	8	30	2.1%	1.15 [0.38, 3.43]	2000
Zhang, G.2000	20	36	6	30	1.0%	5.00 [1.65, 15.17]	2000
Wang, L. 2002	7	29	4	29	1.0%	1.99 [0.51, 7.71]	2000
Wang, C.2003	5	15	2	15	0.5%	3.25 [0.52, 20.37]	2003
Xu, Q.2003	20	32	12	30	1.6%	2.50 [0.90, 6.95]	2003
Li, J.2003	7	23	4	21	1.0%	1.86 [0.46, 7.58]	2003
Chen, H.2004	13	24	6	24	0.9%	3.55 [1.04, 12.06]	2004
Zhu, F.2004	16	34	6	30	1.2%	3.56 [1.16, 10.89]	2004
Mao, W. 2004	8	24	2	16	0.6%	3.50 [0.63, 19.30]	2004
Huang, Y.2005	9	27	4	21	1.0%	2.13 [0.55, 8.21]	2005
Zhang, T. 2005	8	23	5	21	1.2%	1.71 [0.46, 6.39]	2005
Zhao, Z.2006	9	32	5	32	1.2%	2.11 [0.62, 7.20]	2006
Pan, Y.2006	16	28	4	20	0.7%	5.33 [1.41, 20.10]	2006
Chen, Z.2006	4	12	2	11	0.5%	2.25 [0.32, 15.76]	2006
Sun, C.2007	13	24	4	24	0.6%	5.91 [1.55, 22.58]	2007
Yin, W.2007	6	19	4	19	0.9%	1.73 [0.40, 7.51]	2007
Zheng, F.2007	4	24	3	20	0.9%	1.13 [0.22, 5.79]	2007
Li, X.2008	8	25	6	25	1.4%	1.49 [0.43, 5.17]	2008
Zhang, C. 2011	13	19	4	19	0.4%	8.13 [1.87, 35.23]	2011
Yu, D.2012	17	25	12	25	1.3%	2.30 [0.73, 7.27]	2012
Du, Y.2013	31	60	22	60	3.7%	1.85 [0.89, 3.83]	2013
Yao, X.2014	13	25	7	25	1.2%	2.79 [0.86, 9.01]	2014
Chen, J. 2014	6	30	2	30	0.6%	3.50 [0.65, 18.98]	2014
Zhang, Z.2015	28	45	17	45	2.2%	2.71 [1.16, 6.36]	2015
Cai,C.2015	11	21	6	21	1.0%	2.75 [0.77, 9.86]	2015
Zhou, Z.2015	6	21	1	21	0.2%	8.00 [0.87, 73.68]	2015
Yan, L.2016	16	33	7	28	1.3%	2.82 [0.95, 8.44]	2016
Li,D.2016	27	40	19	40	2.1%	2.30 [0.93, 5.69]	2016
Wang, Y. 2018	20	37	17	37	2.7%	1.38 [0.55, 3.45]	2018
Liu, Y. 2019	28	50	7	50	1.1%	7.82 [2.95, 20.72]	2019
Wu, Y. 2019	15	46	7	46	1.6%	2.70 [0.98, 7.43]	2019
**Subtotal (95% CI)**		**985**		**907**	**39.7%**	**2.64 [2.15, 3.24]**	
Total events	432		221				
Heterogeneity: Chi² = 21.12, df = 31 (P = 0.91); I² = 0%							
Test for overall effect: Z = 9.27 (P < 0.00001)							
**Total (95% CI)**		**2486**		**2333**	**100.0%**	**2.59 [2.28, 2.95]**	
Total events	1045		531				
Heterogeneity: Chi² = 52.12, df = 76 (P = 0.98); I² = 0%							
Test for overall effect: Z = 14.40 (P < 0.00001)							
Test for subgroup differences: Chi² = 6.25, df = 3 (P = 0.10), I² = 52.0%							

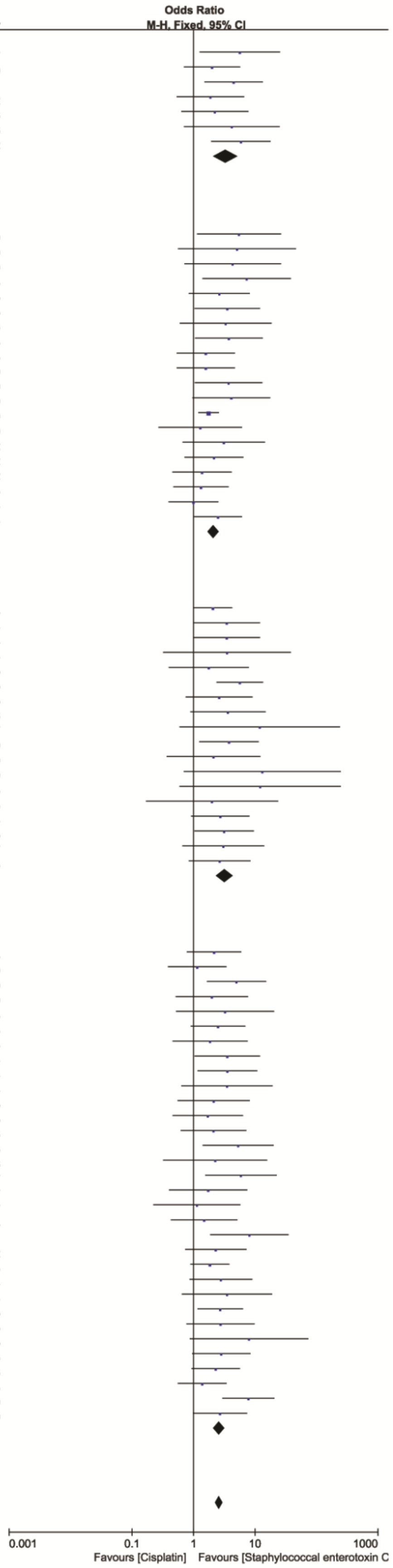

Fig.S22 Subgroups analysis of complete response via KPS score

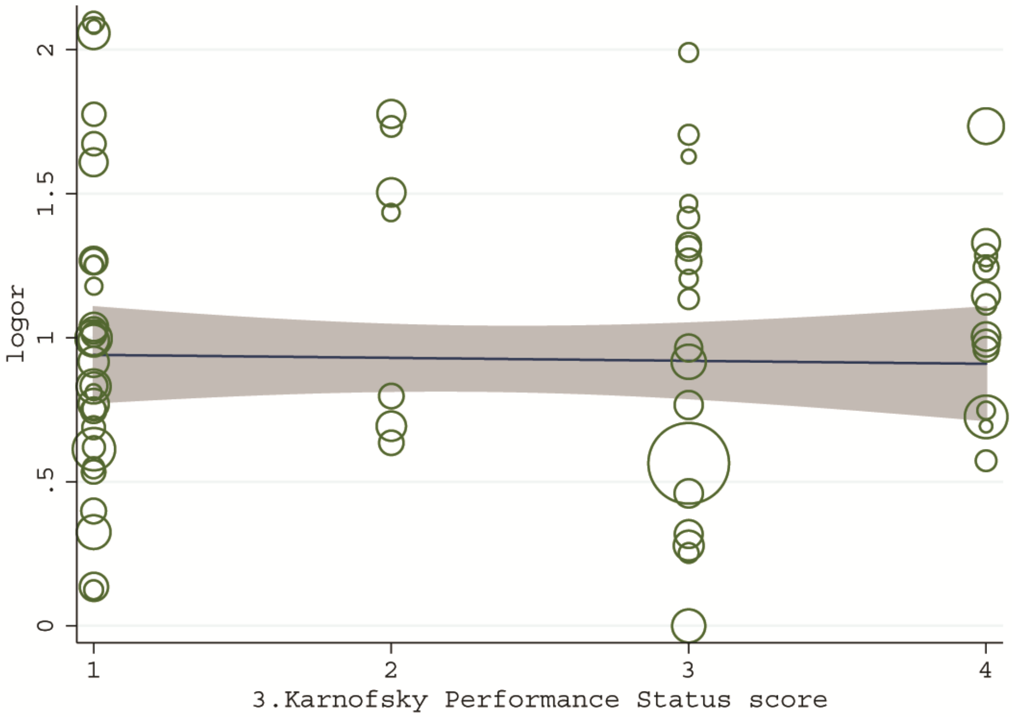

Fig.S23 Meta regression of complete response via KPS score

Study or Subgroup	Staphylococcal enterotoxin C Events	Staphylococcal enterotoxin C Total	Cisplatin Events	Cisplatin Total	Weight	Odds Ratio M-H, Fixed, 95% CI	Year
**Karnofsky Performance Status score (≥ 40)**							
Li, S.1997	3	20	11	20	1.0%	0.14 [0.03, 0.65]	1997
Qiu, Y.1999	7	42	17	42	1.5%	0.29 [0.11, 0.81]	1999
Lang, F.2001	6	56	12	21	1.7%	0.09 [0.03, 0.30]	2001
Wang, Lei.2002	3	21	8	19	0.8%	0.23 [0.05, 1.05]	2002
Xiong, C.2006	4	22	12	22	1.1%	0.19 [0.05, 0.73]	2006
Zhang, Z.2006	2	16	9	16	0.8%	0.11 [0.02, 0.66]	2006
Qin, Q.2012	4	34	23	34	2.2%	0.06 [0.02, 0.23]	2012
**Subtotal (95% CI)**		**211**		**174**	**9.0%**	**0.15 [0.09, 0.24]**	
Total events	29		92				
Heterogeneity: Chi^2 = 4.61, df = 6 (P = 0.59); I^2 = 0%							
Test for overall effect: Z = 7.53 (P < 0.00001)							
**Karnofsky Performance Status score (≥ 50)**							
Zhang, Q.1999	1	15	2	15	0.2%	0.46 [0.04, 5.75]	1999
Cao, H.2000	7	26	11	18	1.0%	0.23 [0.06, 0.85]	2000
Jia, X.2000	2	15	8	15	0.7%	0.13 [0.02, 0.82]	2000
Zhang, J.2003	4	25	8	28	0.7%	0.48 [0.12, 1.83]	2003
Wang, C. 2004	5	23	12	22	1.0%	0.23 [0.06, 0.85]	2004
Liu, S.2005	5	32	14	29	1.3%	0.20 [0.06, 0.66]	2005
Ma, D.2005	3	24	10	24	0.9%	0.20 [0.05, 0.86]	2005
Wang, J.2006	4	30	7	30	0.6%	0.51 [0.13, 1.95]	2006
Fang, H.2006	4	24	11	22	1.0%	0.20 [0.05, 0.78]	2006
Huang, W.2008	5	28	12	28	1.1%	0.29 [0.09, 0.98]	2008
Zhang, M.2008	3	33	13	34	1.2%	0.16 [0.04, 0.64]	2008
Zhang, L.2009	3	34	12	35	1.2%	0.19 [0.05, 0.73]	2009
Wang, H.2009	5	28	12	28	1.1%	0.29 [0.09, 0.98]	2009
Gui, Q. 2010	3	25	11	25	1.0%	0.17 [0.04, 0.73]	2010
Li, Z. 2010	50	230	110	230	9.2%	0.30 [0.20, 0.45]	2010
Liu, Q.2010	5	27	9	20	0.9%	0.28 [0.07, 1.03]	2010
Xing, Q.2012	2	19	12	19	1.1%	0.07 [0.01, 0.39]	2012
Xu, J.2012	3	27	12	27	1.1%	0.16 [0.04, 0.65]	2012
Xu, Q.2012	4	27	9	27	0.8%	0.35 [0.09, 1.31]	2012
Li, W. 2013	3	30	10	30	1.0%	0.22 [0.05, 0.91]	2013
Zhao, Y. 2014	2	40	13	40	1.3%	0.11 [0.02, 0.52]	2014
Tu, D.2014	4	38	13	38	1.2%	0.23 [0.07, 0.78]	2014
**Subtotal (95% CI)**		**800**		**784**	**29.9%**	**0.25 [0.19, 0.32]**	
Total events	127		331				
Heterogeneity: Chi^2 = 8.70, df = 21 (P = 0.99); I^2 = 0%							
Test for overall effect: Z = 11.37 (P < 0.00001)							
**Karnofsky Performance Status score (≥ 60)**							
Duan, R.2003	10	76	37	76	3.4%	0.16 [0.07, 0.36]	2003
Guan, C.2004	4	23	10	22	0.9%	0.25 [0.06, 0.99]	2004
Hu, Y.2004	4	23	10	22	0.9%	0.25 [0.06, 0.99]	2004
Fang, X.2004	3	15	7	15	0.6%	0.29 [0.06, 1.45]	2004
Feng, X.2005	2	17	7	17	0.7%	0.19 [0.03, 1.11]	2005
Liu, M.2005	3	50	21	48	2.2%	0.08 [0.02, 0.30]	2005
Chen, J.2005	2	27	7	24	0.7%	0.19 [0.04, 1.05]	2005
Yue, Z.2006	5	33	13	28	1.3%	0.21 [0.06, 0.69]	2006
Zhou, F.2007	2	16	7	12	0.7%	0.10 [0.02, 0.66]	2007
Gao, H.2009	7	35	17	30	1.6%	0.19 [0.06, 0.57]	2009
Qu, Y.2010	8	40	19	40	1.6%	0.28 [0.10, 0.75]	2010
Cheng, J.2010	6	30	15	30	1.3%	0.25 [0.08, 0.79]	2010
Li, L. 2013	3	15	7	15	0.6%	0.29 [0.06, 1.45]	2013
Yu, L. 2013	4	23	11	22	1.0%	0.21 [0.05, 0.82]	2013
Zhang, J.2014	8	40	25	40	2.1%	0.15 [0.05, 0.41]	2014
Liu, J. 2015	4	30	13	29	1.2%	0.19 [0.05, 0.68]	2015
Yu, M.2017	2	20	13	20	1.3%	0.06 [0.01, 0.34]	2017
Luo, D.2018	3	30	14	30	1.3%	0.13 [0.03, 0.51]	2018
**Subtotal (95% CI)**		**543**		**520**	**23.5%**	**0.18 [0.13, 0.24]**	
Total events	80		253				
Heterogeneity: Chi^2 = 6.03, df = 17 (P = 0.99); I^2 = 0%							
Test for overall effect: Z = 11.38 (P < 0.00001)							
**Karnofsky Performance Status score(Unclear)**							
Li, H.1998	5	68	23	42	2.8%	0.07 [0.02, 0.20]	1998
Xu, N.2000	6	34	12	30	1.1%	0.32 [0.10, 1.01]	2000
Zhang, G.2000	4	36	12	30	1.2%	0.19 [0.05, 0.67]	2000
Wang, L. 2002	5	29	12	29	1.1%	0.30 [0.09, 0.99]	2000
Xu, Q.2003	4	32	10	30	1.0%	0.29 [0.08, 1.04]	2003
Li, J.2003	4	23	10	21	0.9%	0.23 [0.06, 0.92]	2003
Wang, C.2003	3	15	7	15	0.6%	0.29 [0.06, 1.45]	2003
Zhu, F.2004	6	34	15	30	1.4%	0.21 [0.07, 0.67]	2004
Chen, H.2004	3	24	10	24	0.9%	0.20 [0.05, 0.86]	2004
Mao, W. 2004	4	24	7	16	0.7%	0.26 [0.06, 1.11]	2004
Zhang, T. 2005	4	23	10	21	0.9%	0.23 [0.06, 0.92]	2005
Huang, Y.2005	4	27	9	21	0.9%	0.23 [0.06, 0.91]	2005
Zhao, Z.2006	5	32	14	32	1.3%	0.24 [0.07, 0.78]	2006
Pan, Y.2006	3	28	10	20	1.1%	0.12 [0.03, 0.53]	2006
Chen, Z.2006	3	12	6	11	0.5%	0.28 [0.05, 1.62]	2006
Sun, C.2007	3	24	12	24	1.1%	0.14 [0.03, 0.61]	2007
Yin, W.2007	3	19	10	19	0.9%	0.17 [0.04, 0.78]	2007
Zheng, F.2007	6	24	8	20	0.7%	0.50 [0.14, 1.81]	2007
Li, X.2008	4	25	11	25	1.0%	0.24 [0.06, 0.92]	2008
Zhang, C. 2011	1	19	9	19	0.9%	0.06 [0.01, 0.56]	2011
Yu, D.2012	3	25	7	25	0.7%	0.35 [0.08, 1.55]	2012
Du, Y.2013	6	60	25	60	2.4%	0.16 [0.06, 0.42]	2013
Chen, J. 2014	5	30	15	30	1.3%	0.20 [0.06, 0.66]	2014
Yao, X.2014	2	25	12	25	1.2%	0.09 [0.02, 0.49]	2014
Zhou, Z.2015	4	21	11	21	1.0%	0.21 [0.05, 0.85]	2015
Cai,C.2015	3	21	7	21	0.6%	0.33 [0.07, 1.53]	2015
Zhang, Z.2015	2	45	18	45	1.8%	0.07 [0.01, 0.32]	2015
Li,D.2016	1	40	10	40	1.0%	0.08 [0.01, 0.63]	2016
Yan, L.2016	4	33	8	28	0.8%	0.34 [0.09, 1.30]	2016
Wang, Y. 2018	4	37	10	37	1.0%	0.33 [0.09, 1.16]	2018
Liu, Y. 2019	2	50	23	50	2.4%	0.05 [0.01, 0.22]	2019
Wu, Y. 2019	10	46	27	46	2.3%	0.20 [0.08, 0.49]	2019
**Subtotal (95% CI)**		**985**		**907**	**37.6%**	**0.19 [0.15, 0.24]**	
Total events	126		390				
Heterogeneity: Chi^2 = 19.13, df = 31 (P = 0.95); I^2 = 0%							
Test for overall effect: Z = 14.05 (P < 0.00001)							
**Total (95% CI)**		**2539**		**2385**	**100.0%**	**0.20 [0.18, 0.23]**	
Total events	362		1066				
Heterogeneity: Chi^2 = 43.41, df = 78 (P = 1.00); I^2 = 0%							
Test for overall effect: Z = 22.57 (P < 0.00001)							
Test for subgroup differences: Chi^2 = 5.08, df = 3 (P = 0.17), I^2 = 40.9%							

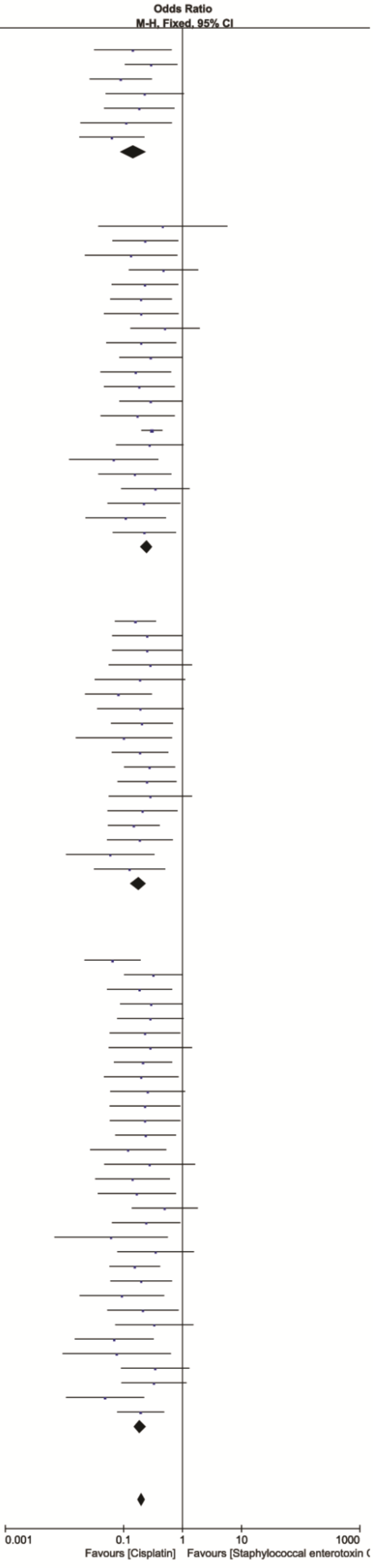

Fig.S24 Subgroups analysis of treatment failure via KPS score

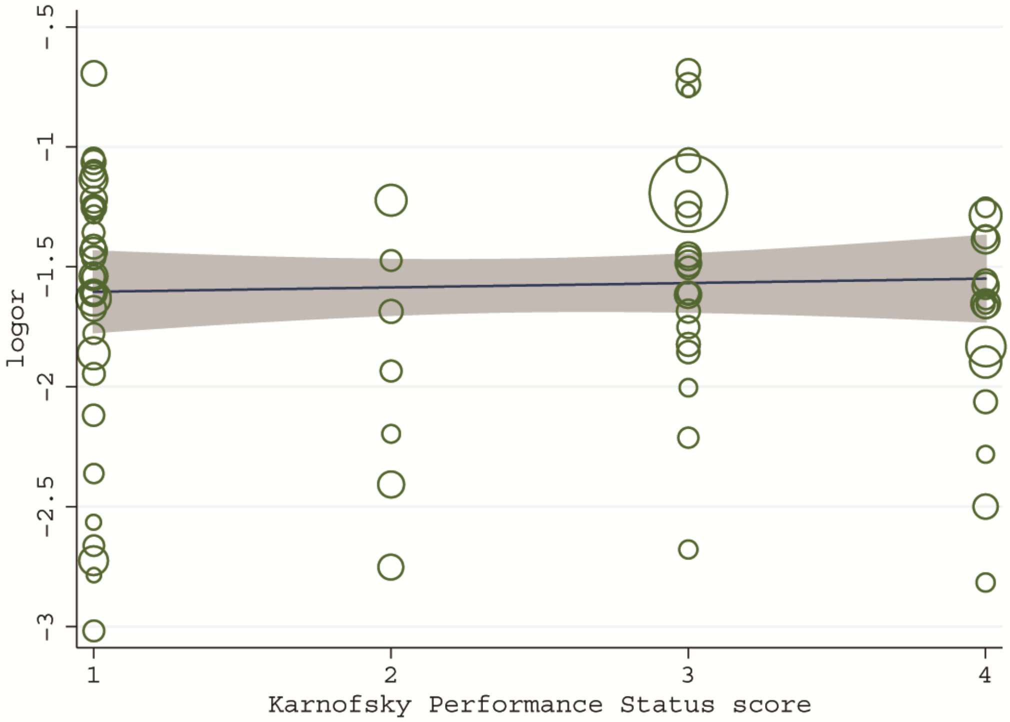

Fig.S25 Meta regression of treatment failure via KPS score

Study or Subgroup	Staphylococcal enterotoxin C Events	Total	Cisplatin Events	Total	Weight	Odds Ratio M-H, Fixed, 95% CI	Year
**Primary treatment**							
Gao, H.2009	17	35	6	30	1.1%	3.78 [1.24, 11.50]	2009
Liu, J. 2015	15	30	7	29	1.2%	3.14 [1.03, 9.55]	2015
**Subtotal (95% CI)**		**65**		**59**	**2.4%**	**3.45 [1.57, 7.57]**	
Total events	32		13				
Heterogeneity: Chi² = 0.05, df = 1 (P = 0.82); I² = 0%							
Test for overall effect: Z = 3.09 (P = 0.002)							
**Unclear**							
Li, S.1997	10	20	3	20	0.5%	5.67 [1.25, 25.61]	1997
Li, H.1998	18	68	6	42	1.9%	2.16 [0.78, 5.98]	1998
Qiu, Y.1999	12	42	7	42	1.7%	2.00 [0.70, 5.73]	1999
Zhang, Q.1999	10	15	4	15	0.5%	5.50 [1.15, 26.41]	1999
Cao, H.2000	6	26	1	18	0.3%	5.10 [0.56, 46.65]	2000
Jia, X.2000	6	15	2	15	0.4%	4.33 [0.71, 26.53]	2000
Xu, N.2000	10	34	8	30	2.1%	1.15 [0.38, 3.43]	2000
Zhang, G.2000	20	36	6	30	1.0%	5.00 [1.65, 15.17]	2000
Wang, L. 2002	7	29	4	29	1.0%	1.99 [0.51, 7.71]	2000
Lang, F.2001	36	56	6	21	1.1%	4.50 [1.51, 13.43]	2001
Wang, Lei.2002	11	21	7	19	1.2%	1.89 [0.53, 6.69]	2002
Duan, R.2003	27	76	16	76	3.6%	2.07 [1.00, 4.26]	2003
Wang, C.2003	5	15	2	15	0.5%	3.25 [0.52, 20.37]	2003
Xu, Q.2003	20	32	12	30	1.6%	2.50 [0.90, 6.95]	2003
Zhang, J.2003	9	25	2	28	0.4%	7.31 [1.40, 38.22]	2003
Li, J.2003	7	23	4	21	1.0%	1.86 [0.46, 7.58]	2003
Chen, H.2004	13	24	6	24	0.9%	3.55 [1.04, 12.06]	2004
Fang, X.2004	3	15	1	15	0.3%	3.50 [0.32, 38.23]	2004
Guan, C.2004	13	23	6	22	0.9%	3.47 [0.99, 12.09]	2004
Hu, Y.2004	13	23	6	22	0.9%	3.47 [0.99, 12.09]	2004
Zhu, F.2004	16	34	6	30	1.2%	3.56 [1.16, 10.89]	2004
Mao, W. 2004	8	24	2	16	0.6%	3.50 [0.63, 19.30]	2004
Liu, S.2005	13	32	6	29	1.3%	2.62 [0.84, 8.22]	2005
Ma, D.2005	13	24	6	24	0.9%	3.55 [1.04, 12.06]	2005
Zhang, T. 2005	8	23	5	21	1.2%	1.71 [0.46, 6.39]	2005
Feng, X.2005	6	17	4	17	0.9%	1.77 [0.40, 7.93]	2005
Chen, J.2005	11	27	5	24	1.1%	2.61 [0.75, 9.11]	2005
Huang, Y.2005	9	27	4	21	1.0%	2.13 [0.55, 8.21]	2005
Liu, M.2005	35	50	14	48	1.5%	5.67 [2.38, 13.50]	2005
Xiong, C.2006	10	22	6	22	1.1%	2.22 [0.63, 7.82]	2006
Yue, Z.2006	10	33	3	28	0.8%	3.62 [0.89, 14.83]	2006
Zhang, Z.2006	6	16	2	16	0.4%	4.20 [0.70, 25.26]	2006
Zhao, Z.2006	9	32	5	32	1.2%	2.11 [0.62, 7.20]	2006
Fang, H.2006	6	24	2	22	0.5%	3.33 [0.60, 18.66]	2006
Pan, Y.2006	16	28	4	20	0.7%	5.33 [1.41, 20.10]	2006
Chen, Z.2006	4	12	2	11	0.5%	2.25 [0.32, 15.76]	2006
Sun, C.2007	13	24	4	24	0.6%	5.91 [1.55, 22.58]	2007
Yin, W.2007	6	19	4	19	0.9%	1.73 [0.40, 7.51]	2007
Zhou, F.2007	5	16	0	12	0.1%	11.96 [0.59, 241.06]	2007
Zheng, F.2007	4	24	3	20	0.9%	1.13 [0.22, 5.79]	2007
Zhang, M.2008	11	33	4	34	0.9%	3.75 [1.05, 13.35]	2008
Huang, W.2008	12	28	9	28	1.8%	1.58 [0.53, 4.71]	2008
Li, X.2008	8	25	6	25	1.4%	1.49 [0.43, 5.17]	2008
Wang, H.2009	12	28	9	28	1.8%	1.58 [0.53, 4.71]	2009
Zhang, L.2009	11	34	4	35	0.9%	3.71 [1.05, 13.13]	2009
Qu, Y.2010	4	40	2	40	0.6%	2.11 [0.36, 12.24]	2010
Cheng, J.2010	5	30	0	30	0.1%	13.16 [0.69, 249.48]	2010
Gui, Q. 2010	9	25	3	25	0.7%	4.13 [0.96, 17.70]	2010
Li, Z. 2010	100	230	70	230	13.7%	1.76 [1.20, 2.58]	2010
Liu, Q.2010	5	27	3	20	1.0%	1.29 [0.27, 6.16]	2010
Zhang, C. 2011	13	19	4	19	0.4%	8.13 [1.87, 35.23]	2011
Xing, Q.2012	7	19	3	19	0.7%	3.11 [0.66, 14.60]	2012
Xu, J.2012	14	27	9	27	1.5%	2.15 [0.72, 6.47]	2012
Xu, Q.2012	11	27	9	27	1.8%	1.38 [0.45, 4.17]	2012
Yu, D.2012	17	25	12	25	1.3%	2.30 [0.73, 7.27]	2012
Qin, Q.2012	19	34	6	34	0.9%	5.91 [1.94, 17.97]	2012
Li, W. 2013	13	30	11	30	2.2%	1.32 [0.47, 3.72]	2013
Du, Y.2013	31	60	22	60	3.7%	1.85 [0.89, 3.83]	2013
Li, L. 2013	4	15	0	15	0.1%	12.13 [0.59, 248.49]	2013
Yu, L. 2013	2	23	1	22	0.3%	2.00 [0.17, 23.78]	2013
Zhao, Y. 2014	25	40	16	40	2.1%	2.50 [1.02, 6.15]	2014
Tu, D.2014	14	38	14	38	3.1%	1.00 [0.39, 2.54]	2014
Yao, X.2014	13	25	7	25	1.2%	2.79 [0.86, 9.01]	2014
Zhang, J.2014	13	40	6	40	1.4%	2.73 [0.92, 8.13]	2014
Chen, J. 2014	6	30	2	30	0.6%	3.50 [0.65, 18.98]	2014
Zhang, Z.2015	28	45	17	45	2.2%	2.71 [1.16, 6.36]	2015
Cai,C.2015	11	21	6	21	1.0%	2.75 [0.77, 9.86]	2015
Zhou, Z.2015	6	21	1	21	0.2%	8.00 [0.87, 73.68]	2015
Yan, L.2016	16	33	7	28	1.3%	2.82 [0.95, 8.44]	2016
Li,D.2016	27	40	19	40	2.1%	2.30 [0.93, 5.69]	2016
Yu, M.2017	7	20	3	20	0.7%	3.05 [0.66, 14.14]	2017
Luo, D.2018	12	30	6	30	1.2%	2.67 [0.84, 8.46]	2018
Wang, Y. 2018	20	37	17	37	2.7%	1.38 [0.55, 3.45]	2018
Liu, Y. 2019	28	50	7	50	1.1%	7.82 [2.95, 20.72]	2019
Wu, Y. 2019	15	46	7	46	1.6%	2.70 [0.98, 7.43]	2019
**Subtotal (95% CI)**		**2421**		**2274**	**97.6%**	**2.57 [2.26, 2.94]**	
Total events	1013		518				
Heterogeneity: Chi² = 51.49, df = 74 (P = 0.98); I² = 0%							
Test for overall effect: Z = 14.08 (P < 0.00001)							
**Total (95% CI)**		**2486**		**2333**	**100.0%**	**2.59 [2.28, 2.95]**	
Total events	1045		531				
Heterogeneity: Chi² = 52.12, df = 76 (P = 0.98); I² = 0%							
Test for overall effect: Z = 14.40 (P < 0.00001)							
Test for subgroup differences: Chi² = 0.52, df = 1 (P = 0.47), I² = 0%							

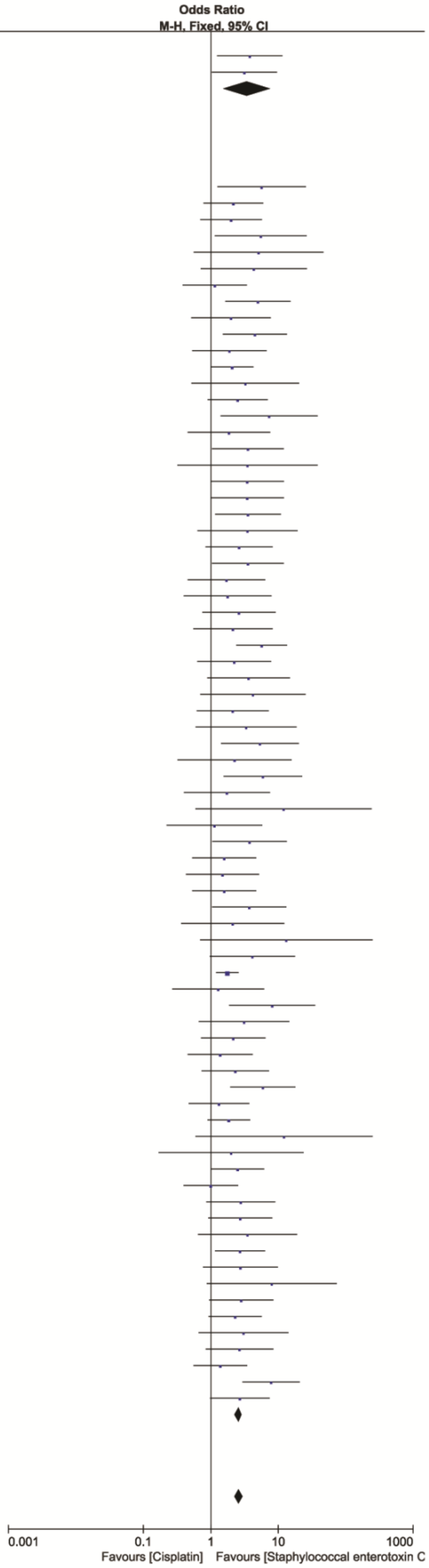

Fig.S26 Subgroups analysis of complete response via treatment history

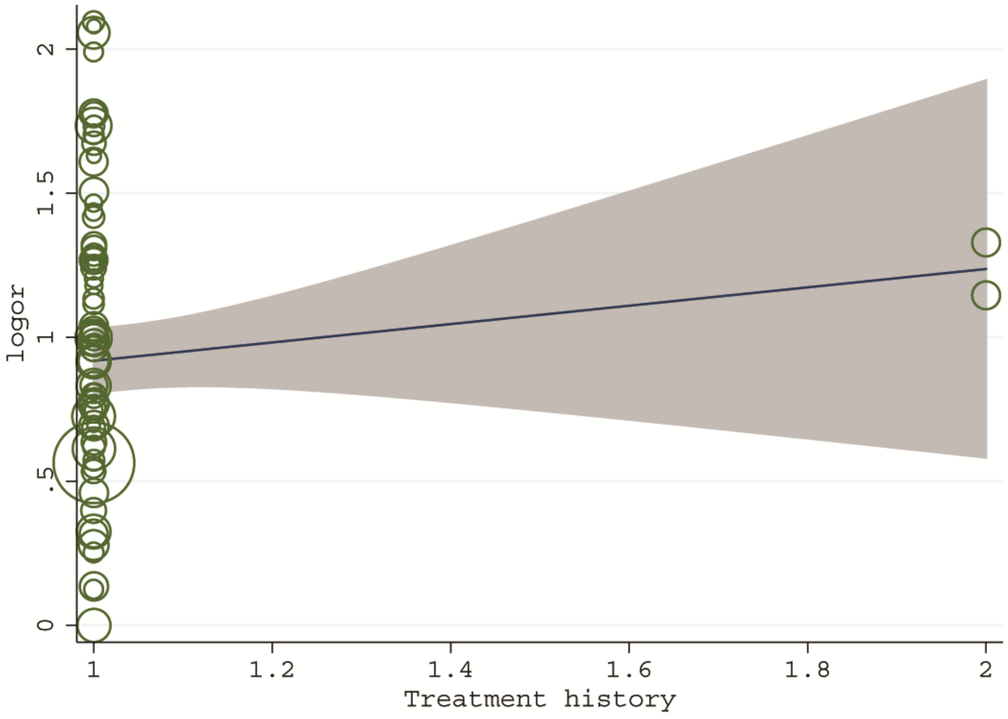

Fig.S27 Meta-regression of complete response via treatment history

Study or Subgroup	Staphylococcal enterotoxin C Events	Staphylococcal enterotoxin C Total	Cisplatin Events	Cisplatin Total	Weight	Odds Ratio M-H, Fixed, 95% CI	Year
**Primary treatment**							
Wang, C. 2004	5	23	12	22	1.0%	0.23 [0.06, 0.85]	2004
Gao, H.2009	7	35	17	30	1.6%	0.19 [0.06, 0.57]	2009
Liu, J. 2015	4	30	13	29	1.2%	0.19 [0.05, 0.68]	2015
**Subtotal (95% CI)**		**88**		**81**	**3.8%**	**0.20 [0.10, 0.41]**	
Total events	16		42				
Heterogeneity: Chi² = 0.06, df = 2 (P = 0.97); I² = 0%							
Test for overall effect: Z = 4.48 (P < 0.00001)							
**Unclear**							
Li, S.1997	3	20	11	20	1.0%	0.14 [0.03, 0.65]	1997
Li, H.1998	5	68	23	42	2.8%	0.07 [0.02, 0.20]	1998
Qiu, Y.1999	7	42	17	42	1.5%	0.29 [0.11, 0.81]	1999
Zhang, Q.1999	1	15	2	15	0.2%	0.46 [0.04, 5.75]	1999
Xu, N.2000	6	34	12	30	1.1%	0.32 [0.10, 1.01]	2000
Zhang, G.2000	4	36	12	30	1.2%	0.19 [0.05, 0.67]	2000
Cao, H.2000	7	26	11	18	1.0%	0.23 [0.06, 0.85]	2000
Jia, X.2000	2	15	8	15	0.7%	0.13 [0.02, 0.82]	2000
Wang, L. 2002	5	29	12	29	1.1%	0.30 [0.09, 0.99]	2000
Lang, F.2001	6	56	12	21	1.7%	0.09 [0.03, 0.30]	2001
Wang, Lei.2002	3	21	8	19	0.8%	0.23 [0.05, 1.05]	2002
Xu, Q.2003	4	32	10	30	1.0%	0.29 [0.08, 1.04]	2003
Zhang, J.2003	4	25	8	28	0.7%	0.48 [0.12, 1.83]	2003
Duan, R.2003	10	76	37	76	3.4%	0.16 [0.07, 0.36]	2003
Li, J.2003	4	23	10	21	0.9%	0.23 [0.06, 0.92]	2003
Wang, C.2003	3	15	7	15	0.6%	0.29 [0.06, 1.45]	2003
Hu, Y.2004	4	23	10	22	0.9%	0.25 [0.06, 0.99]	2004
Zhu, F.2004	6	34	15	30	1.4%	0.21 [0.07, 0.67]	2004
Chen, H.2004	3	24	10	24	0.9%	0.20 [0.05, 0.86]	2004
Mao, W. 2004	4	24	7	16	0.7%	0.26 [0.06, 1.11]	2004
Fang, X.2004	3	15	7	15	0.6%	0.29 [0.06, 1.45]	2004
Guan, C.2004	4	23	10	22	0.9%	0.25 [0.06, 0.99]	2004
Zhang, T. 2005	4	23	10	21	0.9%	0.23 [0.06, 0.92]	2005
Chen, J.2005	2	27	7	24	0.7%	0.19 [0.04, 1.05]	2005
Feng, X.2005	2	17	7	17	0.7%	0.19 [0.03, 1.11]	2005
Huang, Y.2005	4	27	9	21	0.9%	0.23 [0.06, 0.91]	2005
Liu, S.2005	5	32	14	29	1.3%	0.20 [0.06, 0.66]	2005
Ma, D.2005	3	24	10	24	0.9%	0.20 [0.05, 0.86]	2005
Liu, M.2005	3	50	21	48	2.2%	0.08 [0.02, 0.30]	2005
Xiong, C.2006	4	22	12	22	1.1%	0.19 [0.05, 0.73]	2006
Yue, Z.2006	5	33	13	28	1.3%	0.21 [0.06, 0.69]	2006
Zhang, Z.2006	2	16	9	16	0.8%	0.11 [0.02, 0.66]	2006
Zhao, Z.2006	5	32	14	32	1.3%	0.24 [0.07, 0.78]	2006
Fang, H.2006	4	24	11	22	1.0%	0.20 [0.05, 0.78]	2006
Pan, Y.2006	3	28	10	20	1.1%	0.12 [0.03, 0.53]	2006
Wang, J.2006	4	30	7	30	0.6%	0.51 [0.13, 1.95]	2006
Chen, Z.2006	3	12	6	11	0.5%	0.28 [0.05, 1.62]	2006
Sun, C.2007	3	24	12	24	1.1%	0.14 [0.03, 0.61]	2007
Yin, W.2007	3	19	10	19	0.9%	0.17 [0.04, 0.78]	2007
Zhou, F.2007	2	16	7	12	0.7%	0.10 [0.02, 0.66]	2007
Zheng, F.2007	6	24	8	20	0.7%	0.50 [0.14, 1.81]	2007
Huang, W.2008	5	28	12	28	1.1%	0.29 [0.09, 0.98]	2008
Li, X.2008	4	25	11	25	1.0%	0.24 [0.06, 0.92]	2008
Zhang, M.2008	3	33	13	34	1.2%	0.16 [0.04, 0.64]	2008
Wang, H.2009	5	28	12	28	1.1%	0.29 [0.09, 0.98]	2009
Zhang, L.2009	3	34	12	35	1.2%	0.19 [0.05, 0.73]	2009
Li, Z. 2010	50	230	110	230	9.2%	0.30 [0.20, 0.45]	2010
Liu, Q.2010	5	27	9	20	0.9%	0.28 [0.07, 1.03]	2010
Gui, Q. 2010	3	25	11	25	1.0%	0.17 [0.04, 0.73]	2010
Cheng, J.2010	6	30	15	30	1.3%	0.25 [0.08, 0.79]	2010
Qu, Y.2010	8	40	19	40	1.6%	0.28 [0.10, 0.75]	2010
Zhang, C. 2011	1	19	9	19	0.9%	0.06 [0.01, 0.56]	2011
Qin, Q.2012	4	34	23	34	2.2%	0.06 [0.02, 0.23]	2012
Xing, Q.2012	2	19	12	19	1.1%	0.07 [0.01, 0.39]	2012
Xu, J.2012	3	27	12	27	1.1%	0.16 [0.04, 0.65]	2012
Xu, Q.2012	4	27	9	27	0.8%	0.35 [0.09, 1.31]	2012
Yu, D.2012	3	25	7	25	0.7%	0.35 [0.08, 1.55]	2012
Du, Y.2013	6	60	25	60	2.4%	0.16 [0.06, 0.42]	2013
Li, L. 2013	3	15	7	15	0.6%	0.29 [0.06, 1.45]	2013
Li, W. 2013	3	30	10	30	1.0%	0.22 [0.05, 0.91]	2013
Yu, L. 2013	4	23	11	22	1.0%	0.21 [0.05, 0.82]	2013
Zhao, Y. 2014	2	40	13	40	1.3%	0.11 [0.02, 0.52]	2014
Zhang, J.2014	8	40	25	40	2.1%	0.15 [0.05, 0.41]	2014
Chen, J. 2014	5	30	15	30	1.3%	0.20 [0.06, 0.66]	2014
Tu, D.2014	4	38	13	38	1.2%	0.23 [0.07, 0.78]	2014
Yao, X.2014	2	25	12	25	1.2%	0.09 [0.02, 0.49]	2014
Zhou, Z.2015	4	21	11	21	1.0%	0.21 [0.05, 0.85]	2015
Cai,C.2015	3	21	7	21	0.6%	0.33 [0.07, 1.53]	2015
Zhang, Z.2015	2	45	18	45	1.8%	0.07 [0.01, 0.32]	2015
Li,D.2016	1	40	10	40	1.0%	0.08 [0.01, 0.63]	2016
Yan, L.2016	4	33	8	28	0.8%	0.34 [0.09, 1.30]	2016
Yu, M.2017	2	20	13	20	1.3%	0.06 [0.01, 0.34]	2017
Luo, D.2018	3	30	14	30	1.3%	0.13 [0.03, 0.51]	2018
Wang, Y. 2018	4	37	10	37	1.0%	0.33 [0.09, 1.16]	2018
Liu, Y. 2019	2	50	23	50	2.4%	0.05 [0.01, 0.22]	2019
Wu, Y. 2019	10	46	27	46	2.3%	0.20 [0.08, 0.49]	2019
**Subtotal (95% CI)**		**2451**		**2304**	**96.2%**	**0.20 [0.17, 0.23]**	
Total events	346		1024				
Heterogeneity: Chi² = 43.35, df = 75 (P = 1.00); I² = 0%							
Test for overall effect: Z = 22.12 (P < 0.00001)							
**Total (95% CI)**		**2539**		**2385**	**100.0%**	**0.20 [0.18, 0.23]**	
Total events	362		1066				
Heterogeneity: Chi² = 43.41, df = 78 (P = 1.00); I² = 0%							
Test for overall effect: Z = 22.57 (P < 0.00001)							
Test for subgroup differences: Chi² = 0.00, df = 1 (P = 1.00), I² = 0%							

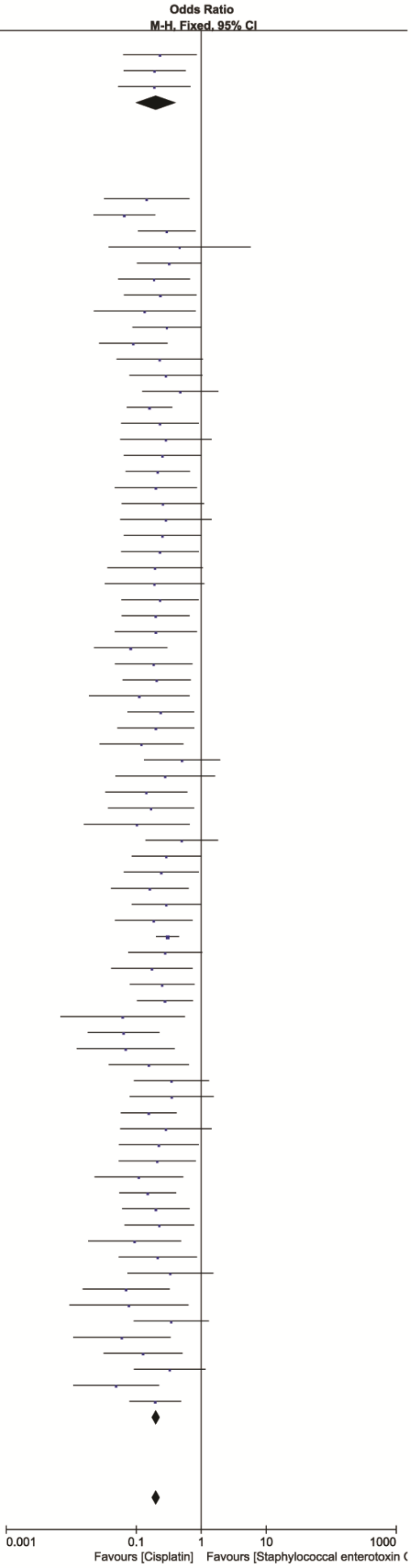

Fig.S28 Subgroups analysis of treatment failure via treatment history

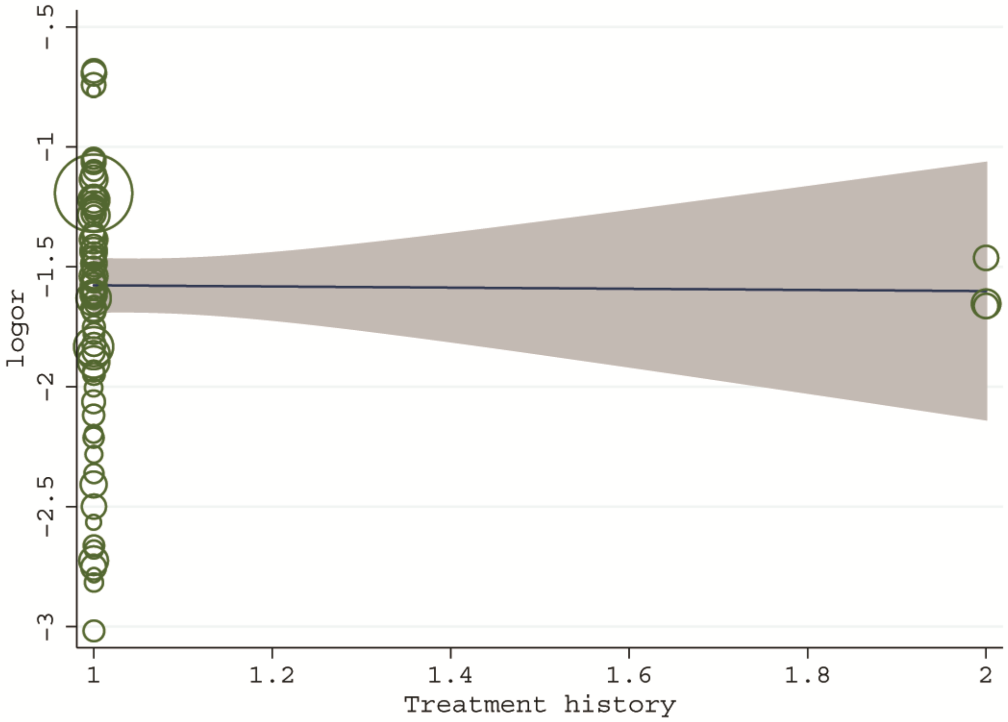

Fig.S29 Meta-regression of treatment failure via treatment history

Study or Subgroup	Staphylococcal enterotoxin C Events	Staphylococcal enterotoxin C Total	Cisplatin Events	Cisplatin Total	Weight	Odds Ratio M-H, Fixed, 95% CI	Year
**Anticipated survival time (>2 months)**							
Liu, M.2005	35	50	14	48	1.5%	5.67 [2.38, 13.50]	2005
Liu, S.2005	13	32	6	29	1.3%	2.62 [0.84, 8.22]	2005
Gui, Q. 2010	9	25	3	25	0.7%	4.13 [0.96, 17.70]	2010
Li, Z. 2010	100	230	70	230	13.7%	1.76 [1.20, 2.58]	2010
**Subtotal (95% CI)**		**337**		**332**	**17.1%**	**2.25 [1.63, 3.11]**	
Total events	157		93				
Heterogeneity: Chi² = 6.68, df = 3 (P = 0.08); I² = 55%							
Test for overall effect: Z = 4.94 (P < 0.00001)							
**Anticipated survival time (≥ 3 months)**							
Lang, F.2001	36	56	6	21	1.1%	4.50 [1.51, 13.43]	2001
Wang, Lei.2002	11	21	7	19	1.2%	1.89 [0.53, 6.69]	2002
Fang, X.2004	3	15	1	15	0.3%	3.50 [0.32, 38.23]	2004
Guan, C.2004	13	23	6	22	0.9%	3.47 [0.99, 12.09]	2004
Hu, Y.2004	13	23	6	22	0.9%	3.47 [0.99, 12.09]	2004
Feng, X.2005	6	17	4	17	0.9%	1.77 [0.40, 7.93]	2005
Fang, H.2006	6	24	2	22	0.5%	3.33 [0.60, 18.66]	2006
Yue, Z.2006	10	33	3	28	0.8%	3.62 [0.89, 14.83]	2006
Zhang, M.2008	11	33	4	34	0.9%	3.75 [1.05, 13.35]	2008
Huang, W.2008	12	28	9	28	1.8%	1.58 [0.53, 4.71]	2008
Zhang, L.2009	11	34	4	35	0.9%	3.71 [1.05, 13.13]	2009
Wang, H.2009	12	28	9	28	1.8%	1.58 [0.53, 4.71]	2009
Cheng, J.2010	5	30	0	30	0.1%	13.16 [0.69, 249.48]	2010
Liu, Q.2010	5	27	3	20	1.0%	1.29 [0.27, 6.16]	2010
Qu, Y.2010	4	40	2	40	0.6%	2.11 [0.36, 12.24]	2010
Xu, Q.2012	11	27	9	27	1.8%	1.38 [0.45, 4.17]	2012
Xu, J.2012	14	27	9	27	1.5%	2.15 [0.72, 6.47]	2012
Du, Y.2013	31	60	22	60	3.7%	1.85 [0.89, 3.83]	2013
Li, W. 2013	13	30	11	30	2.2%	1.32 [0.47, 3.72]	2013
Yu, L. 2013	2	23	1	22	0.3%	2.00 [0.17, 23.78]	2013
Zhao, Y. 2014	25	40	16	40	2.1%	2.50 [1.02, 6.15]	2014
Yao, X.2014	13	25	7	25	1.2%	2.79 [0.86, 9.01]	2014
Liu, J. 2015	15	30	7	29	1.2%	3.14 [1.03, 9.55]	2015
Li,D.2016	27	40	19	40	2.1%	2.30 [0.93, 5.69]	2016
Yu, M.2017	7	20	3	20	0.7%	3.05 [0.66, 14.14]	2017
Luo, D.2018	12	30	6	30	1.2%	2.67 [0.84, 8.46]	2018
Liu, Y. 2019	28	50	7	50	1.1%	7.82 [2.95, 20.72]	2019
**Subtotal (95% CI)**		**834**		**781**	**32.8%**	**2.58 [2.05, 3.23]**	
Total events	356		183				
Heterogeneity: Chi² = 15.46, df = 26 (P = 0.95); I² = 0%							
Test for overall effect: Z = 8.17 (P < 0.00001)							
**Unclear**							
Li, S.1997	10	20	3	20	0.5%	5.67 [1.25, 25.61]	1997
Li, H.1998	18	68	6	42	1.9%	2.16 [0.78, 5.98]	1998
Qiu, Y.1999	12	42	7	42	1.7%	2.00 [0.70, 5.73]	1999
Zhang, Q.1999	10	15	4	15	0.5%	5.50 [1.15, 26.41]	1999
Cao, H.2000	6	26	1	18	0.3%	5.10 [0.56, 46.65]	2000
Jia, X.2000	6	15	2	15	0.4%	4.33 [0.71, 26.53]	2000
Xu, N.2000	10	34	8	30	2.1%	1.15 [0.38, 3.43]	2000
Zhang, G.2000	20	36	6	30	1.0%	5.00 [1.65, 15.17]	2000
Wang, L. 2002	7	29	4	29	1.0%	1.99 [0.51, 7.71]	2000
Duan, R.2003	27	76	16	76	3.6%	2.07 [1.00, 4.26]	2003
Wang, C.2003	5	15	2	15	0.5%	3.25 [0.52, 20.37]	2003
Xu, Q.2003	20	32	12	30	1.6%	2.50 [0.90, 6.95]	2003
Zhang, J.2003	9	25	2	28	0.4%	7.31 [1.40, 38.22]	2003
Li, J.2003	7	23	4	21	1.0%	1.86 [0.46, 7.58]	2003
Chen, H.2004	13	24	6	24	0.9%	3.55 [1.04, 12.06]	2004
Zhu, F.2004	16	34	6	30	1.2%	3.56 [1.16, 10.89]	2004
Mao, W. 2004	8	24	2	16	0.6%	3.50 [0.63, 19.30]	2004
Ma, D.2005	13	24	6	24	0.9%	3.55 [1.04, 12.06]	2005
Zhang, T. 2005	8	23	5	21	1.2%	1.71 [0.46, 6.39]	2005
Chen, J.2005	11	27	5	24	1.1%	2.61 [0.75, 9.11]	2005
Huang, Y.2005	9	27	4	21	1.0%	2.13 [0.55, 8.21]	2005
Xiong, C.2006	10	22	6	22	1.1%	2.22 [0.63, 7.82]	2006
Zhang, Z.2006	6	16	2	16	0.4%	4.20 [0.70, 25.26]	2006
Zhao, Z.2006	9	32	5	32	1.2%	2.11 [0.62, 7.20]	2006
Pan, Y.2006	16	28	4	20	0.7%	5.33 [1.41, 20.10]	2006
Chen, Z.2006	4	12	2	11	0.5%	2.25 [0.32, 15.76]	2006
Sun, C.2007	13	24	4	24	0.6%	5.91 [1.55, 22.58]	2007
Yin, W.2007	6	19	4	19	0.9%	1.73 [0.40, 7.51]	2007
Zhou, F.2007	5	16	0	12	0.1%	11.96 [0.59, 241.06]	2007
Zheng, F.2007	4	24	3	20	0.9%	1.13 [0.22, 5.79]	2007
Li, X.2008	8	25	6	25	1.4%	1.49 [0.43, 5.17]	2008
Gao, H.2009	17	35	6	30	1.1%	3.78 [1.24, 11.50]	2009
Zhang, C. 2011	13	19	4	19	0.4%	8.13 [1.87, 35.23]	2011
Xing, Q.2012	7	19	3	19	0.7%	3.11 [0.66, 14.60]	2012
Yu, D.2012	17	25	12	25	1.3%	2.30 [0.73, 7.27]	2012
Qin, Q.2012	19	34	6	34	0.9%	5.91 [1.94, 17.97]	2012
Li, L. 2013	4	15	0	15	0.1%	12.13 [0.59, 248.49]	2013
Tu, D.2014	14	38	14	38	3.1%	1.00 [0.39, 2.54]	2014
Zhang, J.2014	13	40	6	40	1.4%	2.73 [0.92, 8.13]	2014
Chen, J. 2014	6	30	2	30	0.6%	3.50 [0.65, 18.98]	2014
Zhang, Z.2015	28	45	17	45	2.2%	2.71 [1.16, 6.36]	2015
Cai,C.2015	11	21	6	21	1.0%	2.75 [0.77, 9.86]	2015
Zhou, Z.2015	6	21	1	21	0.2%	8.00 [0.87, 73.68]	2015
Yan, L.2016	16	33	7	28	1.3%	2.82 [0.95, 8.44]	2016
Wang, Y. 2018	20	37	17	37	2.7%	1.38 [0.55, 3.45]	2018
Wu, Y. 2019	15	46	7	46	1.6%	2.70 [0.98, 7.43]	2019
**Subtotal (95% CI)**		**1315**		**1220**	**50.1%**	**2.72 [2.27, 3.27]**	
Total events	532		255				
Heterogeneity: Chi² = 29.10, df = 45 (P = 0.97); I² = 0%							
Test for overall effect: Z = 10.80 (P < 0.00001)							
**Total (95% CI)**		**2486**		**2333**	**100.0%**	**2.59 [2.28, 2.95]**	
Total events	1045		531				
Heterogeneity: Chi² = 52.12, df = 76 (P = 0.98); I² = 0%							
Test for overall effect: Z = 14.40 (P < 0.00001)							
Test for subgroup differences: Chi² = 1.01, df = 2 (P = 0.60), I² = 0%							

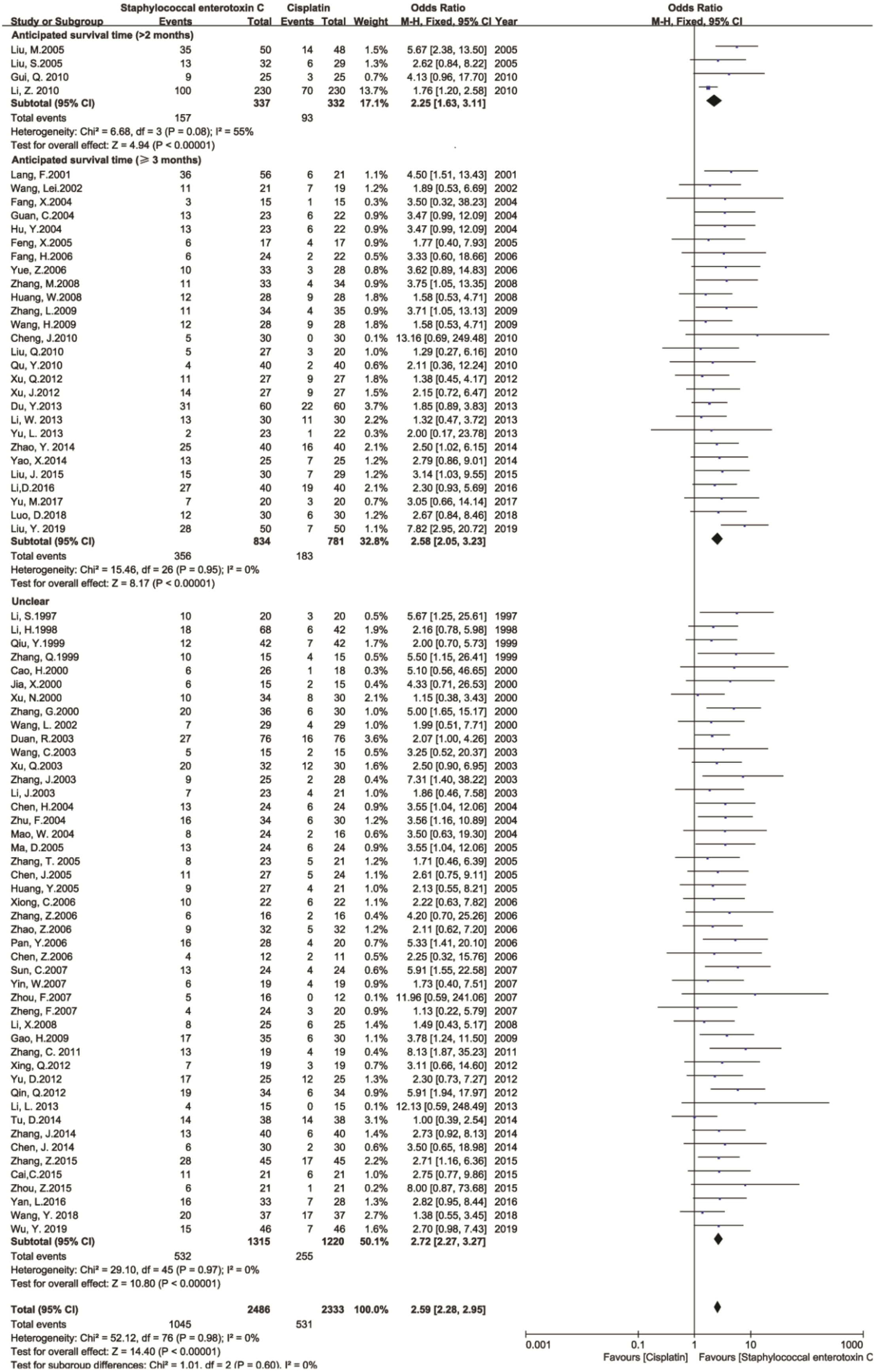

Fig.S30 Subgroups analysis of complete response via anticipated survival time

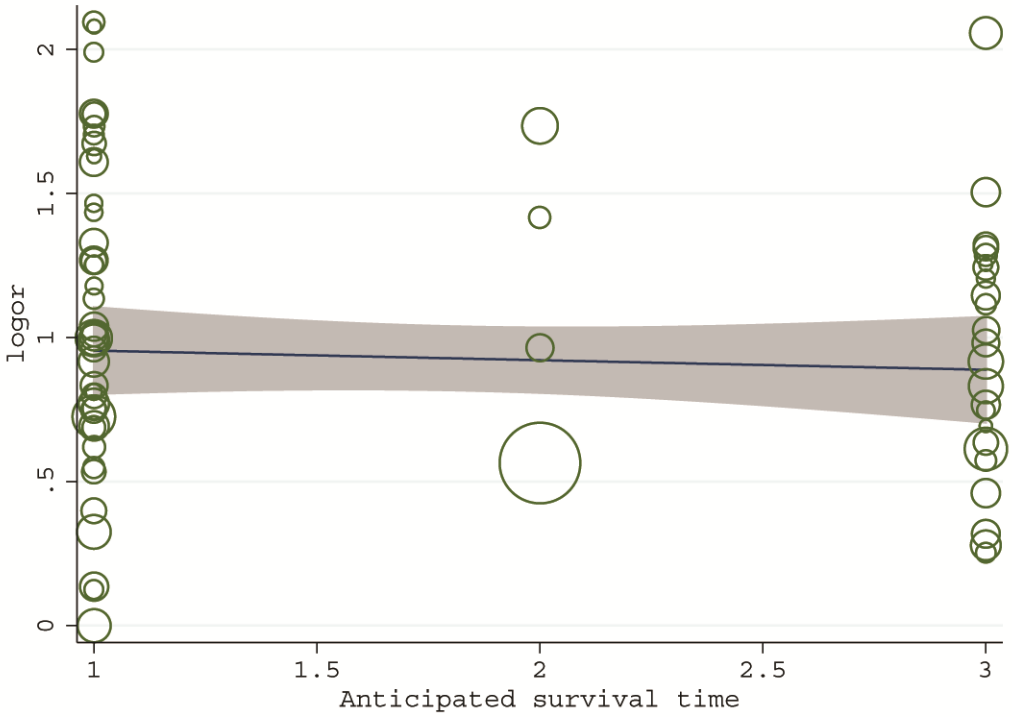

Fig.S31 Meta regression of complete response via anticipated survival time

	Staphylococcal enterotoxin C		Cisplatin			Odds Ratio	
Study or Subgroup	Events	Total	Events	Total	Weight	M-H, Fixed, 95% CI	Year
**Anticipated survival time (>2 months)**							
Liu, M.2005	3	50	21	48	2.2%	0.08 [0.02, 0.30]	2005
Liu, S.2005	5	32	14	29	1.3%	0.20 [0.06, 0.66]	2005
Gui, Q. 2010	3	25	11	25	1.0%	0.17 [0.04, 0.73]	2010
Li, Z. 2010	50	230	110	230	9.2%	0.30 [0.20, 0.45]	2010
**Subtotal (95% CI)**		**337**		**332**	**13.7%**	**0.25 [0.17, 0.35]**	
Total events	61		156				
Heterogeneity: Chi² = 4.08, df = 3 (P = 0.25); I² = 27%							
Test for overall effect: Z = 7.73 (P < 0.00001)							
**Anticipated survival time (⩾ 3 months)**							
Lang, F.2001	6	56	12	21	1.7%	0.09 [0.03, 0.30]	2001
Wang, Lei.2002	3	21	8	19	0.8%	0.23 [0.05, 1.05]	2002
Fang, X.2004	3	15	7	15	0.6%	0.29 [0.06, 1.45]	2004
Guan, C.2004	4	23	10	22	0.9%	0.25 [0.06, 0.99]	2004
Hu, Y.2004	4	23	10	22	0.9%	0.25 [0.06, 0.99]	2004
Feng, X.2005	2	17	7	17	0.7%	0.19 [0.03, 1.11]	2005
Fang, H.2006	4	24	11	22	1.0%	0.20 [0.05, 0.78]	2006
Yue, Z.2006	5	33	13	28	1.3%	0.21 [0.06, 0.69]	2006
Huang, W.2008	5	28	12	28	1.1%	0.29 [0.09, 0.98]	2008
Zhang, M.2008	3	33	13	34	1.2%	0.16 [0.04, 0.64]	2008
Wang, H.2009	5	28	12	28	1.1%	0.29 [0.09, 0.98]	2009
Zhang, L.2009	3	34	12	35	1.2%	0.19 [0.05, 0.73]	2009
Cheng, J.2010	6	30	15	30	1.3%	0.25 [0.08, 0.79]	2010
Liu, Q.2010	5	27	9	20	0.9%	0.28 [0.07, 1.03]	2010
Qu, Y.2010	8	40	19	40	1.6%	0.28 [0.10, 0.75]	2010
Xu, J.2012	3	27	12	27	1.1%	0.16 [0.04, 0.65]	2012
Xu, Q.2012	4	27	9	27	0.8%	0.35 [0.09, 1.31]	2012
Du, Y.2013	6	60	25	60	2.4%	0.16 [0.06, 0.42]	2013
Li, W. 2013	3	30	10	30	1.0%	0.22 [0.05, 0.91]	2013
Yu, L. 2013	4	23	11	22	1.0%	0.21 [0.05, 0.82]	2013
Yao, X.2014	2	25	12	25	1.2%	0.09 [0.02, 0.49]	2014
Zhao, Y. 2014	2	40	13	40	1.3%	0.11 [0.02, 0.52]	2014
Liu, J. 2015	4	30	13	29	1.2%	0.19 [0.05, 0.68]	2015
Li,D.2016	1	40	10	40	1.0%	0.08 [0.01, 0.63]	2016
Yu, M.2017	2	20	13	20	1.3%	0.06 [0.01, 0.34]	2017
Luo, D.2018	3	30	14	30	1.3%	0.13 [0.03, 0.51]	2018
Liu, Y. 2019	2	50	23	50	2.4%	0.05 [0.01, 0.22]	2019
**Subtotal (95% CI)**		**834**		**781**	**32.2%**	**0.18 [0.14, 0.23]**	
Total events	102		335				
Heterogeneity: Chi² = 12.31, df = 26 (P = 0.99); I² = 0%							
Test for overall effect: Z = 13.17 (P < 0.00001)							
**Unclear**							
Li, S.1997	3	20	11	20	1.0%	0.14 [0.03, 0.65]	1997
Li, H.1998	5	68	23	42	2.8%	0.07 [0.02, 0.20]	1998
Qiu, Y.1999	7	42	17	42	1.5%	0.29 [0.11, 0.81]	1999
Zhang, Q.1999	1	15	2	15	0.2%	0.46 [0.04, 5.75]	1999
Xu, N.2000	6	34	12	30	1.1%	0.32 [0.10, 1.01]	2000
Zhang, G.2000	4	36	12	30	1.2%	0.19 [0.05, 0.67]	2000
Cao, H.2000	7	26	11	18	1.0%	0.23 [0.06, 0.85]	2000
Jia, X.2000	2	15	8	15	0.7%	0.13 [0.02, 0.82]	2000
Wang, L. 2002	5	29	12	29	1.1%	0.30 [0.09, 0.99]	2000
Xu, Q.2003	4	32	10	30	1.0%	0.29 [0.08, 1.04]	2003
Zhang, J.2003	4	25	8	28	0.7%	0.48 [0.12, 1.83]	2003
Duan, R.2003	10	76	37	76	3.4%	0.16 [0.07, 0.36]	2003
Li, J.2003	4	23	10	21	0.9%	0.23 [0.06, 0.92]	2003
Wang, C.2003	3	15	7	15	0.6%	0.29 [0.06, 1.45]	2003
Wang, C. 2004	5	23	12	22	1.0%	0.23 [0.06, 0.85]	2004
Zhu, F.2004	6	34	15	30	1.4%	0.21 [0.07, 0.67]	2004
Chen, H.2004	3	24	10	24	0.9%	0.20 [0.05, 0.86]	2004
Mao, W. 2004	4	24	7	16	0.7%	0.26 [0.06, 1.11]	2004
Zhang, T. 2005	4	23	10	21	0.9%	0.23 [0.06, 0.92]	2005
Chen, J.2005	2	27	7	24	0.7%	0.19 [0.04, 1.05]	2005
Huang, Y.2005	4	27	9	21	0.9%	0.23 [0.06, 0.91]	2005
Ma, D.2005	3	24	10	24	0.9%	0.20 [0.05, 0.86]	2005
Xiong, C.2006	4	22	12	22	1.1%	0.19 [0.05, 0.73]	2006
Zhang, Z.2006	2	16	9	16	0.8%	0.11 [0.02, 0.66]	2006
Zhao, Z.2006	5	32	14	32	1.3%	0.24 [0.07, 0.78]	2006
Pan, Y.2006	3	28	10	20	1.1%	0.12 [0.03, 0.53]	2006
Wang, J.2006	4	30	7	30	0.6%	0.51 [0.13, 1.95]	2006
Chen, Z.2006	3	12	6	11	0.5%	0.28 [0.05, 1.62]	2006
Sun, C.2007	3	24	12	24	1.1%	0.14 [0.03, 0.61]	2007
Yin, W.2007	3	19	10	19	0.9%	0.17 [0.04, 0.78]	2007
Zhou, F.2007	2	16	7	12	0.7%	0.10 [0.02, 0.66]	2007
Zheng, F.2007	6	24	8	20	0.7%	0.50 [0.14, 1.81]	2007
Li, X.2008	4	25	11	25	1.0%	0.24 [0.06, 0.92]	2008
Gao, H.2009	7	35	17	30	1.6%	0.19 [0.06, 0.57]	2009
Zhang, C. 2011	1	19	9	19	0.9%	0.06 [0.01, 0.56]	2011
Yu, D.2012	3	25	7	25	0.7%	0.35 [0.08, 1.55]	2012
Qin, Q.2012	4	34	23	34	2.2%	0.06 [0.02, 0.23]	2012
Xing, Q.2012	2	19	12	19	1.1%	0.07 [0.01, 0.39]	2012
Li, L. 2013	3	15	7	15	0.6%	0.29 [0.06, 1.45]	2013
Zhang, J.2014	8	40	25	40	2.1%	0.15 [0.05, 0.41]	2014
Chen, J. 2014	5	30	15	30	1.3%	0.20 [0.06, 0.66]	2014
Tu, D.2014	4	38	13	38	1.2%	0.23 [0.07, 0.78]	2014
Zhou, Z.2015	4	21	11	21	1.0%	0.21 [0.05, 0.85]	2015
Cai,C.2015	3	21	7	21	0.6%	0.33 [0.07, 1.53]	2015
Zhang, Z.2015	2	45	18	45	1.8%	0.07 [0.01, 0.32]	2015
Yan, L.2016	4	33	8	28	0.8%	0.34 [0.09, 1.30]	2016
Wang, Y. 2018	4	37	10	37	1.0%	0.33 [0.09, 1.16]	2018
Wu, Y. 2019	10	46	27	46	2.3%	0.20 [0.08, 0.49]	2019
**Subtotal (95% CI)**		**1368**		**1272**	**54.1%**	**0.20 [0.17, 0.24]**	
Total events	199		575				
Heterogeneity: Chi² = 25.03, df = 47 (P = 1.00); I² = 0%							
Test for overall effect: Z = 16.63 (P < 0.00001)							
**Total (95% CI)**		**2539**		**2385**	**100.0%**	**0.20 [0.18, 0.23]**	
Total events	362		1066				
Heterogeneity: Chi² = 43.41, df = 78 (P = 1.00); I² = 0%							
Test for overall effect: Z = 22.57 (P < 0.00001)							
Test for subgroup differences: Chi² = 2.12, df = 2 (P = 0.35), I² = 5.6%							

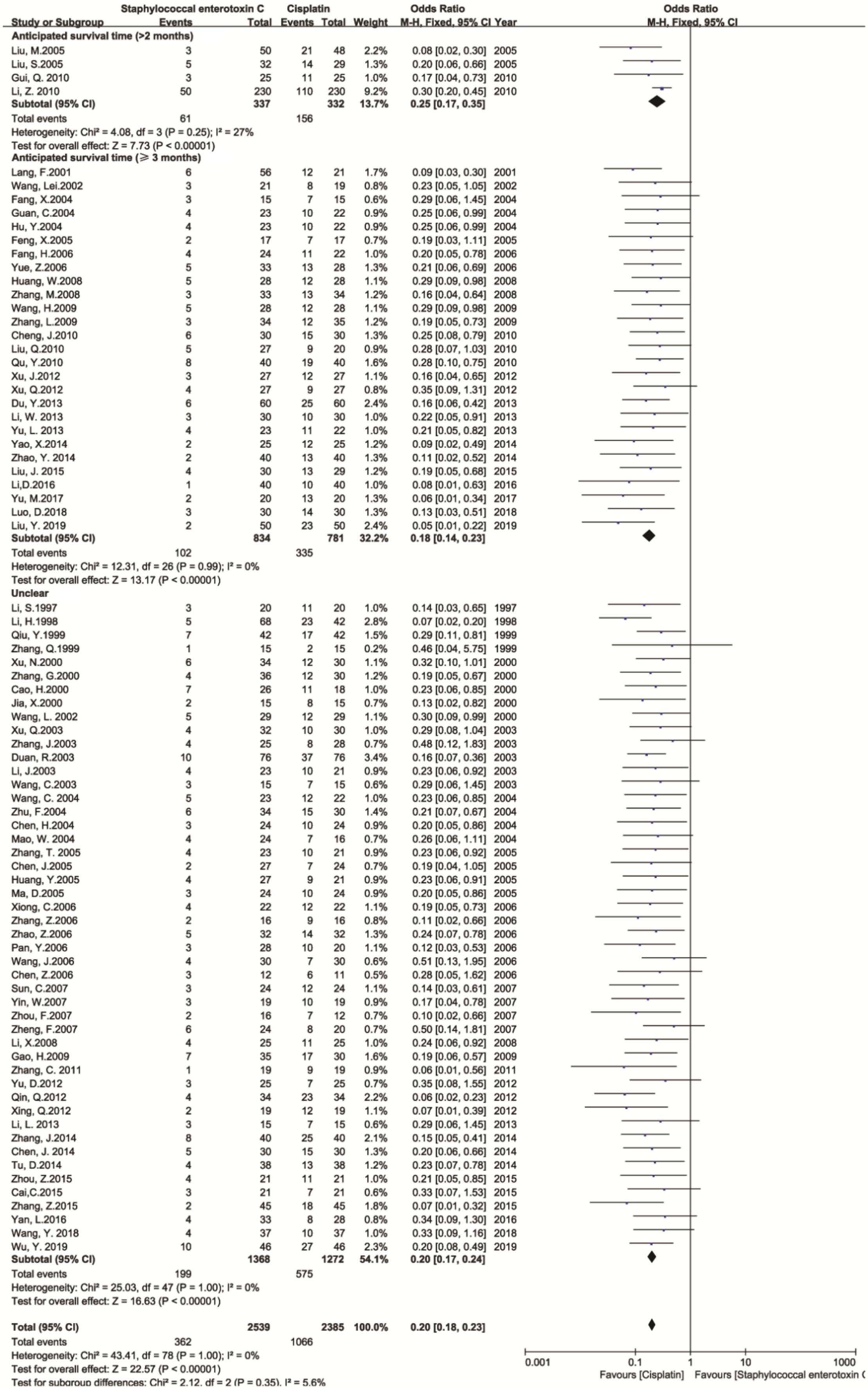

Fig.S32 Subgroups analysis of treatment failure via anticipated survival time

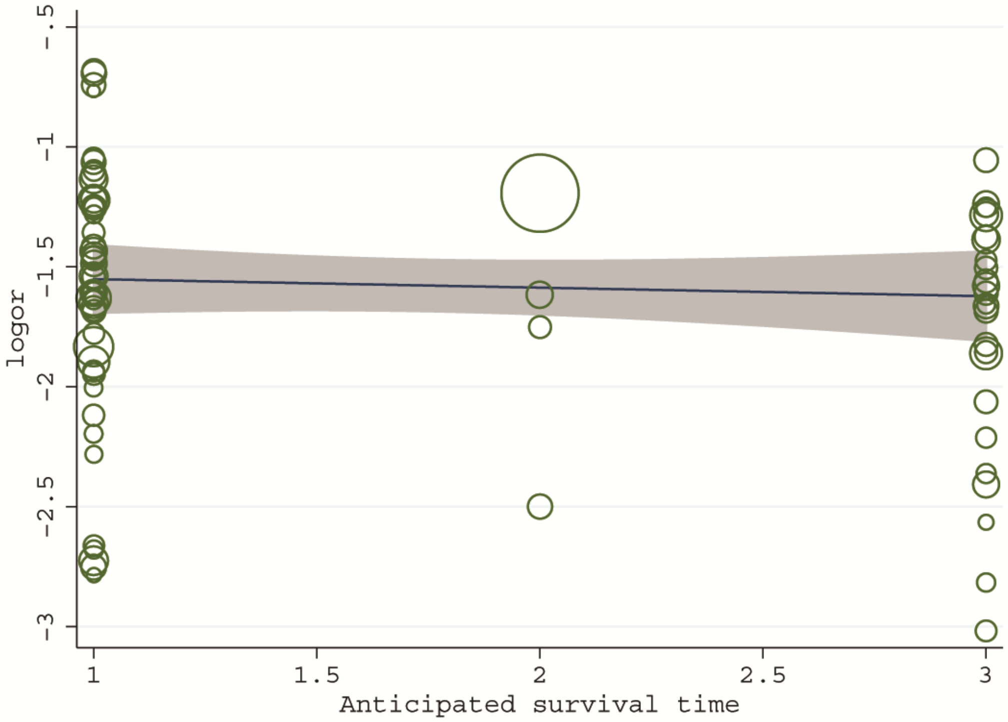

Fig.S33 Meta regression of treatment failure via anticipated survival time

Study or Subgroup	Staphylococcal enterotoxin C Events	Staphylococcal enterotoxin C Total	Cisplatin Events	Cisplatin Total	Weight	Odds Ratio M-H, Fixed, 95% CI	Year
**Indwelling pleural catheter**							
Qiu, Y.1999	12	42	7	42	1.7%	2.00 [0.70, 5.73]	1999
Zhang, Q.1999	10	15	4	15	0.5%	5.50 [1.15, 26.41]	1999
Duan, R.2003	27	76	16	76	3.6%	2.07 [1.00, 4.26]	2003
Xu, Q.2003	20	32	12	30	1.6%	2.50 [0.90, 6.95]	2003
Zhang, J.2003	9	25	2	28	0.4%	7.31 [1.40, 38.22]	2003
Chen, H.2004	13	24	6	24	0.9%	3.55 [1.04, 12.06]	2004
Fang, X.2004	3	15	1	15	0.3%	3.50 [0.32, 38.23]	2004
Guan, C.2004	13	23	6	22	0.9%	3.47 [0.99, 12.09]	2004
Zhu, F.2004	16	34	6	30	1.2%	3.56 [1.16, 10.89]	2004
Liu, S.2005	13	32	6	29	1.3%	2.62 [0.84, 8.22]	2005
Ma, D.2005	13	24	6	24	0.9%	3.55 [1.04, 12.06]	2005
Zhang, T. 2005	8	23	5	21	1.2%	1.71 [0.46, 6.39]	2005
Feng, X.2005	6	17	4	17	0.9%	1.77 [0.40, 7.93]	2005
Chen, J.2005	11	27	5	24	1.1%	2.61 [0.75, 9.11]	2005
Liu, M.2005	35	50	14	48	1.5%	5.67 [2.38, 13.50]	2005
Xiong, C.2006	10	22	6	22	1.1%	2.22 [0.63, 7.82]	2006
Zhang, Z.2006	6	16	2	16	0.4%	4.20 [0.70, 25.26]	2006
Zhao, Z.2006	9	32	5	32	1.2%	2.11 [0.62, 7.20]	2006
Fang, H.2006	6	24	2	22	0.5%	3.33 [0.60, 18.66]	2006
Pan, Y.2006	16	28	4	20	0.7%	5.33 [1.41, 20.10]	2006
Chen, Z.2006	4	12	2	11	0.5%	2.25 [0.32, 15.76]	2006
Sun, C.2007	13	24	4	24	0.6%	5.91 [1.55, 22.58]	2007
Yin, W.2007	6	19	4	19	0.9%	1.73 [0.40, 7.51]	2007
Zhou, F.2007	5	16	0	12	0.1%	11.96 [0.59, 241.06]	2007
Gao, H.2009	17	35	6	30	1.1%	3.78 [1.24, 11.50]	2009
Qu, Y.2010	4	40	2	40	0.6%	2.11 [0.36, 12.24]	2010
Cheng, J.2010	5	30	0	30	0.1%	13.16 [0.69, 249.48]	2010
Gui, Q. 2010	9	25	3	25	0.7%	4.13 [0.96, 17.70]	2010
Li, Z. 2010	100	230	70	230	13.7%	1.76 [1.20, 2.58]	2010
Liu, Q.2010	5	27	3	20	1.0%	1.29 [0.27, 6.16]	2010
Zhang, C. 2011	13	19	4	19	0.4%	8.13 [1.87, 35.23]	2011
Yu, D.2012	17	25	12	25	1.3%	2.30 [0.73, 7.27]	2012
Qin, Q.2012	19	34	6	34	0.9%	5.91 [1.94, 17.97]	2012
Li, L. 2013	4	15	0	15	0.1%	12.13 [0.59, 248.49]	2013
Yu, L. 2013	2	23	1	22	0.3%	2.00 [0.17, 23.78]	2013
Yao, X.2014	13	25	7	25	1.2%	2.79 [0.86, 9.01]	2014
Zhou, Z.2015	6	21	1	21	0.2%	8.00 [0.87, 73.68]	2015
Liu, J. 2015	15	30	7	29	1.2%	3.14 [1.03, 9.55]	2015
Wang, Y. 2018	20	37	17	37	2.7%	1.38 [0.55, 3.45]	2018
**Subtotal (95% CI)**		**1268**		**1225**	**49.8%**	**2.74 [2.29, 3.29]**	
Total events	533		268				
Heterogeneity: Chi² = 27.40, df = 38 (P = 0.90); I² = 0%							
Test for overall effect: Z = 10.89 (P < 0.00001)							
**Thoracentesis**							
Li, H.1998	18	68	6	42	1.9%	2.16 [0.78, 5.98]	1998
Zhang, G.2000	20	36	6	30	1.0%	5.00 [1.65, 15.17]	2000
Jia, X.2000	6	15	2	15	0.4%	4.33 [0.71, 26.53]	2000
Lang, F.2001	36	56	6	21	1.1%	4.50 [1.51, 13.43]	2001
Wang, Lei.2002	11	21	7	19	1.2%	1.89 [0.53, 6.69]	2002
Li, J.2003	7	23	4	21	1.0%	1.86 [0.46, 7.58]	2003
Wang, C.2003	5	15	2	15	0.5%	3.25 [0.52, 20.37]	2003
Hu, Y.2004	13	23	6	22	0.9%	3.47 [0.99, 12.09]	2004
Huang, Y.2005	9	27	4	21	1.0%	2.13 [0.55, 8.21]	2005
Yue, Z.2006	10	33	3	28	0.8%	3.62 [0.89, 14.83]	2006
Huang, W.2008	12	28	9	28	1.8%	1.58 [0.53, 4.71]	2008
Zhang, M.2008	11	33	4	34	0.9%	3.75 [1.05, 13.35]	2008
Li, X.2008	8	25	6	25	1.4%	1.49 [0.43, 5.17]	2008
Zhang, L.2009	11	34	4	35	0.9%	3.71 [1.05, 13.13]	2009
Wang, H.2009	12	28	9	28	1.8%	1.58 [0.53, 4.71]	2009
Xu, J.2012	14	27	9	27	1.5%	2.15 [0.72, 6.47]	2012
Xu, Q.2012	11	27	9	27	1.8%	1.38 [0.45, 4.17]	2012
Du, Y.2013	31	60	22	60	3.7%	1.85 [0.89, 3.83]	2013
Li, W. 2013	13	30	11	30	2.2%	1.32 [0.47, 3.72]	2013
Chen, J. 2014	6	30	2	30	0.6%	3.50 [0.65, 18.98]	2014
Zhang, Z.2015	28	45	17	45	2.2%	2.71 [1.16, 6.36]	2015
Li,D.2016	27	40	19	40	2.1%	2.30 [0.93, 5.69]	2016
**Subtotal (95% CI)**		**724**		**643**	**30.6%**	**2.36 [1.86, 3.00]**	
Total events	319		167				
Heterogeneity: Chi² = 10.10, df = 21 (P = 0.98); I² = 0%							
Test for overall effect: Z = 7.02 (P < 0.00001)							
**Unclear**							
Li, S.1997	10	20	3	20	0.5%	5.67 [1.25, 25.61]	1997
Xu, N.2000	10	34	8	30	2.1%	1.15 [0.38, 3.43]	2000
Cao, H.2000	6	26	1	18	0.3%	5.10 [0.56, 46.65]	2000
Wang, L. 2002	7	29	4	29	1.0%	1.99 [0.51, 7.71]	2000
Mao, W. 2004	8	24	2	16	0.6%	3.50 [0.63, 19.30]	2004
Zheng, F.2007	4	24	3	20	0.9%	1.13 [0.22, 5.79]	2007
Xing, Q.2012	7	19	3	19	0.7%	3.11 [0.66, 14.60]	2012
Zhang, J.2014	13	40	6	40	1.4%	2.73 [0.92, 8.13]	2014
Zhao, Y. 2014	25	40	16	40	2.1%	2.50 [1.02, 6.15]	2014
Tu, D.2014	14	38	14	38	3.1%	1.00 [0.39, 2.54]	2014
Cai,C.2015	11	21	6	21	1.0%	2.75 [0.77, 9.86]	2015
Yan, L.2016	16	33	7	28	1.3%	2.82 [0.95, 8.44]	2016
Yu, M.2017	7	20	3	20	0.7%	3.05 [0.66, 14.14]	2017
Luo, D.2018	12	30	6	30	1.2%	2.67 [0.84, 8.46]	2018
Liu, Y. 2019	28	50	7	50	1.1%	7.82 [2.95, 20.72]	2019
Wu, Y. 2019	15	46	7	46	1.6%	2.70 [0.98, 7.43]	2019
**Subtotal (95% CI)**		**494**		**465**	**19.6%**	**2.59 [1.93, 3.48]**	
Total events	193		96				
Heterogeneity: Chi² = 13.87, df = 15 (P = 0.54); I² = 0%							
Test for overall effect: Z = 6.33 (P < 0.00001)							
**Total (95% CI)**		**2486**		**2333**	**100.0%**	**2.59 [2.28, 2.95]**	
Total events	1045		531				
Heterogeneity: Chi² = 52.12, df = 76 (P = 0.98); I² = 0%							
Test for overall effect: Z = 14.40 (P < 0.00001)							
Test for subgroup differences: Chi² = 0.94, df = 2 (P = 0.62), I² = 0%							

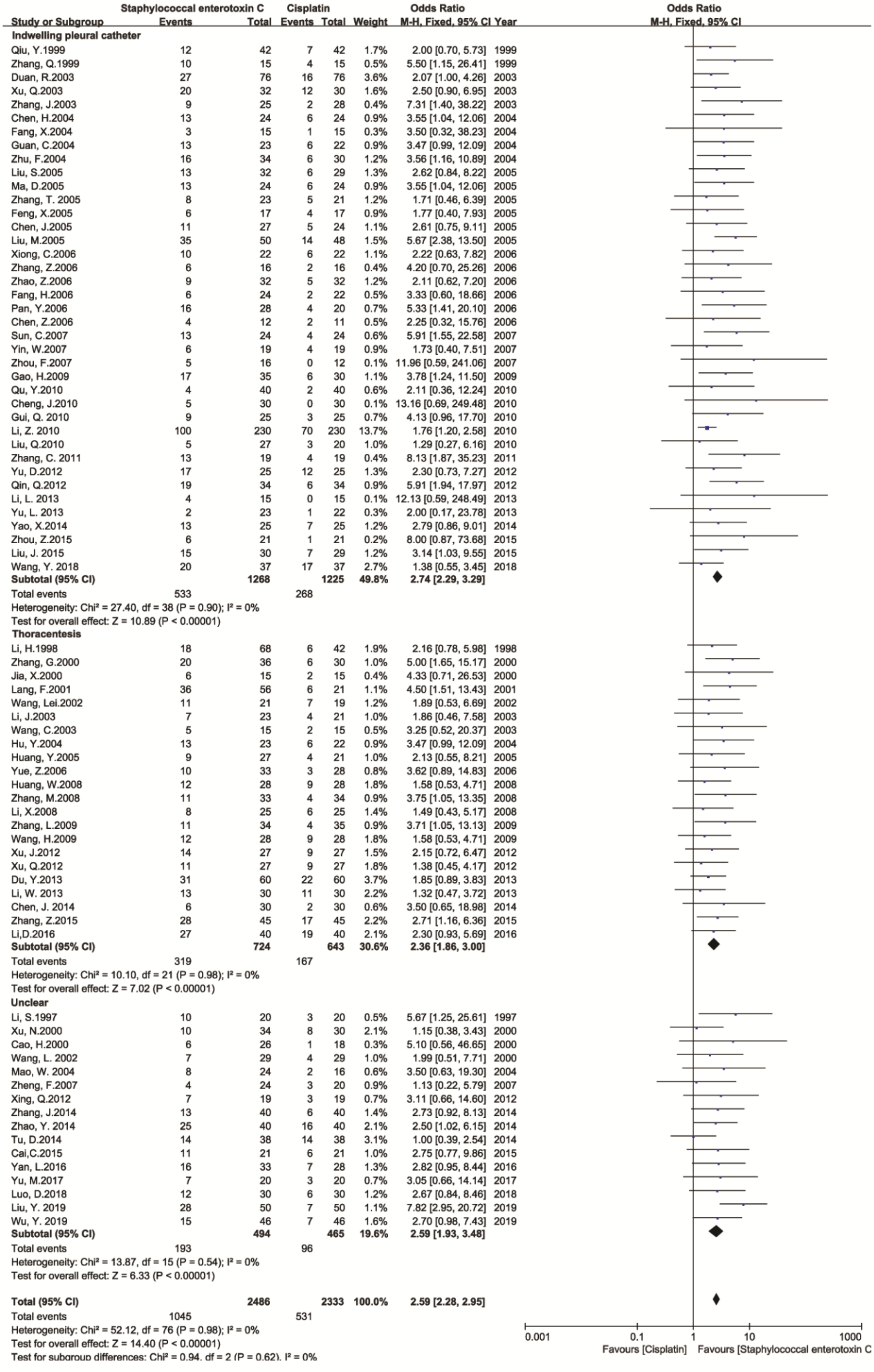

Fig.S34 Subgroups analysis of complete response via indwelling pleural catheter

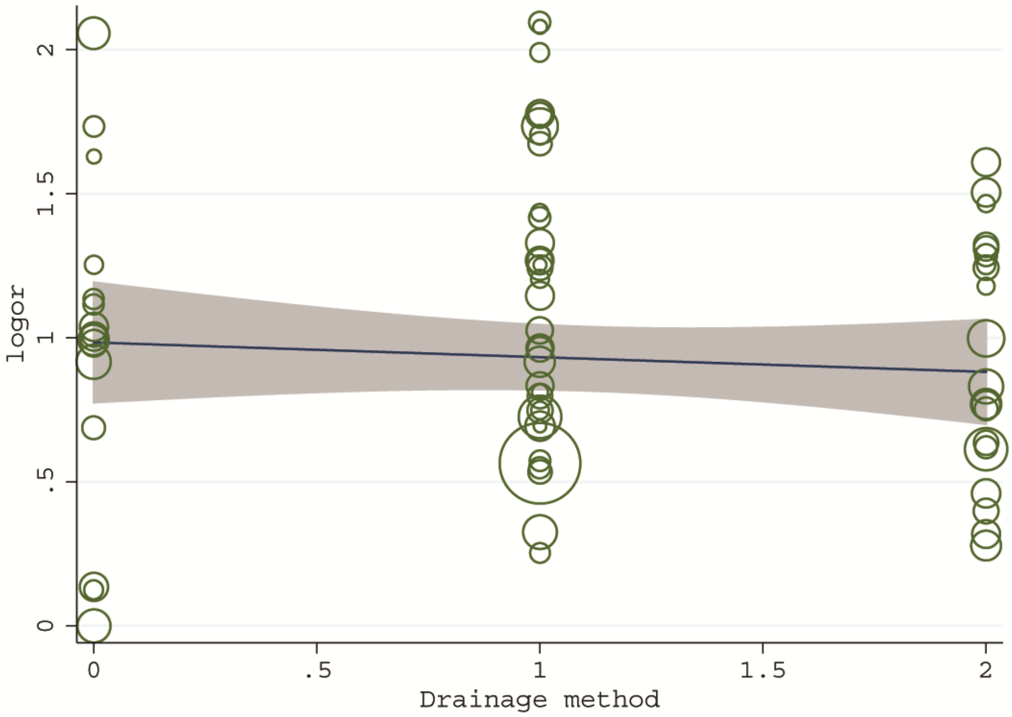

Fig.S35 Meta regression of complete response via indwelling pleural catheter

Study or Subgroup	Staphylococcal enterotoxin C Events	Staphylococcal enterotoxin C Total	Cisplatin Events	Cisplatin Total	Weight	Odds Ratio M-H, Fixed, 95% CI	Year
**Indwelling pleural catheter**							
Qiu, Y.1999	7	42	17	42	1.5%	0.29 [0.11, 0.81]	1999
Zhang, Q.1999	1	15	2	15	0.2%	0.46 [0.04, 5.75]	1999
Xu, Q.2003	4	32	10	30	1.0%	0.29 [0.08, 1.04]	2003
Zhang, J.2003	4	25	8	28	0.7%	0.48 [0.12, 1.83]	2003
Duan, R.2003	10	76	37	76	3.4%	0.16 [0.07, 0.36]	2003
Zhu, F.2004	6	34	15	30	1.4%	0.21 [0.07, 0.67]	2004
Chen, H.2004	3	24	10	24	0.9%	0.20 [0.05, 0.86]	2004
Fang, X.2004	3	15	7	15	0.6%	0.29 [0.06, 1.45]	2004
Guan, C.2004	4	23	10	22	0.9%	0.25 [0.06, 0.99]	2004
Zhang, T. 2005	4	23	10	21	0.9%	0.23 [0.06, 0.92]	2005
Chen, J.2005	2	27	7	24	0.7%	0.19 [0.04, 1.05]	2005
Feng, X.2005	2	17	7	17	0.7%	0.19 [0.03, 1.11]	2005
Liu, S.2005	5	32	14	29	1.3%	0.20 [0.06, 0.66]	2005
Ma, D.2005	3	24	10	24	0.9%	0.20 [0.05, 0.86]	2005
Liu, M.2005	3	50	21	48	2.2%	0.08 [0.02, 0.30]	2005
Xiong, C.2006	4	22	12	22	1.1%	0.19 [0.05, 0.73]	2006
Zhang, Z.2006	2	16	9	16	0.8%	0.11 [0.02, 0.66]	2006
Zhao, Z.2006	5	32	14	32	1.3%	0.24 [0.07, 0.78]	2006
Fang, H.2006	4	24	11	22	1.0%	0.20 [0.05, 0.78]	2006
Pan, Y.2006	3	28	10	20	1.1%	0.12 [0.03, 0.53]	2006
Wang, J.2006	4	30	7	30	0.6%	0.51 [0.13, 1.95]	2006
Chen, Z.2006	3	12	6	11	0.5%	0.28 [0.05, 1.62]	2006
Sun, C.2007	3	24	12	24	1.1%	0.14 [0.03, 0.61]	2007
Yin, W.2007	3	19	10	19	0.9%	0.17 [0.04, 0.78]	2007
Zhou, F.2007	2	16	7	12	0.7%	0.10 [0.02, 0.66]	2007
Gao, H.2009	7	35	17	30	1.6%	0.19 [0.06, 0.57]	2009
Liu, Q.2010	5	27	9	20	0.9%	0.28 [0.07, 1.03]	2010
Gui, Q. 2010	3	25	11	25	1.0%	0.17 [0.04, 0.73]	2010
Cheng, J.2010	6	30	15	30	1.3%	0.25 [0.08, 0.79]	2010
Qu, Y.2010	8	40	19	40	1.6%	0.28 [0.10, 0.75]	2010
Li, Z. 2010	50	230	110	230	9.2%	0.30 [0.20, 0.45]	2010
Zhang, C. 2011	1	19	9	19	0.9%	0.06 [0.01, 0.56]	2011
Yu, D.2012	3	25	7	25	0.7%	0.35 [0.08, 1.55]	2012
Qin, Q.2012	4	34	23	34	2.2%	0.06 [0.02, 0.23]	2012
Li, L. 2013	3	15	7	15	0.6%	0.29 [0.06, 1.45]	2013
Yu, L. 2013	4	23	11	22	1.0%	0.21 [0.05, 0.82]	2013
Yao, X.2014	2	25	12	25	1.2%	0.09 [0.02, 0.49]	2014
Liu, J. 2015	4	30	13	29	1.2%	0.19 [0.05, 0.68]	2015
Zhou, Z.2015	4	21	11	21	1.0%	0.21 [0.05, 0.85]	2015
Wang, Y. 2018	4	37	10	37	1.0%	0.33 [0.09, 1.16]	2018
**Subtotal (95% CI)**		**1298**		**1255**	**51.9%**	**0.22 [0.18, 0.27]**	
Total events	202		567				
Heterogeneity: Chi² = 18.88, df = 39 (P = 1.00); I² = 0%							
Test for overall effect: Z = 15.76 (P < 0.00001)							
**Thoracentesis**							
Li, H.1998	5	68	23	42	2.8%	0.07 [0.02, 0.20]	1998
Zhang, G.2000	4	36	12	30	1.2%	0.19 [0.05, 0.67]	2000
Jia, X.2000	2	15	8	15	0.7%	0.13 [0.02, 0.82]	2000
Lang, F.2001	6	56	12	21	1.7%	0.09 [0.03, 0.30]	2001
Wang, Lei.2002	3	21	8	19	0.8%	0.23 [0.05, 1.05]	2002
Li, J.2003	4	23	10	21	0.9%	0.23 [0.06, 0.92]	2003
Wang, C.2003	3	15	7	15	0.6%	0.29 [0.06, 1.45]	2003
Wang, C. 2004	5	23	12	22	1.0%	0.23 [0.06, 0.85]	2004
Hu, Y.2004	4	23	10	22	0.9%	0.25 [0.06, 0.99]	2004
Huang, Y.2005	4	27	9	21	0.9%	0.23 [0.06, 0.91]	2005
Yue, Z.2006	5	33	13	28	1.3%	0.21 [0.06, 0.69]	2006
Li, X.2008	4	25	11	25	1.0%	0.24 [0.06, 0.92]	2008
Huang, W.2008	5	28	12	28	1.1%	0.29 [0.09, 0.98]	2008
Zhang, M.2008	3	33	13	34	1.2%	0.16 [0.04, 0.64]	2008
Wang, H.2009	5	28	12	28	1.1%	0.29 [0.09, 0.98]	2009
Zhang, L.2009	3	34	12	35	1.2%	0.19 [0.05, 0.73]	2009
Xu, J.2012	3	27	12	27	1.1%	0.16 [0.04, 0.65]	2012
Xu, Q.2012	4	27	9	27	0.8%	0.35 [0.09, 1.31]	2012
Li, W. 2013	3	30	10	30	1.0%	0.22 [0.05, 0.91]	2013
Du, Y.2013	6	60	25	60	2.4%	0.16 [0.06, 0.42]	2013
Chen, J. 2014	5	30	15	30	1.3%	0.20 [0.06, 0.66]	2014
Zhang, Z.2015	2	45	18	45	1.8%	0.07 [0.01, 0.32]	2015
Li,D.2016	1	40	10	40	1.0%	0.08 [0.01, 0.63]	2016
**Subtotal (95% CI)**		**747**		**665**	**27.9%**	**0.18 [0.14, 0.23]**	
Total events	89		283				
Heterogeneity: Chi² = 10.40, df = 22 (P = 0.98); I² = 0%							
Test for overall effect: Z = 12.36 (P < 0.00001)							
**Unclear**							
Li, S.1997	3	20	11	20	1.0%	0.14 [0.03, 0.65]	1997
Wang, L. 2002	5	29	12	29	1.1%	0.30 [0.09, 0.99]	2000
Xu, N.2000	6	34	12	30	1.1%	0.32 [0.10, 1.01]	2000
Cao, H.2000	7	26	11	18	1.0%	0.23 [0.06, 0.85]	2000
Mao, W. 2004	4	24	7	16	0.7%	0.26 [0.06, 1.11]	2004
Zheng, F.2007	6	24	8	20	0.7%	0.50 [0.14, 1.81]	2007
Xing, Q.2012	2	19	12	19	1.1%	0.07 [0.01, 0.39]	2012
Zhang, J.2014	8	40	25	40	2.1%	0.15 [0.05, 0.41]	2014
Zhao, Y. 2014	2	40	13	40	1.3%	0.11 [0.02, 0.52]	2014
Tu, D.2014	4	38	13	38	1.2%	0.23 [0.07, 0.78]	2014
Cai,C.2015	3	21	7	21	0.6%	0.33 [0.07, 1.53]	2015
Yan, L.2016	4	33	8	28	0.8%	0.34 [0.09, 1.30]	2016
Yu, M.2017	2	20	13	20	1.3%	0.06 [0.01, 0.34]	2017
Luo, D.2018	3	30	14	30	1.3%	0.13 [0.03, 0.51]	2018
Wu, Y. 2019	10	46	27	46	2.3%	0.20 [0.08, 0.49]	2019
Liu, Y. 2019	2	50	23	50	2.4%	0.05 [0.01, 0.22]	2019
**Subtotal (95% CI)**		**494**		**465**	**20.2%**	**0.18 [0.13, 0.25]**	
Total events	71		216				
Heterogeneity: Chi² = 12.45, df = 15 (P = 0.64); I² = 0%							
Test for overall effect: Z = 10.43 (P < 0.00001)							
**Total (95% CI)**		**2539**		**2385**	**100.0%**	**0.20 [0.18, 0.23]**	
Total events	362		1066				
Heterogeneity: Chi² = 43.41, df = 78 (P = 1.00); I² = 0%							
Test for overall effect: Z = 22.57 (P < 0.00001)							
Test for subgroup differences: Chi² = 1.96, df = 2 (P = 0.38), I² = 0%							

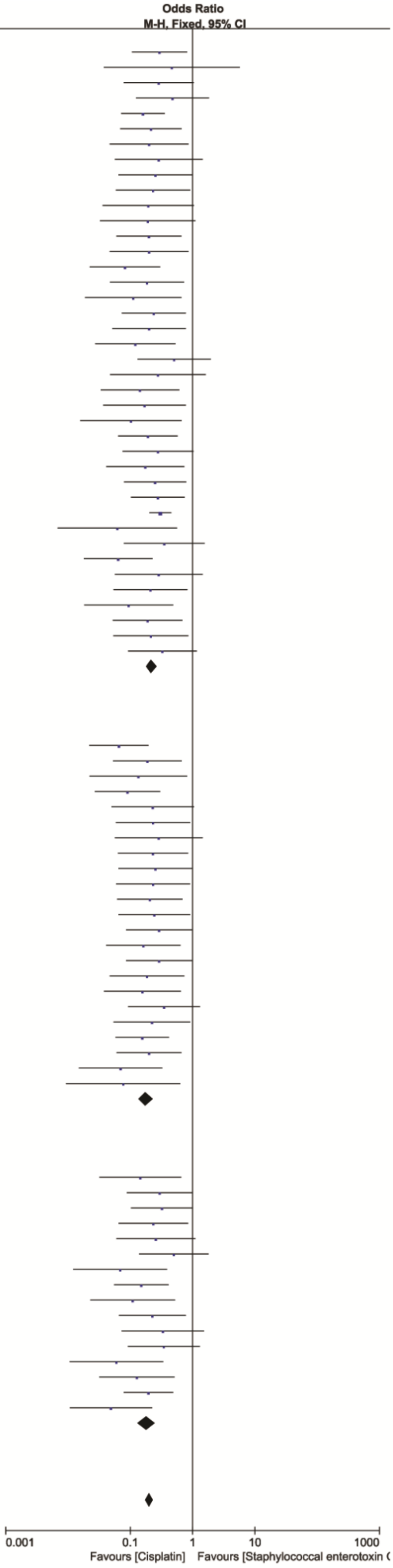

Fig.S36 Subgroups analysis of treatment failure via indwelling pleural catheter

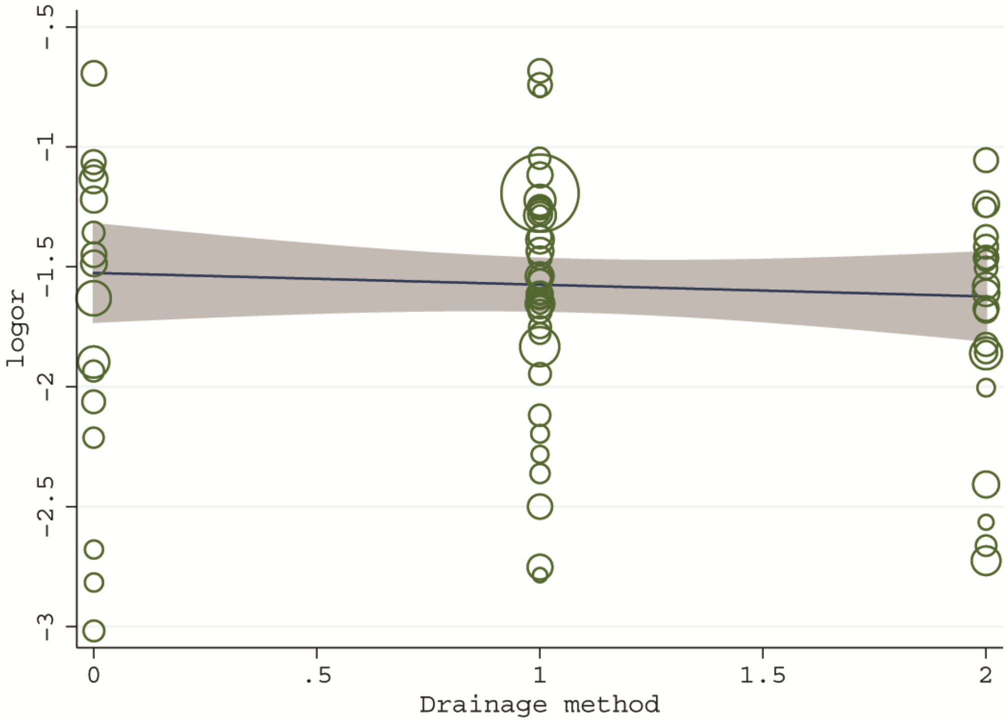

Fig.S37 Meta regression of treatment failure via indwelling pleural catheter

	Staphylococcal enterotoxin C		Cisplatin			Odds Ratio	
**Study or Subgroup**	**Events**	**Total**	**Events**	**Total**	**Weight**	**M-H, Fixed, 95% CI**	**Year**
**Staphylococcal enterotoxin C (8ml,80ng,2000IU)**							
Jia, X.2000	6	15	2	15	0.4%	4.33 [0.71, 26.53]	2000
Zhang, T. 2005	8	23	5	21	1.2%	1.71 [0.46, 6.39]	2005
Huang, Y.2005	9	27	4	21	1.0%	2.13 [0.55, 8.21]	2005
Zhao, Z.2006	9	32	5	32	1.2%	2.11 [0.62, 7.20]	2006
Pan, Y.2006	16	28	4	20	0.7%	5.33 [1.41, 20.10]	2006
Li, X.2008	8	25	6	25	1.4%	1.49 [0.43, 5.17]	2008
Li, Z. 2010	100	230	70	230	13.7%	1.76 [1.20, 2.58]	2010
Li, L. 2013	4	15	0	15	0.1%	12.13 [0.59, 248.49]	2013
Yu, L. 2013	2	23	1	22	0.3%	2.00 [0.17, 23.78]	2013
Wang, Y. 2018	20	37	17	37	2.7%	1.38 [0.55, 3.45]	2018
**Subtotal (95% CI)**		**455**		**438**	**22.8%**	**1.95 [1.46, 2.60]**	
Total events	182		114				
Heterogeneity: Chi² = 5.43, df = 9 (P = 0.80); I² = 0%							
Test for overall effect: Z = 4.49 (P < 0.00001)							
**Staphylococcal enterotoxin C (10ml,100ng,2500IU)**							
Wang, L. 2002	7	29	4	29	1.0%	1.99 [0.51, 7.71]	2000
Mao, W. 2004	8	24	2	16	0.6%	3.50 [0.63, 19.30]	2004
Yue, Z.2006	10	33	3	28	0.8%	3.62 [0.89, 14.83]	2006
Zheng, F.2007	4	24	3	20	0.9%	1.13 [0.22, 5.79]	2007
Zhou, Z.2015	6	21	1	21	0.2%	8.00 [0.87, 73.68]	2015
**Subtotal (95% CI)**		**131**		**114**	**3.6%**	**2.77 [1.38, 5.54]**	
Total events	35		13				
Heterogeneity: Chi² = 2.47, df = 4 (P = 0.65); I² = 0%							
Test for overall effect: Z = 2.88 (P = 0.004)							
**Staphylococcal enterotoxin C (12ml,120ng,3000IU)**							
Duan, R.2003	27	76	16	76	3.6%	2.07 [1.00, 4.26]	2003
Chen, H.2004	13	24	6	24	0.9%	3.55 [1.04, 12.06]	2004
Fang, X.2004	3	15	1	15	0.3%	3.50 [0.32, 38.23]	2004
Ma, D.2005	13	24	6	24	0.9%	3.55 [1.04, 12.06]	2005
Zhang, M.2008	11	33	4	34	0.9%	3.75 [1.05, 13.35]	2008
Zhang, L.2009	11	34	4	35	0.9%	3.71 [1.05, 13.13]	2009
Qu, Y.2010	4	40	2	40	0.6%	2.11 [0.36, 12.24]	2010
**Subtotal (95% CI)**		**246**		**248**	**8.2%**	**2.83 [1.81, 4.42]**	
Total events	82		39				
Heterogeneity: Chi² = 1.49, df = 6 (P = 0.96); I² = 0%							
Test for overall effect: Z = 4.58 (P < 0.00001)							
**Staphylococcal enterotoxin C (16ml,160ng,4000IU)**							
Li, S.1997	10	20	3	20	0.5%	5.67 [1.25, 25.61]	1997
Zhang, Q.1999	10	15	4	15	0.5%	5.50 [1.15, 26.41]	1999
Xu, N.2000	10	34	8	30	2.1%	1.15 [0.38, 3.43]	2000
Wang, C.2003	5	15	2	15	0.5%	3.25 [0.52, 20.37]	2003
Chen, Z.2006	4	12	2	11	0.5%	2.25 [0.32, 15.76]	2006
Yin, W.2007	6	19	4	19	0.9%	1.73 [0.40, 7.51]	2007
Gao, H.2009	17	35	6	30	1.1%	3.78 [1.24, 11.50]	2009
Liu, Q.2010	5	27	3	20	1.0%	1.29 [0.27, 6.16]	2010
**Subtotal (95% CI)**		**177**		**160**	**7.0%**	**2.50 [1.53, 4.10]**	
Total events	67		32				
Heterogeneity: Chi² = 5.60, df = 7 (P = 0.59); I² = 0%							
Test for overall effect: Z = 3.63 (P = 0.0003)							
**Staphylococcal enterotoxin C (20ml,200ng,5000IU)**							
Cao, H.2000	6	26	1	18	0.3%	5.10 [0.56, 46.65]	2000
Lang, F.2001	36	56	6	21	1.1%	4.50 [1.51, 13.43]	2001
Wang, Lei.2002	11	21	7	19	1.2%	1.89 [0.53, 6.69]	2002
Li, J.2003	7	23	4	21	1.0%	1.86 [0.46, 7.58]	2003
Xu, Q.2003	20	32	12	30	1.6%	2.50 [0.90, 6.95]	2003
Zhang, J.2003	9	25	2	28	0.4%	7.31 [1.40, 38.22]	2003
Guan, C.2004	13	23	6	22	0.9%	3.47 [0.99, 12.09]	2004
Hu, Y.2004	13	23	6	22	0.9%	3.47 [0.99, 12.09]	2004
Feng, X.2005	6	17	4	17	0.9%	1.77 [0.40, 7.93]	2005
Liu, M.2005	35	50	14	48	1.5%	5.67 [2.38, 13.50]	2005
Fang, H.2006	6	24	2	22	0.5%	3.33 [0.60, 18.66]	2006
Sun, C.2007	13	24	4	24	0.6%	5.91 [1.55, 22.58]	2007
Cheng, J.2010	5	30	0	30	0.1%	13.16 [0.69, 249.48]	2010
Gui, Q. 2010	9	25	3	25	0.7%	4.13 [0.96, 17.70]	2010
Zhang, C. 2011	13	19	4	19	0.4%	8.13 [1.87, 35.23]	2011
Qin, Q.2012	19	34	6	34	0.9%	5.91 [1.94, 17.97]	2012
Zhao, Y. 2014	25	40	16	40	2.1%	2.50 [1.02, 6.15]	2014
Tu, D.2014	14	38	14	38	3.1%	1.00 [0.39, 2.54]	2014
Yao, X.2014	13	25	7	25	1.2%	2.79 [0.86, 9.01]	2014
Zhang, J.2014	13	40	6	40	1.4%	2.73 [0.92, 8.13]	2014
Cai,C.2015	11	21	6	21	1.0%	2.75 [0.77, 9.86]	2015
Yan, L.2016	16	33	7	28	1.3%	2.82 [0.95, 8.44]	2016
Yu, M.2017	7	20	3	20	0.7%	3.05 [0.66, 14.14]	2017
Luo, D.2018	12	30	6	30	1.2%	2.67 [0.84, 8.46]	2018
Liu, Y. 2019	28	50	7	50	1.1%	7.82 [2.95, 20.72]	2019
Wu, Y. 2019	15	46	7	46	1.6%	2.70 [0.98, 7.43]	2019
**Subtotal (95% CI)**		**795**		**738**	**27.8%**	**3.34 [2.65, 4.22]**	
Total events	375		160				
Heterogeneity: Chi² = 19.69, df = 25 (P = 0.76); I² = 0%							
Test for overall effect: Z = 10.20 (P < 0.00001)							
**Staphylococcal enterotoxin C (10-20ml,100-200ng,2500-5000IU)**							
Chen, J.2005	11	27	5	24	1.1%	2.61 [0.75, 9.11]	2005
Zhang, Z.2006	6	16	2	16	0.4%	4.20 [0.70, 25.26]	2006
Xiong, C.2006	10	22	6	22	1.1%	2.22 [0.63, 7.82]	2006
Huang, W.2008	12	28	9	28	1.8%	1.58 [0.53, 4.71]	2008
Wang, H.2009	12	28	9	28	1.8%	1.58 [0.53, 4.71]	2009
Xu, J.2012	14	27	9	27	1.5%	2.15 [0.72, 6.47]	2012
Xu, Q.2012	11	27	9	27	1.8%	1.38 [0.45, 4.17]	2012
Yu, D.2012	17	25	12	25	1.3%	2.30 [0.73, 7.27]	2012
Du, Y.2013	31	60	22	60	3.7%	1.85 [0.89, 3.83]	2013
Li, W. 2013	13	30	11	30	2.2%	1.32 [0.47, 3.72]	2013
Zhang, Z.2015	28	45	17	45	2.2%	2.71 [1.16, 6.36]	2015
Li,D.2016	27	40	19	40	2.1%	2.30 [0.93, 5.69]	2016
**Subtotal (95% CI)**		**375**		**372**	**21.0%**	**2.00 [1.48, 2.70]**	
Total events	192		130				
Heterogeneity: Chi² = 2.97, df = 11 (P = 0.99); I² = 0%							
Test for overall effect: Z = 4.55 (P < 0.00001)							
**Staphylococcal enterotoxin C (> 20ml, 200ng, 5000IU)**							
Qiu, Y.1999	12	42	7	42	1.7%	2.00 [0.70, 5.73]	1999
Zhang, G.2000	20	36	6	30	1.0%	5.00 [1.65, 15.17]	2000
Zhu, F.2004	16	34	6	30	1.2%	3.56 [1.16, 10.89]	2004
Zhou, F.2007	5	16	0	12	0.1%	11.96 [0.59, 241.06]	2007
Xing, Q.2012	7	19	3	19	0.7%	3.11 [0.66, 14.60]	2012
Liu, J. 2015	15	30	7	29	1.2%	3.14 [1.03, 9.55]	2015
**Subtotal (95% CI)**		**177**		**162**	**5.9%**	**3.40 [2.05, 5.63]**	
Total events	75		29				
Heterogeneity: Chi² = 2.15, df = 5 (P = 0.83); I² = 0%							
Test for overall effect: Z = 4.76 (P < 0.00001)							
**Staphylococcal enterotoxin C (Unable to group or unclear)**							
Li, H.1998	18	68	6	42	1.9%	2.16 [0.78, 5.98]	1998
Liu, S.2005	13	32	6	29	1.3%	2.62 [0.84, 8.22]	2005
Chen, J. 2014	6	30	2	30	0.6%	3.50 [0.65, 18.98]	2014
**Subtotal (95% CI)**		**130**		**101**	**3.7%**	**2.52 [1.26, 5.04]**	
Total events	37		14				
Heterogeneity: Chi² = 0.24, df = 2 (P = 0.89); I² = 0%							
Test for overall effect: Z = 2.61 (P = 0.009)							
**Total (95% CI)**		**2486**		**2333**	**100.0%**	**2.59 [2.28, 2.95]**	
Total events	1045		531				
Heterogeneity: Chi² = 52.12, df = 76 (P = 0.98); I² = 0%							
Test for overall effect: Z = 14.40 (P < 0.00001)							
Test for subgroup differences: Chi² = 12.57, df = 7 (P = 0.08), I² = 44.3%							

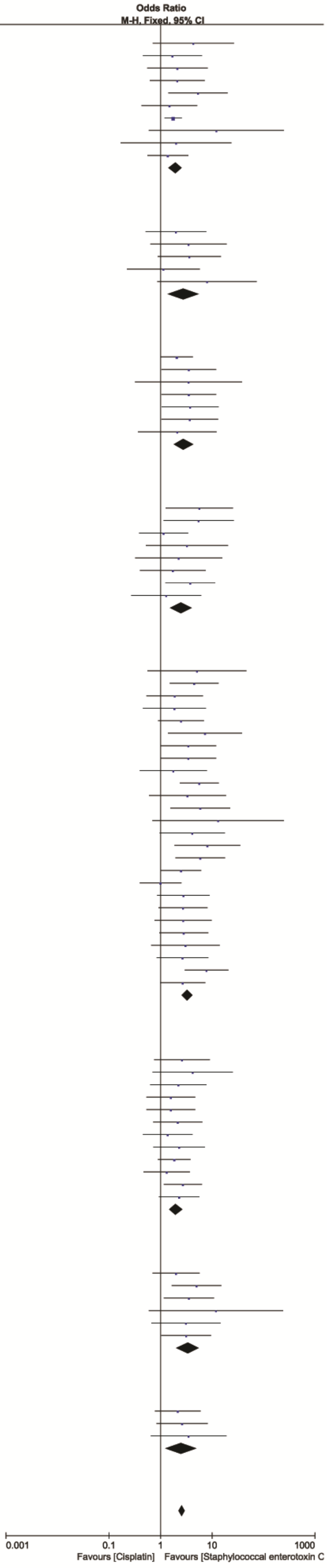

Fig.S38 Subgroups analysis of complete response via staphylococcal enterotoxin C dosage

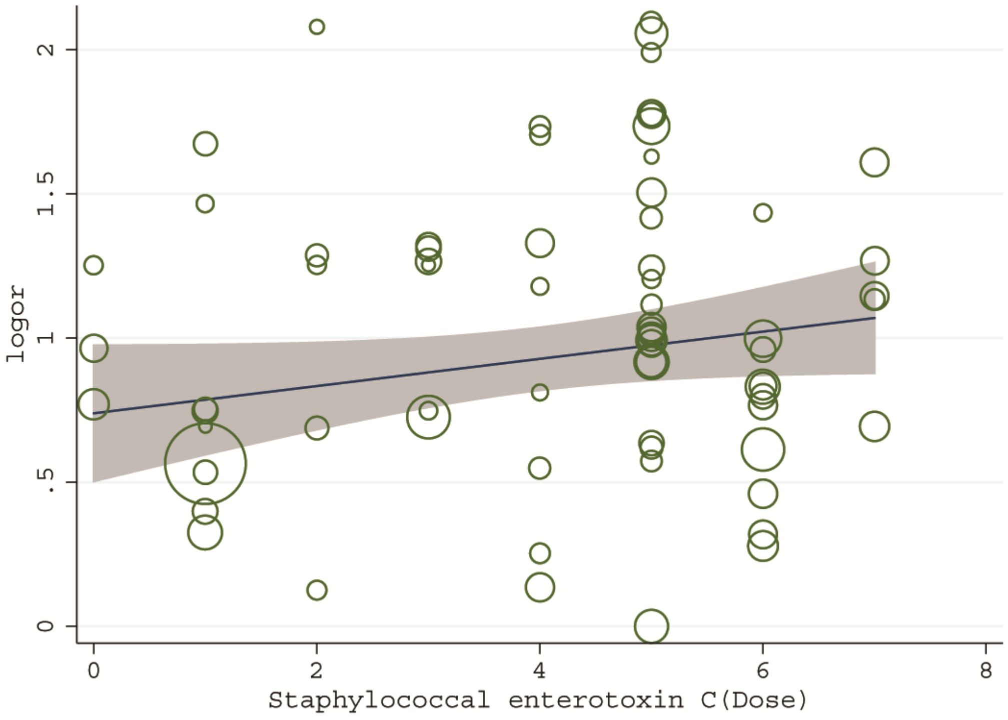

Fig.S39 Meta regression of complete response via staphylococcal enterotoxin C dosage

Study or Subgroup	Staphylococcal enterotoxin C Events	Staphylococcal enterotoxin C Total	Cisplatin Events	Cisplatin Total	Weight	Odds Ratio M-H, Fixed, 95% CI	Year
**Staphylococcal enterotoxin C (8ml,80ng,2000IU)**							
Jia, X.2000	2	15	8	15	0.7%	0.13 [0.02, 0.82]	2000
Wang, C. 2004	5	23	12	22	1.0%	0.23 [0.06, 0.85]	2004
Huang, Y.2005	4	27	9	21	0.9%	0.23 [0.06, 0.91]	2005
Zhang, T. 2005	4	23	10	21	0.9%	0.23 [0.06, 0.92]	2005
Zhao, Z.2006	5	32	14	32	1.3%	0.24 [0.07, 0.78]	2006
Pan, Y.2006	3	28	10	20	1.1%	0.12 [0.03, 0.53]	2006
Li, X.2008	4	25	11	25	1.0%	0.24 [0.06, 0.92]	2008
Li, Z. 2010	50	230	110	230	9.2%	0.30 [0.20, 0.45]	2010
Yu, L. 2013	4	23	11	22	1.0%	0.21 [0.05, 0.82]	2013
Li, L. 2013	3	15	7	15	0.6%	0.29 [0.06, 1.45]	2013
Wang, Y. 2018	4	37	10	37	1.0%	0.33 [0.09, 1.16]	2018
**Subtotal (95% CI)**		**478**		**460**	**18.7%**	**0.26 [0.20, 0.35]**	
Total events	88		212				
Heterogeneity: Chi² = 2.44, df = 10 (P = 0.99); I² = 0%							
Test for overall effect: Z = 8.83 (P < 0.00001)							
**Staphylococcal enterotoxin C (10ml,100ng,2500IU)**							
Wang, L. 2002	5	29	12	29	1.1%	0.30 [0.09, 0.99]	2000
Mao, W. 2004	4	24	7	16	0.7%	0.26 [0.06, 1.11]	2004
Yue, Z.2006	5	33	13	28	1.3%	0.21 [0.06, 0.69]	2006
Zheng, F.2007	6	24	8	20	0.7%	0.50 [0.14, 1.81]	2007
Zhou, Z.2015	4	21	11	21	1.0%	0.21 [0.05, 0.85]	2015
**Subtotal (95% CI)**		**131**		**114**	**4.7%**	**0.28 [0.16, 0.50]**	
Total events	24		51				
Heterogeneity: Chi² = 1.19, df = 4 (P = 0.88); I² = 0%							
Test for overall effect: Z = 4.33 (P < 0.0001)							
**Staphylococcal enterotoxin C (12ml,120ng,3000IU)**							
Duan, R.2003	10	76	37	76	3.4%	0.16 [0.07, 0.36]	2003
Chen, H.2004	3	24	10	24	0.9%	0.20 [0.05, 0.86]	2004
Fang, X.2004	3	15	7	15	0.6%	0.29 [0.06, 1.45]	2004
Ma, D.2005	3	24	10	24	0.9%	0.20 [0.05, 0.86]	2005
Zhang, M.2008	3	33	13	34	1.2%	0.16 [0.04, 0.64]	2008
Zhang, L.2009	3	34	12	35	1.2%	0.19 [0.05, 0.73]	2009
Qu, Y.2010	8	40	19	40	1.6%	0.28 [0.10, 0.75]	2010
**Subtotal (95% CI)**		**246**		**248**	**9.9%**	**0.20 [0.13, 0.31]**	
Total events	33		108				
Heterogeneity: Chi² = 1.00, df = 6 (P = 0.99); I² = 0%							
Test for overall effect: Z = 7.11 (P < 0.00001)							
**Staphylococcal enterotoxin C (16ml,160ng,4000IU)**							
Li, S.1997	3	20	11	20	1.0%	0.14 [0.03, 0.65]	1997
Zhang, Q.1999	1	15	2	15	0.2%	0.46 [0.04, 5.75]	1999
Xu, N.2000	6	34	12	30	1.1%	0.32 [0.10, 1.01]	2000
Wang, C.2003	3	15	7	15	0.6%	0.29 [0.06, 1.45]	2003
Chen, Z.2006	3	12	6	11	0.5%	0.28 [0.05, 1.62]	2006
Yin, W.2007	3	19	10	19	0.9%	0.17 [0.04, 0.78]	2007
Gao, H.2009	7	35	17	30	1.6%	0.19 [0.06, 0.57]	2009
Liu, Q.2010	5	27	9	20	0.9%	0.28 [0.07, 1.03]	2010
**Subtotal (95% CI)**		**177**		**160**	**6.8%**	**0.24 [0.14, 0.39]**	
Total events	31		74				
Heterogeneity: Chi² = 1.44, df = 7 (P = 0.98); I² = 0%							
Test for overall effect: Z = 5.60 (P < 0.00001)							
**Staphylococcal enterotoxin C (20ml,200ng,5000IU)**							
Cao, H.2000	7	26	11	18	1.0%	0.23 [0.06, 0.85]	2000
Lang, F.2001	6	56	12	21	1.7%	0.09 [0.03, 0.30]	2001
Wang, Lei.2002	3	21	8	19	0.8%	0.23 [0.05, 1.05]	2002
Xu, Q.2003	4	32	10	30	1.0%	0.29 [0.08, 1.04]	2003
Zhang, J.2003	4	25	8	28	0.7%	0.48 [0.12, 1.83]	2003
Li, J.2003	4	23	10	21	0.9%	0.23 [0.06, 0.92]	2003
Hu, Y.2004	4	23	10	22	0.9%	0.25 [0.06, 0.99]	2004
Guan, C.2004	4	23	10	22	0.9%	0.25 [0.06, 0.99]	2004
Feng, X.2005	2	17	7	17	0.7%	0.19 [0.03, 1.11]	2005
Liu, M.2005	3	50	21	48	2.2%	0.08 [0.02, 0.30]	2005
Fang, H.2006	4	24	11	22	1.0%	0.20 [0.05, 0.78]	2006
Sun, C.2007	3	24	12	24	1.1%	0.14 [0.03, 0.61]	2007
Gui, Q. 2010	3	25	11	25	1.0%	0.17 [0.04, 0.73]	2010
Cheng, J.2010	6	30	15	30	1.3%	0.25 [0.08, 0.79]	2010
Zhang, C. 2011	1	19	9	19	0.9%	0.06 [0.01, 0.56]	2011
Qin, Q.2012	4	34	23	34	2.2%	0.06 [0.02, 0.23]	2012
Zhao, Y. 2014	2	40	13	40	1.3%	0.11 [0.02, 0.52]	2014
Zhang, J.2014	8	40	25	40	2.1%	0.15 [0.05, 0.41]	2014
Tu, D.2014	4	38	13	38	1.2%	0.23 [0.07, 0.78]	2014
Yao, X.2014	2	25	12	25	1.2%	0.09 [0.02, 0.49]	2014
Cai,C.2015	3	21	7	21	0.6%	0.33 [0.07, 1.53]	2015
Yan, L.2016	4	33	8	28	0.8%	0.34 [0.09, 1.30]	2016
Yu, M.2017	2	20	13	20	1.3%	0.06 [0.01, 0.34]	2017
Luo, D.2018	3	30	14	30	1.3%	0.13 [0.03, 0.51]	2018
Liu, Y. 2019	2	50	23	50	2.4%	0.05 [0.01, 0.22]	2019
Wu, Y. 2019	10	46	27	46	2.3%	0.20 [0.08, 0.49]	2019
**Subtotal (95% CI)**		**795**		**738**	**32.8%**	**0.16 [0.13, 0.21]**	
Total events	102		343				
Heterogeneity: Chi² = 17.25, df = 25 (P = 0.87); I² = 0%							
Test for overall effect: Z = 13.71 (P < 0.00001)							
**Staphylococcal enterotoxin C (10-20ml,100-200ng,2500-5000IU)**							
Chen, J.2005	2	27	7	24	0.7%	0.19 [0.04, 1.05]	2005
Xiong, C.2006	4	22	12	22	1.1%	0.19 [0.05, 0.73]	2006
Zhang, Z.2006	2	16	9	16	0.8%	0.11 [0.02, 0.66]	2006
Huang, W.2008	5	28	12	28	1.1%	0.29 [0.09, 0.98]	2008
Wang, H.2009	5	28	12	28	1.1%	0.29 [0.09, 0.98]	2009
Yu, D.2012	3	25	7	25	0.7%	0.35 [0.08, 1.55]	2012
Xu, J.2012	3	27	12	27	1.1%	0.16 [0.04, 0.65]	2012
Xu, Q.2012	4	27	9	27	0.8%	0.35 [0.09, 1.31]	2012
Li, W. 2013	3	30	10	30	1.0%	0.22 [0.05, 0.91]	2013
Du, Y.2013	6	60	25	60	2.4%	0.16 [0.06, 0.42]	2013
Zhang, Z.2015	2	45	18	45	1.8%	0.07 [0.01, 0.32]	2015
Li,D.2016	1	40	10	40	1.0%	0.08 [0.01, 0.63]	2016
**Subtotal (95% CI)**		**375**		**372**	**13.6%**	**0.19 [0.13, 0.28]**	
Total events	40		143				
Heterogeneity: Chi² = 5.36, df = 11 (P = 0.91); I² = 0%							
Test for overall effect: Z = 8.36 (P < 0.00001)							
**Staphylococcal enterotoxin C (> 20ml, 200ng, 5000IU)**							
Qiu, Y.1999	7	42	17	42	1.5%	0.29 [0.11, 0.81]	1999
Zhang, G.2000	4	36	12	30	1.2%	0.19 [0.05, 0.67]	2000
Zhu, F.2004	6	34	15	30	1.4%	0.21 [0.07, 0.67]	2004
Zhou, F.2007	2	16	7	12	0.7%	0.10 [0.02, 0.66]	2007
Xing, Q.2012	2	19	12	19	1.1%	0.07 [0.01, 0.39]	2012
Liu, J. 2015	4	30	13	29	1.2%	0.19 [0.05, 0.68]	2015
**Subtotal (95% CI)**		**177**		**162**	**7.3%**	**0.19 [0.11, 0.32]**	
Total events	25		76				
Heterogeneity: Chi² = 2.49, df = 5 (P = 0.78); I² = 0%							
Test for overall effect: Z = 6.27 (P < 0.00001)							
**Staphylococcal enterotoxin C (Unable to group or unclear)**							
Li, H.1998	5	68	23	42	2.8%	0.07 [0.02, 0.20]	1998
Liu, S.2005	5	32	14	29	1.3%	0.20 [0.06, 0.66]	2005
Wang, J.2006	4	30	7	30	0.6%	0.51 [0.13, 1.95]	2006
Chen, J. 2014	5	30	15	30	1.3%	0.20 [0.06, 0.66]	2014
**Subtotal (95% CI)**		**160**		**131**	**6.1%**	**0.17 [0.09, 0.31]**	
Total events	19		59				
Heterogeneity: Chi² = 5.55, df = 3 (P = 0.14); I² = 46%							
Test for overall effect: Z = 5.92 (P < 0.00001)							
**Total (95% CI)**		**2539**		**2385**	**100.0%**	**0.20 [0.18, 0.23]**	
Total events	362		1066				
Heterogeneity: Chi² = 43.41, df = 78 (P = 1.00); I² = 0%							
Test for overall effect: Z = 22.57 (P < 0.00001)							
Test for subgroup differences: Chi² = 7.76, df = 7 (P = 0.35), I² = 9.8%							

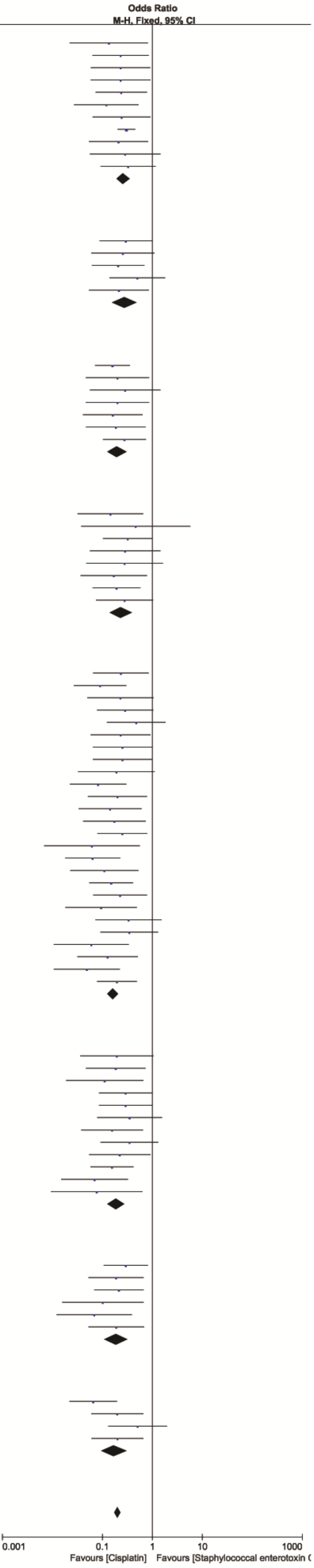

Fig.S40 Subgroups analysis of treatment failure via staphylococcal enterotoxin C dosage

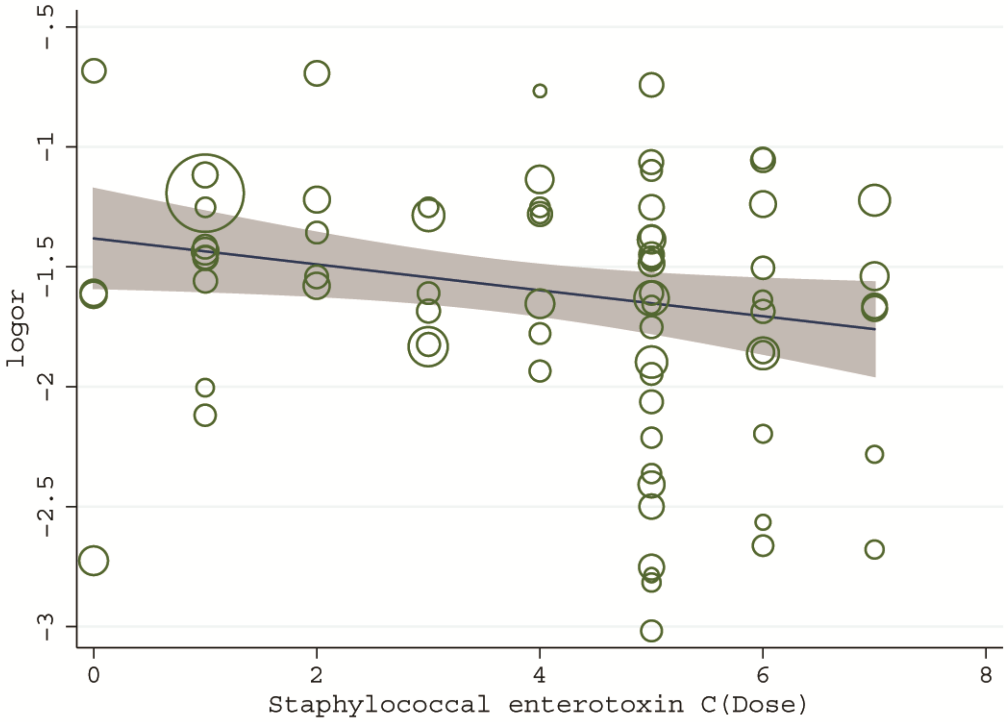

Fig.S41 Meta regression of treatment failure via staphylococcal enterotoxin C dosage

Study or Subgroup	Staphylococcal enterotoxin C Events	Total	Cisplatin Events	Total	Weight	Odds Ratio M-H, Fixed, 95% CI	Year
**One to two times/week**							
Li, S.1997	10	20	3	20	0.5%	5.67 [1.25, 25.61]	1997
Qiu, Y.1999	12	42	7	42	1.7%	2.00 [0.70, 5.73]	1999
Zhang, Q.1999	10	15	4	15	0.5%	5.50 [1.15, 26.41]	1999
Cao, H.2000	6	26	1	18	0.3%	5.10 [0.56, 46.65]	2000
Jia, X.2000	6	15	2	15	0.4%	4.33 [0.71, 26.53]	2000
Xu, N.2000	10	34	8	30	2.1%	1.15 [0.38, 3.43]	2000
Zhang, G.2000	20	36	6	30	1.0%	5.00 [1.65, 15.17]	2000
Wang, L. 2002	7	29	4	29	1.0%	1.99 [0.51, 7.71]	2000
Lang, F.2001	36	56	6	21	1.1%	4.50 [1.51, 13.43]	2001
Wang, Lei.2002	11	21	7	19	1.2%	1.89 [0.53, 6.69]	2002
Duan, R.2003	27	76	16	76	3.6%	2.07 [1.00, 4.26]	2003
Wang, C.2003	5	15	2	15	0.5%	3.25 [0.52, 20.37]	2003
Xu, Q.2003	20	32	12	30	1.6%	2.50 [0.90, 6.95]	2003
Zhang, J.2003	9	25	2	28	0.4%	7.31 [1.40, 38.22]	2003
Li, J.2003	7	23	4	21	1.0%	1.86 [0.46, 7.58]	2003
Chen, H.2004	13	24	6	24	0.9%	3.55 [1.04, 12.06]	2004
Fang, X.2004	3	15	1	15	0.3%	3.50 [0.32, 38.23]	2004
Guan, C.2004	13	23	6	22	0.9%	3.47 [0.99, 12.09]	2004
Hu, Y.2004	13	23	6	22	0.9%	3.47 [0.99, 12.09]	2004
Zhu, F.2004	16	34	6	30	1.2%	3.56 [1.16, 10.89]	2004
Mao, W. 2004	8	24	2	16	0.6%	3.50 [0.63, 19.30]	2004
Liu, S.2005	13	32	6	29	1.3%	2.62 [0.84, 8.22]	2005
Ma, D.2005	13	24	6	24	0.9%	3.55 [1.04, 12.06]	2005
Zhang, T. 2005	8	23	5	21	1.2%	1.71 [0.46, 6.39]	2005
Feng, X.2005	6	17	4	17	0.9%	1.77 [0.40, 7.93]	2005
Chen, J.2005	11	27	5	24	1.1%	2.61 [0.75, 9.11]	2005
Huang, Y.2005	9	27	4	21	1.0%	2.13 [0.55, 8.21]	2005
Liu, M.2005	35	50	14	48	1.5%	5.67 [2.38, 13.50]	2005
Xiong, C.2006	10	22	6	22	1.1%	2.22 [0.63, 7.82]	2006
Yue, Z.2006	10	33	3	28	0.8%	3.62 [0.89, 14.83]	2006
Zhang, Z.2006	6	16	2	16	0.4%	4.20 [0.70, 25.26]	2006
Zhao, Z.2006	9	32	5	32	1.2%	2.11 [0.62, 7.20]	2006
Fang, H.2006	6	24	2	22	0.5%	3.33 [0.60, 18.66]	2006
Pan, Y.2006	16	28	4	20	0.7%	5.33 [1.41, 20.10]	2006
Chen, Z.2006	4	12	2	11	0.5%	2.25 [0.32, 15.76]	2006
Sun, C.2007	13	24	4	24	0.6%	5.91 [1.55, 22.58]	2007
Zhou, F.2007	5	16	0	12	0.1%	11.96 [0.59, 241.06]	2007
Zheng, F.2007	4	24	3	20	0.9%	1.13 [0.22, 5.79]	2007
Zhang, M.2008	11	33	4	34	0.9%	3.75 [1.05, 13.35]	2008
Huang, W.2008	12	28	9	28	1.8%	1.58 [0.53, 4.71]	2008
Li, X.2008	8	25	6	25	1.4%	1.49 [0.43, 5.17]	2008
Wang, H.2009	12	28	9	28	1.8%	1.58 [0.53, 4.71]	2009
Zhang, L.2009	11	34	4	35	0.9%	3.71 [1.05, 13.13]	2009
Qu, Y.2010	4	40	2	40	0.6%	2.11 [0.36, 12.24]	2010
Cheng, J.2010	5	30	0	30	0.1%	13.16 [0.69, 249.48]	2010
Gui, Q. 2010	9	25	3	25	0.7%	4.13 [0.96, 17.70]	2010
Li, Z. 2010	100	230	70	230	13.7%	1.76 [1.20, 2.58]	2010
Liu, Q.2010	5	27	3	20	1.0%	1.29 [0.27, 6.16]	2010
Zhang, C. 2011	13	19	4	19	0.4%	8.13 [1.87, 35.23]	2011
Xing, Q.2012	7	19	3	19	0.7%	3.11 [0.66, 14.60]	2012
Xu, J.2012	14	27	9	27	1.5%	2.15 [0.72, 6.47]	2012
Xu, Q.2012	11	27	9	27	1.8%	1.38 [0.45, 4.17]	2012
Yu, D.2012	17	25	12	25	1.3%	2.30 [0.73, 7.27]	2012
Qin, Q.2012	19	34	6	34	0.9%	5.91 [1.94, 17.97]	2012
Li, W. 2013	13	30	11	30	2.2%	1.32 [0.47, 3.72]	2013
Du, Y.2013	31	60	22	60	3.7%	1.85 [0.89, 3.83]	2013
Li, L. 2013	4	15	0	15	0.1%	12.13 [0.59, 248.49]	2013
Zhao, Y. 2014	25	40	16	40	2.1%	2.50 [1.02, 6.15]	2014
Tu, D.2014	14	38	14	38	3.1%	1.00 [0.39, 2.54]	2014
Yao, X.2014	13	25	7	25	1.2%	2.79 [0.86, 9.01]	2014
Zhang, J.2014	13	40	6	40	1.4%	2.73 [0.92, 8.13]	2014
Zhang, Z.2015	28	45	17	45	2.2%	2.71 [1.16, 6.36]	2015
Cai,C.2015	11	21	6	21	1.0%	2.75 [0.77, 9.86]	2015
Zhou, Z.2015	6	21	1	21	0.2%	8.00 [0.87, 73.68]	2015
Liu, J. 2015	15	30	7	29	1.2%	3.14 [1.03, 9.55]	2015
Yan, L.2016	16	33	7	28	1.3%	2.82 [0.95, 8.44]	2016
Li,D.2016	27	40	19	40	2.1%	2.30 [0.93, 5.69]	2016
Wang, Y. 2018	20	37	17	37	2.7%	1.38 [0.55, 3.45]	2018
Wu, Y. 2019	15	46	7	46	1.6%	2.70 [0.98, 7.43]	2019
**Subtotal (95% CI)**		**2211**		**2090**	**92.2%**	**2.53 [2.21, 2.90]**	
Total events	949		496				
Heterogeneity: Chi² = 45.83, df = 68 (P = 0.98); I² = 0%							
Test for overall effect: Z = 13.39 (P < 0.00001)							
**Others(Unable to group or unclear)**							
Li, H.1998	18	68	6	42	1.9%	2.16 [0.78, 5.98]	1998
Yin, W.2007	6	19	4	19	0.9%	1.73 [0.40, 7.51]	2007
Gao, H.2009	17	35	6	30	1.1%	3.78 [1.24, 11.50]	2009
Yu, L. 2013	2	23	1	22	0.3%	2.00 [0.17, 23.78]	2013
Chen, J. 2014	6	30	2	30	0.6%	3.50 [0.65, 18.98]	2014
Yu, M.2017	7	20	3	20	0.7%	3.05 [0.66, 14.14]	2017
Luo, D.2018	12	30	6	30	1.2%	2.67 [0.84, 8.46]	2018
Liu, Y. 2019	28	50	7	50	1.1%	7.82 [2.95, 20.72]	2019
**Subtotal (95% CI)**		**275**		**243**	**7.8%**	**3.36 [2.16, 5.23]**	
Total events	96		35				
Heterogeneity: Chi² = 4.78, df = 7 (P = 0.69); I² = 0%							
Test for overall effect: Z = 5.36 (P < 0.00001)							
**Total (95% CI)**		**2486**		**2333**	**100.0%**	**2.59 [2.28, 2.95]**	
Total events	1045		531				
Heterogeneity: Chi² = 52.12, df = 76 (P = 0.98); I² = 0%							
Test for overall effect: Z = 14.40 (P < 0.00001)							
Test for subgroup differences: Chi² = 1.44, df = 1 (P = 0.23), I² = 30.4%							

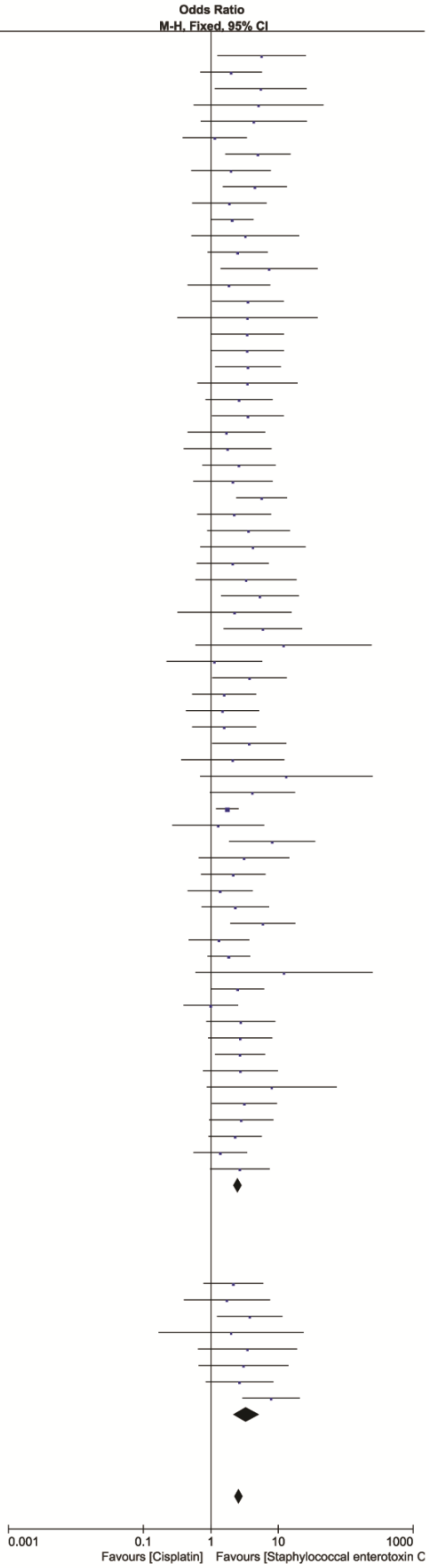

Fig.S42 Subgroups analysis of complete response via treatment frequency

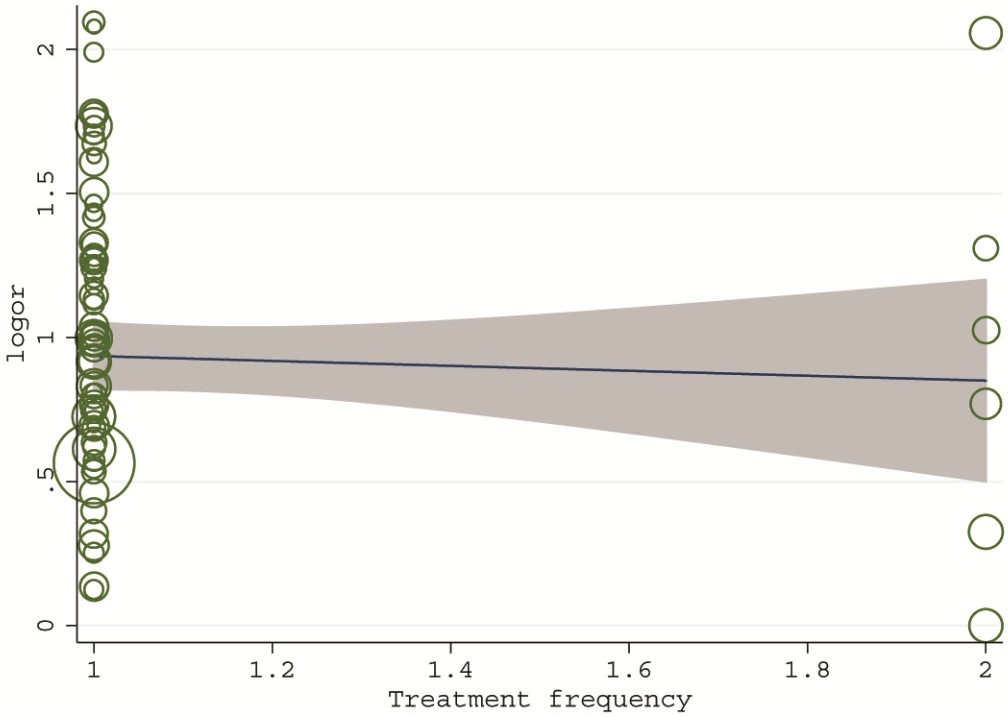

Fig.S43 Meta regression of complete response via treatment frequency

Study or Subgroup	Staphylococcal enterotoxin C Events	Total	Cisplatin Events	Total	Weight	Odds Ratio M-H, Fixed, 95% CI	Year
**One to two times/week**							
Li, S.1997	3	20	11	20	1.0%	0.14 [0.03, 0.65]	1997
Qiu, Y.1999	7	42	17	42	1.5%	0.29 [0.11, 0.81]	1999
Zhang, Q.1999	1	15	2	15	0.2%	0.46 [0.04, 5.75]	1999
Xu, N.2000	6	34	12	30	1.1%	0.32 [0.10, 1.01]	2000
Zhang, G.2000	4	36	12	30	1.2%	0.19 [0.05, 0.67]	2000
Cao, H.2000	7	26	11	18	1.0%	0.23 [0.06, 0.85]	2000
Jia, X.2000	2	15	8	15	0.7%	0.13 [0.02, 0.82]	2000
Wang, L. 2002	5	29	12	29	1.1%	0.30 [0.09, 0.99]	2000
Lang, F.2001	6	56	12	21	1.7%	0.09 [0.03, 0.30]	2001
Wang, Lei.2002	3	21	8	19	0.8%	0.23 [0.05, 1.05]	2002
Xu, Q.2003	4	32	10	30	1.0%	0.29 [0.08, 1.04]	2003
Zhang, J.2003	4	25	8	28	0.7%	0.48 [0.12, 1.83]	2003
Duan, R.2003	10	76	37	76	3.4%	0.16 [0.07, 0.36]	2003
Li, J.2003	4	23	10	21	0.9%	0.23 [0.06, 0.92]	2003
Wang, C.2003	3	15	7	15	0.6%	0.29 [0.06, 1.45]	2003
Hu, Y.2004	4	23	10	22	0.9%	0.25 [0.06, 0.99]	2004
Wang, C. 2004	5	23	12	22	1.0%	0.23 [0.06, 0.85]	2004
Zhu, F.2004	6	34	15	30	1.4%	0.21 [0.07, 0.67]	2004
Chen, H.2004	3	24	10	24	0.9%	0.20 [0.05, 0.86]	2004
Mao, W. 2004	4	24	7	16	0.7%	0.26 [0.06, 1.11]	2004
Fang, X.2004	3	15	7	15	0.6%	0.29 [0.06, 1.45]	2004
Guan, C.2004	4	23	10	22	0.9%	0.25 [0.06, 0.99]	2004
Zhang, T. 2005	4	23	10	21	0.9%	0.23 [0.06, 0.92]	2005
Chen, J.2005	2	27	7	24	0.7%	0.19 [0.04, 1.05]	2005
Feng, X.2005	2	17	7	17	0.7%	0.19 [0.03, 1.11]	2005
Huang, Y.2005	4	27	9	21	0.9%	0.23 [0.06, 0.91]	2005
Liu, S.2005	5	32	14	29	1.3%	0.20 [0.06, 0.66]	2005
Ma, D.2005	3	24	10	24	0.9%	0.20 [0.05, 0.86]	2005
Liu, M.2005	3	50	21	48	2.2%	0.08 [0.02, 0.30]	2005
Xiong, C.2006	4	22	12	22	1.1%	0.19 [0.05, 0.73]	2006
Yue, Z.2006	5	33	13	28	1.3%	0.21 [0.06, 0.69]	2006
Zhang, Z.2006	2	16	9	16	0.8%	0.11 [0.02, 0.66]	2006
Zhao, Z.2006	5	32	14	32	1.3%	0.24 [0.07, 0.78]	2006
Fang, H.2006	4	24	11	22	1.0%	0.20 [0.05, 0.78]	2006
Pan, Y.2006	3	28	10	20	1.1%	0.12 [0.03, 0.53]	2006
Wang, J.2006	4	30	7	30	0.6%	0.51 [0.13, 1.95]	2006
Chen, Z.2006	3	12	6	11	0.5%	0.28 [0.05, 1.62]	2006
Sun, C.2007	3	24	12	24	1.1%	0.14 [0.03, 0.61]	2007
Zhou, F.2007	2	16	7	12	0.7%	0.10 [0.02, 0.66]	2007
Zheng, F.2007	6	24	8	20	0.7%	0.50 [0.14, 1.81]	2007
Huang, W.2008	5	28	12	28	1.1%	0.29 [0.09, 0.98]	2008
Li, X.2008	4	25	11	25	1.0%	0.24 [0.06, 0.92]	2008
Zhang, M.2008	3	33	13	34	1.2%	0.16 [0.04, 0.64]	2008
Wang, H.2009	5	28	12	28	1.1%	0.29 [0.09, 0.98]	2009
Zhang, L.2009	3	34	12	35	1.2%	0.19 [0.05, 0.73]	2009
Liu, Q.2010	5	27	9	20	0.9%	0.28 [0.07, 1.03]	2010
Gui, Q. 2010	3	25	11	25	1.0%	0.17 [0.04, 0.73]	2010
Cheng, J.2010	6	30	15	30	1.3%	0.25 [0.08, 0.79]	2010
Qu, Y.2010	8	40	19	40	1.6%	0.28 [0.10, 0.75]	2010
Li, Z. 2010	50	230	110	230	9.2%	0.30 [0.20, 0.45]	2010
Zhang, C. 2011	1	19	9	19	0.9%	0.06 [0.01, 0.56]	2011
Xu, J.2012	3	27	12	27	1.1%	0.16 [0.04, 0.65]	2012
Xu, Q.2012	4	27	9	27	0.8%	0.35 [0.09, 1.31]	2012
Yu, D.2012	3	25	7	25	0.7%	0.35 [0.08, 1.55]	2012
Qin, Q.2012	4	34	23	34	2.2%	0.06 [0.02, 0.23]	2012
Xing, Q.2012	2	19	12	19	1.1%	0.07 [0.01, 0.39]	2012
Li, L. 2013	3	15	7	15	0.6%	0.29 [0.06, 1.45]	2013
Li, W. 2013	3	30	10	30	1.0%	0.22 [0.05, 0.91]	2013
Du, Y.2013	6	60	25	60	2.4%	0.16 [0.06, 0.42]	2013
Zhao, Y. 2014	2	40	13	40	1.3%	0.11 [0.02, 0.52]	2014
Zhang, J.2014	8	40	25	40	2.1%	0.15 [0.05, 0.41]	2014
Tu, D.2014	4	38	13	38	1.2%	0.23 [0.07, 0.78]	2014
Yao, X.2014	2	25	12	25	1.2%	0.09 [0.02, 0.49]	2014
Liu, J. 2015	4	30	13	29	1.2%	0.19 [0.05, 0.68]	2015
Zhou, Z.2015	4	21	11	21	1.0%	0.21 [0.05, 0.85]	2015
Cai,C.2015	3	21	7	21	0.6%	0.33 [0.07, 1.53]	2015
Zhang, Z.2015	2	45	18	45	1.8%	0.07 [0.01, 0.32]	2015
Li,D.2016	1	40	10	40	1.0%	0.08 [0.01, 0.63]	2016
Yan, L.2016	4	33	8	28	0.8%	0.34 [0.09, 1.30]	2016
Wang, Y. 2018	4	37	10	37	1.0%	0.33 [0.09, 1.16]	2018
Wu, Y. 2019	10	46	27	46	2.3%	0.20 [0.08, 0.49]	2019
**Subtotal (95% CI)**		**2264**		**2142**	**87.4%**	**0.21 [0.18, 0.25]**	
Total events	331		940				
Heterogeneity: Chi² = 32.68, df = 70 (P = 1.00); I² = 0%							
Test for overall effect: Z = 20.67 (P < 0.00001)							
**Others(Unable to group or unclear）**							
Li, H.1998	5	68	23	42	2.8%	0.07 [0.02, 0.20]	1998
Yin, W.2007	3	19	10	19	0.9%	0.17 [0.04, 0.78]	2007
Gao, H.2009	7	35	17	30	1.6%	0.19 [0.06, 0.57]	2009
Yu, L. 2013	4	23	11	22	1.0%	0.21 [0.05, 0.82]	2013
Chen, J. 2014	5	30	15	30	1.3%	0.20 [0.06, 0.66]	2014
Yu, M.2017	2	20	13	20	1.3%	0.06 [0.01, 0.34]	2017
Luo, D.2018	3	30	14	30	1.3%	0.13 [0.03, 0.51]	2018
Liu, Y. 2019	2	50	23	50	2.4%	0.05 [0.01, 0.22]	2019
**Subtotal (95% CI)**		**275**		**243**	**12.6%**	**0.12 [0.07, 0.19]**	
Total events	31		126				
Heterogeneity: Chi² = 5.40, df = 7 (P = 0.61); I² = 0%							
Test for overall effect: Z = 9.19 (P < 0.00001)							
**Total (95% CI)**		**2539**		**2385**	**100.0%**	**0.20 [0.18, 0.23]**	
Total events	362		1066				
Heterogeneity: Chi² = 43.41, df = 78 (P = 1.00); I² = 0%							
Test for overall effect: Z = 22.57 (P < 0.00001)							
Test for subgroup differences: Chi² = 5.97, df = 1 (P = 0.01), I² = 83.2%							

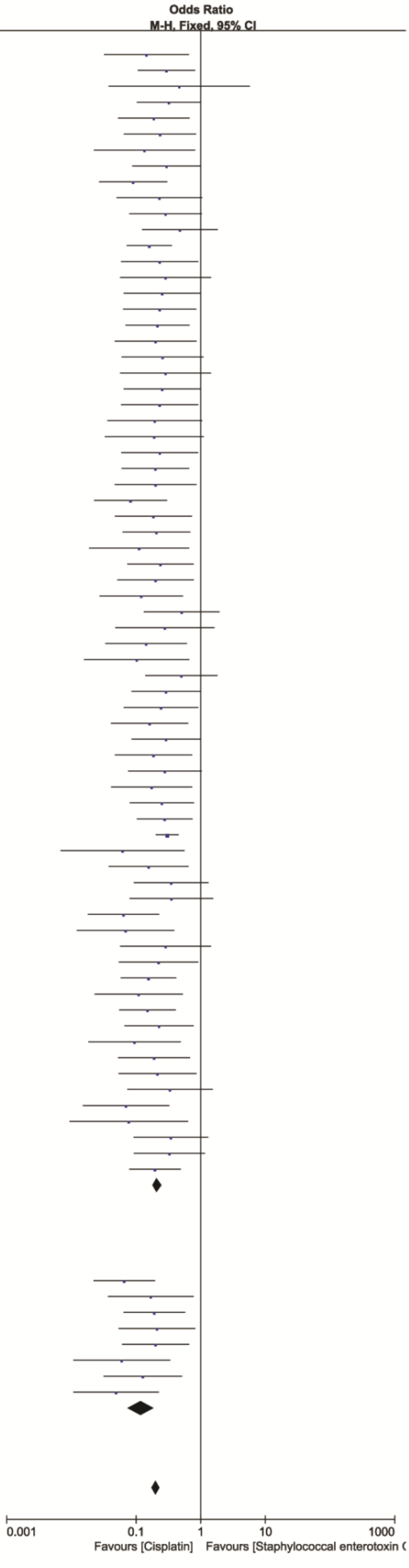

Fig.S44 Subgroups analysis of treatment failure via treatment frequency

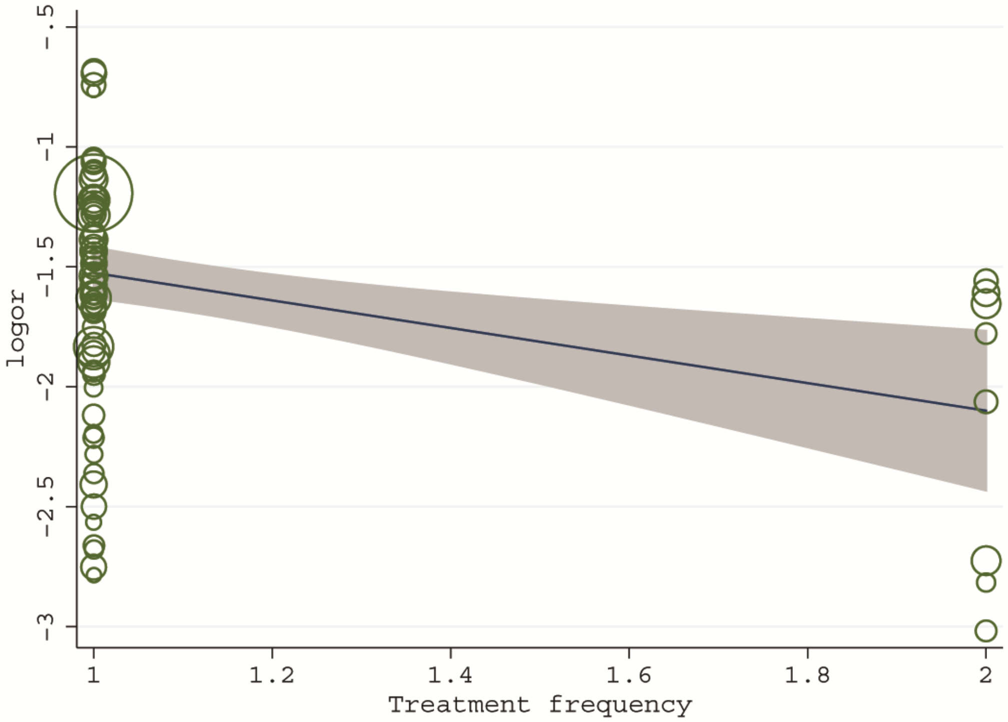

Fig.S45 Meta regression of treatment failure via treatment frequency

Study or Subgroup	Staphylococcal enterotoxin C Events	Staphylococcal enterotoxin C Total	Cisplatin Events	Cisplatin Total	Weight	Odds Ratio M-H, Fixed, 95% CI	Year
**One to four times**							
Qiu, Y.1999	12	42	7	42	1.7%	2.00 [0.70, 5.73]	1999
Zhang, Q.1999	10	15	4	15	0.5%	5.50 [1.15, 26.41]	1999
Cao, H.2000	6	26	1	18	0.3%	5.10 [0.56, 46.65]	2000
Jia, X.2000	6	15	2	15	0.4%	4.33 [0.71, 26.53]	2000
Xu, N.2000	10	34	8	30	2.1%	1.15 [0.38, 3.43]	2000
Wang, L. 2002	7	29	4	29	1.0%	1.99 [0.51, 7.71]	2000
Lang, F.2001	36	56	6	21	1.1%	4.50 [1.51, 13.43]	2001
Wang, Lei.2002	11	21	7	19	1.2%	1.89 [0.53, 6.69]	2002
Duan, R.2003	27	76	16	76	3.6%	2.07 [1.00, 4.26]	2003
Wang, C.2003	5	15	2	15	0.5%	3.25 [0.52, 20.37]	2003
Xu, Q.2003	20	32	12	30	1.6%	2.50 [0.90, 6.95]	2003
Zhang, J.2003	9	25	2	28	0.4%	7.31 [1.40, 38.22]	2003
Li, J.2003	7	23	4	21	1.0%	1.86 [0.46, 7.58]	2003
Chen, H.2004	13	24	6	24	0.9%	3.55 [1.04, 12.06]	2004
Fang, X.2004	3	15	1	15	0.3%	3.50 [0.32, 38.23]	2004
Guan, C.2004	13	23	6	22	0.9%	3.47 [0.99, 12.09]	2004
Hu, Y.2004	13	23	6	22	0.9%	3.47 [0.99, 12.09]	2004
Zhu, F.2004	16	34	6	30	1.2%	3.56 [1.16, 10.89]	2004
Mao, W. 2004	8	24	2	16	0.6%	3.50 [0.63, 19.30]	2004
Ma, D.2005	13	24	6	24	0.9%	3.55 [1.04, 12.06]	2005
Zhang, T. 2005	8	23	5	21	1.2%	1.71 [0.46, 6.39]	2005
Feng, X.2005	6	17	4	17	0.9%	1.77 [0.40, 7.93]	2005
Huang, Y.2005	9	27	4	21	1.0%	2.13 [0.55, 8.21]	2005
Liu, M.2005	35	50	14	48	1.5%	5.67 [2.38, 13.50]	2005
Xiong, C.2006	10	22	6	22	1.1%	2.22 [0.63, 7.82]	2006
Yue, Z.2006	10	33	3	28	0.8%	3.62 [0.89, 14.83]	2006
Fang, H.2006	6	24	2	22	0.5%	3.33 [0.60, 18.66]	2006
Pan, Y.2006	16	28	4	20	0.7%	5.33 [1.41, 20.10]	2006
Chen, Z.2006	4	12	2	11	0.5%	2.25 [0.32, 15.76]	2006
Sun, C.2007	13	24	4	24	0.6%	5.91 [1.55, 22.58]	2007
Zheng, F.2007	4	24	3	20	0.9%	1.13 [0.22, 5.79]	2007
Zhang, M.2008	11	33	4	34	0.9%	3.75 [1.05, 13.35]	2008
Huang, W.2008	12	28	9	28	1.8%	1.58 [0.53, 4.71]	2008
Li, X.2008	8	25	6	25	1.4%	1.49 [0.43, 5.17]	2008
Wang, H.2009	12	28	9	28	1.8%	1.58 [0.53, 4.71]	2009
Zhang, L.2009	11	34	4	35	0.9%	3.71 [1.05, 13.13]	2009
Qu, Y.2010	4	40	2	40	0.6%	2.11 [0.36, 12.24]	2010
Cheng, J.2010	5	30	0	30	0.1%	13.16 [0.69, 249.48]	2010
Gui, Q. 2010	9	25	3	25	0.7%	4.13 [0.96, 17.70]	2010
Li, Z. 2010	100	230	70	230	13.7%	1.76 [1.20, 2.58]	2010
Liu, Q.2010	5	27	3	20	1.0%	1.29 [0.27, 6.16]	2010
Xing, Q.2012	7	19	3	19	0.7%	3.11 [0.66, 14.60]	2012
Xu, J.2012	14	27	9	27	1.5%	2.15 [0.72, 6.47]	2012
Xu, Q.2012	11	27	9	27	1.8%	1.38 [0.45, 4.17]	2012
Yu, D.2012	17	25	12	25	1.3%	2.30 [0.73, 7.27]	2012
Li, W. 2013	13	30	11	30	2.2%	1.32 [0.47, 3.72]	2013
Du, Y.2013	31	60	22	60	3.7%	1.85 [0.89, 3.83]	2013
Li, L. 2013	4	15	0	15	0.1%	12.13 [0.59, 248.49]	2013
Zhao, Y. 2014	25	40	16	40	2.1%	2.50 [1.02, 6.15]	2014
Tu, D.2014	14	38	14	38	3.1%	1.00 [0.39, 2.54]	2014
Yao, X.2014	13	25	7	25	1.2%	2.79 [0.86, 9.01]	2014
Zhang, Z.2015	28	45	17	45	2.2%	2.71 [1.16, 6.36]	2015
Cai,C.2015	11	21	6	21	1.0%	2.75 [0.77, 9.86]	2015
Zhou, Z.2015	6	21	1	21	0.2%	8.00 [0.87, 73.68]	2015
Liu, J. 2015	15	30	7	29	1.2%	3.14 [1.03, 9.55]	2015
Li,D.2016	27	40	19	40	2.1%	2.30 [0.93, 5.69]	2016
Yu, M.2017	7	20	3	20	0.7%	3.05 [0.66, 14.14]	2017
Luo, D.2018	12	30	6	30	1.2%	2.67 [0.84, 8.46]	2018
Liu, Y. 2019	28	50	7	50	1.1%	7.82 [2.95, 20.72]	2019
**Subtotal (95% CI)**		**1923**		**1823**	**81.0%**	**2.49 [2.16, 2.88]**	
Total events	826		438				
Heterogeneity: Chi² = 40.54, df = 58 (P = 0.96); I² = 0%							
Test for overall effect: Z = 12.32 (P < 0.00001)							
**>4 times**							
Li, S.1997	10	20	3	20	0.5%	5.67 [1.25, 25.61]	1997
Li, H.1998	18	68	6	42	1.9%	2.16 [0.78, 5.98]	1998
Zhang, Z.2006	6	16	2	16	0.4%	4.20 [0.70, 25.26]	2006
Zhou, F.2007	5	16	0	12	0.1%	11.96 [0.59, 241.06]	2007
Zhang, J.2014	13	40	6	40	1.4%	2.73 [0.92, 8.13]	2014
Yan, L.2016	16	33	7	28	1.3%	2.82 [0.95, 8.44]	2016
Wu, Y. 2019	15	46	7	46	1.6%	2.70 [0.98, 7.43]	2019
**Subtotal (95% CI)**		**239**		**204**	**7.3%**	**3.05 [1.91, 4.88]**	
Total events	83		31				
Heterogeneity: Chi² = 2.12, df = 6 (P = 0.91); I² = 0%							
Test for overall effect: Z = 4.66 (P < 0.00001)							
**Others (Unable to group or unclear)**							
Zhang, G.2000	20	36	6	30	1.0%	5.00 [1.65, 15.17]	2000
Chen, J.2005	11	27	5	24	1.1%	2.61 [0.75, 9.11]	2005
Liu, S.2005	13	32	6	29	1.3%	2.62 [0.84, 8.22]	2005
Zhao, Z.2006	9	32	5	32	1.2%	2.11 [0.62, 7.20]	2006
Yin, W.2007	6	19	4	19	0.9%	1.73 [0.40, 7.51]	2007
Gao, H.2009	17	35	6	30	1.1%	3.78 [1.24, 11.50]	2009
Zhang, C. 2011	13	19	4	19	0.4%	8.13 [1.87, 35.23]	2011
Qin, Q.2012	19	34	6	34	0.9%	5.91 [1.94, 17.97]	2012
Yu, L. 2013	2	23	1	22	0.3%	2.00 [0.17, 23.78]	2013
Chen, J. 2014	6	30	2	30	0.6%	3.50 [0.65, 18.98]	2014
Wang, Y. 2018	20	37	17	37	2.7%	1.38 [0.55, 3.45]	2018
**Subtotal (95% CI)**		**324**		**306**	**11.6%**	**3.02 [2.09, 4.36]**	
Total events	136		62				
Heterogeneity: Chi² = 8.02, df = 10 (P = 0.63); I² = 0%							
Test for overall effect: Z = 5.88 (P < 0.00001)							
**Total (95% CI)**		**2486**		**2333**	**100.0%**	**2.59 [2.28, 2.95]**	
Total events	1045		531				
Heterogeneity: Chi² = 52.12, df = 76 (P = 0.98); I² = 0%							
Test for overall effect: Z = 14.40 (P < 0.00001)							
Test for subgroup differences: Chi² = 1.40, df = 2 (P = 0.50), I² = 0%							

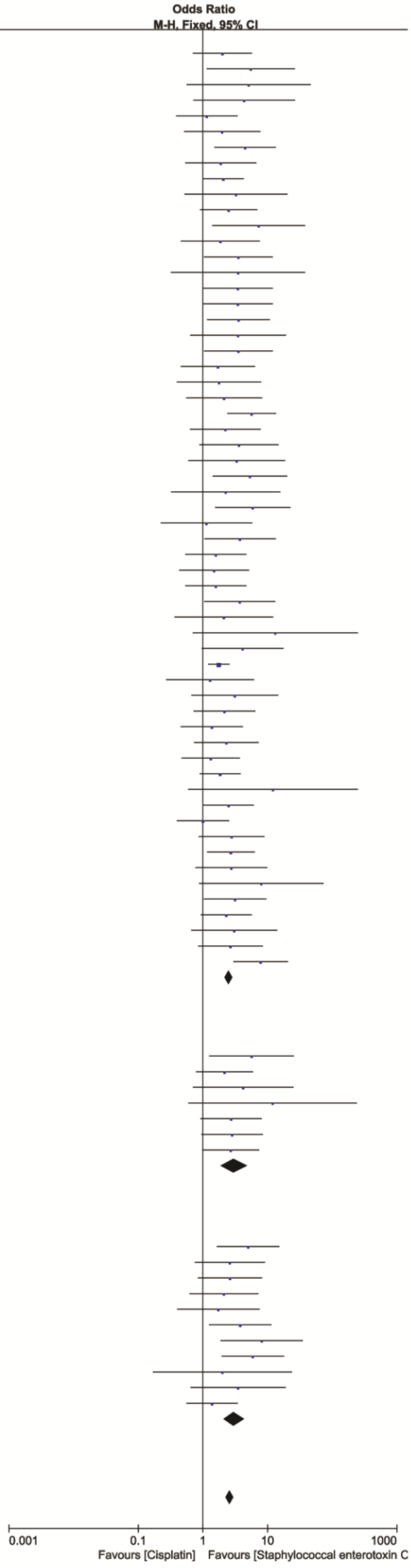

Fig.S46 Subgroups analysis of complete response via treatment times

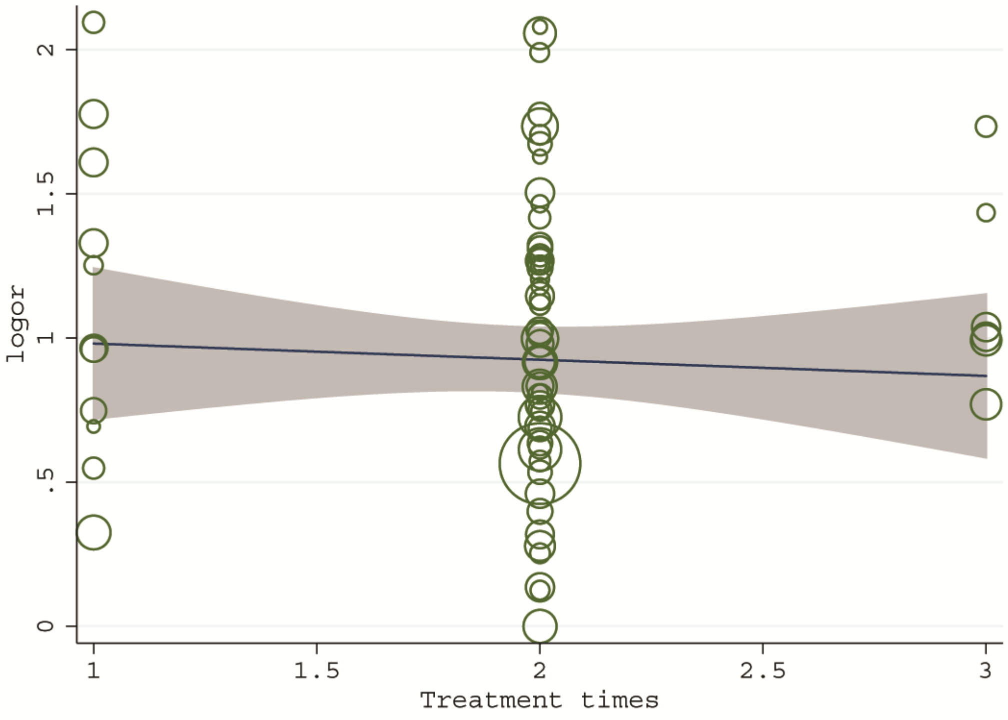

Fig.S47 Meta regression of complete response via treatment times

Study or Subgroup	Staphylococcal enterotoxin C Events	Staphylococcal enterotoxin C Total	Cisplatin Events	Cisplatin Total	Weight	Odds Ratio M-H, Fixed, 95% CI	Year
**One to four times**							
Qiu, Y.1999	7	42	17	42	1.5%	0.29 [0.11, 0.81]	1999
Zhang, Q.1999	1	15	2	15	0.2%	0.46 [0.04, 5.75]	1999
Xu, N.2000	6	34	12	30	1.1%	0.32 [0.10, 1.01]	2000
Cao, H.2000	7	26	11	18	1.0%	0.23 [0.06, 0.85]	2000
Jia, X.2000	2	15	8	15	0.7%	0.13 [0.02, 0.82]	2000
Wang, L. 2002	5	29	12	29	1.1%	0.30 [0.09, 0.99]	2000
Lang, F.2001	6	56	12	21	1.7%	0.09 [0.03, 0.30]	2001
Wang, Lei.2002	3	21	8	19	0.8%	0.23 [0.05, 1.05]	2002
Xu, Q.2003	4	32	10	30	1.0%	0.29 [0.08, 1.04]	2003
Zhang, J.2003	4	25	8	28	0.7%	0.48 [0.12, 1.83]	2003
Duan, R.2003	10	76	37	76	3.4%	0.16 [0.07, 0.36]	2003
Li, J.2003	4	23	10	21	0.9%	0.23 [0.06, 0.92]	2003
Wang, C.2003	3	15	7	15	0.6%	0.29 [0.06, 1.45]	2003
Hu, Y.2004	4	23	10	22	0.9%	0.25 [0.06, 0.99]	2004
Wang, C. 2004	5	23	12	22	1.0%	0.23 [0.06, 0.85]	2004
Zhu, F.2004	6	34	15	30	1.4%	0.21 [0.07, 0.67]	2004
Chen, H.2004	3	24	10	24	0.9%	0.20 [0.05, 0.86]	2004
Mao, W. 2004	4	24	7	16	0.7%	0.26 [0.06, 1.11]	2004
Fang, X.2004	3	15	7	15	0.6%	0.29 [0.06, 1.45]	2004
Guan, C.2004	4	23	10	22	0.9%	0.25 [0.06, 0.99]	2004
Zhang, T. 2005	4	23	10	21	0.9%	0.23 [0.06, 0.92]	2005
Feng, X.2005	2	17	7	17	0.7%	0.19 [0.03, 1.11]	2005
Huang, Y.2005	4	27	9	21	0.9%	0.23 [0.06, 0.91]	2005
Ma, D.2005	3	24	10	24	0.9%	0.20 [0.05, 0.86]	2005
Liu, M.2005	3	50	21	48	2.2%	0.08 [0.02, 0.30]	2005
Xiong, C.2006	4	22	12	22	1.1%	0.19 [0.05, 0.73]	2006
Yue, Z.2006	5	33	13	28	1.3%	0.21 [0.06, 0.69]	2006
Fang, H.2006	4	24	11	22	1.0%	0.20 [0.05, 0.78]	2006
Pan, Y.2006	3	28	10	20	1.1%	0.12 [0.03, 0.53]	2006
Wang, J.2006	4	30	7	30	0.6%	0.51 [0.13, 1.95]	2006
Chen, Z.2006	3	12	6	11	0.5%	0.28 [0.05, 1.62]	2006
Sun, C.2007	3	24	12	24	1.1%	0.14 [0.03, 0.61]	2007
Zheng, F.2007	6	24	8	20	0.7%	0.50 [0.14, 1.81]	2007
Huang, W.2008	5	28	12	28	1.1%	0.29 [0.09, 0.98]	2008
Li, X.2008	4	25	11	25	1.0%	0.24 [0.06, 0.92]	2008
Zhang, M.2008	3	33	13	34	1.2%	0.16 [0.04, 0.64]	2008
Wang, H.2009	5	28	12	28	1.1%	0.29 [0.09, 0.98]	2009
Zhang, L.2009	3	34	12	35	1.2%	0.19 [0.05, 0.73]	2009
Liu, Q.2010	5	27	9	20	0.9%	0.28 [0.07, 1.03]	2010
Gui, Q. 2010	3	25	11	25	1.0%	0.17 [0.04, 0.73]	2010
Cheng, J.2010	6	30	15	30	1.3%	0.25 [0.08, 0.79]	2010
Qu, Y.2010	8	40	19	40	1.6%	0.28 [0.10, 0.75]	2010
Li, Z. 2010	50	230	110	230	9.2%	0.30 [0.20, 0.45]	2010
Xing, Q.2012	2	19	12	19	1.1%	0.07 [0.01, 0.39]	2012
Xu, J.2012	3	27	12	27	1.1%	0.16 [0.04, 0.65]	2012
Xu, Q.2012	4	27	9	27	0.8%	0.35 [0.09, 1.31]	2012
Yu, D.2012	3	25	7	25	0.7%	0.35 [0.08, 1.55]	2012
Du, Y.2013	6	60	25	60	2.4%	0.16 [0.06, 0.42]	2013
Li, L. 2013	3	15	7	15	0.6%	0.29 [0.06, 1.45]	2013
Li, W. 2013	3	30	10	30	1.0%	0.22 [0.05, 0.91]	2013
Zhao, Y. 2014	2	40	13	40	1.3%	0.11 [0.02, 0.52]	2014
Tu, D.2014	4	38	13	38	1.2%	0.23 [0.07, 0.78]	2014
Yao, X.2014	2	25	12	25	1.2%	0.09 [0.02, 0.49]	2014
Liu, J. 2015	4	30	13	29	1.2%	0.19 [0.05, 0.68]	2015
Zhou, Z.2015	4	21	11	21	1.0%	0.21 [0.05, 0.85]	2015
Cai,C.2015	3	21	7	21	0.6%	0.33 [0.07, 1.53]	2015
Zhang, Z.2015	2	45	18	45	1.8%	0.07 [0.01, 0.32]	2015
Li,D.2016	1	40	10	40	1.0%	0.08 [0.01, 0.63]	2016
Yu, M.2017	2	20	13	20	1.3%	0.06 [0.01, 0.34]	2017
Luo, D.2018	3	30	14	30	1.3%	0.13 [0.03, 0.51]	2018
Liu, Y. 2019	2	50	23	50	2.4%	0.05 [0.01, 0.22]	2019
**Subtotal (95% CI)**		**1976**		**1875**	**76.0%**	**0.21 [0.18, 0.25]**	
Total events	284		814				
Heterogeneity: Chi² = 31.29, df = 60 (P = 1.00); I² = 0%							
Test for overall effect: Z = 19.23 (P < 0.00001)							
**>4 times**							
Li, S.1997	3	20	11	20	1.0%	0.14 [0.03, 0.65]	1997
Li, H.1998	5	68	23	42	2.8%	0.07 [0.02, 0.20]	1998
Zhang, Z.2006	2	16	9	16	0.8%	0.11 [0.02, 0.66]	2006
Zhou, F.2007	2	16	7	12	0.7%	0.10 [0.02, 0.66]	2007
Zhang, J.2014	8	40	25	40	2.1%	0.15 [0.05, 0.41]	2014
Yan, L.2016	4	33	8	28	0.8%	0.34 [0.09, 1.30]	2016
Wu, Y. 2019	10	46	27	46	2.3%	0.20 [0.08, 0.49]	2019
**Subtotal (95% CI)**		**239**		**204**	**10.6%**	**0.15 [0.09, 0.23]**	
Total events	34		110				
Heterogeneity: Chi² = 4.29, df = 6 (P = 0.64); I² = 0%							
Test for overall effect: Z = 8.23 (P < 0.00001)							
**Others (Unable to group or unclear）**							
Zhang, G.2000	4	36	12	30	1.2%	0.19 [0.05, 0.67]	2000
Chen, J.2005	2	27	7	24	0.7%	0.19 [0.04, 1.05]	2005
Liu, S.2005	5	32	14	29	1.3%	0.20 [0.06, 0.66]	2005
Zhao, Z.2006	5	32	14	32	1.3%	0.24 [0.07, 0.78]	2006
Yin, W.2007	3	19	10	19	0.9%	0.17 [0.04, 0.78]	2007
Gao, H.2009	7	35	17	30	1.6%	0.19 [0.06, 0.57]	2009
Zhang, C. 2011	1	19	9	19	0.9%	0.06 [0.01, 0.56]	2011
Qin, Q.2012	4	34	23	34	2.2%	0.06 [0.02, 0.23]	2012
Yu, L. 2013	4	23	11	22	1.0%	0.21 [0.05, 0.82]	2013
Chen, J. 2014	5	30	15	30	1.3%	0.20 [0.06, 0.66]	2014
Wang, Y. 2018	4	37	10	37	1.0%	0.33 [0.09, 1.16]	2018
**Subtotal (95% CI)**		**324**		**306**	**13.4%**	**0.18 [0.12, 0.26]**	
Total events	44		142				
Heterogeneity: Chi² = 4.70, df = 10 (P = 0.91); I² = 0%							
Test for overall effect: Z = 8.62 (P < 0.00001)							
**Total (95% CI)**		**2539**		**2385**	**100.0%**	**0.20 [0.18, 0.23]**	
Total events	362		1066				
Heterogeneity: Chi² = 43.41, df = 78 (P = 1.00); I² = 0%							
Test for overall effect: Z = 22.57 (P < 0.00001)							
Test for subgroup differences: Chi² = 2.86, df = 2 (P = 0.24), I² = 30.0%							

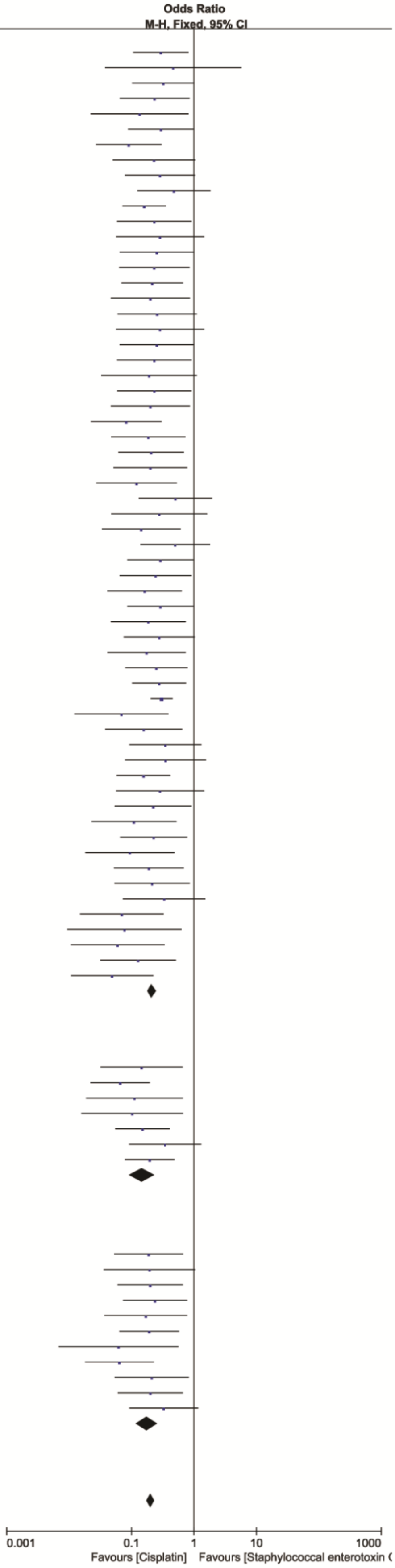

Fig.S48 Subgroups analysis of treatment failure via treatment times

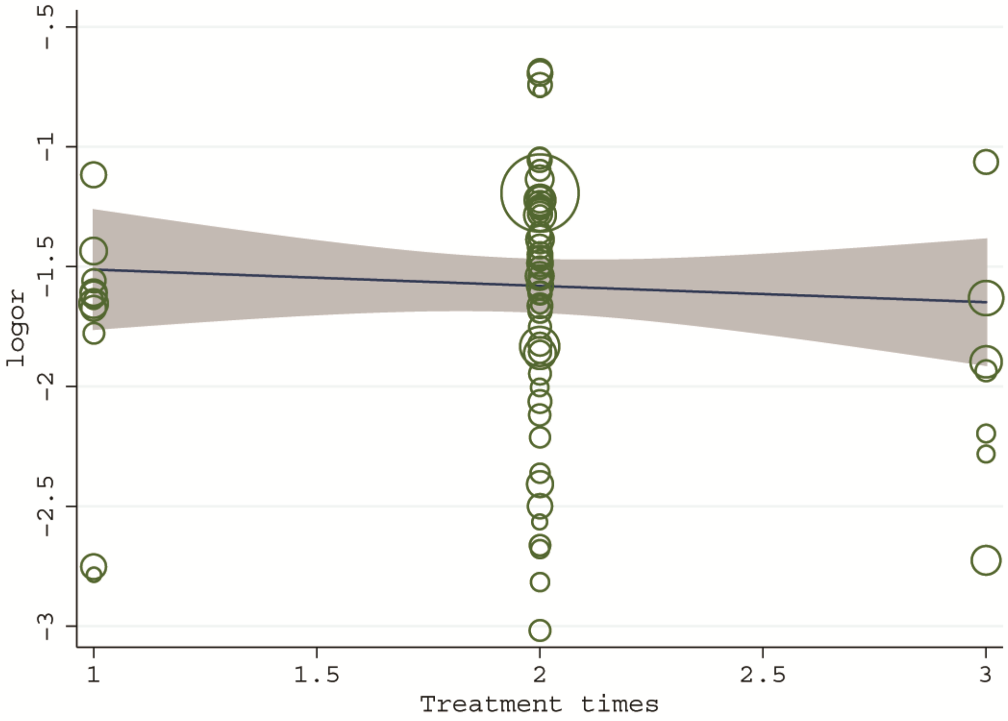

Fig.S49 Meta regression of treatment failure via treatment times

	Staphylococcal enterotoxin C		Cisplatin			Odds Ratio	
Study or Subgroup	Events	Total	Events	Total	Weight	M-H, Fixed, 95% CI	Year
**Cisplatin (30-40 mg each time)**							
Zhu, F.2004	16	34	6	30	1.2%	3.56 [1.16, 10.89]	2004
Chen, J.2005	11	27	5	24	1.1%	2.61 [0.75, 9.11]	2005
Sun, C.2007	13	24	4	24	0.6%	5.91 [1.55, 22.58]	2007
Li, X.2008	8	25	6	25	1.4%	1.49 [0.43, 5.17]	2008
Cheng, J.2010	5	30	0	30	0.1%	13.16 [0.69, 249.48]	2010
Qin, Q.2012	19	34	6	34	0.9%	5.91 [1.94, 17.97]	2012
Yu, L. 2013	2	23	1	22	0.3%	2.00 [0.17, 23.78]	2013
Zhou, Z.2015	6	21	1	21	0.2%	8.00 [0.87, 73.68]	2015
Liu, J. 2015	15	30	7	29	1.2%	3.14 [1.03, 9.55]	2015
**Subtotal (95% CI)**		**248**		**239**	**7.1%**	**3.72 [2.37, 5.83]**	
Total events	95		36				
Heterogeneity: Chi² = 5.01, df = 8 (P = 0.76); I² = 0%							
Test for overall effect: Z = 5.72 (P < 0.00001)							
**Cisplatin (50-60 mg each time)**							
Xu, N.2000	10	34	8	30	2.1%	1.15 [0.38, 3.43]	2000
Zhang, G.2000	20	36	6	30	1.0%	5.00 [1.65, 15.17]	2000
Wang, L. 2002	7	29	4	29	1.0%	1.99 [0.51, 7.71]	2000
Wang, C.2003	5	15	2	15	0.5%	3.25 [0.52, 20.37]	2003
Zhang, J.2003	9	25	2	28	0.4%	7.31 [1.40, 38.22]	2003
Chen, H.2004	13	24	6	24	0.9%	3.55 [1.04, 12.06]	2004
Hu, Y.2004	13	23	6	22	0.9%	3.47 [0.99, 12.09]	2004
Mao, W. 2004	8	24	2	16	0.6%	3.50 [0.63, 19.30]	2004
Ma, D.2005	13	24	6	24	0.9%	3.55 [1.04, 12.06]	2005
Zhang, T. 2005	8	23	5	21	1.2%	1.71 [0.46, 6.39]	2005
Feng, X.2005	6	17	4	17	0.9%	1.77 [0.40, 7.93]	2005
Xiong, C.2006	10	22	6	22	1.1%	2.22 [0.63, 7.82]	2006
Yue, Z.2006	10	33	3	28	0.8%	3.62 [0.89, 14.83]	2006
Zhao, Z.2006	9	32	5	32	1.2%	2.11 [0.62, 7.20]	2006
Fang, H.2006	6	24	2	22	0.5%	3.33 [0.60, 18.66]	2006
Zheng, F.2007	4	24	3	20	0.9%	1.13 [0.22, 5.79]	2007
Gao, H.2009	17	35	6	30	1.1%	3.78 [1.24, 11.50]	2009
Qu, Y.2010	4	40	2	40	0.6%	2.11 [0.36, 12.24]	2010
Gui, Q. 2010	9	25	3	25	0.7%	4.13 [0.96, 17.70]	2010
Li, Z. 2010	100	230	70	230	13.7%	1.76 [1.20, 2.58]	2010
Liu, Q.2010	5	27	3	20	1.0%	1.29 [0.27, 6.16]	2010
Xing, Q.2012	7	19	3	19	0.7%	3.11 [0.66, 14.60]	2012
Li, L. 2013	4	15	0	15	0.1%	12.13 [0.59, 248.49]	2013
Zhao, Y. 2014	25	40	16	40	2.1%	2.50 [1.02, 6.15]	2014
Tu, D.2014	14	38	14	38	3.1%	1.00 [0.39, 2.54]	2014
Yao, X.2014	13	25	7	25	1.2%	2.79 [0.86, 9.01]	2014
Zhang, J.2014	13	40	6	40	1.4%	2.73 [0.92, 8.13]	2014
Cai,C.2015	11	21	6	21	1.0%	2.75 [0.77, 9.86]	2015
Yan, L.2016	16	33	7	28	1.3%	2.82 [0.95, 8.44]	2016
Yu, M.2017	7	20	3	20	0.7%	3.05 [0.66, 14.14]	2017
Luo, D.2018	12	30	6	30	1.2%	2.67 [0.84, 8.46]	2018
Liu, Y. 2019	28	50	7	50	1.1%	7.82 [2.95, 20.72]	2019
Wu, Y. 2019	15	46	7	46	1.6%	2.70 [0.98, 7.43]	2019
**Subtotal (95% CI)**		**1143**		**1097**	**47.5%**	**2.47 [2.04, 2.99]**	
Total events	451		236				
Heterogeneity: Chi² = 23.41, df = 32 (P = 0.86); I² = 0%							
Test for overall effect: Z = 9.30 (P < 0.00001)							
**Cisplatin (70-100 mg each time)**							
Qiu, Y.1999	12	42	7	42	1.7%	2.00 [0.70, 5.73]	1999
Cao, H.2000	6	26	1	18	0.3%	5.10 [0.56, 46.65]	2000
Wang, Lei.2002	11	21	7	19	1.2%	1.89 [0.53, 6.69]	2002
Duan, R.2003	27	76	16	76	3.6%	2.07 [1.00, 4.26]	2003
Xu, Q.2003	20	32	12	30	1.6%	2.50 [0.90, 6.95]	2003
Guan, C.2004	13	23	6	22	0.9%	3.47 [0.99, 12.09]	2004
Fang, X.2004	3	15	1	15	0.3%	3.50 [0.32, 38.23]	2004
Chen, Z.2006	4	12	2	11	0.5%	2.25 [0.32, 15.76]	2006
Pan, Y.2006	16	28	4	20	0.7%	5.33 [1.41, 20.10]	2006
Zhou, F.2007	5	16	0	12	0.1%	11.96 [0.59, 241.06]	2007
Huang, W.2008	12	28	9	28	1.8%	1.58 [0.53, 4.71]	2008
Wang, H.2009	12	28	9	28	1.8%	1.58 [0.53, 4.71]	2009
Zhang, C. 2011	13	19	4	19	0.4%	8.13 [1.87, 35.23]	2011
Xu, J.2012	14	27	9	27	1.5%	2.15 [0.72, 6.47]	2012
Xu, Q.2012	11	27	9	27	1.8%	1.38 [0.45, 4.17]	2012
Du, Y.2013	31	60	22	60	3.7%	1.85 [0.89, 3.83]	2013
Li, W. 2013	13	30	11	30	2.2%	1.32 [0.47, 3.72]	2013
Zhang, Z.2015	28	45	17	45	2.2%	2.71 [1.16, 6.36]	2015
Li,D.2016	27	40	19	40	2.1%	2.30 [0.93, 5.69]	2016
Wang, Y. 2018	20	37	17	37	2.7%	1.38 [0.55, 3.45]	2018
**Subtotal (95% CI)**		**632**		**606**	**31.1%**	**2.20 [1.73, 2.80]**	
Total events	298		182				
Heterogeneity: Chi² = 11.13, df = 19 (P = 0.92); I² = 0%							
Test for overall effect: Z = 6.40 (P < 0.00001)							
**Cisplatin (others)**							
Li, S.1997	10	20	3	20	0.5%	5.67 [1.25, 25.61]	1997
Li, H.1998	18	68	6	42	1.9%	2.16 [0.78, 5.98]	1998
Zhang, Q.1999	10	15	4	15	0.5%	5.50 [1.15, 26.41]	1999
Jia, X.2000	6	15	2	15	0.4%	4.33 [0.71, 26.53]	2000
Lang, F.2001	36	56	6	21	1.1%	4.50 [1.51, 13.43]	2001
Li, J.2003	7	23	4	21	1.0%	1.86 [0.46, 7.58]	2003
Huang, Y.2005	9	27	4	21	1.0%	2.13 [0.55, 8.21]	2005
Liu, S.2005	13	32	6	29	1.3%	2.62 [0.84, 8.22]	2005
Liu, M.2005	35	50	14	48	1.5%	5.67 [2.38, 13.50]	2005
Zhang, Z.2006	6	16	2	16	0.4%	4.20 [0.70, 25.26]	2006
Yin, W.2007	6	19	4	19	0.9%	1.73 [0.40, 7.51]	2007
Zhang, M.2008	11	33	4	34	0.9%	3.75 [1.05, 13.35]	2008
Zhang, L.2009	11	34	4	35	0.9%	3.71 [1.05, 13.13]	2009
Yu, D.2012	17	25	12	25	1.3%	2.30 [0.73, 7.27]	2012
Chen, J. 2014	6	30	2	30	0.6%	3.50 [0.65, 18.98]	2014
**Subtotal (95% CI)**		**463**		**391**	**14.2%**	**3.32 [2.39, 4.60]**	
Total events	201		77				
Heterogeneity: Chi² = 5.92, df = 14 (P = 0.97); I² = 0%							
Test for overall effect: Z = 7.18 (P < 0.00001)							
**Total (95% CI)**		**2486**		**2333**	**100.0%**	**2.59 [2.28, 2.95]**	
Total events	1045		531				
Heterogeneity: Chi² = 52.12, df = 76 (P = 0.98); I² = 0%							
Test for overall effect: Z = 14.40 (P < 0.00001)							
Test for subgroup differences: Chi² = 6.70, df = 3 (P = 0.08), I² = 55.2%							

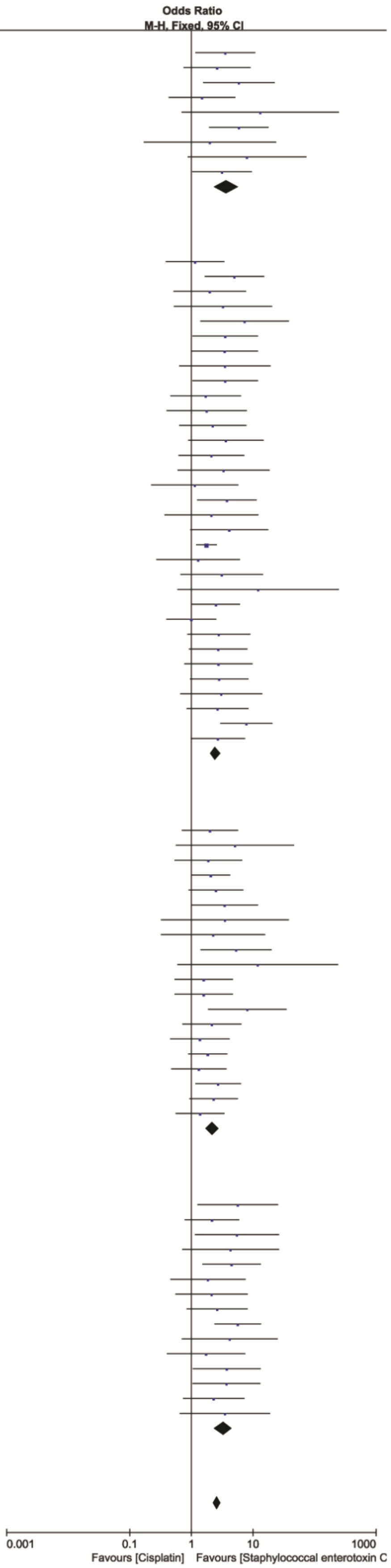

Fig.S50 Subgroups analysis of complete response via Cisplatin (dosage)

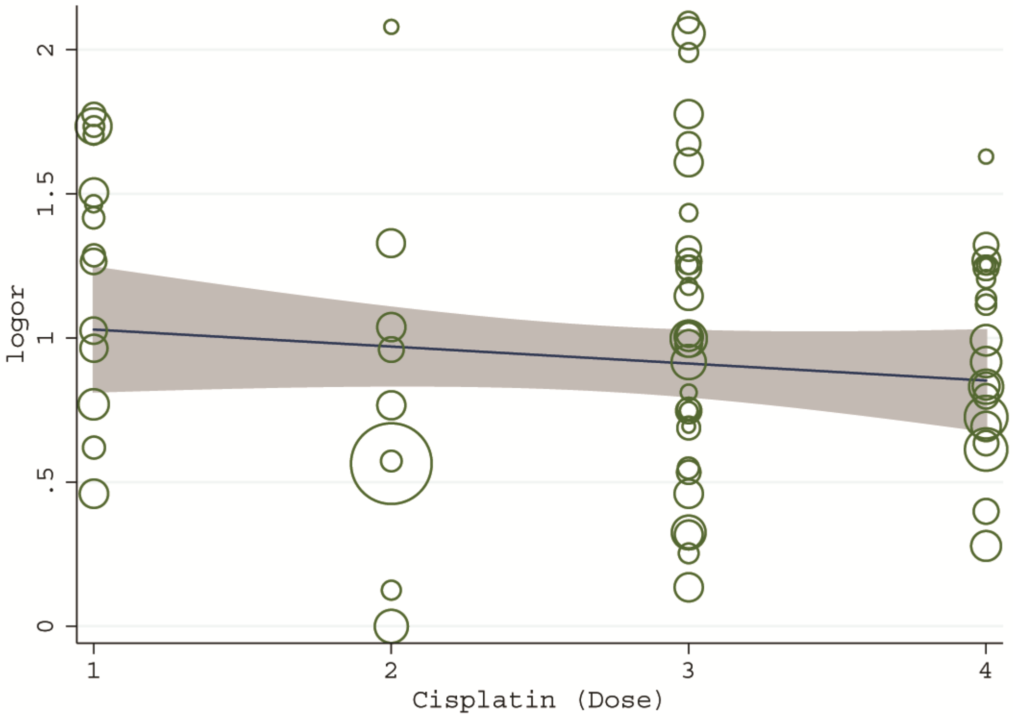

Fig.S51 Meta regression of complete response via Cisplatin (dosage)

Study or Subgroup	Staphylococcal enterotoxin C Events	Staphylococcal enterotoxin C Total	Cisplatin Events	Cisplatin Total	Weight	Odds Ratio M-H, Fixed, 95% CI	Year
**Cisplatin (30-40 mg each time)**							
Zhu, F.2004	6	34	15	30	1.4%	0.21 [0.07, 0.67]	2004
Chen, J.2005	2	27	7	24	0.7%	0.19 [0.04, 1.05]	2005
Sun, C.2007	3	24	12	24	1.1%	0.14 [0.03, 0.61]	2007
Li, X.2008	4	25	11	25	1.0%	0.24 [0.06, 0.92]	2008
Cheng, J.2010	6	30	15	30	1.3%	0.25 [0.08, 0.79]	2010
Qin, Q.2012	4	34	23	34	2.2%	0.06 [0.02, 0.23]	2012
Yu, L. 2013	4	23	11	22	1.0%	0.21 [0.05, 0.82]	2013
Zhou, Z.2015	4	21	11	21	1.0%	0.21 [0.05, 0.85]	2015
Liu, J. 2015	4	30	13	29	1.2%	0.19 [0.05, 0.68]	2015
**Subtotal (95% CI)**		**248**		**239**	**10.9%**	**0.18 [0.12, 0.28]**	
Total events	37		118				
Heterogeneity: Chi² = 3.40, df = 8 (P = 0.91); I² = 0%							
Test for overall effect: Z = 7.78 (P < 0.00001)							
**Cisplatin (50-60 mg each time)**							
Xu, N.2000	6	34	12	30	1.1%	0.32 [0.10, 1.01]	2000
Zhang, G.2000	4	36	12	30	1.2%	0.19 [0.05, 0.67]	2000
Wang, L. 2002	5	29	12	29	1.1%	0.30 [0.09, 0.99]	2000
Zhang, J.2003	4	25	8	28	0.7%	0.48 [0.12, 1.83]	2003
Wang, C.2003	3	15	7	15	0.6%	0.29 [0.06, 1.45]	2003
Hu, Y.2004	4	23	10	22	0.9%	0.25 [0.06, 0.99]	2004
Chen, H.2004	3	24	10	24	0.9%	0.20 [0.05, 0.86]	2004
Mao, W. 2004	4	24	7	16	0.7%	0.26 [0.06, 1.11]	2004
Zhang, T. 2005	4	23	10	21	0.9%	0.23 [0.06, 0.92]	2005
Feng, X.2005	2	17	7	17	0.7%	0.19 [0.03, 1.11]	2005
Ma, D.2005	3	24	10	24	0.9%	0.20 [0.05, 0.86]	2005
Xiong, C.2006	4	22	12	22	1.1%	0.19 [0.05, 0.73]	2006
Yue, Z.2006	5	33	13	28	1.3%	0.21 [0.06, 0.69]	2006
Zhao, Z.2006	5	32	14	32	1.3%	0.24 [0.07, 0.78]	2006
Fang, H.2006	4	24	11	22	1.0%	0.20 [0.05, 0.78]	2006
Zheng, F.2007	6	24	8	20	0.7%	0.50 [0.14, 1.81]	2007
Gao, H.2009	7	35	17	30	1.6%	0.19 [0.06, 0.57]	2009
Liu, Q.2010	5	27	9	20	0.9%	0.28 [0.07, 1.03]	2010
Gui, Q. 2010	3	25	11	25	1.0%	0.17 [0.04, 0.73]	2010
Qu, Y.2010	8	40	19	40	1.6%	0.28 [0.10, 0.75]	2010
Li, Z. 2010	50	230	110	230	9.2%	0.30 [0.20, 0.45]	2010
Xing, Q.2012	2	19	12	19	1.1%	0.07 [0.01, 0.39]	2012
Li, L. 2013	3	15	7	15	0.6%	0.29 [0.06, 1.45]	2013
Zhao, Y. 2014	2	40	13	40	1.3%	0.11 [0.02, 0.52]	2014
Zhang, J.2014	8	40	25	40	2.1%	0.15 [0.05, 0.41]	2014
Tu, D.2014	4	38	13	38	1.2%	0.23 [0.07, 0.78]	2014
Yao, X.2014	2	25	12	25	1.2%	0.09 [0.02, 0.49]	2014
Cai,C.2015	3	21	7	21	0.6%	0.33 [0.07, 1.53]	2015
Yan, L.2016	4	33	8	28	0.8%	0.34 [0.09, 1.30]	2016
Yu, M.2017	2	20	13	20	1.3%	0.06 [0.01, 0.34]	2017
Luo, D.2018	3	30	14	30	1.3%	0.13 [0.03, 0.51]	2018
Liu, Y. 2019	2	50	23	50	2.4%	0.05 [0.01, 0.22]	2019
Wu, Y. 2019	10	46	27	46	2.3%	0.20 [0.08, 0.49]	2019
**Subtotal (95% CI)**		**1143**		**1097**	**45.8%**	**0.22 [0.18, 0.27]**	
Total events	184		503				
Heterogeneity: Chi² = 18.17, df = 32 (P = 0.98); I² = 0%							
Test for overall effect: Z = 14.74 (P < 0.00001)							
**Cisplatin (70-100 mg each time)**							
Qiu, Y.1999	7	42	17	42	1.5%	0.29 [0.11, 0.81]	1999
Cao, H.2000	7	26	11	18	1.0%	0.23 [0.06, 0.85]	2000
Wang, Lei.2002	3	21	8	19	0.8%	0.23 [0.05, 1.05]	2002
Duan, R.2003	10	76	37	76	3.4%	0.16 [0.07, 0.36]	2003
Xu, Q.2003	4	32	10	30	1.0%	0.29 [0.08, 1.04]	2003
Fang, X.2004	3	15	7	15	0.6%	0.29 [0.06, 1.45]	2004
Guan, C.2004	4	23	10	22	0.9%	0.25 [0.06, 0.99]	2004
Wang, C. 2004	5	23	12	22	1.0%	0.23 [0.06, 0.85]	2004
Chen, Z.2006	3	12	6	11	0.5%	0.28 [0.05, 1.62]	2006
Pan, Y.2006	3	28	10	20	1.1%	0.12 [0.03, 0.53]	2006
Zhou, F.2007	2	16	7	12	0.7%	0.10 [0.02, 0.66]	2007
Huang, W.2008	5	28	12	28	1.1%	0.29 [0.09, 0.98]	2008
Wang, H.2009	5	28	12	28	1.1%	0.29 [0.09, 0.98]	2009
Zhang, C. 2011	1	19	9	19	0.9%	0.06 [0.01, 0.56]	2011
Xu, J.2012	3	27	12	27	1.1%	0.16 [0.04, 0.65]	2012
Xu, Q.2012	4	27	9	27	0.8%	0.35 [0.09, 1.31]	2012
Du, Y.2013	6	60	25	60	2.4%	0.16 [0.06, 0.42]	2013
Li, W. 2013	3	30	10	30	1.0%	0.22 [0.05, 0.91]	2013
Zhang, Z.2015	2	45	18	45	1.8%	0.07 [0.01, 0.32]	2015
Li,D.2016	1	40	10	40	1.0%	0.08 [0.01, 0.63]	2016
Wang, Y. 2018	4	37	10	37	1.0%	0.33 [0.09, 1.16]	2018
**Subtotal (95% CI)**		**655**		**628**	**24.8%**	**0.20 [0.15, 0.26]**	
Total events	85		262				
Heterogeneity: Chi² = 8.71, df = 20 (P = 0.99); I² = 0%							
Test for overall effect: Z = 11.20 (P < 0.00001)							
**Cisplatin (others)**							
Li, S.1997	3	20	11	20	1.0%	0.14 [0.03, 0.65]	1997
Li, H.1998	5	68	23	42	2.8%	0.07 [0.02, 0.20]	1998
Zhang, Q.1999	1	15	2	15	0.2%	0.46 [0.04, 5.75]	1999
Jia, X.2000	2	15	8	15	0.7%	0.13 [0.02, 0.82]	2000
Lang, F.2001	6	56	12	21	1.7%	0.09 [0.03, 0.30]	2001
Li, J.2003	4	23	10	21	0.9%	0.23 [0.06, 0.92]	2003
Huang, Y.2005	4	27	9	21	0.9%	0.23 [0.06, 0.91]	2005
Liu, S.2005	5	32	14	29	1.3%	0.20 [0.06, 0.66]	2005
Liu, M.2005	3	50	21	48	2.2%	0.08 [0.02, 0.30]	2005
Wang, J.2006	4	30	7	30	0.6%	0.51 [0.13, 1.95]	2006
Zhang, Z.2006	2	16	9	16	0.8%	0.11 [0.02, 0.66]	2006
Yin, W.2007	3	19	10	19	0.9%	0.17 [0.04, 0.78]	2007
Zhang, M.2008	3	33	13	34	1.2%	0.16 [0.04, 0.64]	2008
Zhang, L.2009	3	34	12	35	1.2%	0.19 [0.05, 0.73]	2009
Yu, D.2012	3	25	7	25	0.7%	0.35 [0.08, 1.55]	2012
Chen, J. 2014	5	30	15	30	1.3%	0.20 [0.06, 0.66]	2014
**Subtotal (95% CI)**		**493**		**421**	**18.5%**	**0.16 [0.12, 0.23]**	
Total events	56		183				
Heterogeneity: Chi² = 10.04, df = 15 (P = 0.82); I² = 0%							
Test for overall effect: Z = 10.35 (P < 0.00001)							
**Total (95% CI)**		**2539**		**2385**	**100.0%**	**0.20 [0.18, 0.23]**	
Total events	362		1066				
Heterogeneity: Chi² = 43.41, df = 78 (P = 1.00); I² = 0%							
Test for overall effect: Z = 22.57 (P < 0.00001)							
Test for subgroup differences: Chi² = 2.77, df = 3 (P = 0.43), I² = 0%							

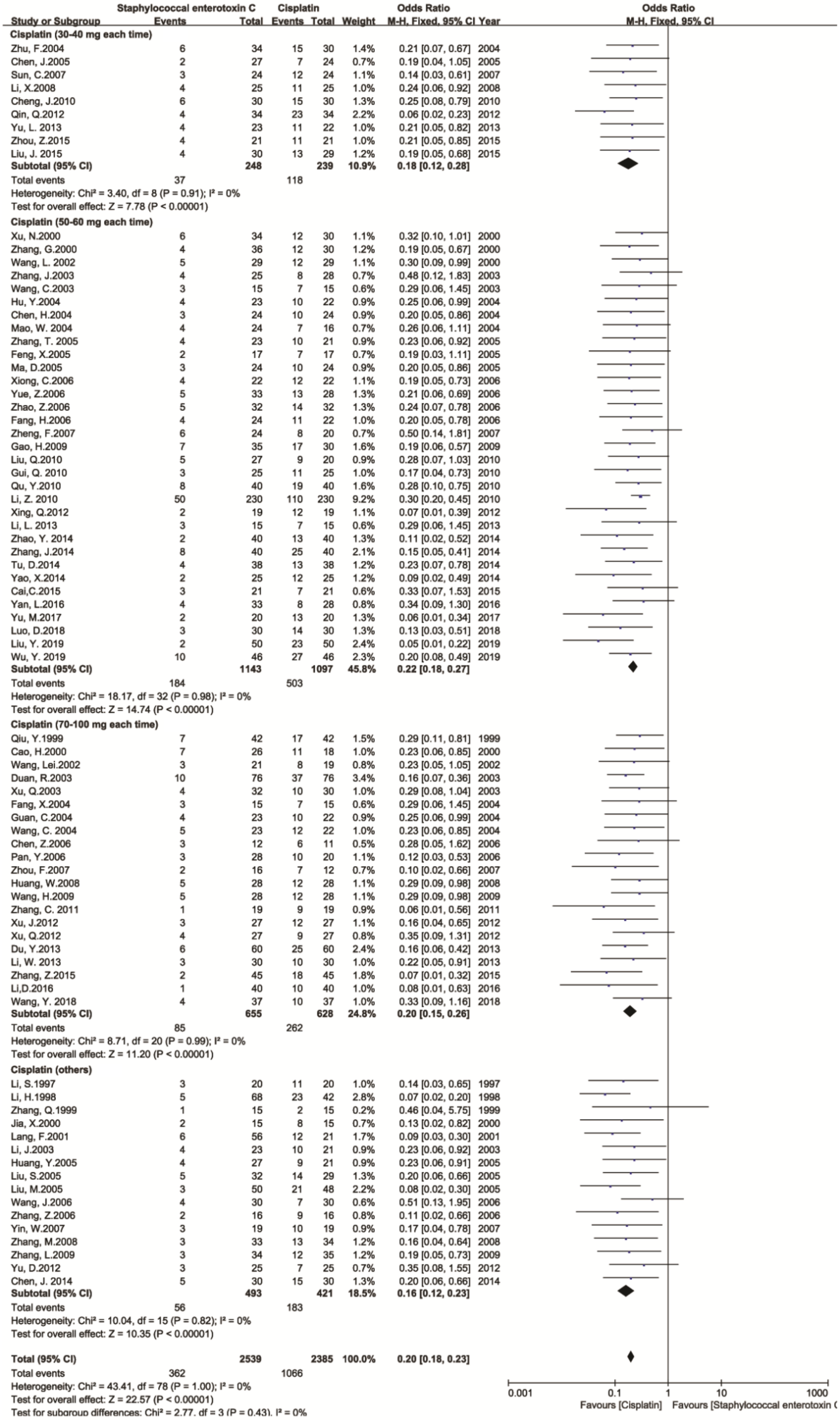

Fig.S52 Subgroups analysis of treatment failure via Cisplatin (dosage)

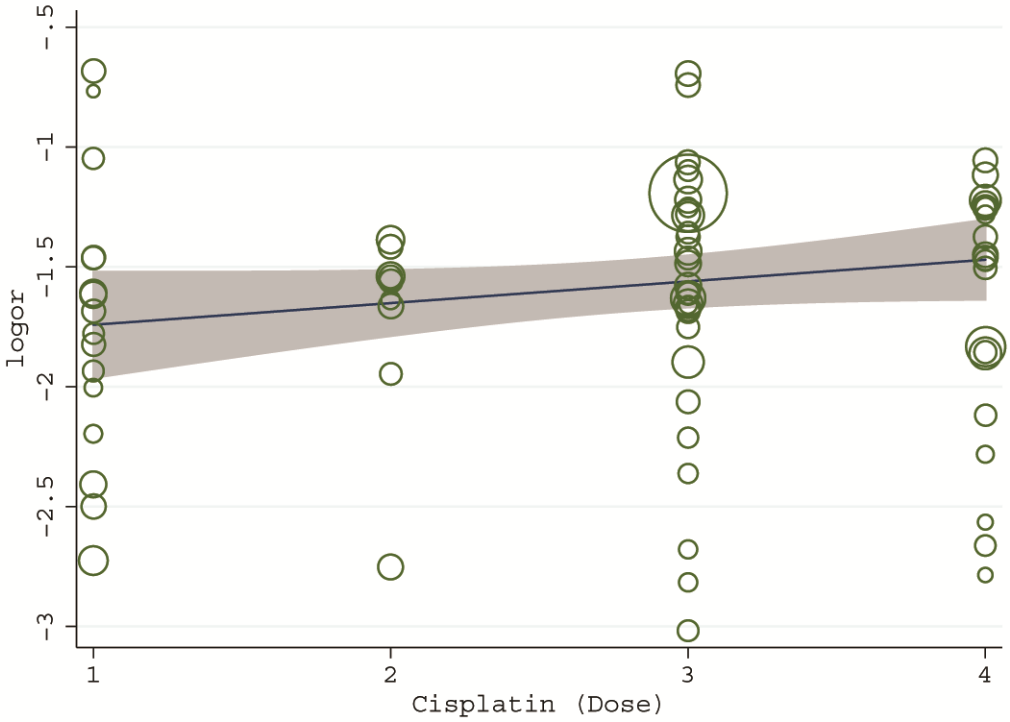

Fig.S53 Meta regression of treatment failure via Cisplatin (dosage)

	Staphylococcal enterotoxin C		Cisplatin			Odds Ratio	
Study or Subgroup	Events	Total	Events	Total	Weight	M-H, Fixed, 95% CI	Year
**Equivalent dosage**							
Li, S.1997	10	20	3	20	0.5%	5.67 [1.25, 25.61]	1997
Li, H.1998	18	68	6	42	1.9%	2.16 [0.78, 5.98]	1998
Qiu, Y.1999	12	42	7	42	1.7%	2.00 [0.70, 5.73]	1999
Zhang, Q.1999	10	15	4	15	0.5%	5.50 [1.15, 26.41]	1999
Cao, H.2000	6	26	1	18	0.3%	5.10 [0.56, 46.65]	2000
Jia, X.2000	6	15	2	15	0.4%	4.33 [0.71, 26.53]	2000
Xu, N.2000	10	34	8	30	2.1%	1.15 [0.38, 3.43]	2000
Zhang, G.2000	20	36	6	30	1.0%	5.00 [1.65, 15.17]	2000
Wang, L. 2002	7	29	4	29	1.0%	1.99 [0.51, 7.71]	2000
Lang, F.2001	36	56	6	21	1.1%	4.50 [1.51, 13.43]	2001
Wang, Lei.2002	11	21	7	19	1.2%	1.89 [0.53, 6.69]	2002
Duan, R.2003	27	76	16	76	3.6%	2.07 [1.00, 4.26]	2003
Wang, C.2003	5	15	2	15	0.5%	3.25 [0.52, 20.37]	2003
Li, J.2003	7	23	4	21	1.0%	1.86 [0.46, 7.58]	2003
Chen, H.2004	13	24	6	24	0.9%	3.55 [1.04, 12.06]	2004
Guan, C.2004	13	23	6	22	0.9%	3.47 [0.99, 12.09]	2004
Hu, Y.2004	13	23	6	22	0.9%	3.47 [0.99, 12.09]	2004
Zhu, F.2004	16	34	6	30	1.2%	3.56 [1.16, 10.89]	2004
Mao, W. 2004	8	24	2	16	0.6%	3.50 [0.63, 19.30]	2004
Liu, S.2005	13	32	6	29	1.3%	2.62 [0.84, 8.22]	2005
Ma, D.2005	13	24	6	24	0.9%	3.55 [1.04, 12.06]	2005
Zhang, T. 2005	8	23	5	21	1.2%	1.71 [0.46, 6.39]	2005
Feng, X.2005	6	17	4	17	0.9%	1.77 [0.40, 7.93]	2005
Chen, J.2005	11	27	5	24	1.1%	2.61 [0.75, 9.11]	2005
Liu, M.2005	35	50	14	48	1.5%	5.67 [2.38, 13.50]	2005
Xiong, C.2006	10	22	6	22	1.1%	2.22 [0.63, 7.82]	2006
Yue, Z.2006	10	33	3	28	0.8%	3.62 [0.89, 14.83]	2006
Zhang, Z.2006	6	16	2	16	0.4%	4.20 [0.70, 25.26]	2006
Zhao, Z.2006	9	32	5	32	1.2%	2.11 [0.62, 7.20]	2006
Fang, H.2006	6	24	2	22	0.5%	3.33 [0.60, 18.66]	2006
Pan, Y.2006	16	28	4	20	0.7%	5.33 [1.41, 20.10]	2006
Chen, Z.2006	4	12	2	11	0.5%	2.25 [0.32, 15.76]	2006
Sun, C.2007	13	24	4	24	0.6%	5.91 [1.55, 22.58]	2007
Yin, W.2007	6	19	4	19	0.9%	1.73 [0.40, 7.51]	2007
Zhou, F.2007	5	16	0	12	0.1%	11.96 [0.59, 241.06]	2007
Zheng, F.2007	4	24	3	20	0.9%	1.13 [0.22, 5.79]	2007
Zhang, M.2008	11	33	4	34	0.9%	3.75 [1.05, 13.35]	2008
Huang, W.2008	12	28	9	28	1.8%	1.58 [0.53, 4.71]	2008
Li, X.2008	8	25	6	25	1.4%	1.49 [0.43, 5.17]	2008
Zhang, L.2009	11	34	4	35	0.9%	3.71 [1.05, 13.13]	2009
Wang, H.2009	12	28	9	28	1.8%	1.58 [0.53, 4.71]	2009
Gao, H.2009	17	35	6	30	1.1%	3.78 [1.24, 11.50]	2009
Qu, Y.2010	4	40	2	40	0.6%	2.11 [0.36, 12.24]	2010
Cheng, J.2010	5	30	0	30	0.1%	13.16 [0.69, 249.48]	2010
Gui, Q. 2010	9	25	3	25	0.7%	4.13 [0.96, 17.70]	2010
Li, Z. 2010	100	230	70	230	13.7%	1.76 [1.20, 2.58]	2010
Liu, Q.2010	5	27	3	20	1.0%	1.29 [0.27, 6.16]	2010
Zhang, C. 2011	13	19	4	19	0.4%	8.13 [1.87, 35.23]	2011
Xing, Q.2012	7	19	3	19	0.7%	3.11 [0.66, 14.60]	2012
Xu, J.2012	14	27	9	27	1.5%	2.15 [0.72, 6.47]	2012
Xu, Q.2012	11	27	9	27	1.8%	1.38 [0.45, 4.17]	2012
Yu, D.2012	17	25	12	25	1.3%	2.30 [0.73, 7.27]	2012
Qin, Q.2012	19	34	6	34	0.9%	5.91 [1.94, 17.97]	2012
Du, Y.2013	31	60	22	60	3.7%	1.85 [0.89, 3.83]	2013
Li, L. 2013	4	15	0	15	0.1%	12.13 [0.59, 248.49]	2013
Yu, L. 2013	2	23	1	22	0.3%	2.00 [0.17, 23.78]	2013
Zhao, Y. 2014	25	40	16	40	2.1%	2.50 [1.02, 6.15]	2014
Tu, D.2014	14	38	14	38	3.1%	1.00 [0.39, 2.54]	2014
Yao, X.2014	13	25	7	25	1.2%	2.79 [0.86, 9.01]	2014
Zhang, J.2014	13	40	6	40	1.4%	2.73 [0.92, 8.13]	2014
Chen, J. 2014	6	30	2	30	0.6%	3.50 [0.65, 18.98]	2014
Zhang, Z.2015	28	45	17	45	2.2%	2.71 [1.16, 6.36]	2015
Cai,C.2015	11	21	6	21	1.0%	2.75 [0.77, 9.86]	2015
Zhou, Z.2015	6	21	1	21	0.2%	8.00 [0.87, 73.68]	2015
Liu, J. 2015	15	30	7	29	1.2%	3.14 [1.03, 9.55]	2015
Yan, L.2016	16	33	7	28	1.3%	2.82 [0.95, 8.44]	2016
Li,D.2016	27	40	19	40	2.1%	2.30 [0.93, 5.69]	2016
Yu, M.2017	7	20	3	20	0.7%	3.05 [0.66, 14.14]	2017
Luo, D.2018	12	30	6	30	1.2%	2.67 [0.84, 8.46]	2018
Wang, Y. 2018	20	37	17	37	2.7%	1.38 [0.55, 3.45]	2018
Liu, Y. 2019	28	50	7	50	1.1%	7.82 [2.95, 20.72]	2019
Wu, Y. 2019	15	46	7	46	1.6%	2.70 [0.98, 7.43]	2019
**Subtotal (95% CI)**		**2357**		**2209**	**94.5%**	**2.61 [2.28, 2.98]**	
Total events	991		501				
Heterogeneity: Chi² = 48.86, df = 71 (P = 0.98); I² = 0%							
Test for overall effect: Z = 14.08 (P < 0.00001)							
**Low vs high-dosage**							
Xu, Q.2003	20	32	12	30	1.6%	2.50 [0.90, 6.95]	2003
Zhang, J.2003	9	25	2	28	0.4%	7.31 [1.40, 38.22]	2003
Fang, X.2004	3	15	1	15	0.3%	3.50 [0.32, 38.23]	2004
Huang, Y.2005	9	27	4	21	1.0%	2.13 [0.55, 8.21]	2005
Li, W. 2013	13	30	11	30	2.2%	1.32 [0.47, 3.72]	2013
**Subtotal (95% CI)**		**129**		**124**	**5.5%**	**2.38 [1.35, 4.20]**	
Total events	54		30				
Heterogeneity: Chi² = 3.15, df = 4 (P = 0.53); I² = 0%							
Test for overall effect: Z = 3.00 (P = 0.003)							
**Total (95% CI)**		**2486**		**2333**	**100.0%**	**2.59 [2.28, 2.95]**	
Total events	1045		531				
Heterogeneity: Chi² = 52.12, df = 76 (P = 0.98); I² = 0%							
Test for overall effect: Z = 14.40 (P < 0.00001)							
Test for subgroup differences: Chi² = 0.09, df = 1 (P = 0.76), I² = 0%							

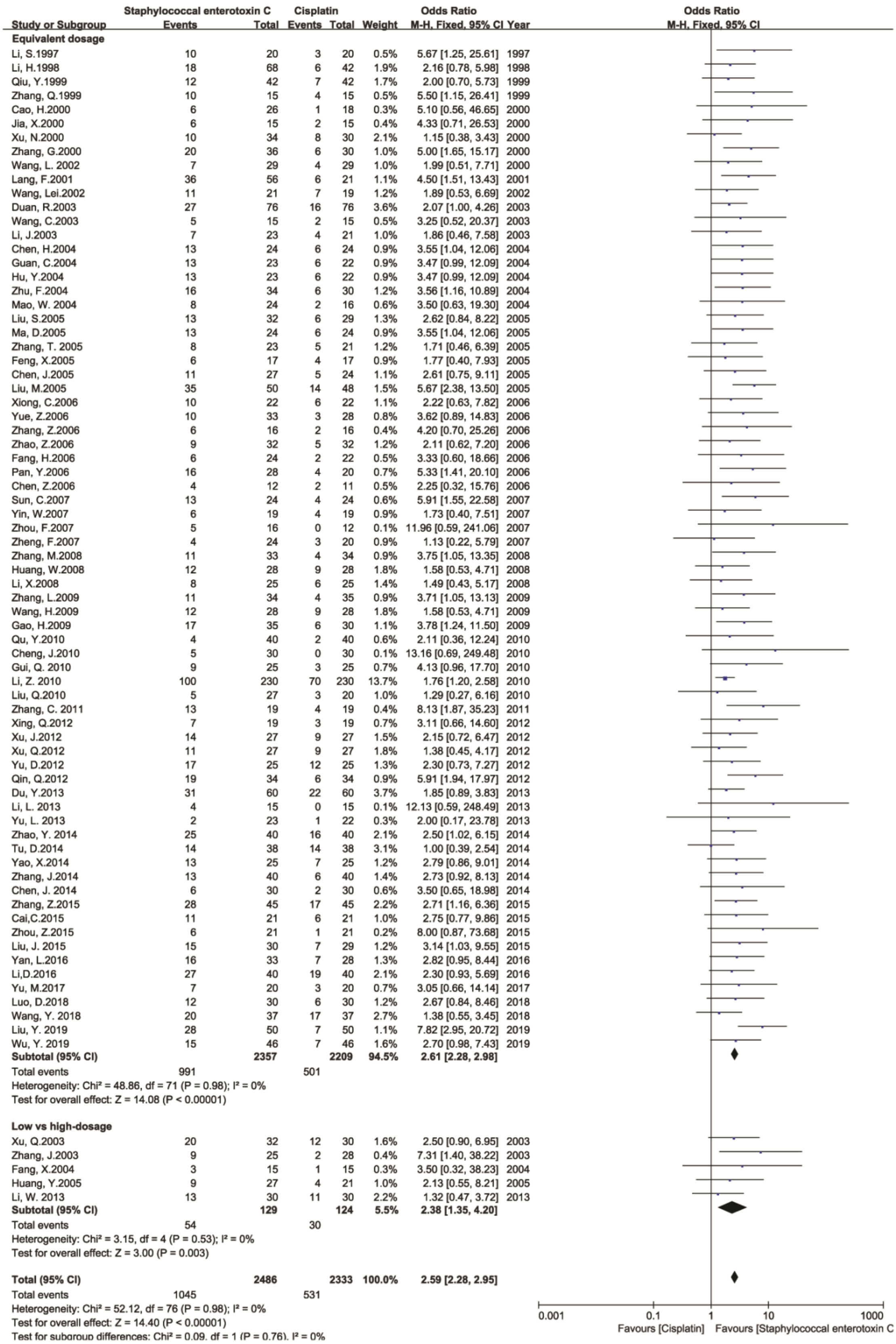

Fig.S54 Subgroups analysis of complete response via dosage difference of cisplatin

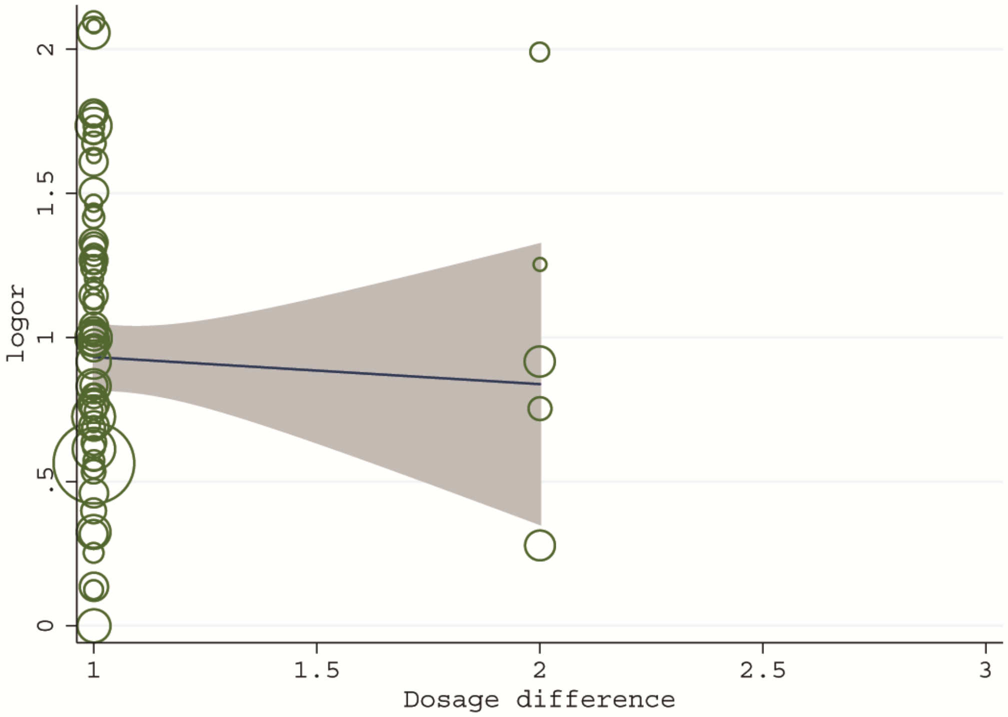

Fig.S55 Meta regression of complete response via dosage difference of cisplatin

Study or Subgroup	Staphylococcal enterotoxin C Events	Staphylococcal enterotoxin C Total	Cisplatin Events	Cisplatin Total	Weight	Odds Ratio M-H, Fixed, 95% CI	Year
**Equivalent dosage**							
Li, S.1997	3	20	11	20	1.0%	0.14 [0.03, 0.65]	1997
Li, H.1998	5	68	23	42	2.8%	0.07 [0.02, 0.20]	1998
Qiu, Y.1999	7	42	17	42	1.5%	0.29 [0.11, 0.81]	1999
Zhang, Q.1999	1	15	2	15	0.2%	0.46 [0.04, 5.75]	1999
Xu, N.2000	6	34	12	30	1.1%	0.32 [0.10, 1.01]	2000
Zhang, G.2000	4	36	12	30	1.2%	0.19 [0.05, 0.67]	2000
Cao, H.2000	7	26	11	18	1.0%	0.23 [0.06, 0.85]	2000
Jia, X.2000	2	15	8	15	0.7%	0.13 [0.02, 0.82]	2000
Wang, L. 2002	5	29	12	29	1.1%	0.30 [0.09, 0.99]	2000
Lang, F.2001	6	56	12	21	1.7%	0.09 [0.03, 0.30]	2001
Wang, Lei.2002	3	21	8	19	0.8%	0.23 [0.05, 1.05]	2002
Li, J.2003	4	23	10	21	0.9%	0.23 [0.06, 0.92]	2003
Wang, C.2003	3	15	7	15	0.6%	0.29 [0.06, 1.45]	2003
Duan, R.2003	10	76	37	76	3.4%	0.16 [0.07, 0.36]	2003
Hu, Y.2004	4	23	10	22	0.9%	0.25 [0.06, 0.99]	2004
Wang, C. 2004	5	23	12	22	1.0%	0.23 [0.06, 0.85]	2004
Zhu, F.2004	6	34	15	30	1.4%	0.21 [0.07, 0.67]	2004
Chen, H.2004	3	24	10	24	0.9%	0.20 [0.05, 0.86]	2004
Mao, W. 2004	4	24	7	16	0.7%	0.26 [0.06, 1.11]	2004
Guan, C.2004	4	23	10	22	0.9%	0.25 [0.06, 0.99]	2004
Zhang, T. 2005	4	23	10	21	0.9%	0.23 [0.06, 0.92]	2005
Chen, J.2005	2	27	7	24	0.7%	0.19 [0.04, 1.05]	2005
Feng, X.2005	2	17	7	17	0.7%	0.19 [0.03, 1.11]	2005
Liu, S.2005	5	32	14	29	1.3%	0.20 [0.06, 0.66]	2005
Ma, D.2005	3	24	10	24	0.9%	0.20 [0.05, 0.86]	2005
Liu, M.2005	3	50	21	48	2.2%	0.08 [0.02, 0.30]	2005
Xiong, C.2006	4	22	12	22	1.1%	0.19 [0.05, 0.73]	2006
Yue, Z.2006	5	33	13	28	1.3%	0.21 [0.06, 0.69]	2006
Zhang, Z.2006	2	16	9	16	0.8%	0.11 [0.02, 0.66]	2006
Zhao, Z.2006	5	32	14	32	1.3%	0.24 [0.07, 0.78]	2006
Fang, H.2006	4	24	11	22	1.0%	0.20 [0.05, 0.78]	2006
Pan, Y.2006	3	28	10	20	1.1%	0.12 [0.03, 0.53]	2006
Wang, J.2006	4	30	7	30	0.6%	0.51 [0.13, 1.95]	2006
Chen, Z.2006	3	12	6	11	0.5%	0.28 [0.05, 1.62]	2006
Sun, C.2007	3	24	12	24	1.1%	0.14 [0.03, 0.61]	2007
Yin, W.2007	3	19	10	19	0.9%	0.17 [0.04, 0.78]	2007
Zhou, F.2007	2	16	7	12	0.7%	0.10 [0.02, 0.66]	2007
Zheng, F.2007	6	24	8	20	0.7%	0.50 [0.14, 1.81]	2007
Huang, W.2008	5	28	12	28	1.1%	0.29 [0.09, 0.98]	2008
Li, X.2008	4	25	11	25	1.0%	0.24 [0.06, 0.92]	2008
Zhang, M.2008	3	33	13	34	1.2%	0.16 [0.04, 0.64]	2008
Gao, H.2009	7	35	17	30	1.6%	0.19 [0.06, 0.57]	2009
Wang, H.2009	5	28	12	28	1.1%	0.29 [0.09, 0.98]	2009
Zhang, L.2009	3	34	12	35	1.2%	0.19 [0.05, 0.73]	2009
Liu, Q.2010	5	27	9	20	0.9%	0.28 [0.07, 1.03]	2010
Gui, Q. 2010	3	25	11	25	1.0%	0.17 [0.04, 0.73]	2010
Cheng, J.2010	6	30	15	30	1.3%	0.25 [0.08, 0.79]	2010
Qu, Y.2010	8	40	19	40	1.6%	0.28 [0.10, 0.75]	2010
Li, Z. 2010	50	230	110	230	9.2%	0.30 [0.20, 0.45]	2010
Zhang, C. 2011	1	19	9	19	0.9%	0.06 [0.01, 0.56]	2011
Qin, Q.2012	4	34	23	34	2.2%	0.06 [0.02, 0.23]	2012
Xing, Q.2012	2	19	12	19	1.1%	0.07 [0.01, 0.39]	2012
Xu, J.2012	3	27	12	27	1.1%	0.16 [0.04, 0.65]	2012
Xu, Q.2012	4	27	9	27	0.8%	0.35 [0.09, 1.31]	2012
Yu, D.2012	3	25	7	25	0.7%	0.35 [0.08, 1.55]	2012
Du, Y.2013	6	60	25	60	2.4%	0.16 [0.06, 0.42]	2013
Li, L. 2013	3	15	7	15	0.6%	0.29 [0.06, 1.45]	2013
Yu, L. 2013	4	23	11	22	1.0%	0.21 [0.05, 0.82]	2013
Zhao, Y. 2014	2	40	13	40	1.3%	0.11 [0.02, 0.52]	2014
Zhang, J.2014	8	40	25	40	2.1%	0.15 [0.05, 0.41]	2014
Chen, J. 2014	5	30	15	30	1.3%	0.20 [0.06, 0.66]	2014
Tu, D.2014	4	38	13	38	1.2%	0.23 [0.07, 0.78]	2014
Yao, X.2014	2	25	12	25	1.2%	0.09 [0.02, 0.49]	2014
Liu, J. 2015	4	30	13	29	1.2%	0.19 [0.05, 0.68]	2015
Zhou, Z.2015	4	21	11	21	1.0%	0.21 [0.05, 0.85]	2015
Cai,C.2015	3	21	7	21	0.6%	0.33 [0.07, 1.53]	2015
Zhang, Z.2015	2	45	18	45	1.8%	0.07 [0.01, 0.32]	2015
Li,D.2016	1	40	10	40	1.0%	0.08 [0.01, 0.63]	2016
Yan, L.2016	4	33	8	28	0.8%	0.34 [0.09, 1.30]	2016
Yu, M.2017	2	20	13	20	1.3%	0.06 [0.01, 0.34]	2017
Luo, D.2018	3	30	14	30	1.3%	0.13 [0.03, 0.51]	2018
Wang, Y. 2018	4	37	10	37	1.0%	0.33 [0.09, 1.16]	2018
Liu, Y. 2019	2	50	23	50	2.4%	0.05 [0.01, 0.22]	2019
Wu, Y. 2019	10	46	27	46	2.3%	0.20 [0.08, 0.49]	2019
**Subtotal (95% CI)**		**2410**		**2261**	**95.9%**	**0.20 [0.17, 0.23]**	
Total events	344		1022				
Heterogeneity: Chi² = 41.43, df = 73 (P = 1.00); I² = 0%							
Test for overall effect: Z = 22.25 (P < 0.00001)							
**Low vs high-dosage**							
Xu, Q.2003	4	32	10	30	1.0%	0.29 [0.08, 1.04]	2003
Zhang, J.2003	4	25	8	28	0.7%	0.48 [0.12, 1.83]	2003
Fang, X.2004	3	15	7	15	0.6%	0.29 [0.06, 1.45]	2004
Huang, Y.2005	4	27	9	21	0.9%	0.23 [0.06, 0.91]	2005
Li, W. 2013	3	30	10	30	1.0%	0.22 [0.05, 0.91]	2013
**Subtotal (95% CI)**		**129**		**124**	**4.1%**	**0.29 [0.16, 0.54]**	
Total events	18		44				
Heterogeneity: Chi² = 0.76, df = 4 (P = 0.94); I² = 0%							
Test for overall effect: Z = 3.90 (P < 0.0001)							
**Total (95% CI)**		**2539**		**2385**	**100.0%**	**0.20 [0.18, 0.23]**	
Total events	362		1066				
Heterogeneity: Chi² = 43.41, df = 78 (P = 1.00); I² = 0%							
Test for overall effect: Z = 22.57 (P < 0.00001)							
Test for subgroup differences: Chi² = 1.40, df = 1 (P = 0.24), I² = 28.6%							

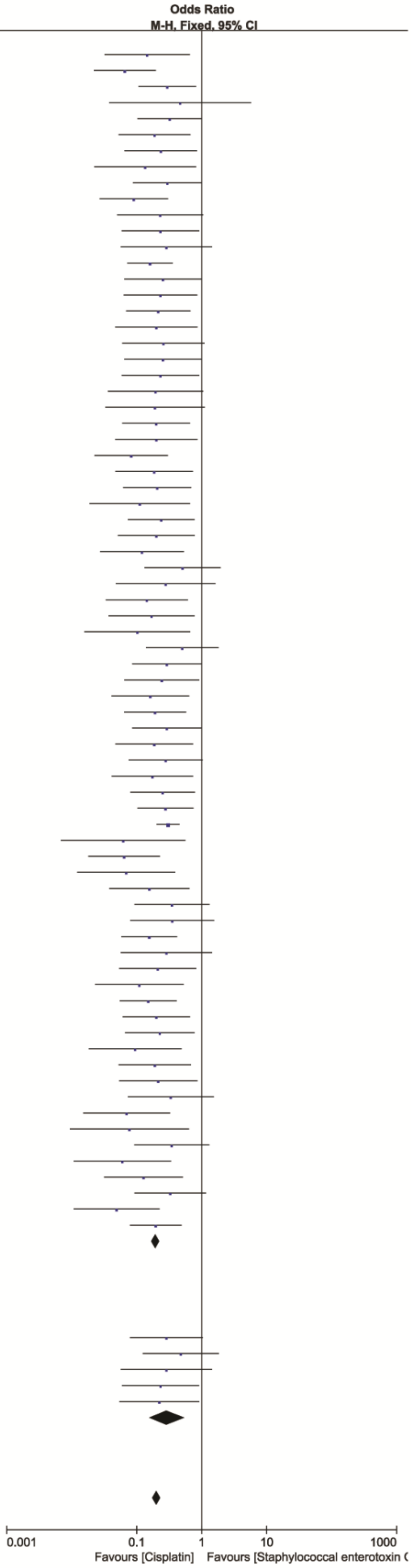

Fig.S56 Subgroups analysis of treatment failure via dosage difference of cisplatin

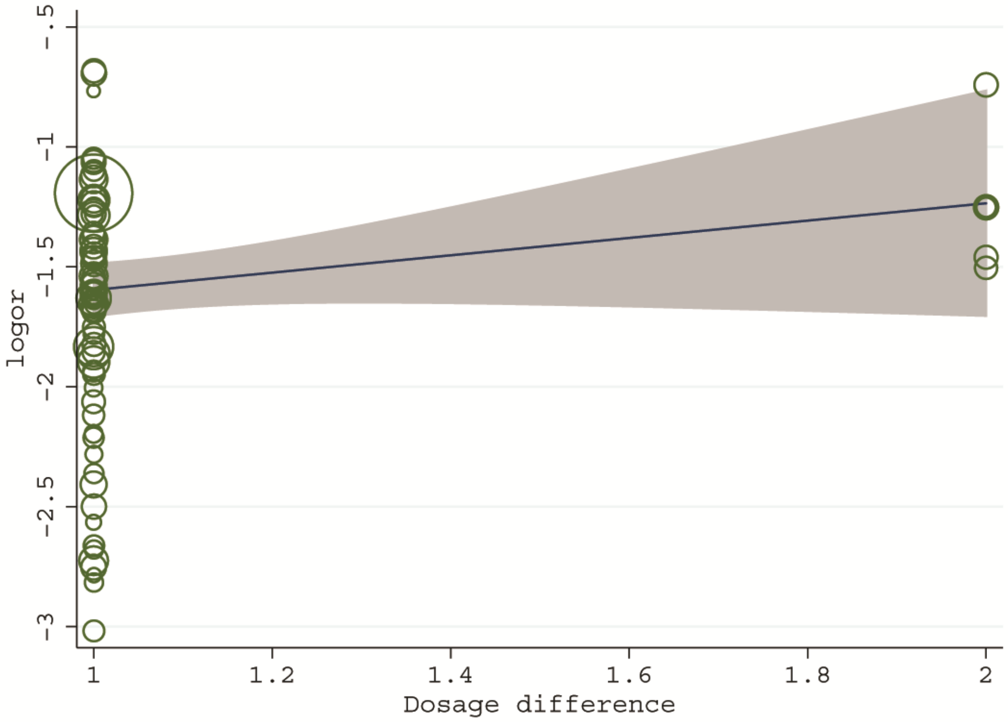

Fig.S57 Meta regression of treatment failure via dosage difference of cisplatin

	Staphylococcal enterotoxin C		Cisplatin			Odds Ratio		Odds Ratio
Study or Subgroup	Events	Total	Events	Total	Weight	M-H, Fixed, 95% CI	Year	M-H, Fixed, 95% CI
**Millar**								
Li, H.1998	18	68	6	42	1.9%	2.16 [0.78, 5.98]	1998	
Wang, L. 2002	7	29	4	29	1.0%	1.99 [0.51, 7.71]	2000	
Lang, F.2001	36	56	6	21	1.1%	4.50 [1.51, 13.43]	2001	
Li, J.2003	7	23	4	21	1.0%	1.86 [0.46, 7.58]	2003	
Liu, M.2005	35	50	14	48	1.5%	5.67 [2.38, 13.50]	2005	
Chen, Z.2006	4	12	2	11	0.5%	2.25 [0.32, 15.76]	2006	
Zheng, F.2007	4	24	3	20	0.9%	1.13 [0.22, 5.79]	2007	
Zhang, L.2009	11	34	4	35	0.9%	3.71 [1.05, 13.13]	2009	
Gui, Q. 2010	9	25	3	25	0.7%	4.13 [0.96, 17.70]	2010	
Li, Z. 2010	100	230	70	230	13.7%	1.76 [1.20, 2.58]	2010	
Liu, Q.2010	5	27	3	20	1.0%	1.29 [0.27, 6.16]	2010	
Zhou, Z.2015	6	21	1	21	0.2%	8.00 [0.87, 73.68]	2015	
Liu, J. 2015	15	30	7	29	1.2%	3.14 [1.03, 9.55]	2015	
**Subtotal (95% CI)**		**629**		**552**	**25.6%**	**2.37 [1.82, 3.08]**		
Total events	257		127					
Heterogeneity: Chi² = 11.55, df = 12 (P = 0.48); I² = 0%								
Test for overall effect: Z = 6.44 (P < 0.00001)								
**Ostrowskimj**								
Li, S.1997	10	20	3	20	0.5%	5.67 [1.25, 25.61]	1997	
Qiu, Y.1999	12	42	7	42	1.7%	2.00 [0.70, 5.73]	1999	
Zhang, Q.1999	10	15	4	15	0.5%	5.50 [1.15, 26.41]	1999	
Cao, H.2000	6	26	1	18	0.3%	5.10 [0.56, 46.65]	2000	
Jia, X.2000	6	15	2	15	0.4%	4.33 [0.71, 26.53]	2000	
Xu, N.2000	10	34	8	30	2.1%	1.15 [0.38, 3.43]	2000	
Zhang, G.2000	20	36	6	30	1.0%	5.00 [1.65, 15.17]	2000	
Wang, Lei.2002	11	21	7	19	1.2%	1.89 [0.53, 6.69]	2002	
Duan, R.2003	27	76	16	76	3.6%	2.07 [1.00, 4.26]	2003	
Wang, C.2003	5	15	2	15	0.5%	3.25 [0.52, 20.37]	2003	
Xu, Q.2003	20	32	12	30	1.6%	2.50 [0.90, 6.95]	2003	
Zhang, J.2003	9	25	2	28	0.4%	7.31 [1.40, 38.22]	2003	
Chen, H.2004	13	24	6	24	0.9%	3.55 [1.04, 12.06]	2004	
Fang, X.2004	3	15	1	15	0.3%	3.50 [0.32, 38.23]	2004	
Guan, C.2004	13	23	6	22	0.9%	3.47 [0.99, 12.09]	2004	
Hu, Y.2004	13	23	6	22	0.9%	3.47 [0.99, 12.09]	2004	
Zhu, F.2004	16	34	6	30	1.2%	3.56 [1.16, 10.89]	2004	
Mao, W. 2004	8	24	2	16	0.6%	3.50 [0.63, 19.30]	2004	
Liu, S.2005	13	32	6	29	1.3%	2.62 [0.84, 8.22]	2005	
Ma, D.2005	13	24	6	24	0.9%	3.55 [1.04, 12.06]	2005	
Zhang, T. 2005	8	23	5	21	1.2%	1.71 [0.46, 6.39]	2005	
Feng, X.2005	6	17	4	17	0.9%	1.77 [0.40, 7.93]	2005	
Chen, J.2005	11	27	5	24	1.1%	2.61 [0.75, 9.11]	2005	
Huang, Y.2005	9	27	4	21	1.0%	2.13 [0.55, 8.21]	2005	
Xiong, C.2006	10	22	6	22	1.1%	2.22 [0.63, 7.82]	2006	
Yue, Z.2006	10	33	3	28	0.8%	3.62 [0.89, 14.83]	2006	
Zhang, Z.2006	6	16	2	16	0.4%	4.20 [0.70, 25.26]	2006	
Zhao, Z.2006	9	32	5	32	1.2%	2.11 [0.62, 7.20]	2006	
Fang, H.2006	6	24	2	22	0.5%	3.33 [0.60, 18.66]	2006	
Pan, Y.2006	16	28	4	20	0.7%	5.33 [1.41, 20.10]	2006	
Sun, C.2007	13	24	4	24	0.6%	5.91 [1.55, 22.58]	2007	
Yin, W.2007	6	19	4	19	0.9%	1.73 [0.40, 7.51]	2007	
Zhou, F.2007	5	16	0	12	0.1%	11.96 [0.59, 241.06]	2007	
Zhang, M.2008	11	33	4	34	0.9%	3.75 [1.05, 13.35]	2008	
Huang, W.2008	12	28	9	28	1.8%	1.58 [0.53, 4.71]	2008	
Li, X.2008	8	25	6	25	1.4%	1.49 [0.43, 5.17]	2008	
Wang, H.2009	12	28	9	28	1.8%	1.58 [0.53, 4.71]	2009	
Gao, H.2009	17	35	6	30	1.1%	3.78 [1.24, 11.50]	2009	
Qu, Y.2010	4	40	2	40	0.6%	2.11 [0.36, 12.24]	2010	
Cheng, J.2010	5	30	0	30	0.1%	13.16 [0.69, 249.48]	2010	
Zhang, C. 2011	13	19	4	19	0.4%	8.13 [1.87, 35.23]	2011	
Xing, Q.2012	7	19	3	19	0.7%	3.11 [0.66, 14.60]	2012	
Xu, J.2012	14	27	9	27	1.5%	2.15 [0.72, 6.47]	2012	
Xu, Q.2012	11	27	9	27	1.8%	1.38 [0.45, 4.17]	2012	
Yu, D.2012	17	25	12	25	1.3%	2.30 [0.73, 7.27]	2012	
Qin, Q.2012	19	34	6	34	0.9%	5.91 [1.94, 17.97]	2012	
Li, W. 2013	13	30	11	30	2.2%	1.32 [0.47, 3.72]	2013	
Du, Y.2013	31	60	22	60	3.7%	1.85 [0.89, 3.83]	2013	
Li, L. 2013	4	15	0	15	0.1%	12.13 [0.59, 248.49]	2013	
Yu, L. 2013	2	23	1	22	0.3%	2.00 [0.17, 23.78]	2013	
Zhao, Y. 2014	25	40	16	40	2.1%	2.50 [1.02, 6.15]	2014	
Tu, D.2014	14	38	14	38	3.1%	1.00 [0.39, 2.54]	2014	
Yao, X.2014	13	25	7	25	1.2%	2.79 [0.86, 9.01]	2014	
Zhang, J.2014	13	40	6	40	1.4%	2.73 [0.92, 8.13]	2014	
Chen, J. 2014	6	30	2	30	0.6%	3.50 [0.65, 18.98]	2014	
Zhang, Z.2015	28	45	17	45	2.2%	2.71 [1.16, 6.36]	2015	
Cai,C.2015	11	21	6	21	1.0%	2.75 [0.77, 9.86]	2015	
Yan, L.2016	16	33	7	28	1.3%	2.82 [0.95, 8.44]	2016	
Li,D.2016	27	40	19	40	2.1%	2.30 [0.93, 5.69]	2016	
Yu, M.2017	7	20	3	20	0.7%	3.05 [0.66, 14.14]	2017	
Luo, D.2018	12	30	6	30	1.2%	2.67 [0.84, 8.46]	2018	
Wang, Y. 2018	20	37	17	37	2.7%	1.38 [0.55, 3.45]	2018	
Liu, Y. 2019	28	50	7	50	1.1%	7.82 [2.95, 20.72]	2019	
Wu, Y. 2019	15	46	7	46	1.6%	2.70 [0.98, 7.43]	2019	
**Subtotal (95% CI)**		**1857**		**1781**	**74.4%**	**2.67 [2.30, 3.10]**		
Total events	788		404					
Heterogeneity: Chi² = 40.04, df = 63 (P = 0.99); I² = 0%								
Test for overall effect: Z = 12.89 (P < 0.00001)								
**Total (95% CI)**		**2486**		**2333**	**100.0%**	**2.59 [2.28, 2.95]**		
Total events	1045		531					
Heterogeneity: Chi² = 52.12, df = 76 (P = 0.98); I² = 0%								
Test for overall effect: Z = 14.40 (P < 0.00001)								
Test for subgroup differences: Chi² = 0.61, df = 1 (P = 0.43), I² = 0%								

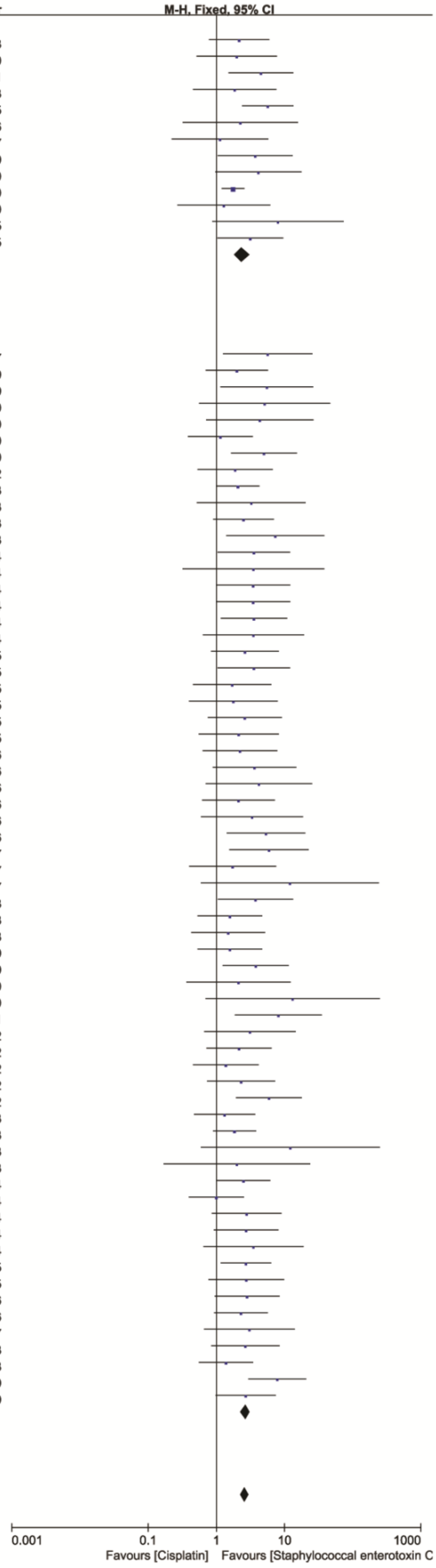

Fig.S58 Subgroups analysis of complete response via evaluation criteria

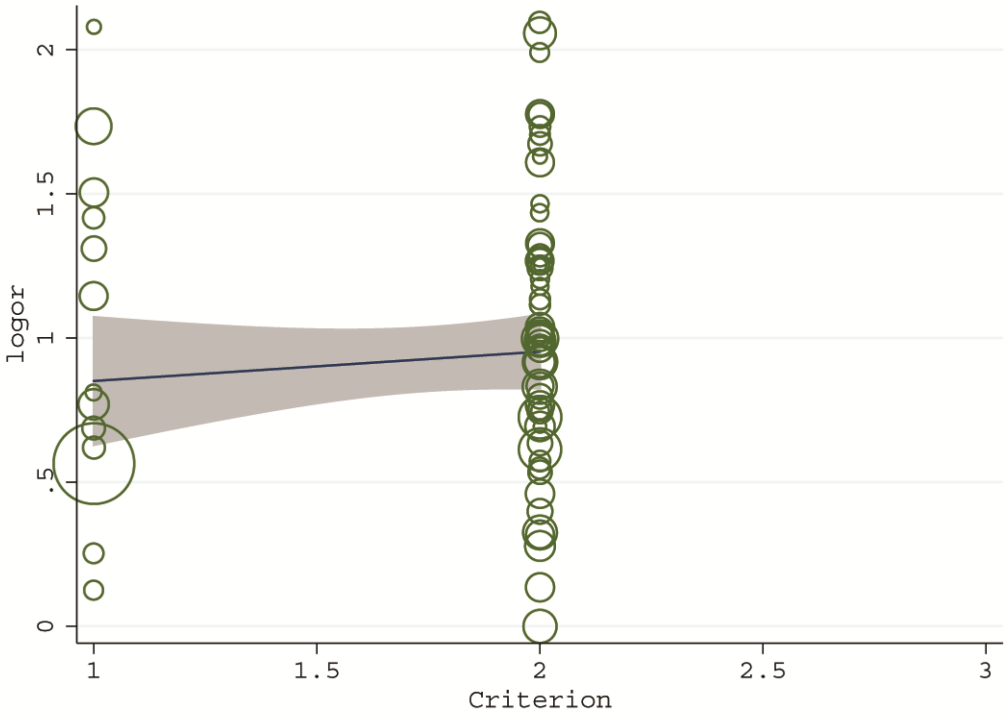

Fig.S59 Meta regression of complete response via evaluation criteria

Study or Subgroup	Staphylococcal enterotoxin C Events	Total	Cisplatin Events	Total	Weight	Odds Ratio M-H, Fixed, 95% CI	Year
**Millar**							
Li, H.1998	5	68	23	42	2.8%	0.07 [0.02, 0.20]	1998
Wang, L. 2002	5	29	12	29	1.1%	0.30 [0.09, 0.99]	2000
Lang, F.2001	6	56	12	21	1.7%	0.09 [0.03, 0.30]	2001
Li, J.2003	4	23	10	21	0.9%	0.23 [0.06, 0.92]	2003
Wang, C. 2004	5	23	12	22	1.0%	0.23 [0.06, 0.85]	2004
Liu, M.2005	3	50	21	48	2.2%	0.08 [0.02, 0.30]	2005
Wang, J.2006	4	30	7	30	0.6%	0.51 [0.13, 1.95]	2006
Chen, Z.2006	3	12	6	11	0.5%	0.28 [0.05, 1.62]	2006
Zheng, F.2007	6	24	8	20	0.7%	0.50 [0.14, 1.81]	2007
Zhang, L.2009	3	34	12	35	1.2%	0.19 [0.05, 0.73]	2009
Li, Z. 2010	50	230	110	230	9.2%	0.30 [0.20, 0.45]	2010
Liu, Q.2010	5	27	9	20	0.9%	0.28 [0.07, 1.03]	2010
Gui, Q. 2010	3	25	11	25	1.0%	0.17 [0.04, 0.73]	2010
Liu, J. 2015	4	30	13	29	1.2%	0.19 [0.05, 0.68]	2015
Zhou, Z.2015	4	21	11	21	1.0%	0.21 [0.05, 0.85]	2015
**Subtotal (95% CI)**		**682**		**604**	**26.0%**	**0.23 [0.18, 0.30]**	
Total events	110		277				
Heterogeneity: Chi² = 14.88, df = 14 (P = 0.39); I² = 6%							
Test for overall effect: Z = 11.03 (P < 0.00001)							
**Ostrowskimj**							
Li, S.1997	3	20	11	20	1.0%	0.14 [0.03, 0.65]	1997
Qiu, Y.1999	7	42	17	42	1.5%	0.29 [0.11, 0.81]	1999
Zhang, Q.1999	1	15	2	15	0.2%	0.46 [0.04, 5.75]	1999
Xu, N.2000	6	34	12	30	1.1%	0.32 [0.10, 1.01]	2000
Zhang, G.2000	4	36	12	30	1.2%	0.19 [0.05, 0.67]	2000
Cao, H.2000	7	26	11	18	1.0%	0.23 [0.06, 0.85]	2000
Jia, X.2000	2	15	8	15	0.7%	0.13 [0.02, 0.82]	2000
Wang, Lei.2002	3	21	8	19	0.8%	0.23 [0.05, 1.05]	2002
Wang, C.2003	3	15	7	15	0.6%	0.29 [0.06, 1.45]	2003
Xu, Q.2003	4	32	10	30	1.0%	0.29 [0.08, 1.04]	2003
Zhang, J.2003	4	25	8	28	0.7%	0.48 [0.12, 1.83]	2003
Duan, R.2003	10	76	37	76	3.4%	0.16 [0.07, 0.36]	2003
Hu, Y.2004	4	23	10	22	0.9%	0.25 [0.06, 0.99]	2004
Zhu, F.2004	6	34	15	30	1.4%	0.21 [0.07, 0.67]	2004
Chen, H.2004	3	24	10	24	0.9%	0.20 [0.05, 0.86]	2004
Mao, W. 2004	4	24	7	16	0.7%	0.26 [0.06, 1.11]	2004
Fang, X.2004	3	15	7	15	0.6%	0.29 [0.06, 1.45]	2004
Guan, C.2004	4	23	10	22	0.9%	0.25 [0.06, 0.99]	2004
Zhang, T. 2005	4	23	10	21	0.9%	0.23 [0.06, 0.92]	2005
Chen, J.2005	2	27	7	24	0.7%	0.19 [0.04, 1.05]	2005
Feng, X.2005	2	17	7	17	0.7%	0.19 [0.03, 1.11]	2005
Huang, Y.2005	4	27	9	21	0.9%	0.23 [0.06, 0.91]	2005
Liu, S.2005	5	32	14	29	1.3%	0.20 [0.06, 0.66]	2005
Ma, D.2005	3	24	10	24	0.9%	0.20 [0.05, 0.86]	2005
Xiong, C.2006	4	22	12	22	1.1%	0.19 [0.05, 0.73]	2006
Yue, Z.2006	5	33	13	28	1.3%	0.21 [0.06, 0.69]	2006
Zhang, Z.2006	2	16	9	16	0.8%	0.11 [0.02, 0.66]	2006
Zhao, Z.2006	5	32	14	32	1.3%	0.24 [0.07, 0.78]	2006
Fang, H.2006	4	24	11	22	1.0%	0.20 [0.05, 0.78]	2006
Pan, Y.2006	3	28	10	20	1.1%	0.12 [0.03, 0.53]	2006
Sun, C.2007	3	24	12	24	1.1%	0.14 [0.03, 0.61]	2007
Yin, W.2007	3	19	10	19	0.9%	0.17 [0.04, 0.78]	2007
Zhou, F.2007	2	16	7	12	0.7%	0.10 [0.02, 0.66]	2007
Huang, W.2008	5	28	12	28	1.1%	0.29 [0.09, 0.98]	2008
Li, X.2008	4	25	11	25	1.0%	0.24 [0.06, 0.92]	2008
Zhang, M.2008	3	33	13	34	1.2%	0.16 [0.04, 0.64]	2008
Gao, H.2009	7	35	17	30	1.6%	0.19 [0.06, 0.57]	2009
Wang, H.2009	5	28	12	28	1.1%	0.29 [0.09, 0.98]	2009
Cheng, J.2010	6	30	15	30	1.3%	0.25 [0.08, 0.79]	2010
Qu, Y.2010	8	40	19	40	1.6%	0.28 [0.10, 0.75]	2010
Zhang, C. 2011	1	19	9	19	0.9%	0.06 [0.01, 0.56]	2011
Yu, D.2012	3	25	7	25	0.7%	0.35 [0.08, 1.55]	2012
Qin, Q.2012	4	34	23	34	2.2%	0.06 [0.02, 0.23]	2012
Xing, Q.2012	2	19	12	19	1.1%	0.07 [0.01, 0.39]	2012
Xu, J.2012	3	27	12	27	1.1%	0.16 [0.04, 0.65]	2012
Xu, Q.2012	4	27	9	27	0.8%	0.35 [0.09, 1.31]	2012
Du, Y.2013	6	60	25	60	2.4%	0.16 [0.06, 0.42]	2013
Li, L. 2013	3	15	7	15	0.6%	0.29 [0.06, 1.45]	2013
Li, W. 2013	3	30	10	30	1.0%	0.22 [0.05, 0.91]	2013
Yu, L. 2013	4	23	11	22	1.0%	0.21 [0.05, 0.82]	2013
Zhao, Y. 2014	2	40	13	40	1.3%	0.11 [0.02, 0.52]	2014
Zhang, J.2014	8	40	25	40	2.1%	0.15 [0.05, 0.41]	2014
Chen, J. 2014	5	30	15	30	1.3%	0.20 [0.06, 0.66]	2014
Tu, D.2014	4	38	13	38	1.2%	0.23 [0.07, 0.78]	2014
Yao, X.2014	2	25	12	25	1.2%	0.09 [0.02, 0.49]	2014
Cai,C.2015	3	21	7	21	0.6%	0.33 [0.07, 1.53]	2015
Zhang, Z.2015	2	45	18	45	1.8%	0.07 [0.01, 0.32]	2015
Li,D.2016	1	40	10	40	1.0%	0.08 [0.01, 0.63]	2016
Yan, L.2016	4	33	8	28	0.8%	0.34 [0.09, 1.30]	2016
Yu, M.2017	2	20	13	20	1.3%	0.06 [0.01, 0.34]	2017
Luo, D.2018	3	30	14	30	1.3%	0.13 [0.03, 0.51]	2018
Wang, Y. 2018	4	37	10	37	1.0%	0.33 [0.09, 1.16]	2018
Liu, Y. 2019	2	50	23	50	2.4%	0.05 [0.01, 0.22]	2019
Wu, Y. 2019	10	46	27	46	2.3%	0.20 [0.08, 0.49]	2019
**Subtotal (95% CI)**		**1857**		**1781**	**74.0%**	**0.19 [0.16, 0.23]**	
Total events	252		789				
Heterogeneity: Chi² = 27.36, df = 63 (P = 1.00); I² = 0%							
Test for overall effect: Z = 19.69 (P < 0.00001)							
**Total (95% CI)**		**2539**		**2385**	**100.0%**	**0.20 [0.18, 0.23]**	
Total events	362		1066				
Heterogeneity: Chi² = 43.41, df = 78 (P = 1.00); I² = 0%							
Test for overall effect: Z = 22.57 (P < 0.00001)							
Test for subgroup differences: Chi² = 1.35, df = 1 (P = 0.25), I² = 25.8%							

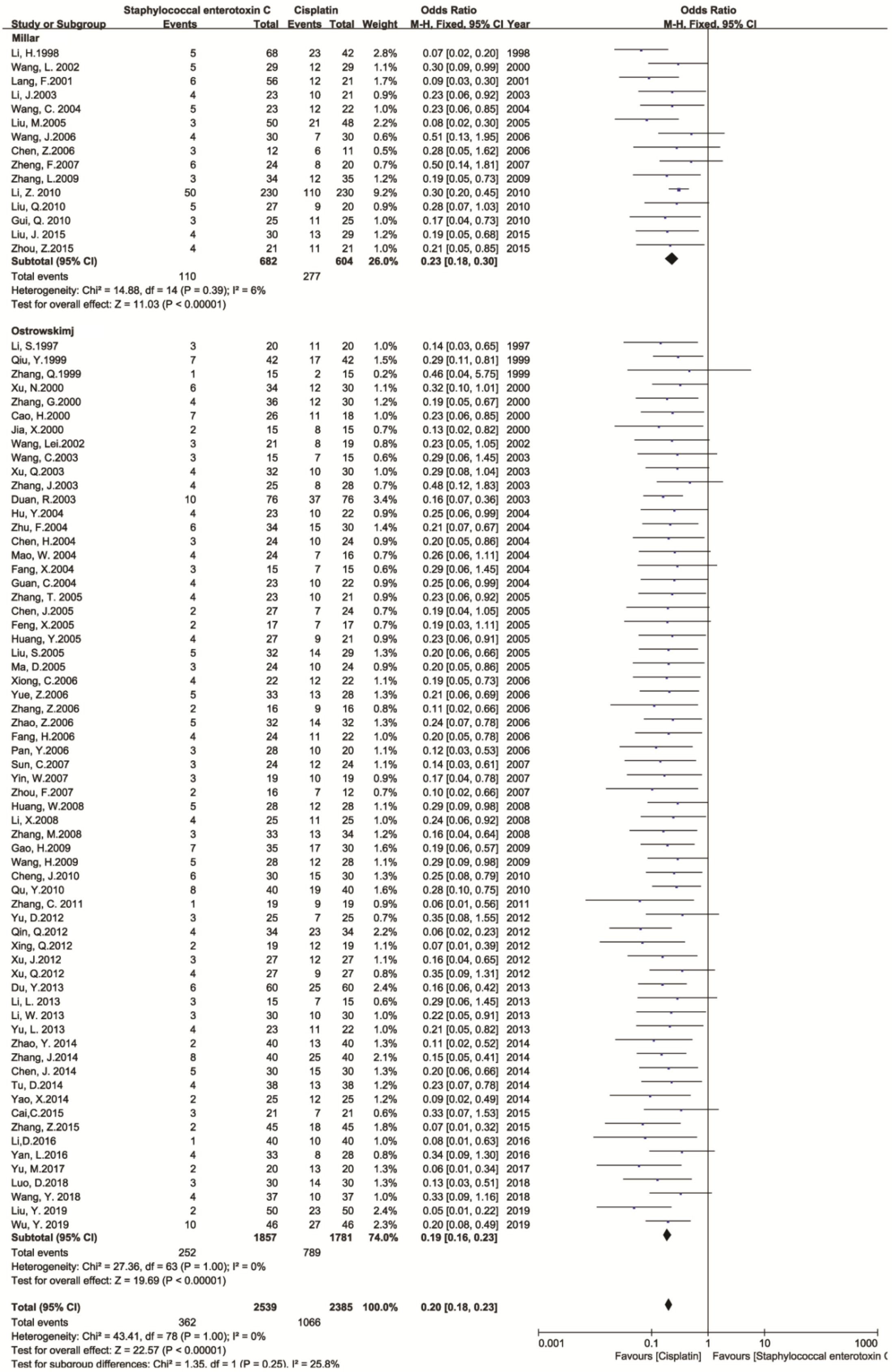

Fig.S60 Subgroups analysis of treatment failure via evaluation criteria

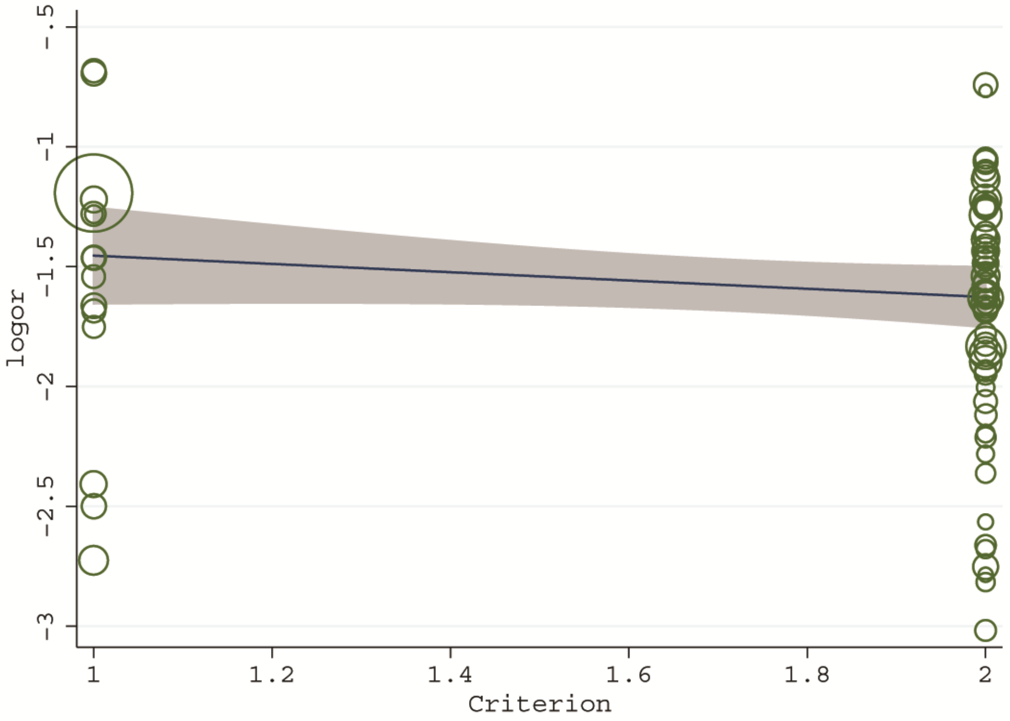

Fig.S61 Meta regression of treatment failure via evaluation criteria

Study or Subgroup	Staphylococcal enterotoxin C Events	Staphylococcal enterotoxin C Total	Cisplatin Events	Cisplatin Total	Weight	Odds Ratio M-H, Fixed, 95% CI	Year
**Before 2010 year**							
Li, S.1997	10	20	3	20	0.5%	5.67 [1.25, 25.61]	1997
Li, H.1998	18	68	6	42	1.9%	2.16 [0.78, 5.98]	1998
Qiu, Y.1999	12	42	7	42	1.7%	2.00 [0.70, 5.73]	1999
Zhang, Q.1999	10	15	4	15	0.5%	5.50 [1.15, 26.41]	1999
Wang, L. 2002	7	29	4	29	1.0%	1.99 [0.51, 7.71]	2000
Xu, N.2000	10	34	8	30	2.1%	1.15 [0.38, 3.43]	2000
Zhang, G.2000	20	36	6	30	1.0%	5.00 [1.65, 15.17]	2000
Cao, H.2000	6	26	1	18	0.3%	5.10 [0.56, 46.65]	2000
Jia, X.2000	6	15	2	15	0.4%	4.33 [0.71, 26.53]	2000
Lang, F.2001	36	56	6	21	1.1%	4.50 [1.51, 13.43]	2001
Wang, Lei.2002	11	21	7	19	1.2%	1.89 [0.53, 6.69]	2002
Duan, R.2003	27	76	16	76	3.6%	2.07 [1.00, 4.26]	2003
Li, J.2003	7	23	4	21	1.0%	1.86 [0.46, 7.58]	2003
Wang, C.2003	5	15	2	15	0.5%	3.25 [0.52, 20.37]	2003
Xu, Q.2003	20	32	12	30	1.6%	2.50 [0.90, 6.95]	2003
Zhang, J.2003	9	25	2	28	0.4%	7.31 [1.40, 38.22]	2003
Chen, H.2004	13	24	6	24	0.9%	3.55 [1.04, 12.06]	2004
Fang, X.2004	3	15	1	15	0.3%	3.50 [0.32, 38.23]	2004
Guan, C.2004	13	23	6	22	0.9%	3.47 [0.99, 12.09]	2004
Hu, Y.2004	13	23	6	22	0.9%	3.47 [0.99, 12.09]	2004
Zhu, F.2004	16	34	6	30	1.2%	3.56 [1.16, 10.89]	2004
Mao, W. 2004	8	24	2	16	0.6%	3.50 [0.63, 19.30]	2004
Liu, S.2005	13	32	6	29	1.3%	2.62 [0.84, 8.22]	2005
Ma, D.2005	13	24	6	24	0.9%	3.55 [1.04, 12.06]	2005
Zhang, T. 2005	8	23	5	21	1.2%	1.71 [0.46, 6.39]	2005
Feng, X.2005	6	17	4	17	0.9%	1.77 [0.40, 7.93]	2005
Chen, J.2005	11	27	5	24	1.1%	2.61 [0.75, 9.11]	2005
Huang, Y.2005	9	27	4	21	1.0%	2.13 [0.55, 8.21]	2005
Liu, M.2005	35	50	14	48	1.5%	5.67 [2.38, 13.50]	2005
Xiong, C.2006	10	22	6	22	1.1%	2.22 [0.63, 7.82]	2006
Yue, Z.2006	10	33	3	28	0.8%	3.62 [0.89, 14.83]	2006
Zhang, Z.2006	6	16	2	16	0.4%	4.20 [0.70, 25.26]	2006
Zhao, Z.2006	9	32	5	32	1.2%	2.11 [0.62, 7.20]	2006
Chen, Z.2006	4	12	2	11	0.5%	2.25 [0.32, 15.76]	2006
Fang, H.2006	6	24	2	22	0.5%	3.33 [0.60, 18.66]	2006
Pan, Y.2006	16	28	4	20	0.7%	5.33 [1.41, 20.10]	2006
Sun, C.2007	13	24	4	24	0.6%	5.91 [1.55, 22.58]	2007
Yin, W.2007	6	19	4	19	0.9%	1.73 [0.40, 7.51]	2007
Zheng, F.2007	4	24	3	20	0.9%	1.13 [0.22, 5.79]	2007
Zhou, F.2007	5	16	0	12	0.1%	11.96 [0.59, 241.06]	2007
Zhang, M.2008	11	33	4	34	0.9%	3.75 [1.05, 13.35]	2008
Huang, W.2008	12	28	9	28	1.8%	1.58 [0.53, 4.71]	2008
Li, X.2008	8	25	6	25	1.4%	1.49 [0.43, 5.17]	2008
Wang, H.2009	12	28	9	28	1.8%	1.58 [0.53, 4.71]	2009
Zhang, L.2009	11	34	4	35	0.9%	3.71 [1.05, 13.13]	2009
Gao, H.2009	17	35	6	30	1.1%	3.78 [1.24, 11.50]	2009
**Subtotal (95% CI)**		**1309**		**1170**	**47.3%**	**2.84 [2.36, 3.42]**	
Total events	535		234				
Heterogeneity: Chi² = 23.52, df = 45 (P = 1.00); I² = 0%							
Test for overall effect: Z = 11.04 (P < 0.00001)							
**From 2010 to now**							
Cheng, J.2010	5	30	0	30	0.1%	13.16 [0.69, 249.48]	2010
Gui, Q. 2010	9	25	3	25	0.7%	4.13 [0.96, 17.70]	2010
Li, Z. 2010	100	230	70	230	13.7%	1.76 [1.20, 2.58]	2010
Liu, Q.2010	5	27	3	20	1.0%	1.29 [0.27, 6.16]	2010
Qu, Y.2010	4	40	2	40	0.6%	2.11 [0.36, 12.24]	2010
Zhang, C. 2011	13	19	4	19	0.4%	8.13 [1.87, 35.23]	2011
Qin, Q.2012	19	34	6	34	0.9%	5.91 [1.94, 17.97]	2012
Xing, Q.2012	7	19	3	19	0.7%	3.11 [0.66, 14.60]	2012
Xu, J.2012	14	27	9	27	1.5%	2.15 [0.72, 6.47]	2012
Xu, Q.2012	11	27	9	27	1.8%	1.38 [0.45, 4.17]	2012
Yu, D.2012	17	25	12	25	1.3%	2.30 [0.73, 7.27]	2012
Du, Y.2013	31	60	22	60	3.7%	1.85 [0.89, 3.83]	2013
Li, L. 2013	4	15	0	15	0.1%	12.13 [0.59, 248.49]	2013
Li, W. 2013	13	30	11	30	2.2%	1.32 [0.47, 3.72]	2013
Yu, L. 2013	2	23	1	22	0.3%	2.00 [0.17, 23.78]	2013
Chen, J. 2014	6	30	2	30	0.6%	3.50 [0.65, 18.98]	2014
Tu, D.2014	14	38	14	38	3.1%	1.00 [0.39, 2.54]	2014
Yao, X.2014	13	25	7	25	1.2%	2.79 [0.86, 9.01]	2014
Zhang, J.2014	13	40	6	40	1.4%	2.73 [0.92, 8.13]	2014
Zhao, Y. 2014	25	40	16	40	2.1%	2.50 [1.02, 6.15]	2014
Zhang, Z.2015	28	45	17	45	2.2%	2.71 [1.16, 6.36]	2015
Zhou, Z.2015	6	21	1	21	0.2%	8.00 [0.87, 73.68]	2015
Cai,C.2015	11	21	6	21	1.0%	2.75 [0.77, 9.86]	2015
Liu, J. 2015	15	30	7	29	1.2%	3.14 [1.03, 9.55]	2015
Li,D.2016	27	40	19	40	2.1%	2.30 [0.93, 5.69]	2016
Yan, L.2016	16	33	7	28	1.3%	2.82 [0.95, 8.44]	2016
Yu, M.2017	7	20	3	20	0.7%	3.05 [0.66, 14.14]	2017
Luo, D.2018	12	30	6	30	1.2%	2.67 [0.84, 8.46]	2018
Wang, Y. 2018	20	37	17	37	2.7%	1.38 [0.55, 3.45]	2018
Liu, Y. 2019	28	50	7	50	1.1%	7.82 [2.95, 20.72]	2019
Wu, Y. 2019	15	46	7	46	1.6%	2.70 [0.98, 7.43]	2019
**Subtotal (95% CI)**		**1177**		**1163**	**52.7%**	**2.37 [1.98, 2.85]**	
Total events	510		297				
Heterogeneity: Chi² = 26.59, df = 30 (P = 0.64); I² = 0%							
Test for overall effect: Z = 9.30 (P < 0.00001)							
**Total (95% CI)**		**2486**		**2333**	**100.0%**	**2.59 [2.28, 2.95]**	
Total events	1045		531				
Heterogeneity: Chi² = 52.12, df = 76 (P = 0.98); I² = 0%							
Test for overall effect: Z = 14.40 (P < 0.00001)							
Test for subgroup differences: Chi² = 1.85, df = 1 (P = 0.17), I² = 46.0%							

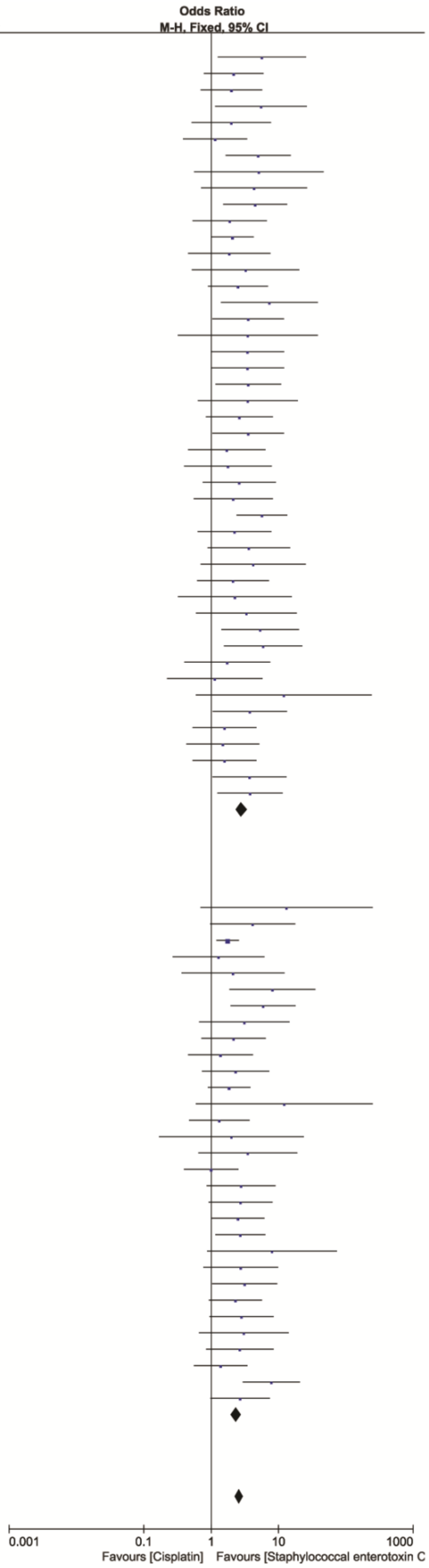

Fig.S62 Subgroups analysis of complete response via publication year

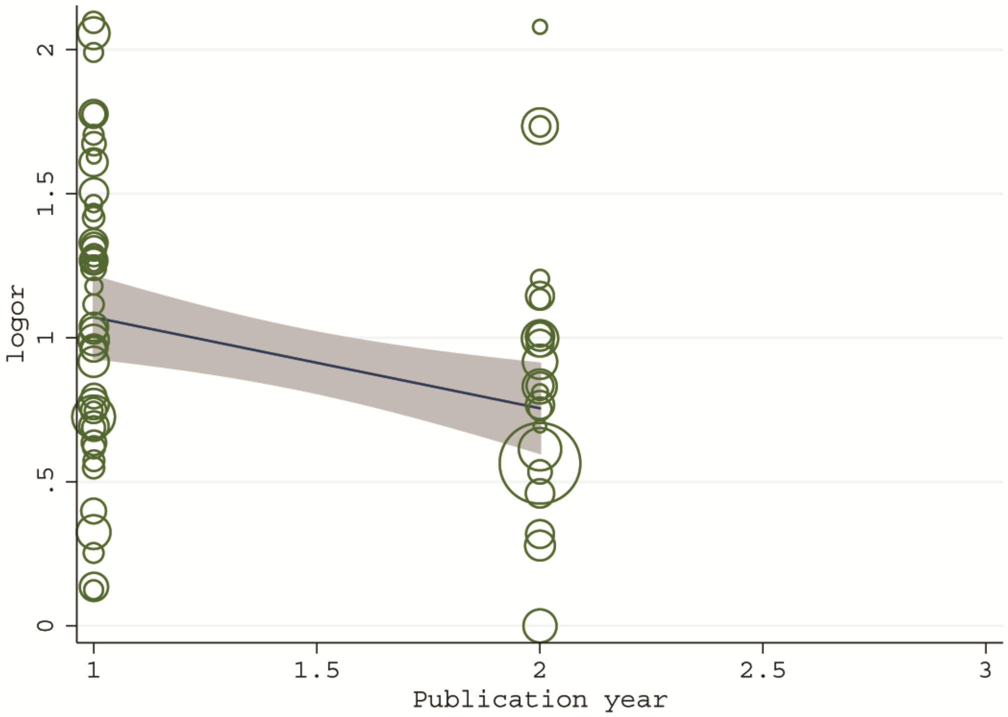

Fig.S63 Meta regression of complete response via publication year

Study or Subgroup	Staphylococcal enterotoxin C Events	Staphylococcal enterotoxin C Total	Cisplatin Events	Cisplatin Total	Weight	Odds Ratio M-H, Fixed, 95% CI	Year
**Before 2010 year**							
Li, S.1997	3	20	11	20	1.0%	0.14 [0.03, 0.65]	1997
Li, H.1998	5	68	23	42	2.8%	0.07 [0.02, 0.20]	1998
Qiu, Y.1999	7	42	17	42	1.5%	0.29 [0.11, 0.81]	1999
Zhang, Q.1999	1	15	2	15	0.2%	0.46 [0.04, 5.75]	1999
Wang, L. 2002	5	29	12	29	1.1%	0.30 [0.09, 0.99]	2000
Xu, N.2000	6	34	12	30	1.1%	0.32 [0.10, 1.01]	2000
Zhang, G.2000	4	36	12	30	1.2%	0.19 [0.05, 0.67]	2000
Cao, H.2000	7	26	11	18	1.0%	0.23 [0.06, 0.85]	2000
Jia, X.2000	2	15	8	15	0.7%	0.13 [0.02, 0.82]	2000
Lang, F.2001	6	56	12	21	1.7%	0.09 [0.03, 0.30]	2001
Wang, Lei.2002	3	21	8	19	0.8%	0.23 [0.05, 1.05]	2002
Xu, Q.2003	4	32	10	30	1.0%	0.29 [0.08, 1.04]	2003
Zhang, J.2003	4	25	8	28	0.7%	0.48 [0.12, 1.83]	2003
Duan, R.2003	10	76	37	76	3.4%	0.16 [0.07, 0.36]	2003
Li, J.2003	4	23	10	21	0.9%	0.23 [0.06, 0.92]	2003
Wang, C.2003	3	15	7	15	0.6%	0.29 [0.06, 1.45]	2003
Hu, Y.2004	4	23	10	22	0.9%	0.25 [0.06, 0.99]	2004
Wang, C. 2004	5	23	12	22	1.0%	0.23 [0.06, 0.85]	2004
Zhu, F.2004	6	34	15	30	1.4%	0.21 [0.07, 0.67]	2004
Chen, H.2004	3	24	10	24	0.9%	0.20 [0.05, 0.86]	2004
Mao, W. 2004	4	24	7	16	0.7%	0.26 [0.06, 1.11]	2004
Fang, X.2004	3	15	7	15	0.6%	0.29 [0.06, 1.45]	2004
Guan, C.2004	4	23	10	22	0.9%	0.25 [0.06, 0.99]	2004
Zhang, T. 2005	4	23	10	21	0.9%	0.23 [0.06, 0.92]	2005
Chen, J.2005	2	27	7	24	0.7%	0.19 [0.04, 1.05]	2005
Feng, X.2005	2	17	7	17	0.7%	0.19 [0.03, 1.11]	2005
Huang, Y.2005	4	27	9	21	0.9%	0.23 [0.06, 0.91]	2005
Liu, M.2005	3	50	21	48	2.2%	0.08 [0.02, 0.30]	2005
Liu, S.2005	5	32	14	29	1.3%	0.20 [0.06, 0.66]	2005
Ma, D.2005	3	24	10	24	0.9%	0.20 [0.05, 0.86]	2005
Fang, H.2006	4	24	11	22	1.0%	0.20 [0.05, 0.78]	2006
Pan, Y.2006	3	28	10	20	1.1%	0.12 [0.03, 0.53]	2006
Wang, J.2006	4	30	7	30	0.6%	0.51 [0.13, 1.95]	2006
Xiong, C.2006	4	22	12	22	1.1%	0.19 [0.05, 0.73]	2006
Yue, Z.2006	5	33	13	28	1.3%	0.21 [0.06, 0.69]	2006
Zhang, Z.2006	2	16	9	16	0.8%	0.11 [0.02, 0.66]	2006
Zhao, Z.2006	5	32	14	32	1.3%	0.24 [0.07, 0.78]	2006
Chen, Z.2006	3	12	6	11	0.5%	0.28 [0.05, 1.62]	2006
Zhou, F.2007	2	16	7	12	0.7%	0.10 [0.02, 0.66]	2007
Sun, C.2007	3	24	12	24	1.1%	0.14 [0.03, 0.61]	2007
Yin, W.2007	3	19	10	19	0.9%	0.17 [0.04, 0.78]	2007
Zheng, F.2007	6	24	8	20	0.7%	0.50 [0.14, 1.81]	2007
Huang, W.2008	5	28	12	28	1.1%	0.29 [0.09, 0.98]	2008
Li, X.2008	4	25	11	25	1.0%	0.24 [0.06, 0.92]	2008
Zhang, M.2008	3	33	13	34	1.2%	0.16 [0.04, 0.64]	2008
Gao, H.2009	7	35	17	30	1.6%	0.19 [0.06, 0.57]	2009
Wang, H.2009	5	28	12	28	1.1%	0.29 [0.09, 0.98]	2009
Zhang, L.2009	3	34	12	35	1.2%	0.19 [0.05, 0.73]	2009
**Subtotal (95% CI)**		**1362**		**1222**	**52.2%**	**0.21 [0.17, 0.25]**	
Total events	197		545				
Heterogeneity: Chi² = 19.39, df = 47 (P = 1.00); I² = 0%							
Test for overall effect: Z = 16.22 (P < 0.00001)							
**From 2010 to now**							
Cheng, J.2010	6	30	15	30	1.3%	0.25 [0.08, 0.79]	2010
Gui, Q. 2010	3	25	11	25	1.0%	0.17 [0.04, 0.73]	2010
Li, Z. 2010	50	230	110	230	9.2%	0.30 [0.20, 0.45]	2010
Liu, Q.2010	5	27	9	20	0.9%	0.28 [0.07, 1.03]	2010
Qu, Y.2010	8	40	19	40	1.6%	0.28 [0.10, 0.75]	2010
Zhang, C. 2011	1	19	9	19	0.9%	0.06 [0.01, 0.56]	2011
Xing, Q.2012	2	19	12	19	1.1%	0.07 [0.01, 0.39]	2012
Xu, J.2012	3	27	12	27	1.1%	0.16 [0.04, 0.65]	2012
Xu, Q.2012	4	27	9	27	0.8%	0.35 [0.09, 1.31]	2012
Yu, D.2012	3	25	7	25	0.7%	0.35 [0.08, 1.55]	2012
Qin, Q.2012	4	34	23	34	2.2%	0.06 [0.02, 0.23]	2012
Du, Y.2013	6	60	25	60	2.4%	0.16 [0.06, 0.42]	2013
Li, L. 2013	3	15	7	15	0.6%	0.29 [0.06, 1.45]	2013
Li, W. 2013	3	30	10	30	1.0%	0.22 [0.05, 0.91]	2013
Yu, L. 2013	4	23	11	22	1.0%	0.21 [0.05, 0.82]	2013
Zhang, J.2014	8	40	25	40	2.1%	0.15 [0.05, 0.41]	2014
Zhao, Y. 2014	2	40	13	40	1.3%	0.11 [0.02, 0.52]	2014
Chen, J. 2014	5	30	15	30	1.3%	0.20 [0.06, 0.66]	2014
Tu, D.2014	4	38	13	38	1.2%	0.23 [0.07, 0.78]	2014
Yao, X.2014	2	25	12	25	1.2%	0.09 [0.02, 0.49]	2014
Cai,C.2015	3	21	7	21	0.6%	0.33 [0.07, 1.53]	2015
Liu, J. 2015	4	30	13	29	1.2%	0.19 [0.05, 0.68]	2015
Zhang, Z.2015	2	45	18	45	1.8%	0.07 [0.01, 0.32]	2015
Zhou, Z.2015	4	21	11	21	1.0%	0.21 [0.05, 0.85]	2015
Li,D.2016	1	40	10	40	1.0%	0.08 [0.01, 0.63]	2016
Yan, L.2016	4	33	8	28	0.8%	0.34 [0.09, 1.30]	2016
Yu, M.2017	2	20	13	20	1.3%	0.06 [0.01, 0.34]	2017
Luo, D.2018	3	30	14	30	1.3%	0.13 [0.03, 0.51]	2018
Wang, Y. 2018	4	37	10	37	1.0%	0.33 [0.09, 1.16]	2018
Liu, Y. 2019	2	50	23	50	2.4%	0.05 [0.01, 0.22]	2019
Wu, Y. 2019	10	46	27	46	2.3%	0.20 [0.08, 0.49]	2019
**Subtotal (95% CI)**		**1177**		**1163**	**47.8%**	**0.19 [0.16, 0.24]**	
Total events	165		521				
Heterogeneity: Chi² = 24.04, df = 30 (P = 0.77); I² = 0%							
Test for overall effect: Z = 15.70 (P < 0.00001)							
**Total (95% CI)**		**2539**		**2385**	**100.0%**	**0.20 [0.18, 0.23]**	
Total events	362		1066				
Heterogeneity: Chi² = 43.41, df = 78 (P = 1.00); I² = 0%							
Test for overall effect: Z = 22.57 (P < 0.00001)							
Test for subgroup differences: Chi² = 0.18, df = 1 (P = 0.67), I² = 0%							

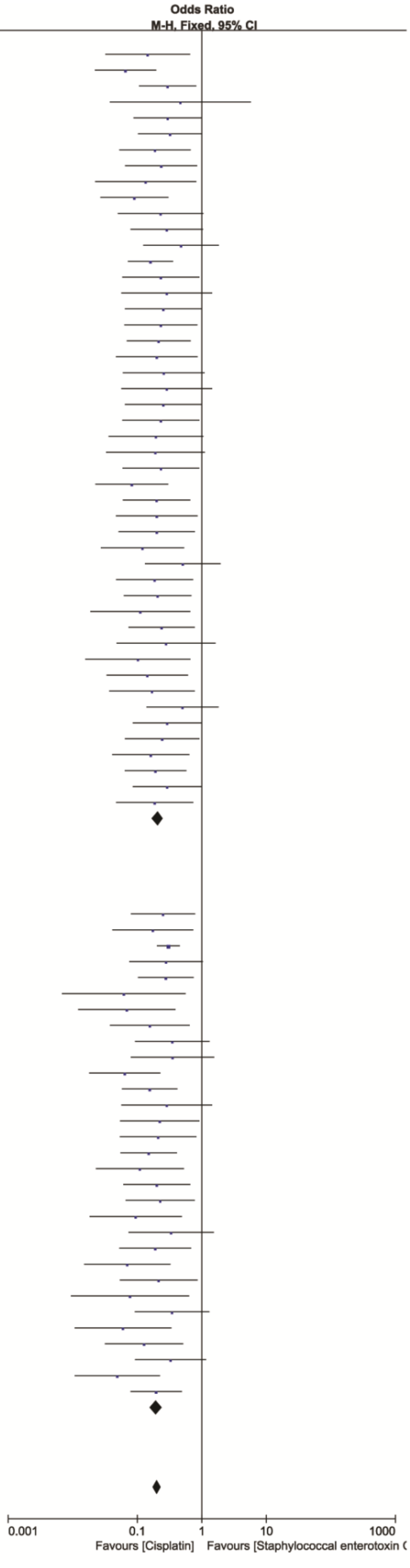

Fig.S64 Subgroups analysis of treatment failure via publication year

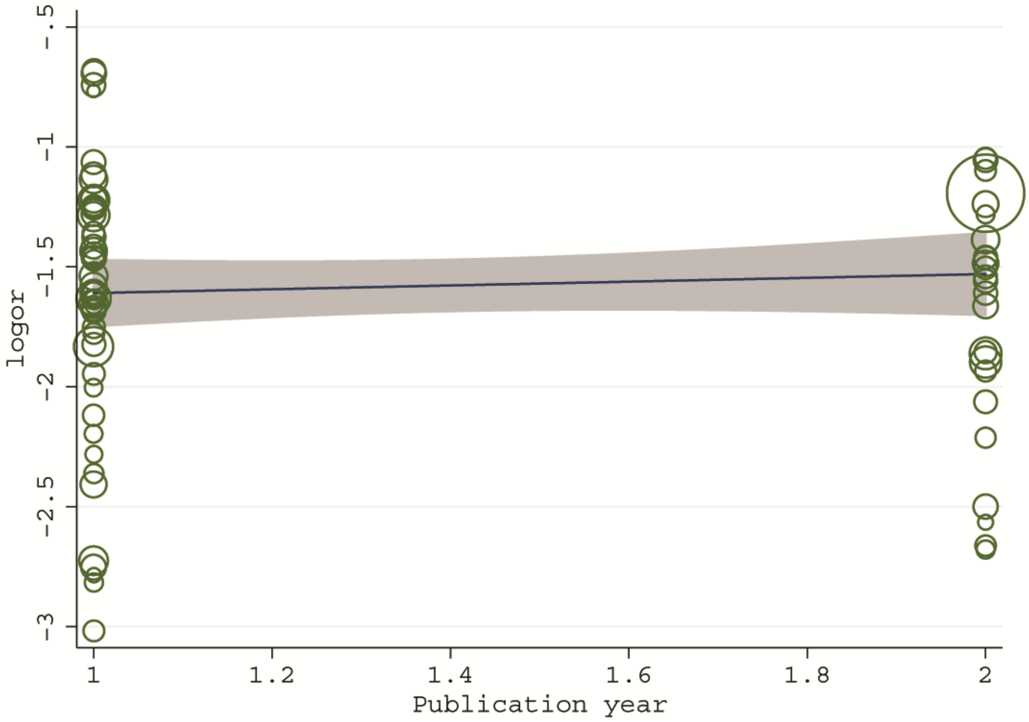

Fig.S65 Meta regression of treatment failure via publication year
