# Supplementary material for: Intrapleural Perfusion With Staphylococcal Enterotoxin C for Malignant Pleural Effusion: A Clustered Systematic Review and Meta-Analysis
Source: Front Med (Lausanne). 2022 Apr 25;9:816973. doi: 10.3389/fmed.2022.816973 (PMC9081816; doi:10.3389/fmed.2022.816973)
Supplement: Supplementary file 6 [file Data_Sheet_6.PDF]

Appendix 6. The publication bias analysis (Figs.S66 to S79)

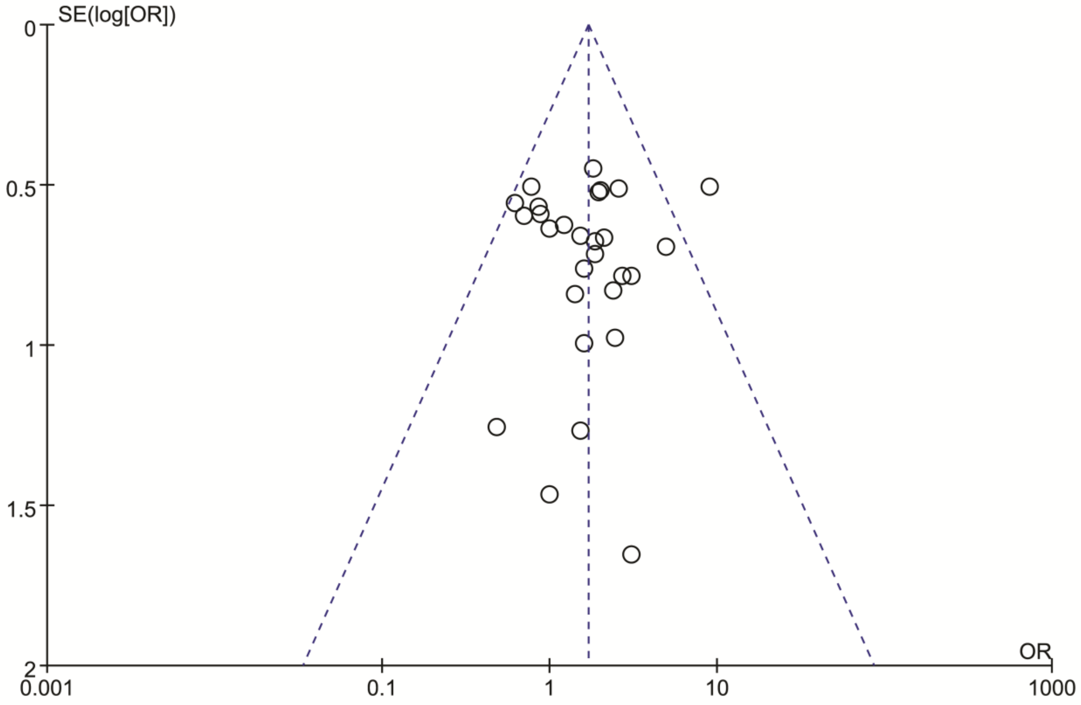

Fig.S66 The funnel plot of complete response in staphylococcal enterotoxin C vs Cisplatin

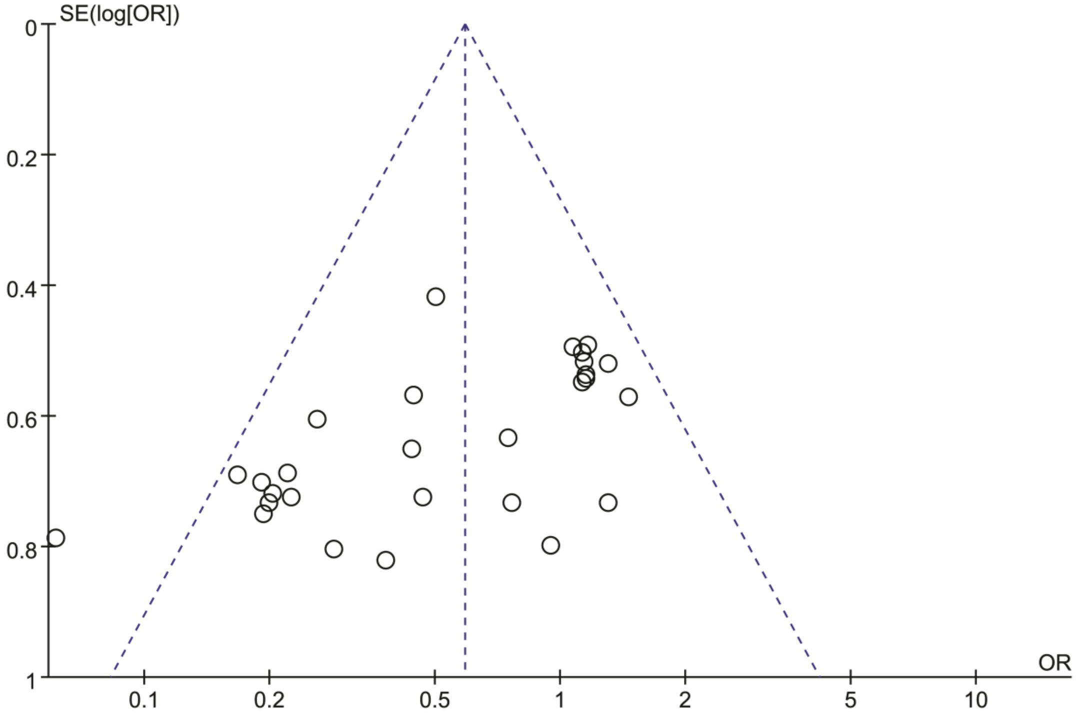

Fig.S67 The funnel plot of treatment failure in staphylococcal enterotoxin C vs Cisplatin

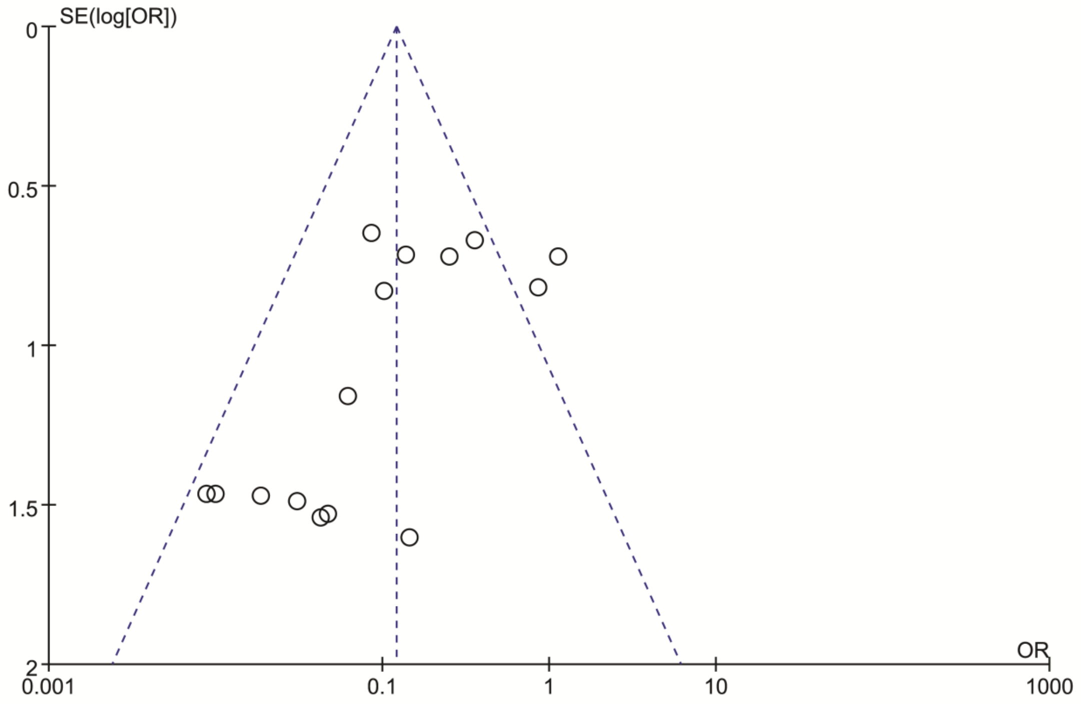

Fig.S68 The funnel plot of gastrointestinal reactions in staphylococcal enterotoxin C vs Cisplatin

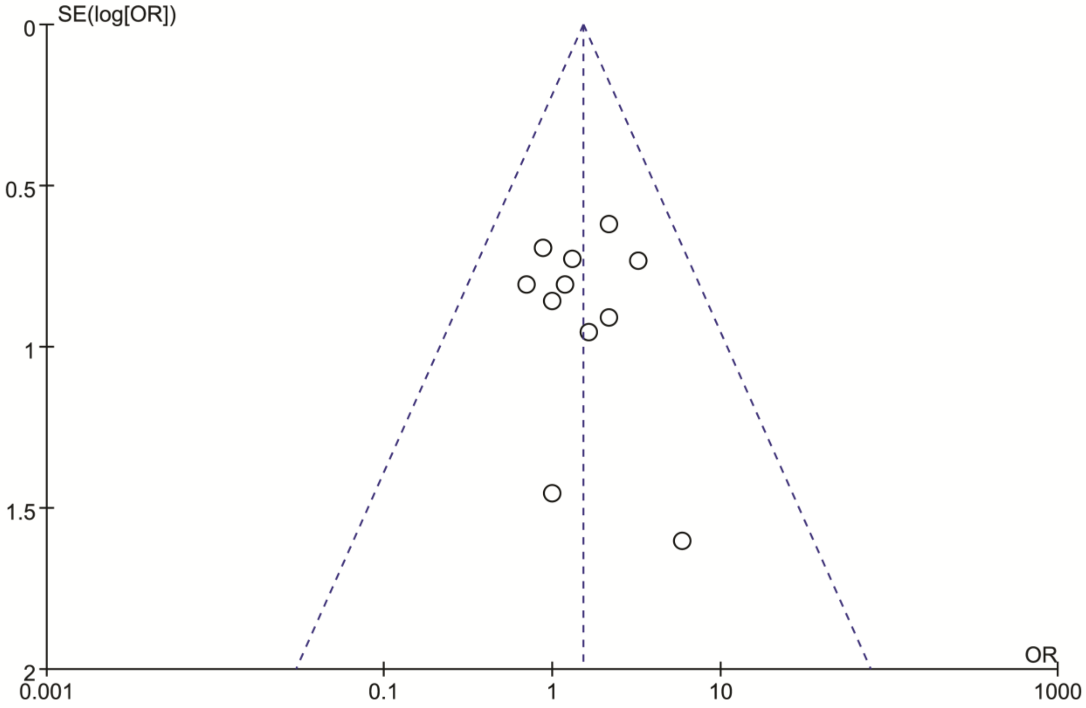

Fig.S69 The funnel plot of thoracodynia in staphylococcal enterotoxin C vs Cisplatin

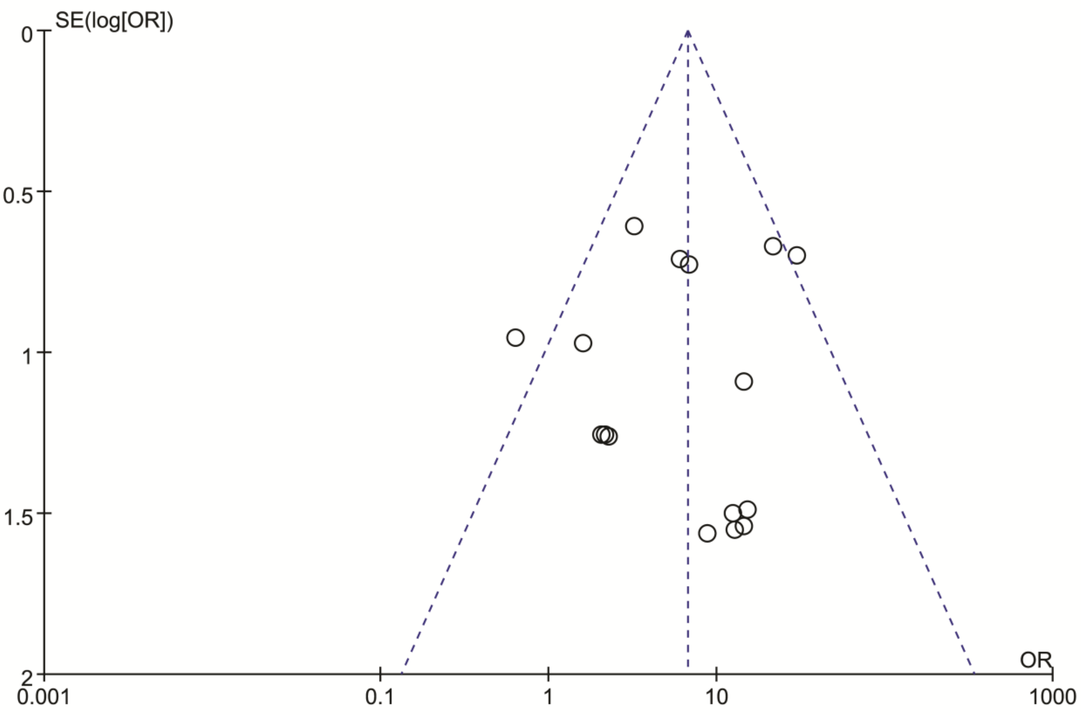

Fig.S70 The funnel plot of fever in staphylococcal enterotoxin C vs DDP

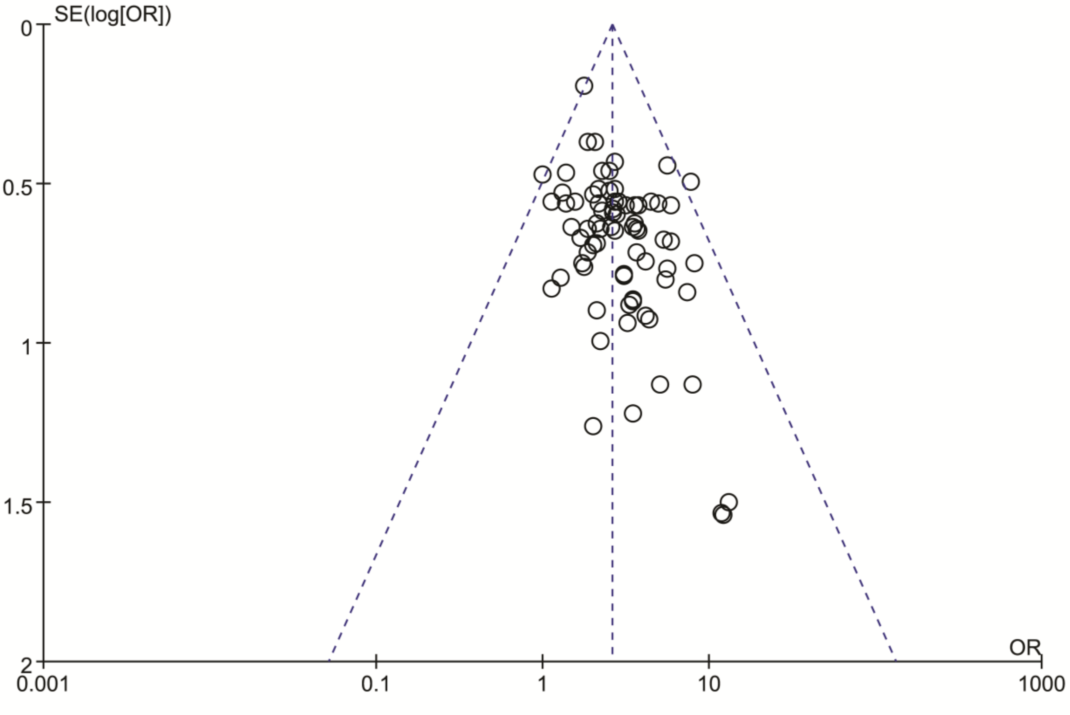

Fig.S71 The funnel plot of complete response in staphylococcal enterotoxin C plus Cisplatin

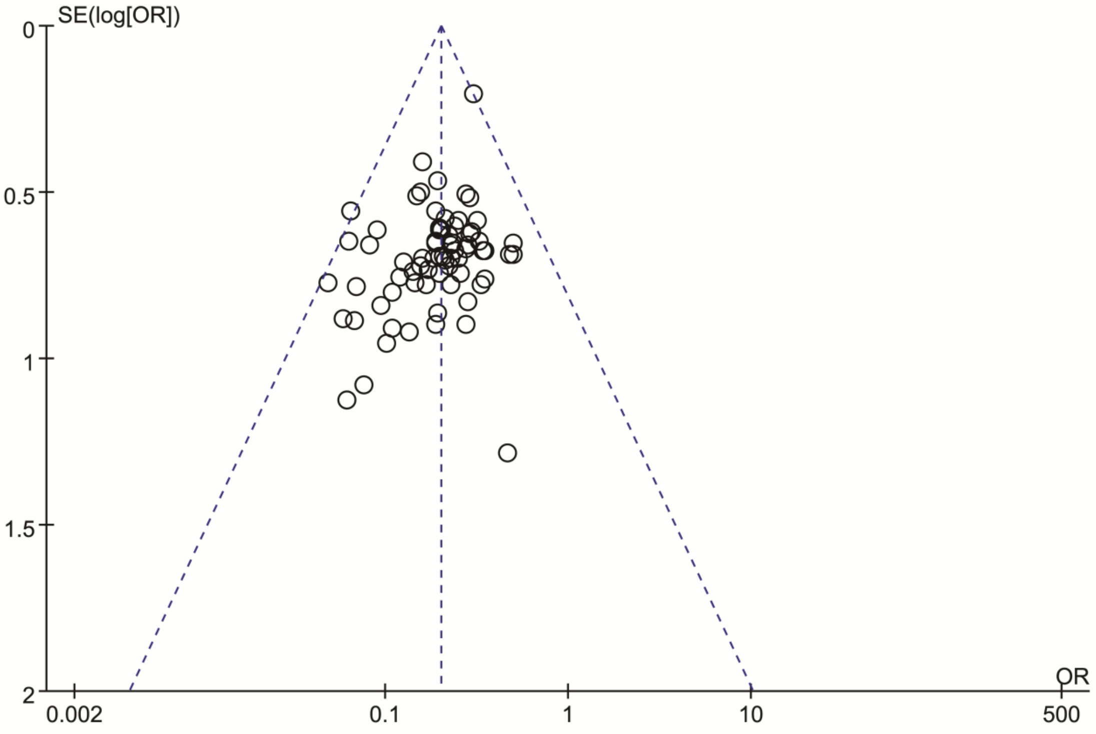

Fig.S72 The funnel plot of treatment failure in staphylococcal enterotoxin C plus Cisplatin

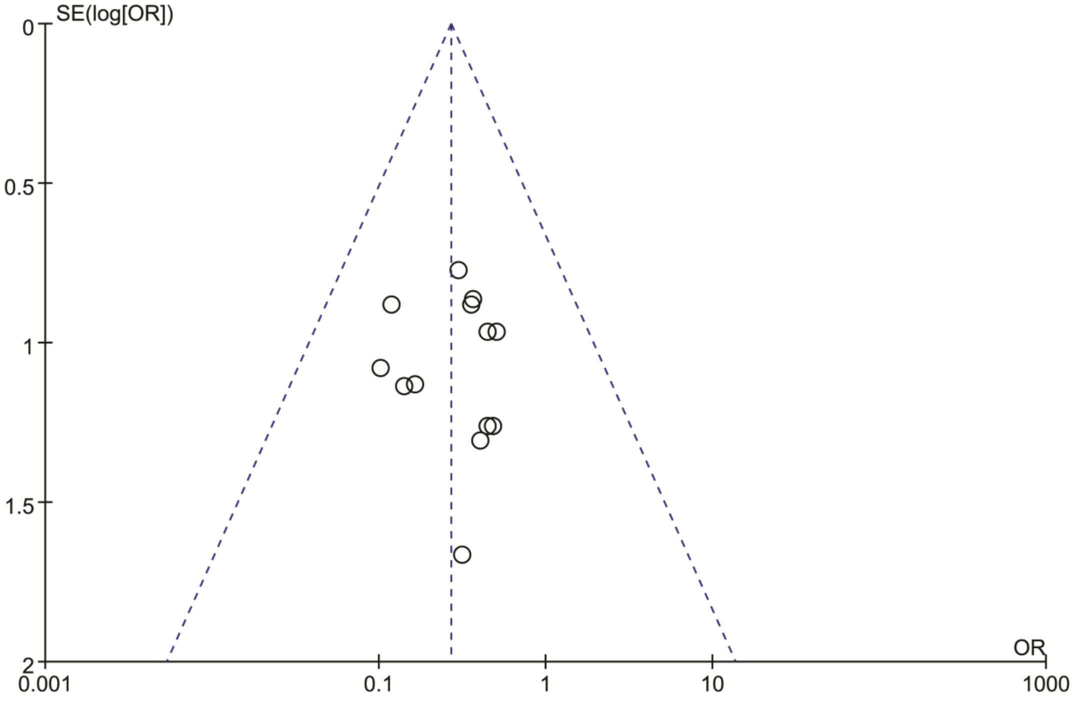

Fig.S73 The funnel plot of disease progression in staphylococcal enterotoxin C plus Cisplatin

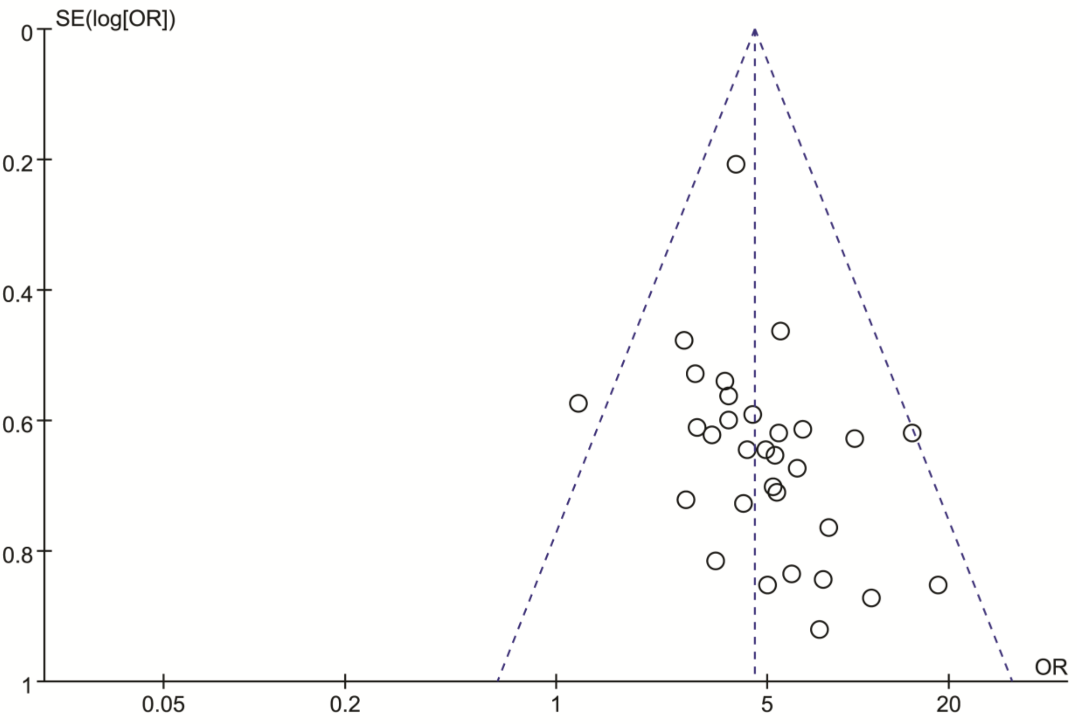

Fig.S74 The funnel plot of quality of life in staphylococcal enterotoxin C plus Cisplatin

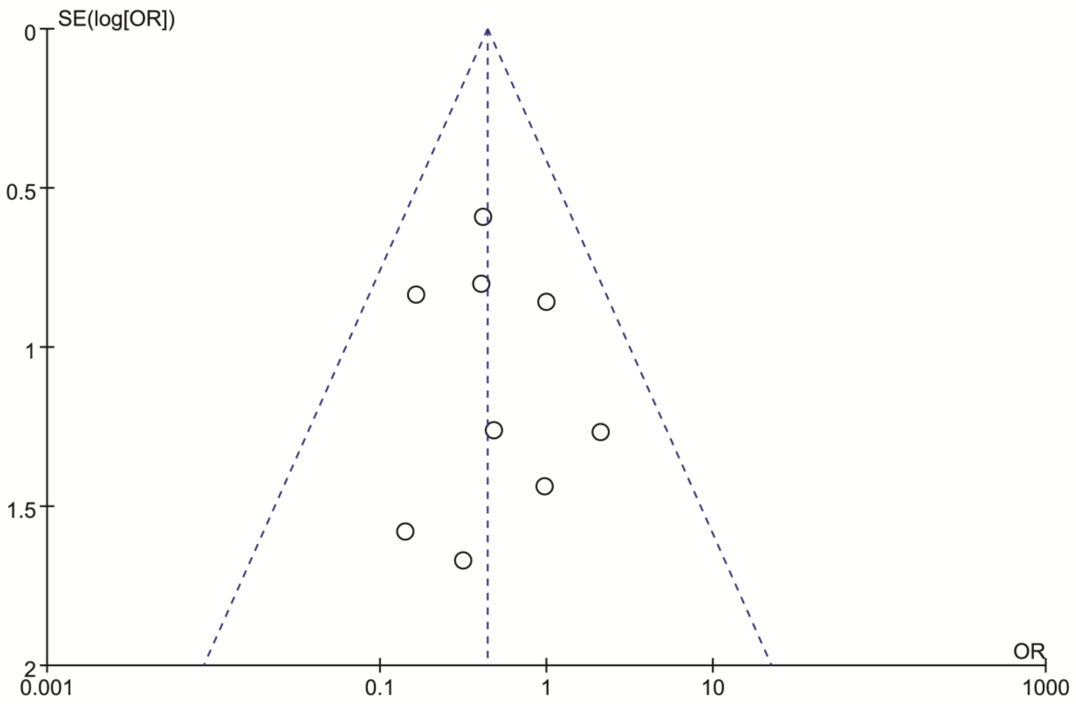

Fig.S75 The funnel plot of myelosuppression in staphylococcal enterotoxin C plus Cisplatin

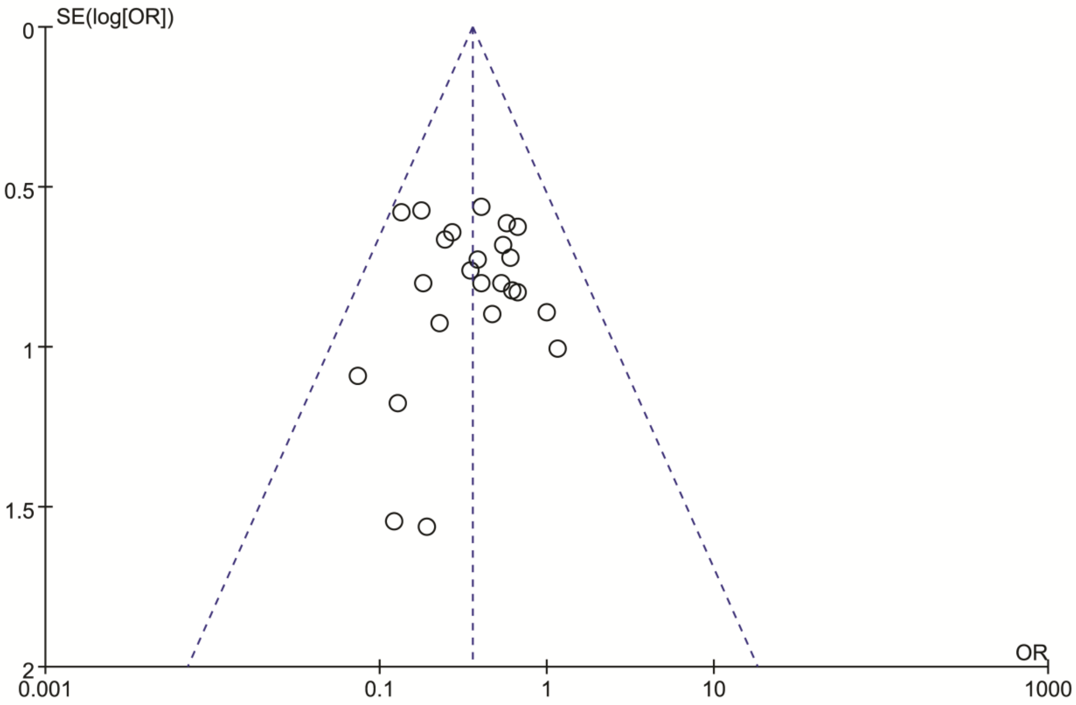

Fig.S76 The funnel plot of leukopenia in staphylococcal enterotoxin C plus Cisplatin (DDP)

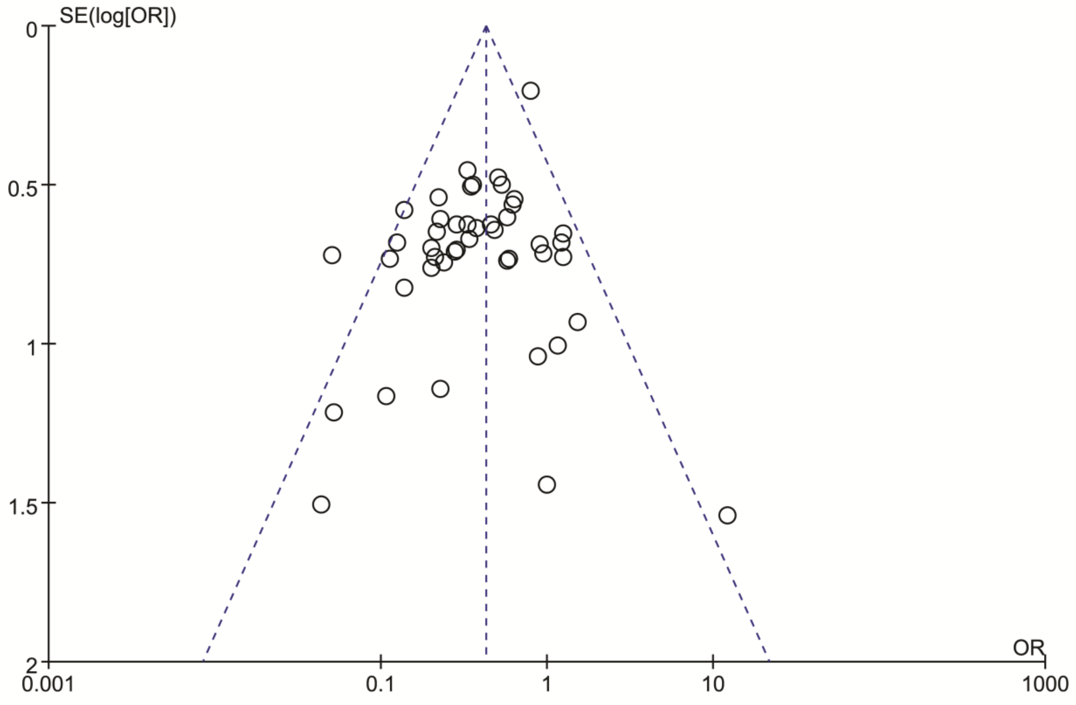

Fig.S77 The funnel plot of gastrointestinal reactions in staphylococcal enterotoxin C plus Cisplatin

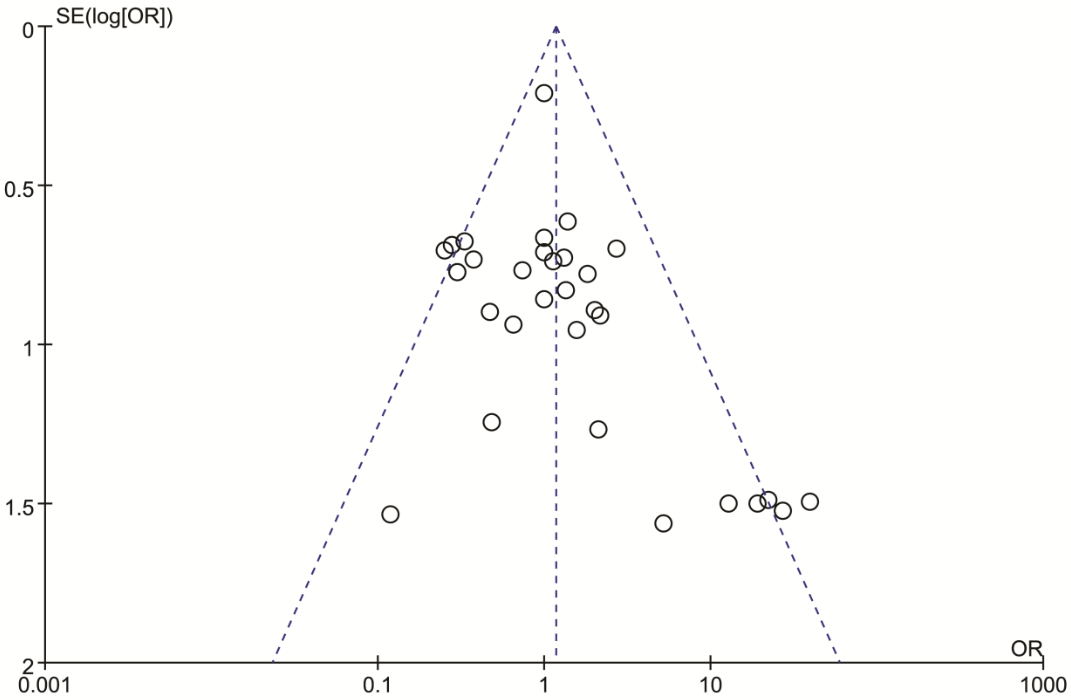

Fig.S78 The funnel plot of thoracodynia in staphylococcal enterotoxin C plus Cisplatin

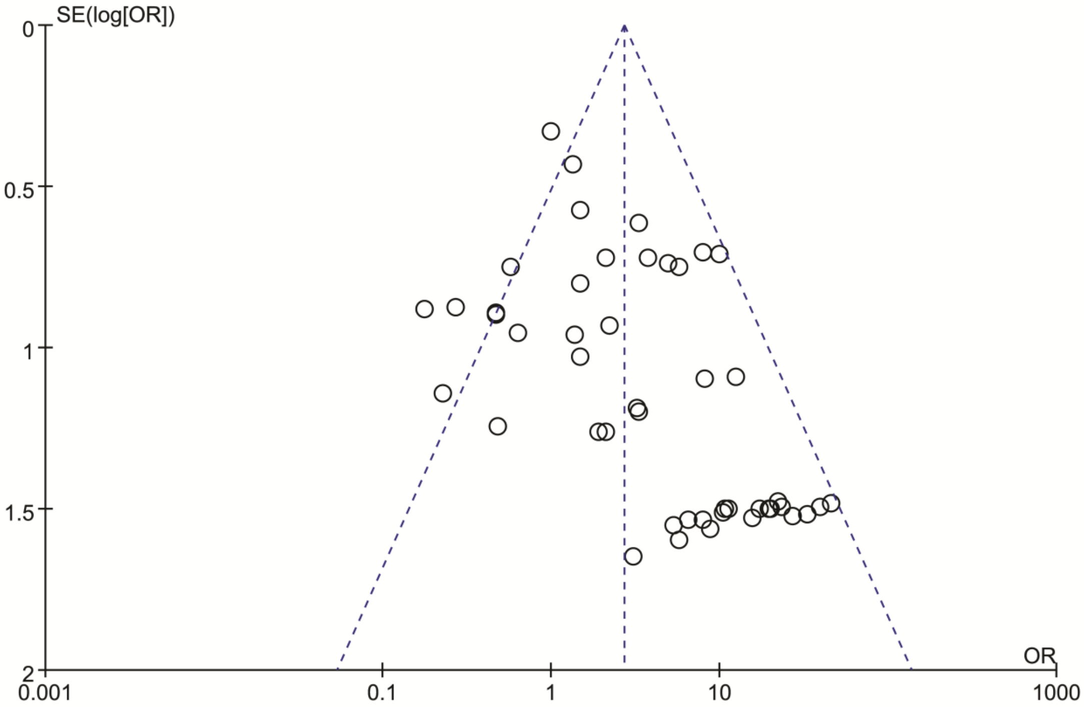

Fig.S79 The funnel plot of fever in staphylococcal enterotoxin C plus Cisplatin
